# Enantioselective acyl transfer catalysis by a combination of common catalytic motifs and electrostatic interactions

**DOI:** 10.1038/ncomms11297

**Published:** 2016-04-15

**Authors:** Hiroki Mandai, Kazuki Fujii, Hiroshi Yasuhara, Kenko Abe, Koichi Mitsudo, Toshinobu Korenaga, Seiji Suga

**Affiliations:** 1 Division of Applied Chemistry, Graduate School of Natural Science and Technology, Okayama University, 3-1-1 Tsushima-naka, Kita-ku, Okayama 700-8530, Japan; 2 Faculty of Engineering, Department of Chemistry and Bioengineering, Iwate University, Morioka, Iwate 020-8551, Japan; 3 Research Center of New Functional Materials for Energy Production, Storage and Transport Okayama University, 3-1-1 Tsushima-naka, Kita-ku, Okayama 700-8530, Japan; 4 Japan Science and Technology Agency, ACT-C, 4-1-8 Honcho, Kawaguchi, Saitama 332-0012, Japan

## Abstract

Catalysts that can promote acyl transfer processes are important to enantioselective synthesis and their development has received significant attention in recent years. Despite noteworthy advances, discovery of small-molecule catalysts that are robust, efficient, recyclable and promote reactions with high enantioselectivity can be easily and cost-effectively prepared in significant quantities (that is, >10 g) has remained elusive. Here, we demonstrate that by attaching a binaphthyl moiety, appropriately modified to establish H-bonding interactions within the key intermediates in the catalytic cycle, and a 4-aminopyridyl unit, exceptionally efficient organic molecules can be prepared that facilitate enantioselective acyl transfer reactions. As little as 0.5 mol% of a member of the new catalyst class is sufficient to generate acyl-substituted all-carbon quaternary stereogenic centres in quantitative yield and in up to 98:2 enantiomeric ratio (er) in 5 h. Kinetic resolution or desymmetrization of 1,2-diol can be performed with high efficiency and enantioselectivity as well.

## Abstract (web summary)

Nucleophilic catalysts are widely used for acyl transfer reactions, but chiral variants can be difficult to design or synthesise. Here, the authors report catalysts with chirality imparted from a binaphtyl moiety with tert-alcohol unit that show both high activity and enantioselectivity for a range of acyl transfer reactions.

## Graphical abstract

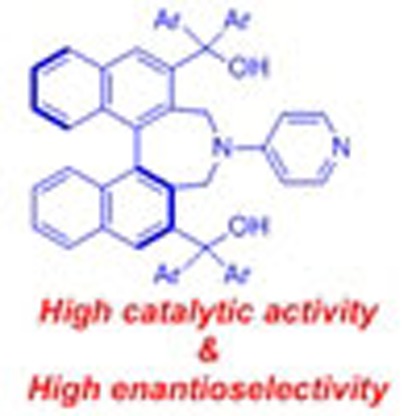

N,*N*-4-dimethylaminopyridine (DMAP) has long been recognized as a uniquely effective and broadly applicable nucleophilic catalyst for a number of important reactions in organic chemistry^1^. One set of transformations for which DMAP is commonly utilized is the acylation of hydroxyl group in the presence of acid anhydride. The commonly accepted mechanism for this class of reactions, which supported by experimental as well as computational findings, is presented in Fig. 1a (refs 2, 3). The nucleophilic is believed to react first with the acylating agent **i** to generate *N*-acylpyridinium salt intermediate **ii**, which is likely subjected to nucleophilic attack by the alcohol substrate (R^2^OH) via intermediate **iii**. A critical feature of this general class of catalytic processes is that the acetate anion that resides within complex **ii** serves as a Brønsted base to enhance the reactivity of the otherwise relatively mildly nucleophilic hydroxyl unit. Ester product **iv** and pyridinium salt **v** are thus formed, and the latter is then neutralized by the stoichiometric base (B; typically Et_3_N) to re-generate the nucleophilic catalyst. The efficiency with which *N*-acylpyridinium ion **ii** is generated^4^, the Lewis basicity of the counteranion unit as well as the degree to which it remains associated with the positively charged acylating agent (versus loose ion pair) are critical to the facility of the overall transformation^5^. A number of strategies have been adopted to modulate the effectiveness of acyl transfer agents for which DMAP serves as the parent compound. Among these is the utilization of electronic factors to extend the lifetime of the *N*-acylpyridinium salt, or by manipulation of conformational effects to enhance electron donation by the amino substituent; there are also instances where a combination of the aforementioned approaches has been adopted (for example, 4-pyrrolidinopyridine (PPY) or 9-azajulolidine)^6^. A variety of chiral variants, employed for kinetic resolution of alcohol or enantioselective acyl transfer processes, have also been introduced^7^,^8^. Such investigations, which have led to the development of various enantiomerically pure promoter molecules that are based on DMAP or PPY scaffolds, as originally put forth by the notable advances reported by Vedejs^9^ and Fu^10^, may be classified in four major categories (Fig. 1b–e). One strategy entailed the use of *N*-acylpyridinium salt with a chiral substituent at the pyridyl ring's C2 position; however, this structural alteration proved to be detrimental to reaction efficiency, requiring the use of stoichiometric amounts of the catalyst (Fig. 1b)^9^. A similar approach but involving the C3 site of the heterocyclic ring, has been extensively examined^11^,^12^,^13^,^14^,^15^,^16^,^17^,^18^,^19^. Nonetheless, reactivity levels were again generally reduced, probably as a result of hampering of proper electron donation by the amino substituent, which raises the energy of the critical pyridinium ion intermediates (cf. **ii**, Fig. 1a)^20^,^21^. The issue of diminished catalyst efficiency applies to DMAP or PPY derivatives that carry a ring that connects the C2 and C3 carbons of the pyridyl ring (Fig. 1d)^22^. In most cases, diminished stability of the key ion-pair intermediate because of steric repulsion between substituents and 4-amino moiety and/or *N*-acetyl group was a complication^21^. An exception was the ferrocene-based catalysts developed by Fu *et al*.^23^,^24^; comparatively high catalyst activity and enantioselectivity was observed in a number of different applications. Obtaining this set of chiral catalysts in the enantiomerically pure form, however, requires costly resolution procedures^25^. The efficiency and considerable longevity of *N*-acylpyridinium ion has been attributed to the exceptional electron-donating ability of transition metal framework; what's more, the cyclopentadienyl moiety attached to pyridyl ring is sufficiently small to prevent unfavourable interaction with the *N*-acetyl and/or the *N*-dimethyl- and pyrrolidino groups^21^. Chiral amino derivatives have been positioned at the pyridyl group's C4 site as well (Fig. 1e)^26^,^27^,^28^,^29^, but these distally positioned moieties did not generate high degrees of stereochemical differentiation for a wide variety of substrates and, for electronic reasons already mentioned (cf. Fig. 1c), this came at the cost of significant diminution in efficiency. Thus, relatively large substituents are required to construct chiral environment to achieve high enantioselectivity. Another notable concept (dual catalysis/anion-binding approach), which is not classified into aforementioned categories (Fig. 1b–e), was also very effective for enantioselective acyl transfer reactions^30^,^31^,^32^,^33^,^34^,^35^,^36^,^37^,^38^.

## Results

### Catalyst design

**Figure 1:**
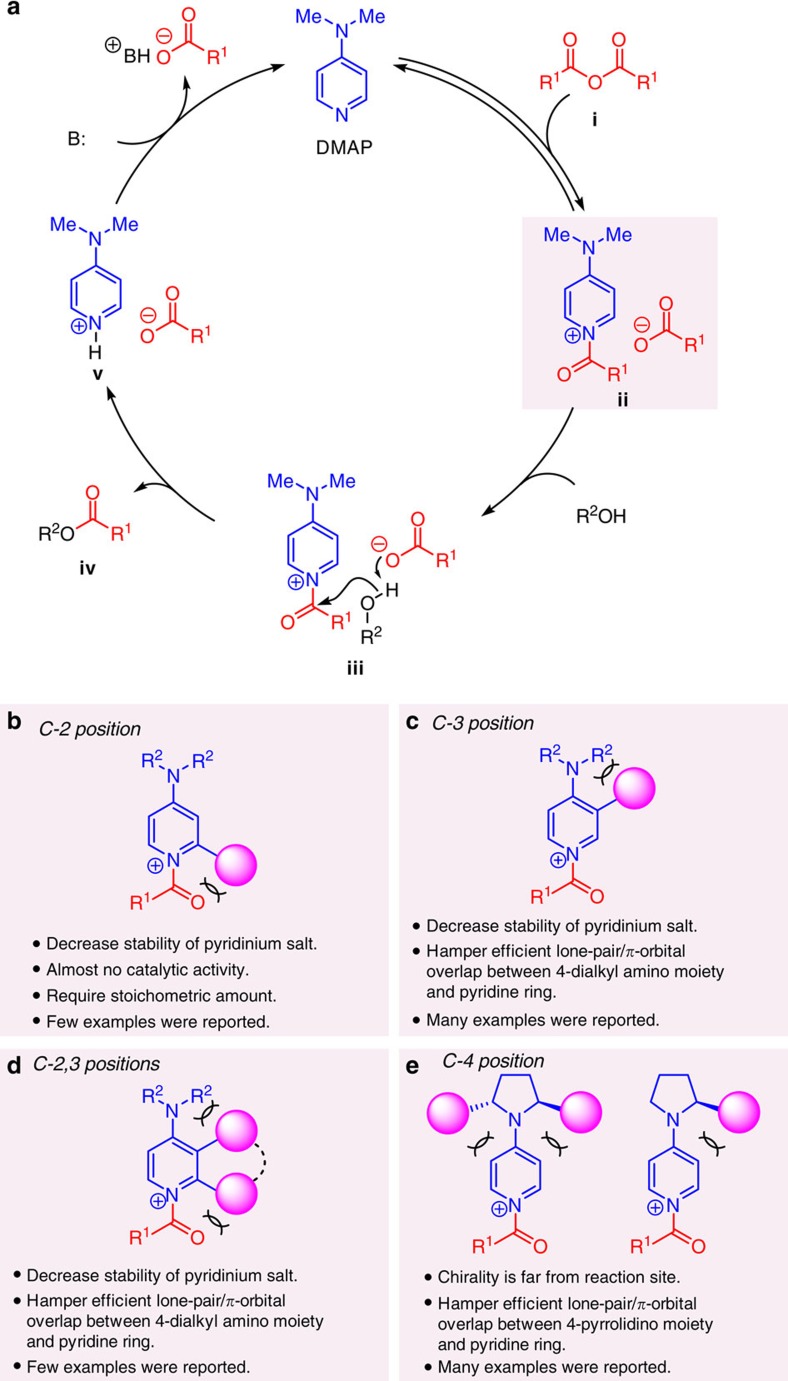
Nucleophilic catalyst in acylation reaction. (**a**) The proposed and generally accepted catalytic cycle for acyl transfer reactions in the presence of acid anhydride promoted by DMAP; this general scheme was used as the framework for the catalyst development studies described in this report. The anionic component within the loose ion-pair intermediate (**ii**) is believed to serve as a general base, playing a critical role in determining the rate of the overall processes. (**b−e**) General strategies, and their specific attributes, of the previously employed strategies involving structural alteration of DMAP-containing molecules to generate enantioselective acyl transfer catalysts. B, base; R^1^, R^2^, FG, various functional groups.

**Figure 2:**
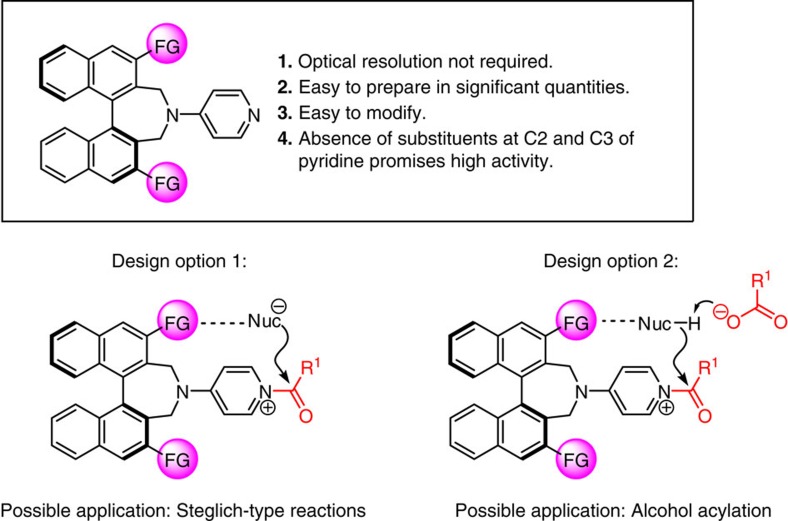
Initial consideration and options in catalyst design. The basic design of the new acyl transfer catalysts, their projected advantages and the possible means by which electrostatic interactions can facilitate transformation. R^1^, FG, various functional groups.

According to our continuous efforts for the development of chiral nucleophilic catalysts^17^,^18^,^19^ to design an efficient and highly enantioselective acyl transfer catalyst that can be prepared in significant quantities without the need for expensive and/or specialized techniques, we envisioned chiral DMAP derivatives that might contain a binaphthyl unit at C4 position of a pyridine ring (Fig. 2). We reasoned that this blueprint would have several noteworthy advantages. First, either enantiomeric form of 1,1′-bi-2-naphthol (BINOL) is inexpensively available, thus obviating resolution procedures. Second, the catalyst platform allows synthesis and screening of *C*_1_- and *C*_2_-symmetric variants from a common intermediate. Third, the proposed catalyst construct easily lends to steric and/or electronic modification through the use of well-established protocols^39^,^40^. Finally, the absence of substituents at the C2 or C3 sites of the pyridyl ring (cf. Fig. 1b,c) would ensure that efficiency levels remain high. The suggested line of attack poses several significant challenges, however. One is that because the source of stereogenicity is somewhat distal from the pyridine ring, an attribute can result in minimal enantioselectivity. Thus, appropriate structural modification would be needed if the stereochemical bias inherent in the binaphthyl moiety is to exert a meaningful role in the acyl bond forming event. Towards this end, preliminary examination of molecular models implied that the substituents (functional group) at C3 and C3′ positions of the binaphthyl moiety are located sufficiently proximal to the reaction site, offering an attractive option for influencing enantioselectivity through catalyst structure alteration. Thus, we surmised that attractive interactions (for example, *π*–*π* stacking, cation-*π* affinity^27^,^41^ or hydrogen-bonding^26^) between an appropriate unit within the catalyst structure and counteranion of *N*-acylpyridinium salt (design option 1, Fig. 2), or the nucleophile (design option 2, Fig. 2) might give rise to enhancement of enantioselectivity and perhaps reaction efficiency. Below, we outline the successful realization of the above plan.

### Catalyst synthesis

**Figure 3:**
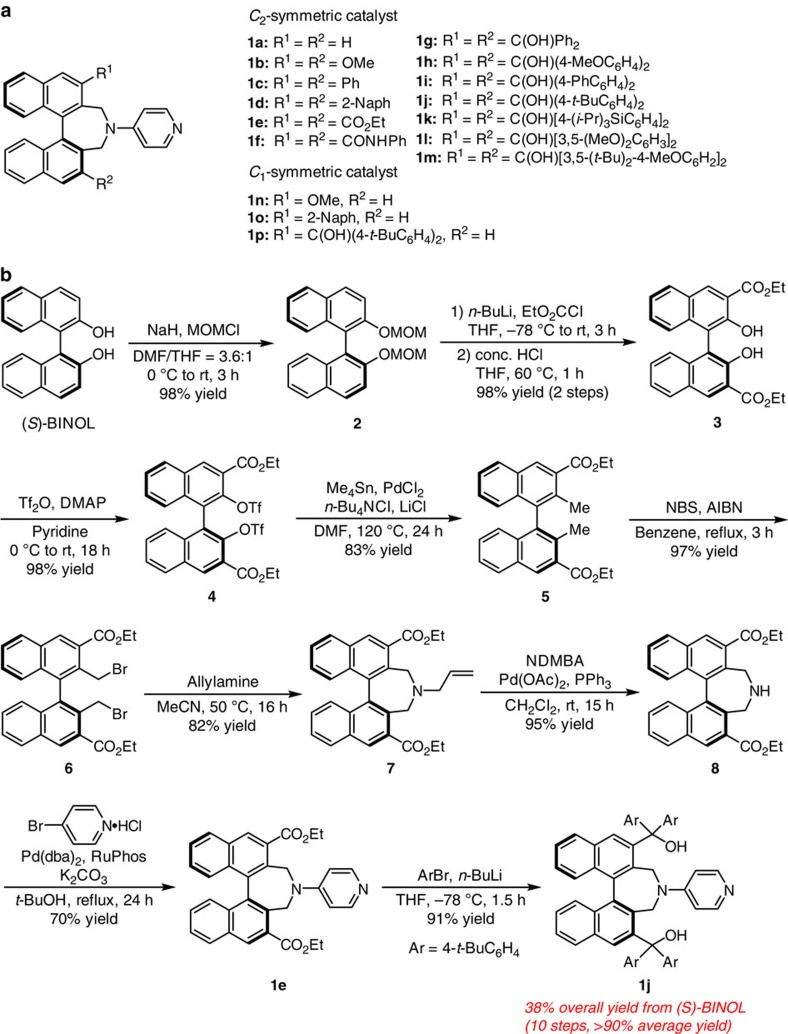
Synthesis of binaphthyl-based chiral nucleophilic catalyst candidates. (**a**) Catalyst libraries containing *C*_2_- and *C*_1_-symmetic catalyst with different substitution pattern were used in the Steglich rearrangement of *O*-acylated oxindole derivatives. (**b**) *C*_2_-symmetric catalyst with polar functional group at 3,3′-positions of binaphthyl moiety can be prepared by a synthesis scheme that is high yielding and is readily amenable to scale-up with minimal column chromatography purification, as the representative example clearly illustrates. AIBN, 2,3′-azodiisobutyronitrile; NBS, *N*-bromosuccinimide; NDMBA, N,N′-dimethylbarbituric acid; RuPhos, 2-dicyclohexylphosphino-2**′**,6**′**-di-i-propoxy-1,1'-biphenyl.

We began by preparing a series of enantiomerically pure DMAP derivatives (**1a**−**p**) that contain a 1,1′-binaphthyl unit with different substituents pattern at their C3 and C3′ sites with reference to chiral quaternary ammonium salt syntheses from BINOL (refs 39, 40; Fig. 3a, Supplementary Figs 1–26 and Supplementary Methods). The representative route for synthesis of catalyst **1j** is presented in Fig. 3b. Ortho-lithiation of MOM-protected compound **2** derived from (*S*)-BINOL with *n*-BuLi, followed by quenching with ethyl chloroformate, and deprotection of MOM group under acidic conditions gave the desired BINOL with 3,3′-diesters **3** in 98% yield over 2 steps (78.9 mmol scale). Then, two hydroxy groups of **3** were converted to the corresponding ditriflate, and subjected to Migita–Kosugi–Stille coupling reaction in the presence of tetra *n*-butyl ammonium chloride and lithium chloride as additives to afford **5** in 83% yield. Our studies revealed that Migita–Kosugi–Stille coupling reaction using a Pd nanoparticle generated *in situ* was found to be superior to Negishi coupling reaction^40^ in large scale reaction because Me_2_Zn is costly and pyrophoric reagent. The benzylic positions of **5** were brominated using *N*-bromosuccinimide in the presence of 2,2′-azodiisobutyronitrile to give the desired dibromide **6** in 97% yield. Seven-membered ring formation from dibromide **6** using allylamine gave rise to the formation of amine **7** in 82% yield, and deprotection of ally group afforded amine **8** in 95% yield. Installation of pyridine ring was accomplished by using Buchwald–Hartwig amination of 4-bromopyridine hydrochoride with amine **8** to provide the key intermediate **1e** in 70% yield. The formation of bis-tertiary alcohols **1j** was readily accomplished by the addition of ArLi to bis-ester **1e** in 91% yield. All transformations can be easily carried out in large scale (>10 g scale for **1e** from BINOL throughout the whole process, and >1 g scale for **1j** from **1e**), and overall yield of **1j** from (*S*)-BINOL was 38% (10 steps, >90% average yield for each step) with only 4 times of silica gel column chromatography purifications. The present synthetic route sufficiently secures facile accessibility to the key catalyst. The structures of **1e** and **1g** were identified by X-ray single-crystal analysis (Supplementary Figs 27 and 28).

### Enantioselective steglich rearrangement

**Figure 4:**
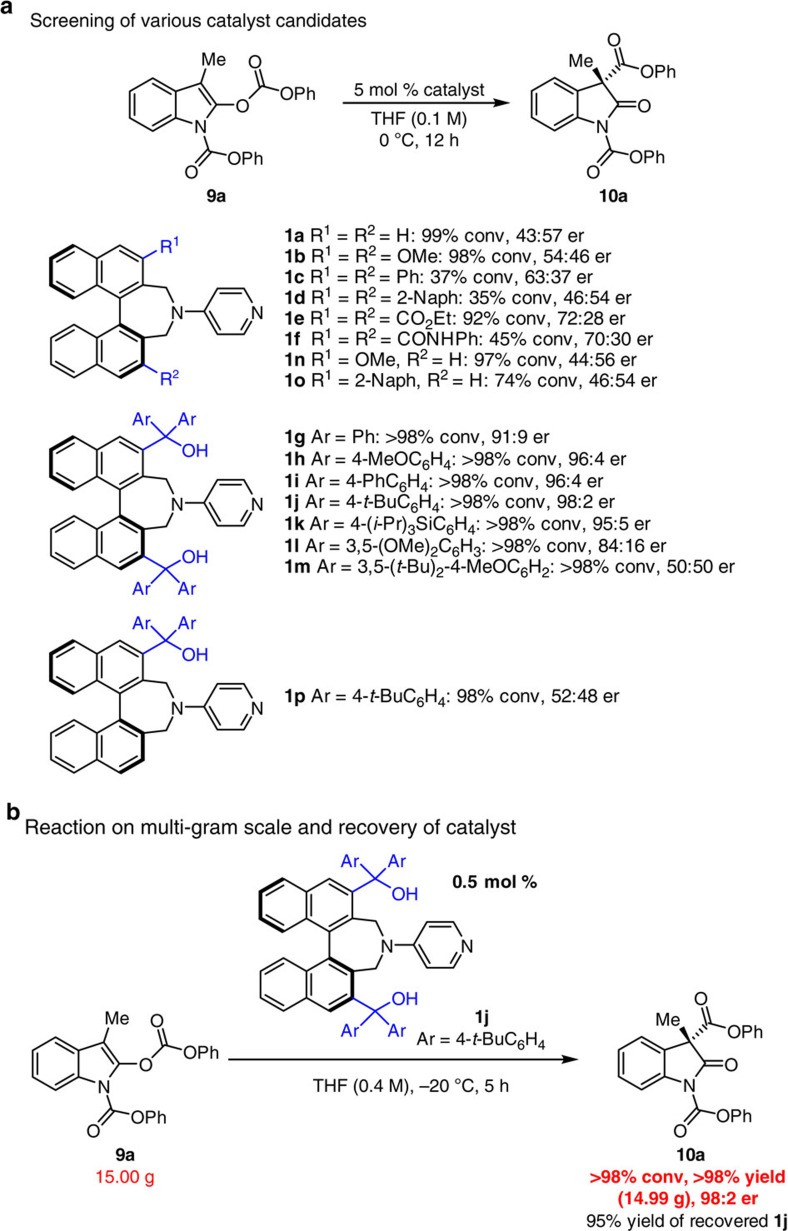
Enantioselective Steglich-type reactions promoted by catalyst 1j. (**a**) Initial catalysts screening for the Steglich rearrangement of *O*-acylated oxindole derivative with *C*_2_- or *C*_1_-symmetric binaphthyl-based chiral nucleophilic catalyst. The results clearly indicated that tertiary alcohol unit at C3 and C3′ of binaphthyl are essential for achieving high yield and enantioselectivity. (**b**) The enantioselective reaction can be easily performed in multigram scale without any adverse effect on the efficiency or enantioselectivity of the process. Moreover, the same silica gel chromatography procedure delivers the recovered chiral catalyst in 95% yield.

With a collection of possible catalysts in hand, we chose Steglich rearrangement^1^,^15^,^19^,^34^,^42^,^43^ of oxindole derivative **9a** (Design option 1, Fig. 2) as the model transformation for identifying the optimal promoter molecule. Reactions were performed using 5 mol % of **1a**−**p** in tetrahydrofuran (THF) (0.1 M) at 0 °C for 12 h (Fig. 4a). Reaction with 4-amino pyridine **1a** was efficient but generated nearly racemic product (99% conv., 43:57 enantiomeric ratio (er)). Structurally modified versions **1b−f**, which contain methoxy, phenyl, 2-naphthyl, carboxyl ester or amide groups at their C3 and C3′ positions were either equally or less efficient than **1a**, and were all ineffective in generating **10a** with significant enantioselectivity (no more than 72:28 er). The surprising breakthrough came when we evaluated compounds that contain tertiary alcohols unit within their chiral binaphthyl moiety (**1g−m**); under otherwise identical conditions, use of these catalysts led to the formation of the all-carbon quaternary stereogenic centre not only with exceptional efficiency (>98% conv.) but also in up to 98:2 er. In sharp contrast, *C*_1_-symmetric systems, represented by **1n**, **1o** and **1p** proved to be substantially less effective, and afforded nearly racemic mixture of product. Apparently, *C*_2_-symmetric scaffold are essential for the establishment of proper enantiofacial discrimination. Subsequent optimization studies (Supplementary Figs 29–38 and Supplementary Tables 1–5), revealed that THF is indeed the optimal solvent and when the transformation is performed at –20 °C with no more than 0.5 mol % **1j**, the reaction proceeds to complete conversion within five hours to afford **10a** in 98:2 er. Finally, we find that use of 15 g of substrate **9a** under the latter conditions (193 mg of **1j**) delivers the desired product in quantitative yield (purification by standard silica gel chromatography) and 98:2 er (Fig. 4b, Supplementary Figs 39 and 40); furthermore, catalyst **1j** could be recovered in 95% yield, and used in same reaction (0.1 mmol scale) to afford the desired product **10a** in >98% yield with 98:2 er without loss of any catalytic activity (Supplementary Fig. 41 and Supplementary Method).

**Figure 5:**
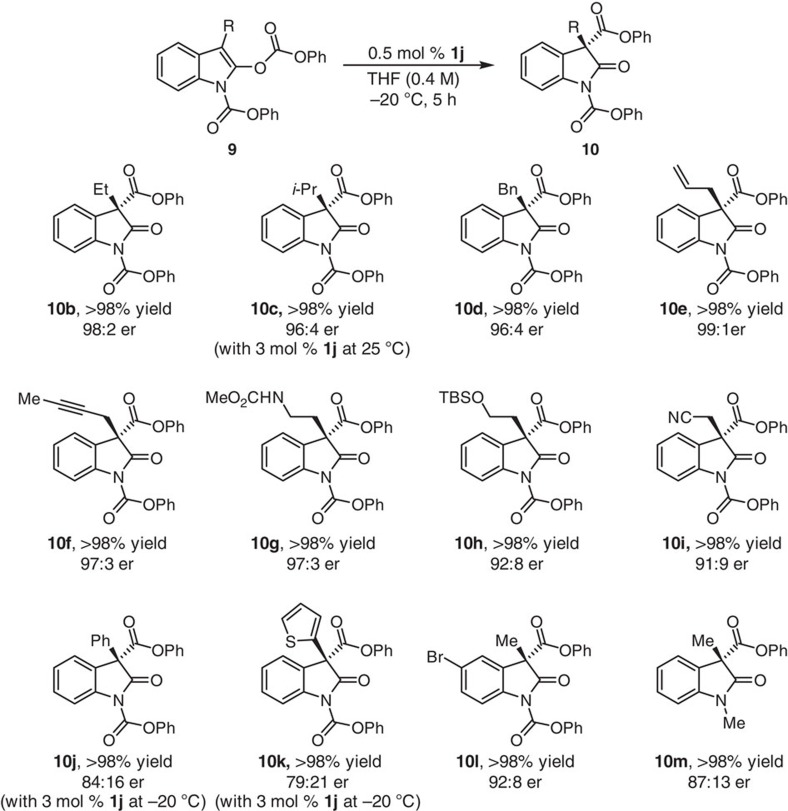
Enantioselective Steglich-type reactions of various substrates promoted by catalyst 1j. A wide range of substrates may be subjected to enantioselective acyl rearrangement processes that afford quaternary carbon stereogenic centres in quantitative yield and in up to 98:2 er. The level of enantioselectivity can depend on the nature of the substituents within the oxindole ring. Reactions were performed on a 0.1 or 0.2 mmol (**10b**, **10d** and **10l**) scale in THF (0.4 M) under an argon atmosphere. Yields are of isolated and purified products after silica gel chromatography (±2%). Er values (±1%) were determined by high-performance liquid chromatography (HPLC) analysis.

A variety of different substrates can be used in the enantioselective Steglich rearrangement process (Fig. 5 and Supplementary Figs 42–66). Thus, 3-alkyl-, 3-allyl- or 3-propargyl-substituted oxindole products **10b**−**f** were isolated in quantitative yields and in 96:4–99:1 er. Higher catalyst loading (3.0 versus 0.5 mol%) and temperature (25 versus –20 °C) was required in the case of **10c**, probably as a result of the presence of the more sterically demanding *iso*-propyl group. The stereochemical identity of **10c** was established through an X-ray structure (Supplementary Fig. 67). Transformation with substrates that contain an alkyl chain that bears a functionalizable polar group, such as an amide (**10g**), a silyl ether (**10h**) or a cyano unit (**10i**) proved to be exceptionally efficient (>98% yield) and highly enantioselective (91:9–97:3 er). Rearrangements involving 3-phenyl- (**10j**) or 3-thienyl-substituted oxindoles (**10k**) were more sluggish (3.0 mol % **1j** at −20 °C was needed) likely due to their sizeable nature, but were also less enantioselective than the previous examples (79:21–84:16 er). Efficient and enantioselective preparation of bromo-substituted **10l** (>98% yield, 92:8 er) and *N*-Me oxindole **10m** (>98% yield, 87:13 er) further illustrates the scope of the catalytic process.

### Mechanistic studies

**Figure 6:**
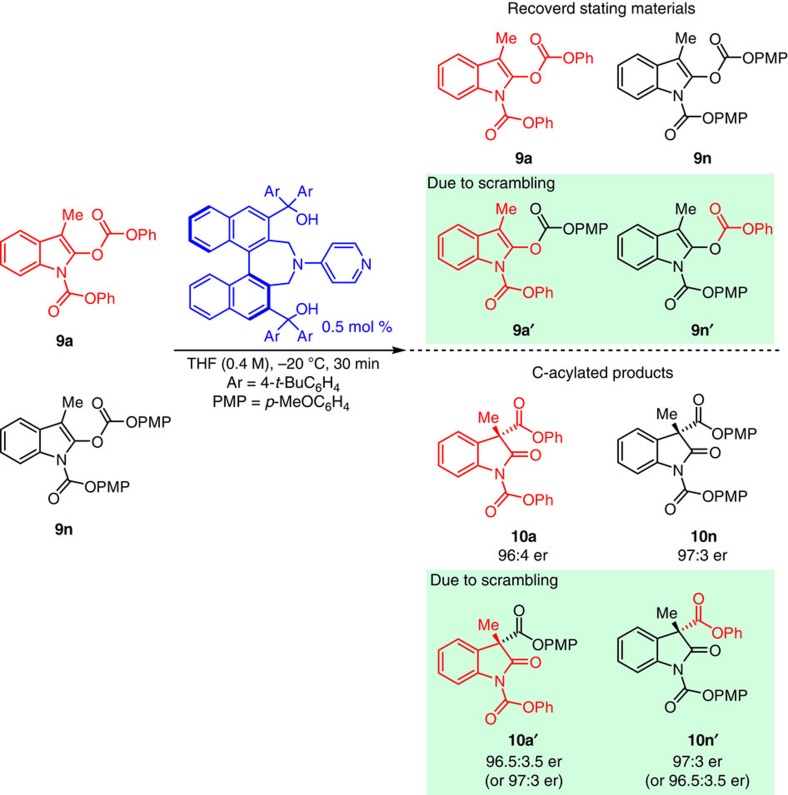
Cross-over studies of Steglich rearrangement of oxindole derivatives 9a and 9n. Cross-over studies between two marked substrates **9a** and **9n** with optimal catalyst **1j** under optimal conditions for 30 min. Four starting materials **9a**, **9n**, **9a′** and **9n′**, and four products **10a**, **10n**, **10a′** and **10n′** were observed, indicating that formation of an ion-pair is involved in the reaction proceed.

**Figure 7:**
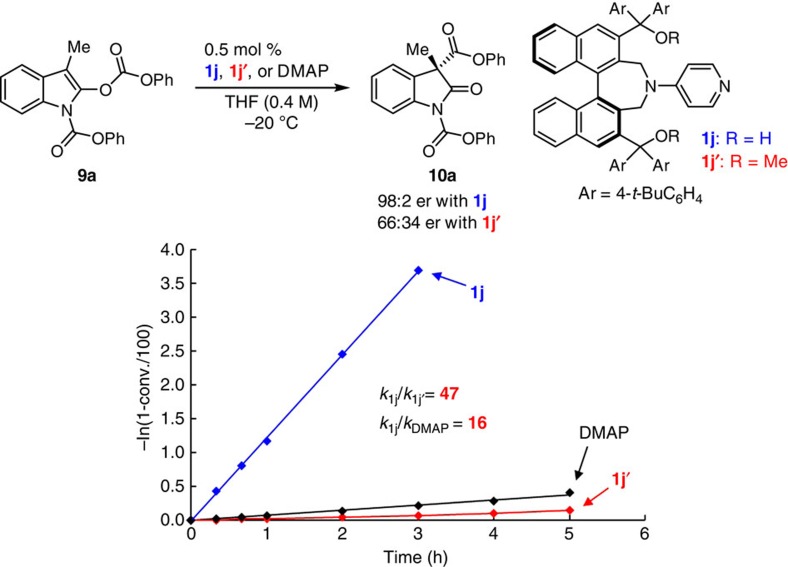
Kinetic studies in enantioselective Steglich rearrangement with catalyst 1j, 1j′ and DMAP. Kinetic profiles with catalyst **1j**, **1j′** and DMAP in Steglich rearrangement of **9a** under the optimal conditions. When **1j** was used as catalyst, higher catalytic activity (*k*_**1j**_=1.22 h^−1^) and enantioselectivity (98:2 er) were observed compared with bis-methyl ether catalyst **1j′** (*k*_**1j′**_=2.62 × 10^−2^ h^−1^ and 66:34 er).

To investigate the mechanism of the catalytic enantioselective rearrangement process, we performed a number of key experiments. First, cross-over studies (Fig. 6) between two marked substrates **9a** and **9n** clearly indicated that reactions proceed via an ion-pair complex, identifying the recovered starting material **9a** and **9n** (starting material); **9a′** and **9n′** (scrambled starting material) along with corresponding products **10a**, **10n**, **10a′** and **10n′** (Supplementary Fig. 68)^44^. Second, we carried out kinetic measurements (Fig. 7) with transformations promoted by catalyst **1j**, the corresponding bis-methyl ether **1j′**, and DMAP; oxindole **9a** served as the substrate under the optimal conditions (0.5 mol% catalyst, THF (0.4 M), −20 °C, 5 h). In all cases, the reactions were found to be first-order with respect to the substrate (Supplementary Table 6). Moreover, substantially higher activity was observed with catalyst **1j** (*k*_**1j**_=1.22 h^−1^) versus its derived bis-methyl ether **1j′** (*k*_**1j′**_=2.62 × 10^−2^ h^−1^), or DMAP (*k*_**DMAP**_=7.47 × 10^−2^ h^−1^). Thus, acyl rearrangement was nearly 50 times faster with **1j** compared with **1j′** and 16 times faster than DMAP. Additionally, whereas the desired product was obtained in 98:2 er when **1j** was use, there was hardly any enantiofacial selectivity when the hydroxyl units were protected (66:34 er with **1j′**). The presence of tertiary alcohols clearly has a significant impact on the rate as well as enantioselectivity of the catalytic process.

**Figure 8:**
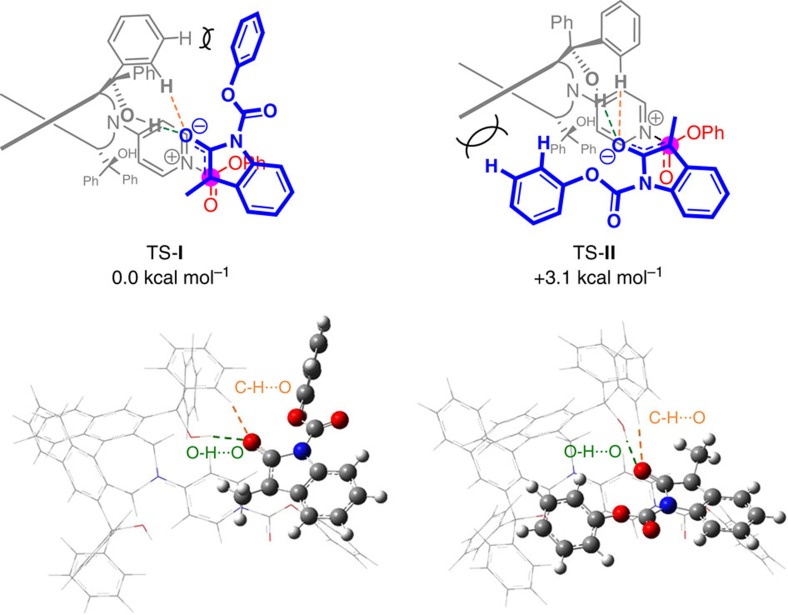
DFT calculations of two transition states TS-I and II in enantioselective Steglich rearrangement of 9a with 1g. Geometries of transition states TS-**I** and **II** from **9a** with **1g** were fully optimized by calculation using the B3LYP (Becke's three-parameter hybrid method using the Lee-Yang-Parr correlation functional) DFT with the 6-31G(d) basis set. Harmonic vibrational frequencies were computed for all stationary points to characterize them as saddle points.

To gain further insight regarding the role of the chiral catalyst's tertiary hydroxyl groups, density functional theory (DFT) calculations were performed at the B3LYP/6-31G(d) level, with **9a** as the substrate and compound **1g** as the catalyst, which represent simplified catalyst of **1j**. These investigations point to two critical attractive interactions, which directly involve the tertiary alcohol moiety, between the chiral catalyst and tight ion-pair intermediate. In the lowest energy transition state TS-**I** (Fig. 8 and Supplementary Table 7), which consistent with the experimental observations leads to the observed major enantiomer, there appears to be an H-bonding between the enolate oxygen of the ion pair and the catalyst's hydroxy unit; it is likely that the conformational rigidity imposed by the presence of the two aryl unit on the same carbon, forces the hydroxyl unit to be properly positioned to associate with the negatively charged oxygen. Additionally, there appears to be an additional electrostatic attraction^45^,^46^ between the enolate and an ortho hydrogen of one of the aforementioned aryl units at the C3 site of the chiral catalyst. The second lowest mode of addition, as indicated by DFT calculations, is transition state **II** (Fig. 8 and Supplementary Table 8). While there appears to be similar electrostatic attractive forces operative here as well, **II** seems to suffer from a significant steric repulsion between the substrates phenoxy unit and the backbone of the catalyst's biaryl moiety. Nevertheless, DFT calculations indicate that there is in all likelihood steric repulsion between phenyl unit and the tertiary hydroxyl fragment that is involved in attractive interactions with the enolate group (see transition state (TS)-**I**, Fig. 8); this is supported by the findings that a catalyst with meta-(3,5)-substituted aryl groups give lower enantioselectivities in the reaction of **9a** (cf. **1l** or **1m** in Fig. 4). Further, as indicated by the transformation that afforded **10m** (Fig. 5) involving a more diminutive *N*-Me group, while less enantioselective than when a benzoate group is present (**9a**), there is still appreciable enantiofacial differentiation observed (98:2 er versus 87:13 er for **10a** and **10m**, respectively). This suggests that other, non-steric, factors are at play here as well. Towards this end, second order perturbation theory analysis in NBO at the M062X/6-31g** level indicates that C–H/enolate attraction might be notably stronger in the favoured TS-**I** (Supplementary Table 8).

### The scope of the reaction

**Figure 9:**
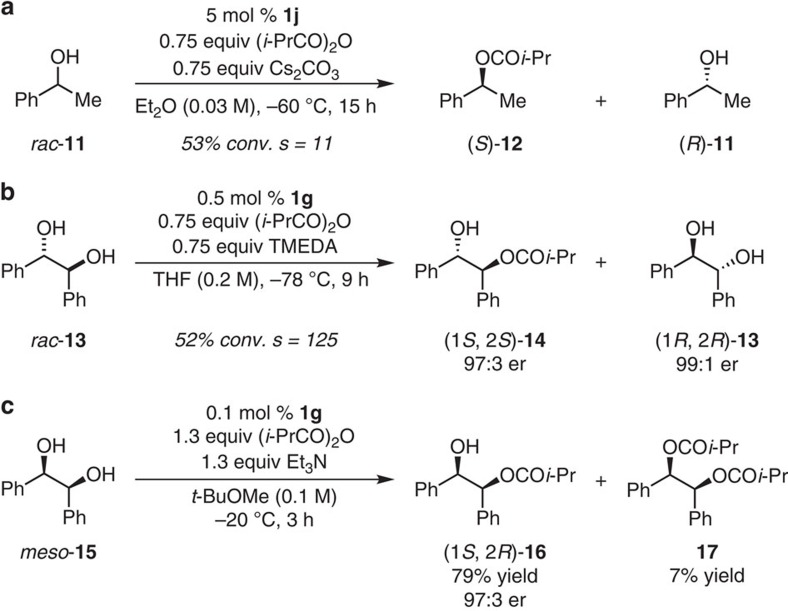
Application of the catalyst with *tert*-alcohols at 3,3′-positions of a 1,1′-binaphthyl unit to intermolecular acylations. (**a**) Kinetic resolution of secondary carbinol with 5 mol% **1j**. The reaction proceeded smoothly with moderate selectivity factor. Conversion values (±1%) and er values (±1%) were determined by high-performance liquid chromatography (HPLC) analysis. (**b**) Kinetic resolution of acyclic *d,l*-1,2-diol **13** with 0.5 mol % **1g**. The reaction proceeded smoothly to afford (1*S*, 2*S*)-**14** and (1*R*, 2*R*)-**13** with high enantioselectivity. None of the di-acylated product was observed. Conversion values (±2%) were determined by ^1^H nuclear magnetic resonance (NMR) analysis of the unpurified reaction mixtures. Er values (±1%) were determined by HPLC analysis. (**c**) Desymmetrization of acyclic meso-1,2-diol **15** with 0.1 mol% **1g**. Monoprotected diol (1*S*, 2*R*)-**16** was obtained in 79% yield with high enantioselectivity (97:3 er). Small amount of di-acylated product **17** and meso-**15** was obtained in 7 and 12% yield, respectively. Er values (±1%) were determined by HPLC analysis.

The utility of the present class of catalysts is not confined to Steglich rearrangements. For examples, as the representative data in Fig. 9 indicate, we find that binaphthyl-based DMAP derivatives with *tert*-alcohols at 3,3′-positions of a 1,1′-binaphthyl unit is not only effective in kinetic resolution of secondary carbinol and *d,l*-1,2-diol but in enantioselective desymmetrization of meso-1,2-diol as well. Initially, kinetic resolution of secondary carbinol rac-**11** with an array of binaphthyl-based DMAP derivatives was performed. The reaction in the presence of 5 mol % **1j** proceeded smoothly with moderate *s*-factor (*s*=11, Fig. 9a, and Supplementary Figs 69–72). We then turned our attention to 1,2-diol which might be more suitable than simple carbinols, expecting positive interactions between a substrate and a catalyst through the hydrogen bonding.

Its merits note that, although a number of organocatalysts for kinetic resolution and desymmetrization of 1,2-diol have been disclosed, the significant majority involve the use of cyclic substrates because enantioselectivity was usually high compared with acyclic substrate^8^. The successful organocatalytic approaches with high enantioselectivity for kinetic resolution^47^ and desymmetrization^48^,^49^ of acyclic 1,2-diols were only reported by Fujimoto utilizing cinchona alkaloids-based catalyst. However, higher catalyst loading (30 mol %) were required for these transformation. Other examples for desymmetrization of acyclic meso-1,2-diols resulted in moderate enantioselectivity of mono-acylated product along with significant amount of undesired bis-acylated product^14^,^50^,^51^,^52^. Thus, an efficient resolution (*s*=125) of *d,l*-1,2-diol **13** was observed in an acylation reaction promoted by 0.5 mol % **1g** performed in THF at −78 °C for 9 h (Fig. 9b and Supplementary Figs 73–76). None of the di-acylation product was observed. Furthermore, desymmetrization of acyclic meso-1,2-diol **15** required only 0.1 mol% **1g** and proceeded to furnish the desired mono-ester **16** in 79% yield with 97:3 er along with small amount of di-acylated product **17** and recovered meso-**15** in 7% and 12% yield, respectively (Fig. 9c and Supplementary Figs 77 and 78). The substantially faster rate of the first acylation reaction points to a high degree of catalyst-substrate molecular recognition that might be largely due to precise arrangements that are required for the type of H-bonding interactions depicted for ‘design option 2' in Fig. 2. These preliminary results regarding enantioselective acylation processes, along with the aforementioned Steglich rearrangement reactions, suggest that the present set of catalysts may be applicable to a broad range of acyl transfer-type process.

## Discussion

The studies described above illustrate that the combination of a binaphthyl moiety, a widely utilized chiral motif in enantioselective catalysis, a 4-aminopyridine, a most effective nucleophilic catalyst, can be effectively used in the development of exceptionally effective small organic molecules that can serve as chiral catalysts for acyl transfer processes. Another crucial design element relates to the presence of suitably positioned hydroxy groups that can facilitate reaction and promote enantioselectivity by establishing electrostatic association with an anionic Lewis basic fragment. The need for incorporation of additional substituents within the pyridyl ring, which can cause significant lowering of catalyst activity, may therefore be obviated. With the present blueprint in hand, development of highly efficient and selective catalysts that belong to the same class and may be used for related transformations such as intermolecular phosphorylation^53^, silylation^54^,^55^,^56^,^57^,^58^ or tosylation^59^ of polyhydroxylated organic molecules may be envisioned. Investigations along these lines are now underway.

## Method

### General procedure fur enantioselective Steglich rearrangement under optimal conditions

A solution of the catalyst in THF (50.0 mM) was prepared in advance using the following reaction.

To a solution of substrate **9** (0.2 mmol) in THF (0.5 ml, 0.4 M) was added a solution of the catalyst (20 μl, 50 mM in THF) at −20 °C. The reaction mixture was stirred for 5 h and then 1 M aqueous HCl was added. The resulting mixture was extracted with EtOAc, dried over MgSO_4_ and concentrated *in vacuo*. The purification of the crude product by flash column chromatography on a short pad of silica gel (eluent:hexane/Et_2_O=1/1, v/v) gave the corresponding product **10** (>98% yield). For nuclear magnetic resonance and high-performance liquid chromatography analyses of the products, see the Supplementary Figs 39–41 and 43–66.

## Additional information

**Accession codes:** The X-ray crystallographic coordinates for structures reported in this study have been deposited at the Cambridge Crystallographic Data Centre (CCDC), under deposition numbers CCDC 1432805–1432807. These data can be obtained free of charge from The Cambridge Crystallographic Data Centre via www.ccdc.cam.ac.uk/data_request/cif.

**How to cite this article:** Mandai, H. *et al*. Enantioselective acyl transfer catalysis by a combination of common catalytic motifs and electrostatic interactions. *Nat. Commun.* 7:11297 doi: 10.1038/ncomms11297 (2016).

## Supplementary Material

Supplementary Information Supplementary Figures 1-78, Supplementary Tables 1-8, Supplementary Methods and Supplementary References
