# Supplementary material for: Enantioselective acyl transfer catalysis by a combination of common catalytic motifs and electrostatic interactions
Source: Nat Commun. 2016 Apr 15;7:11297. doi: 10.1038/ncomms11297 (PMC4835565; doi:10.1038/ncomms11297)
Supplement: Supplementary Information — Supplementary Figures 1-78, Supplementary Tables 1-8, Supplementary Methods and Supplementary References [file ncomms11297-s1.pdf]

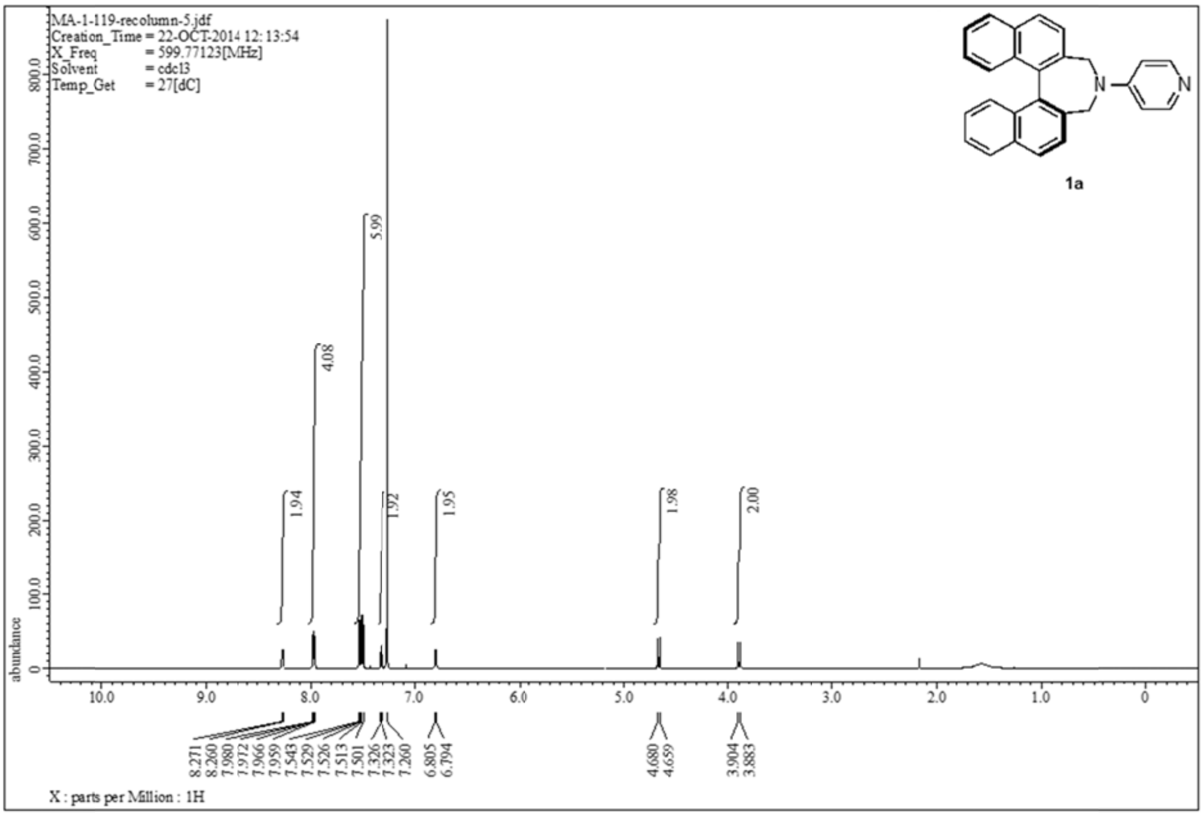

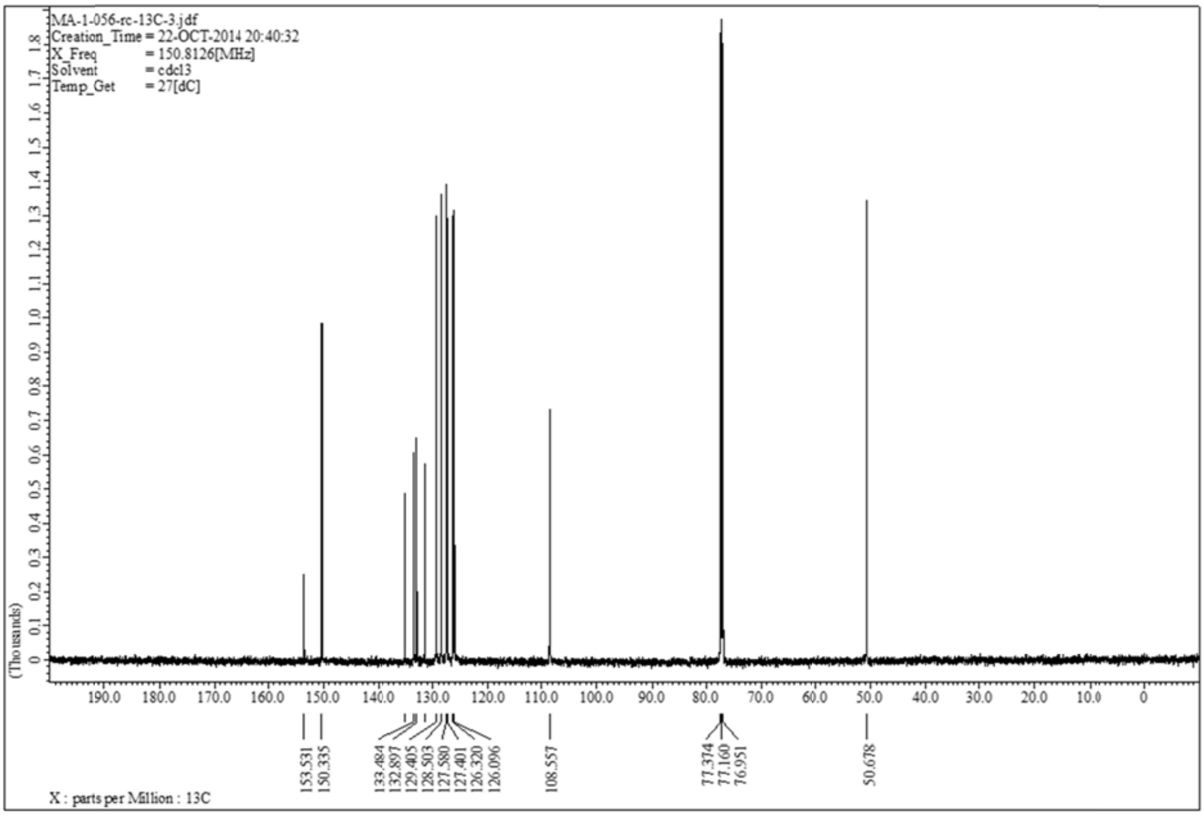

Supplementary Figure 1. ^{1}H and ^{13}C-NMR spectra for catalyst **1a**

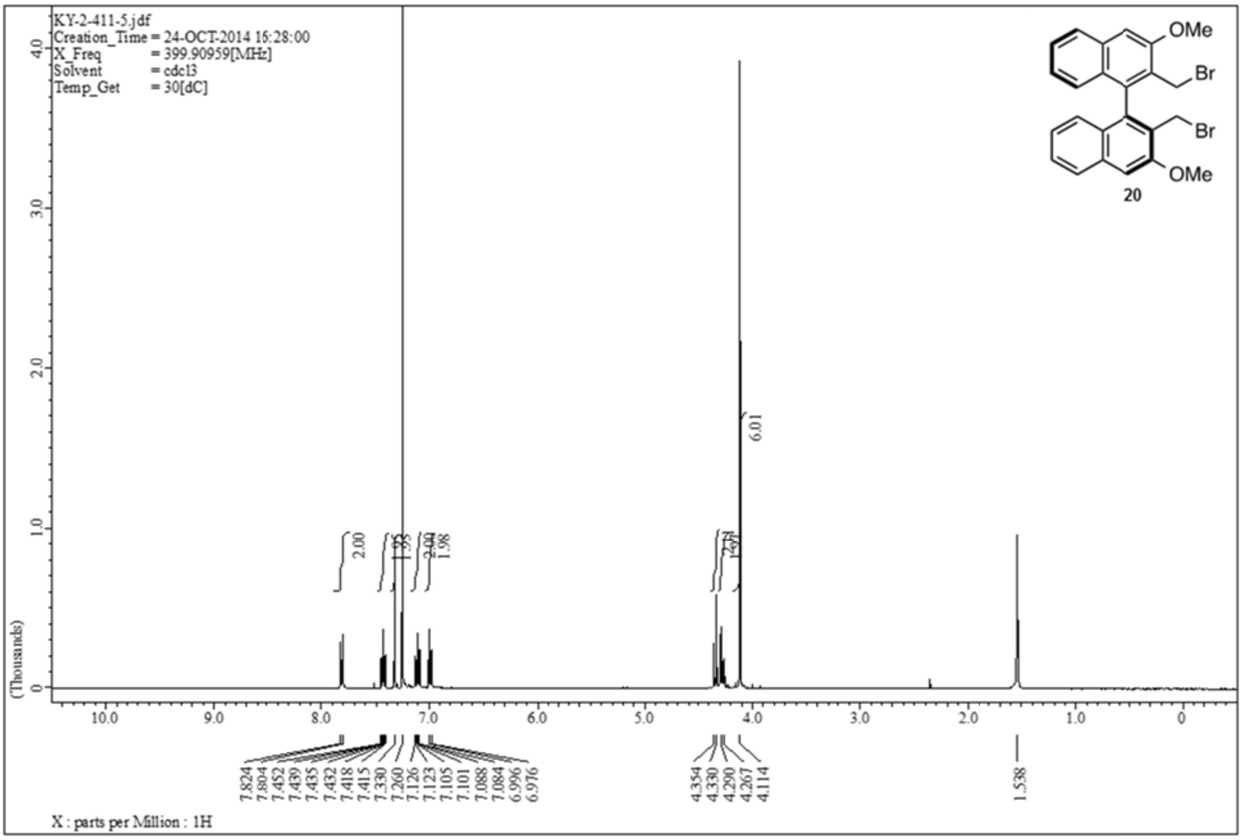

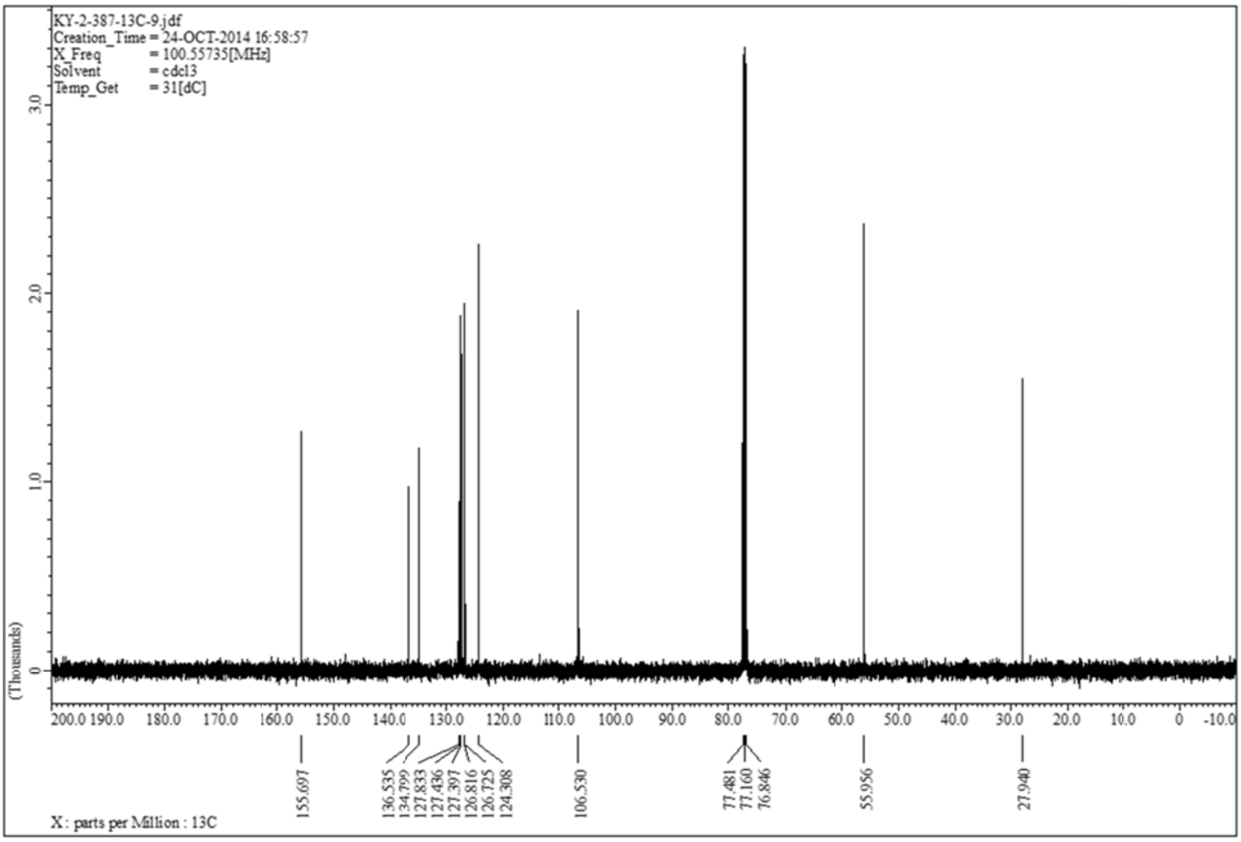

Supplementary Figure 2. ^{1}H and ^{13}C-NMR spectra for **20**

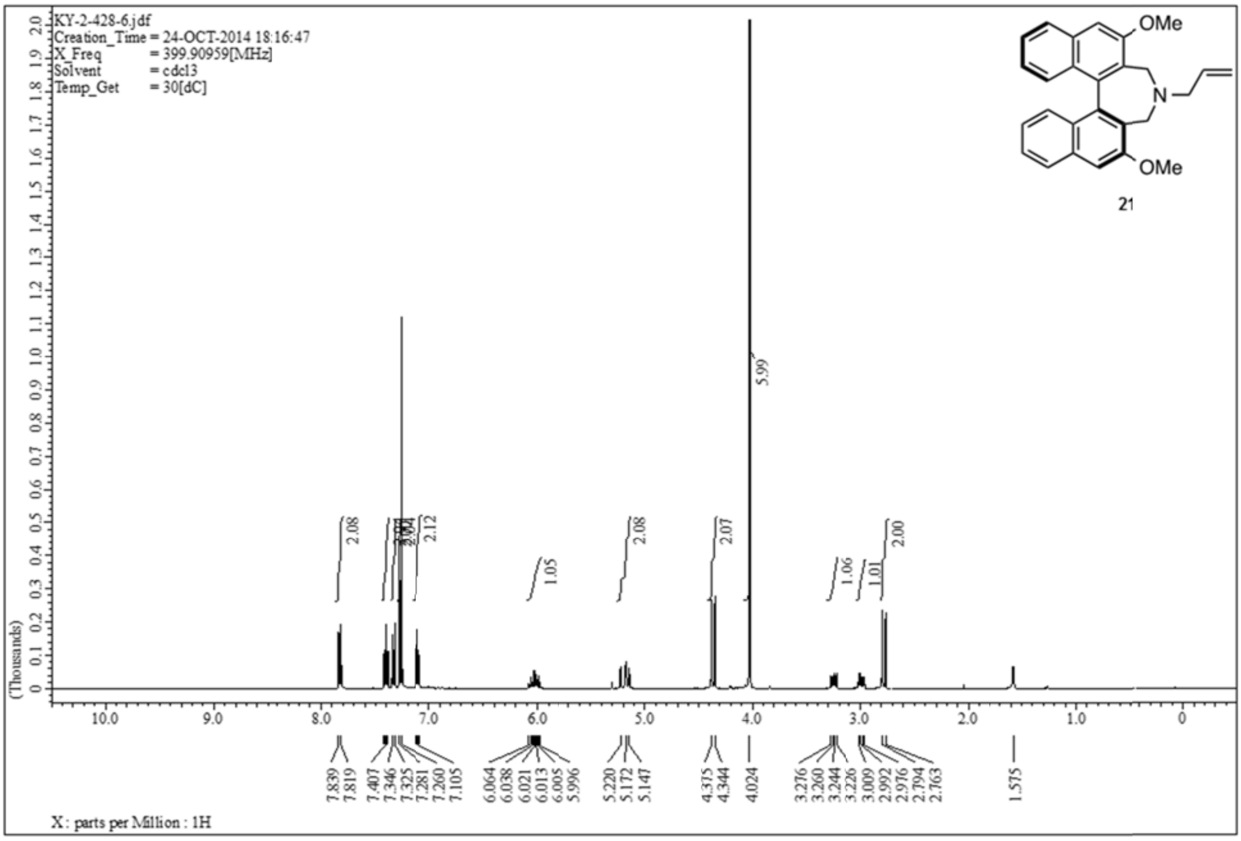

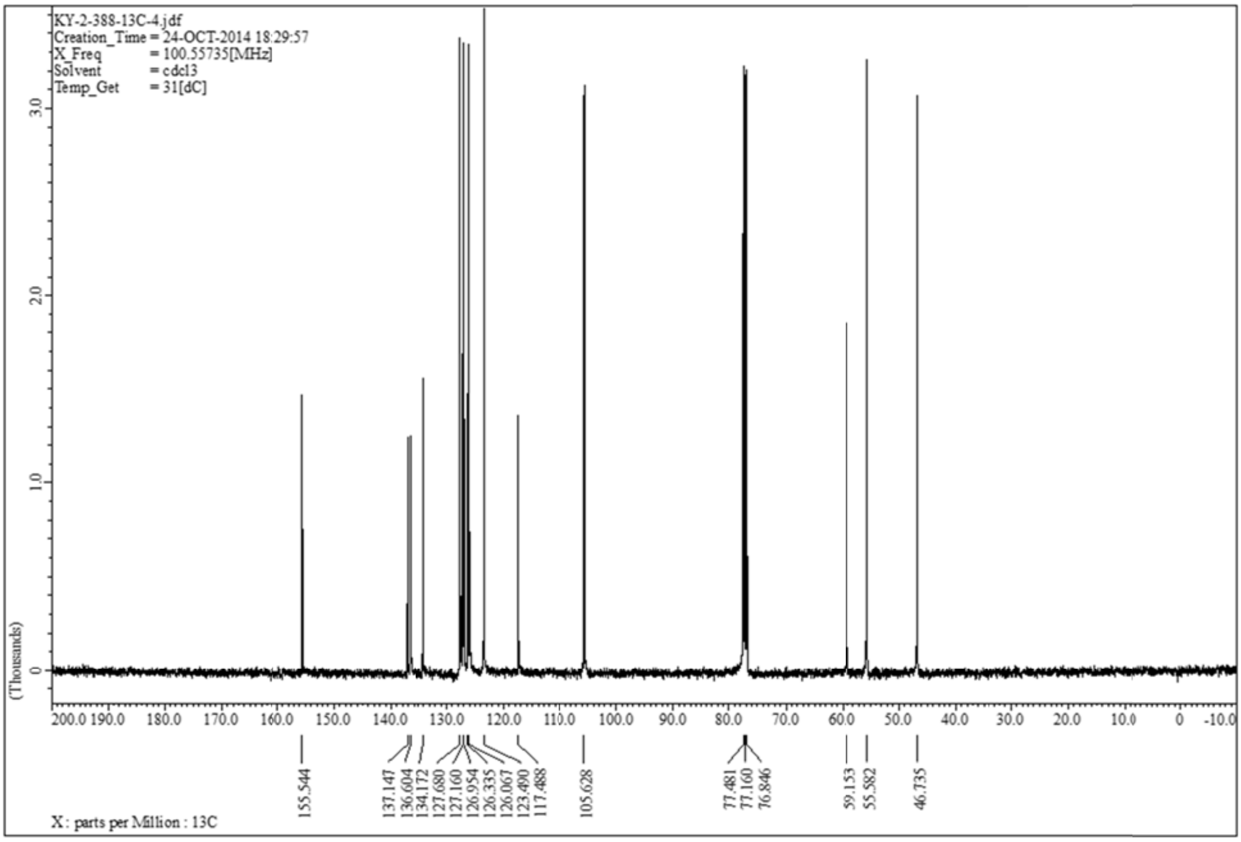

Supplementary Figure 3. ^{1}H and ^{13}C-NMR spectra for **21**

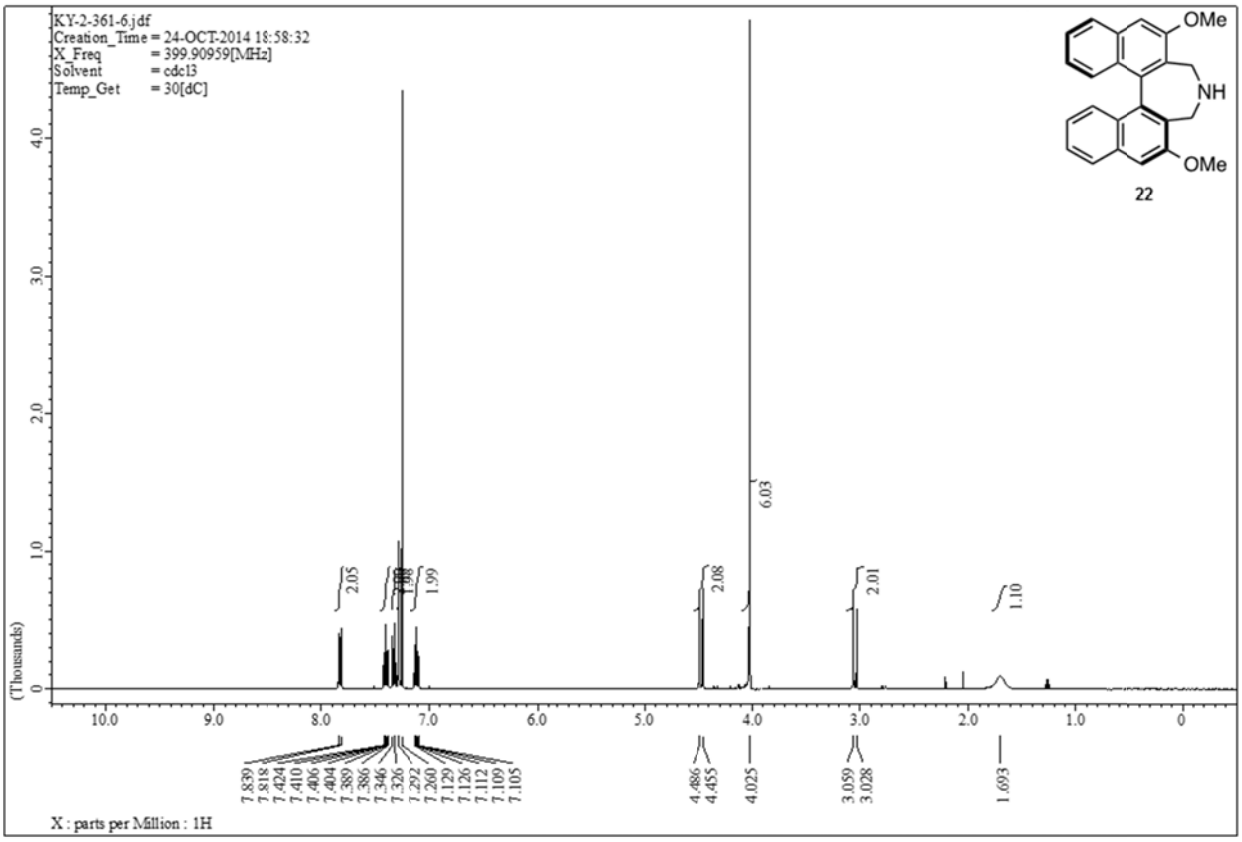

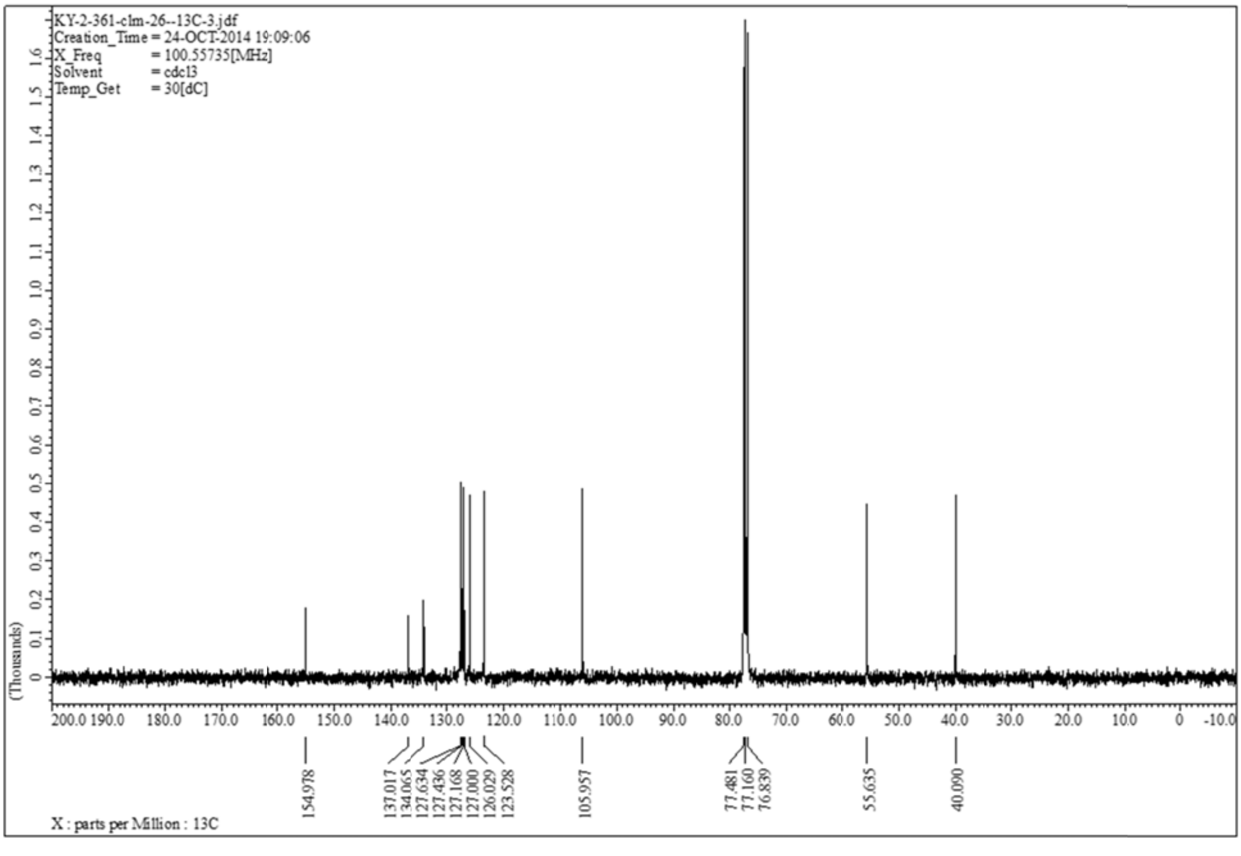

Supplementary Figure 4. ^{1}H and ^{13}C-NMR spectra for **22**

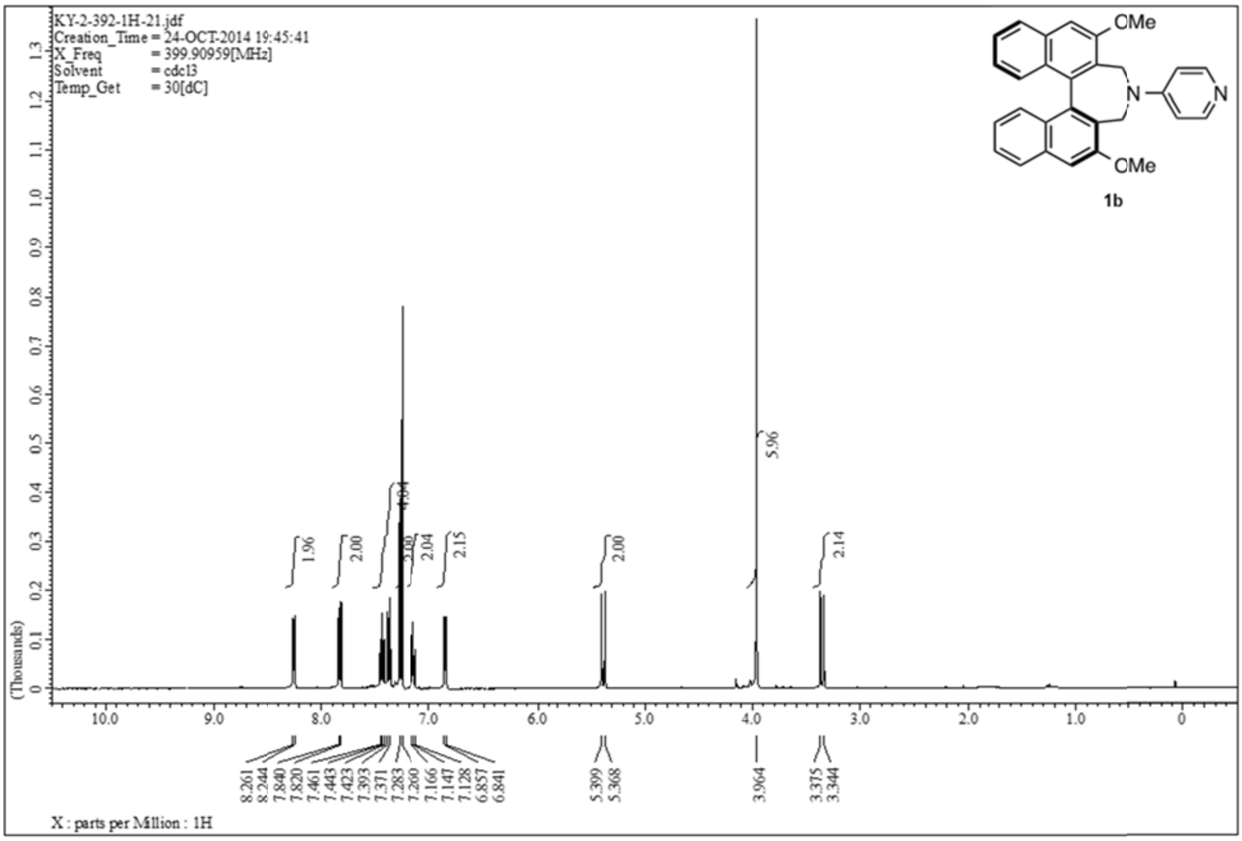

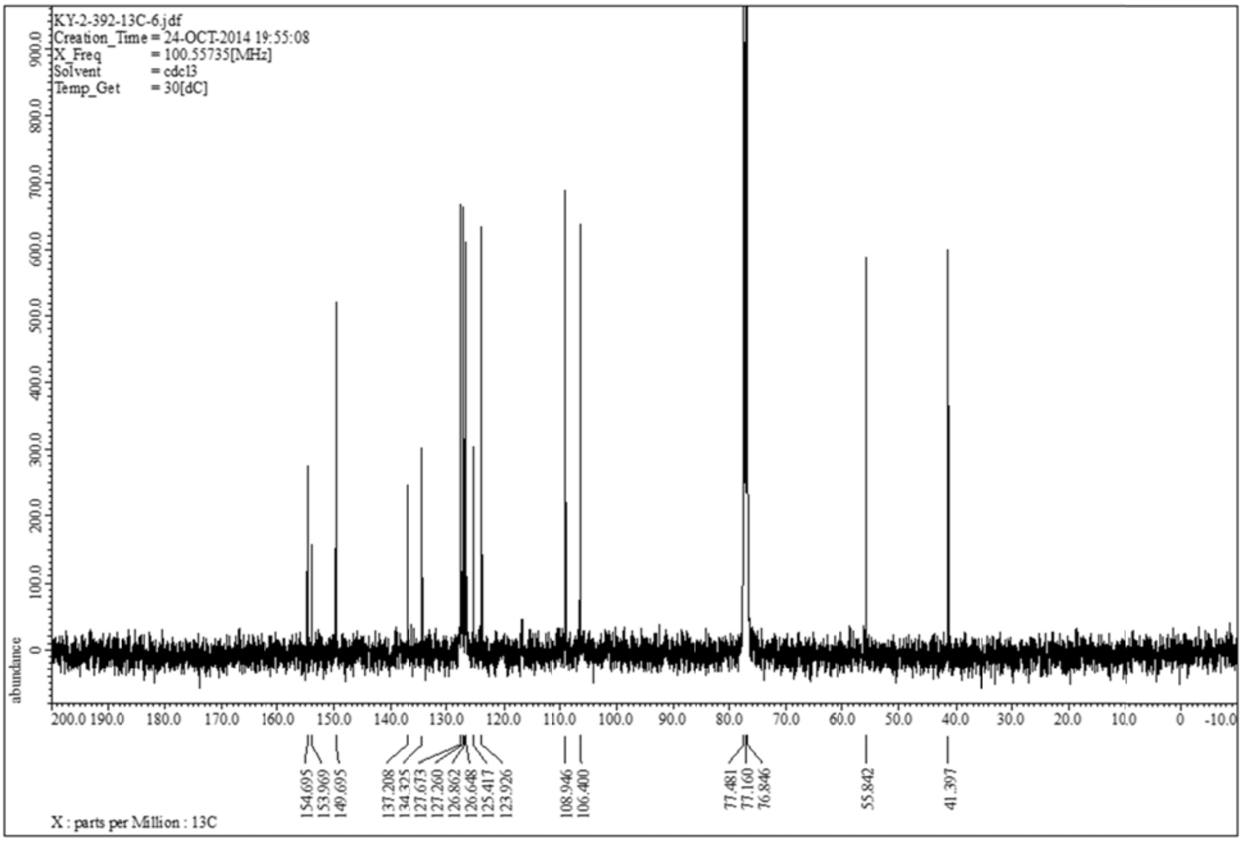

Supplementary Figure 5. ^{1}H and ^{13}C-NMR spectra for catalyst **1b**

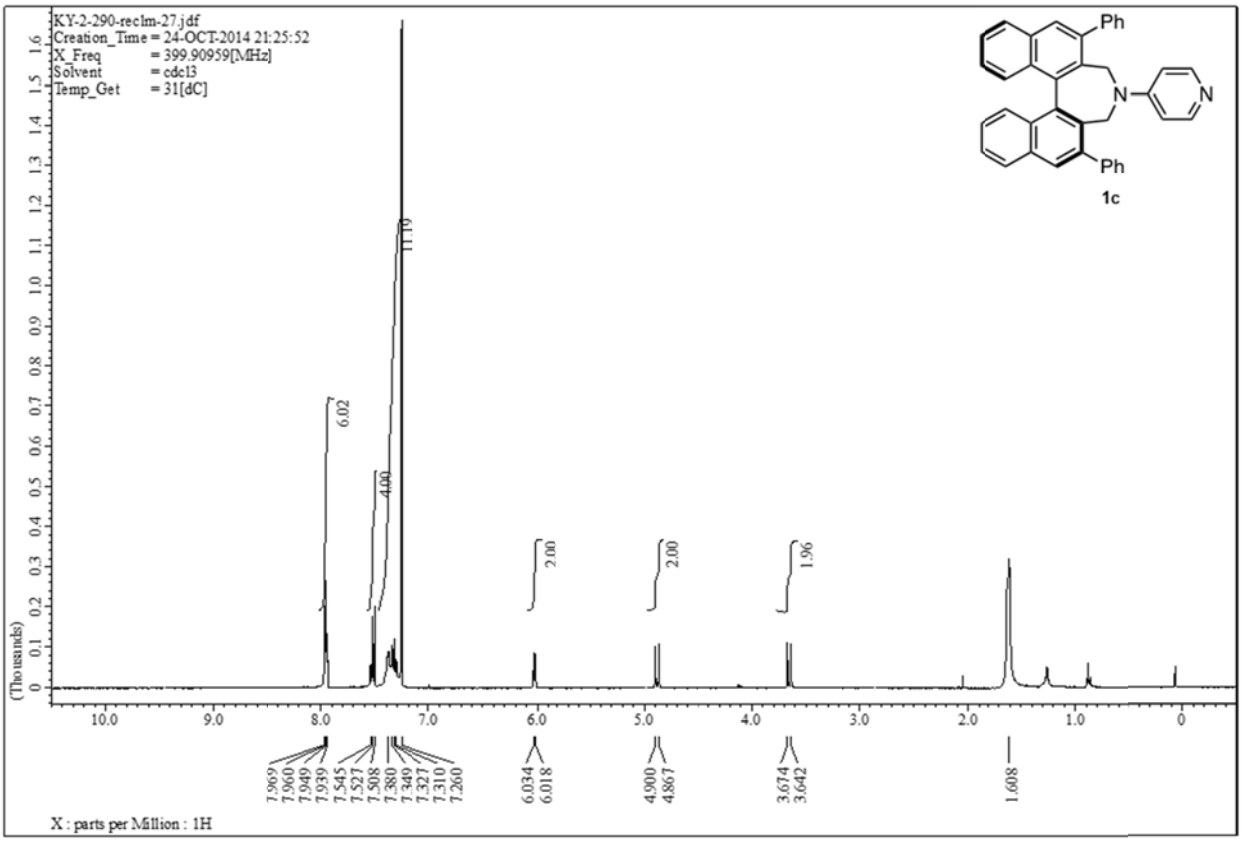

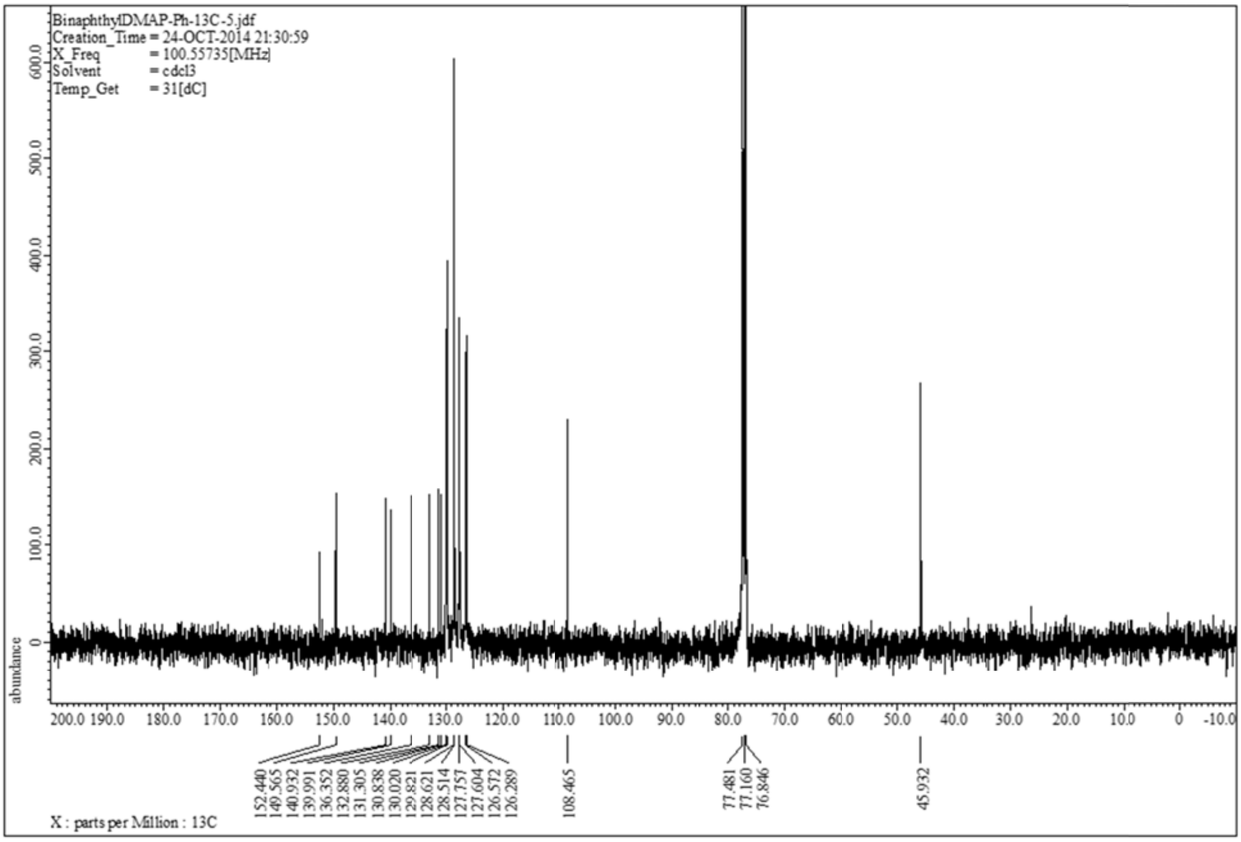

Supplementary Figure 6. ^{1}H and ^{13}C-NMR spectra for catalyst **1c**

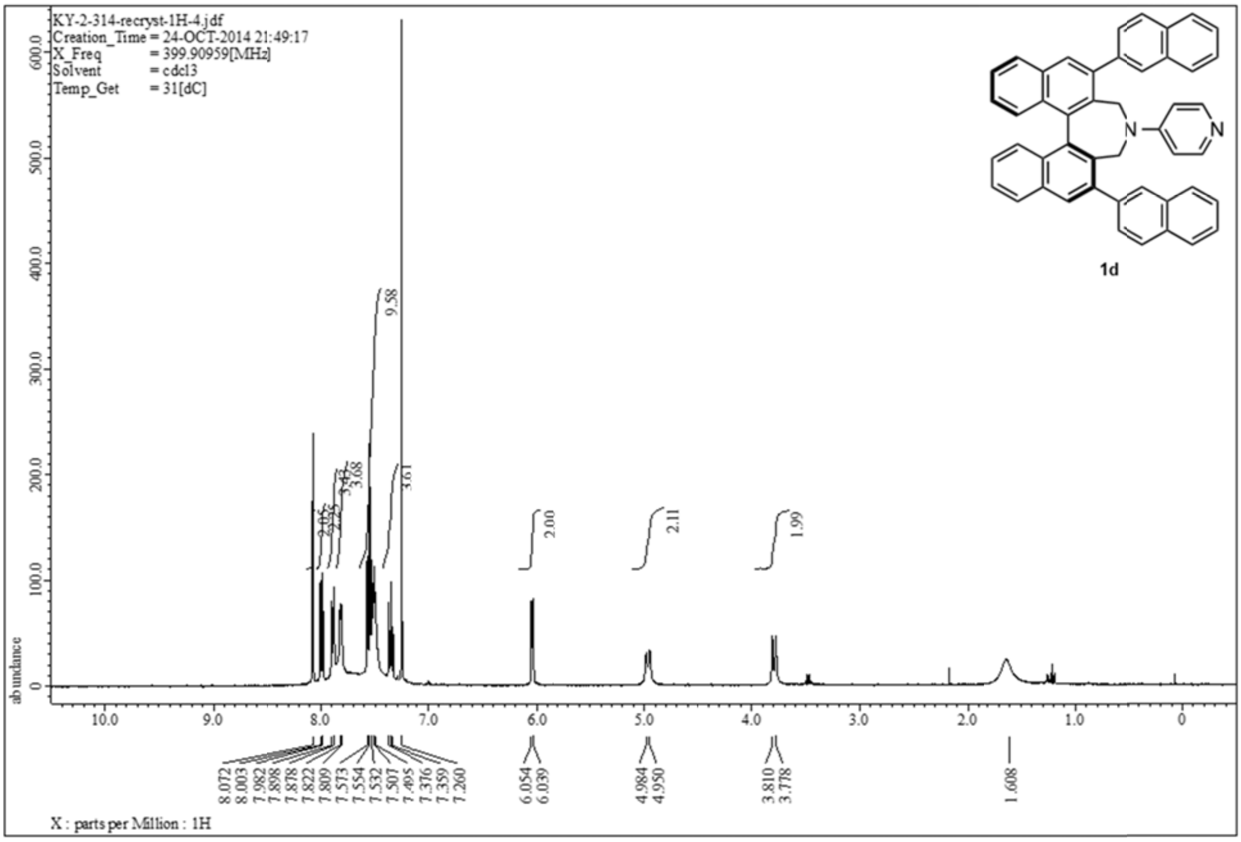

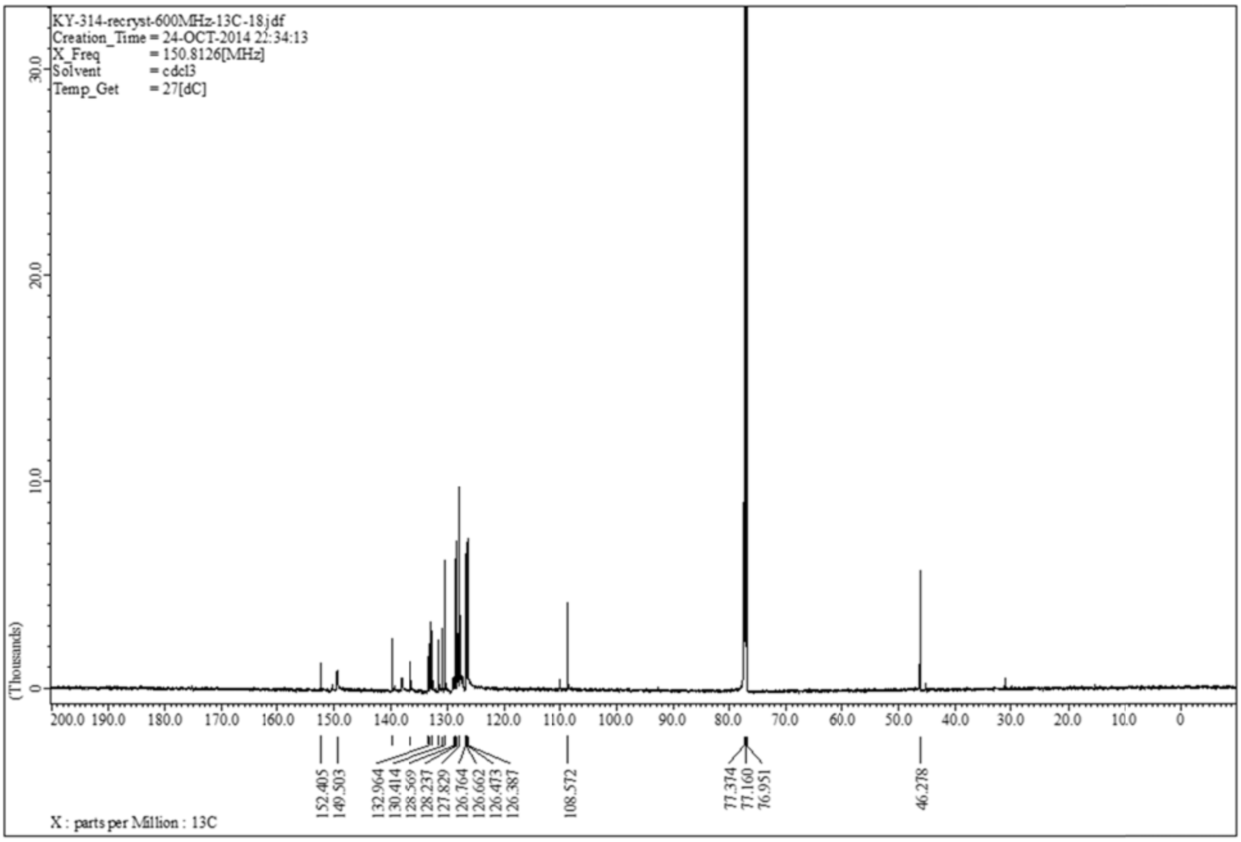

Supplementary Figure 7. ^{1}H and ^{13}C-NMR spectra for catalyst **1d**

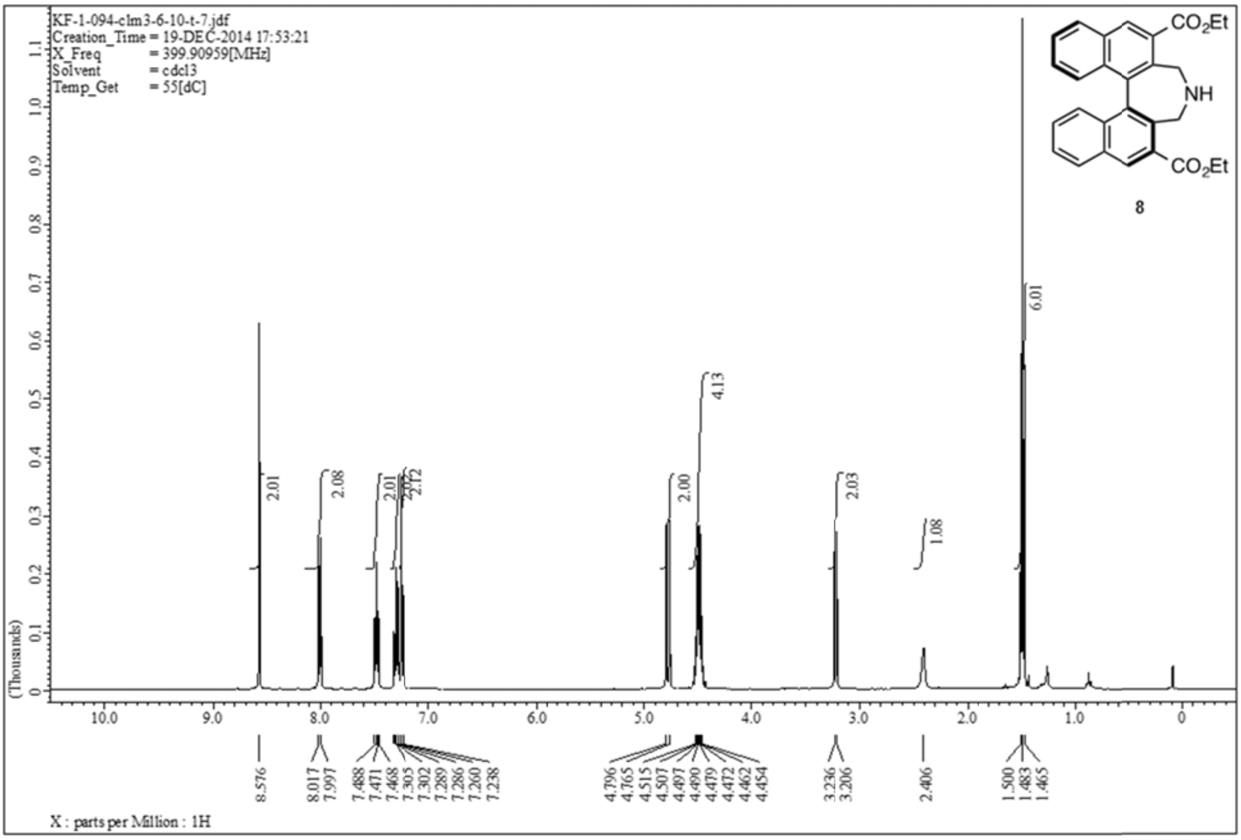

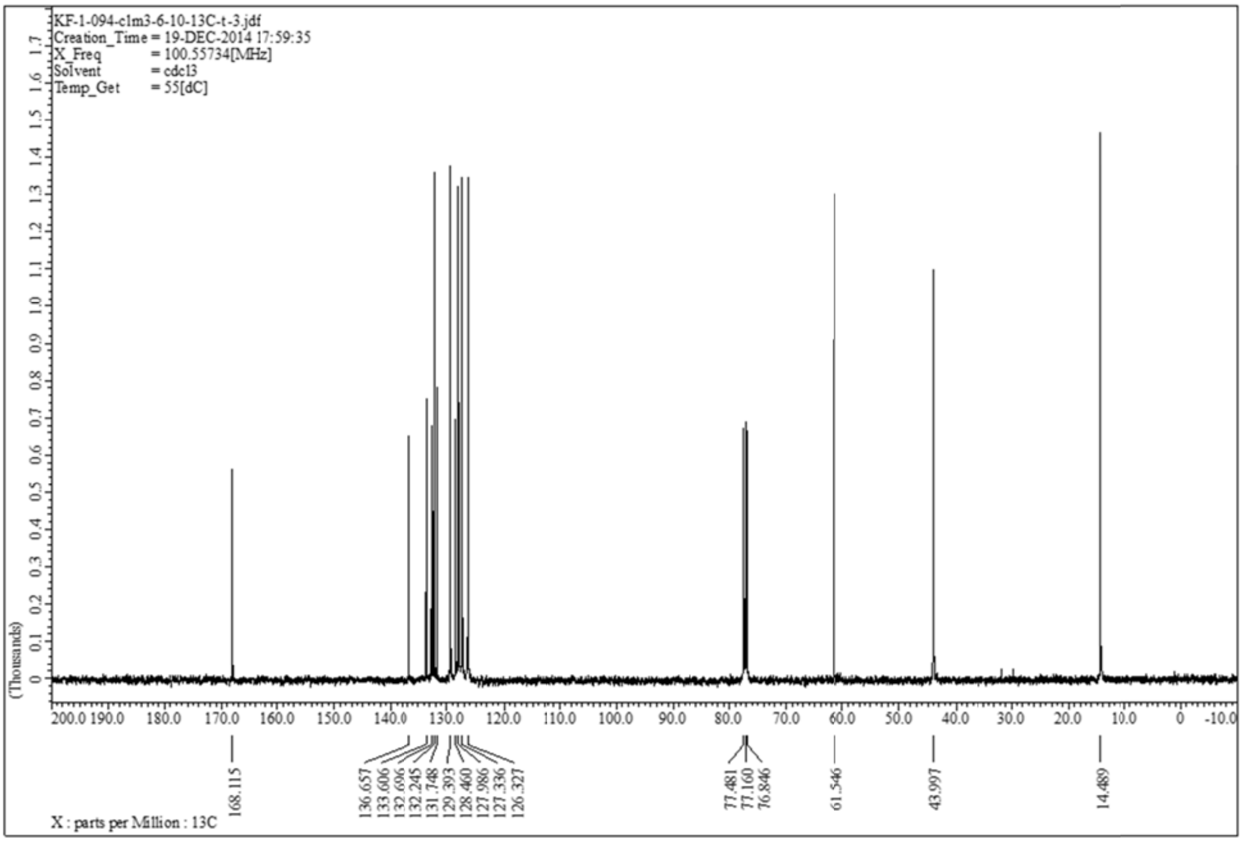

Supplementary Figure 8. ^{1}H and ^{13}C-NMR spectra for **8**

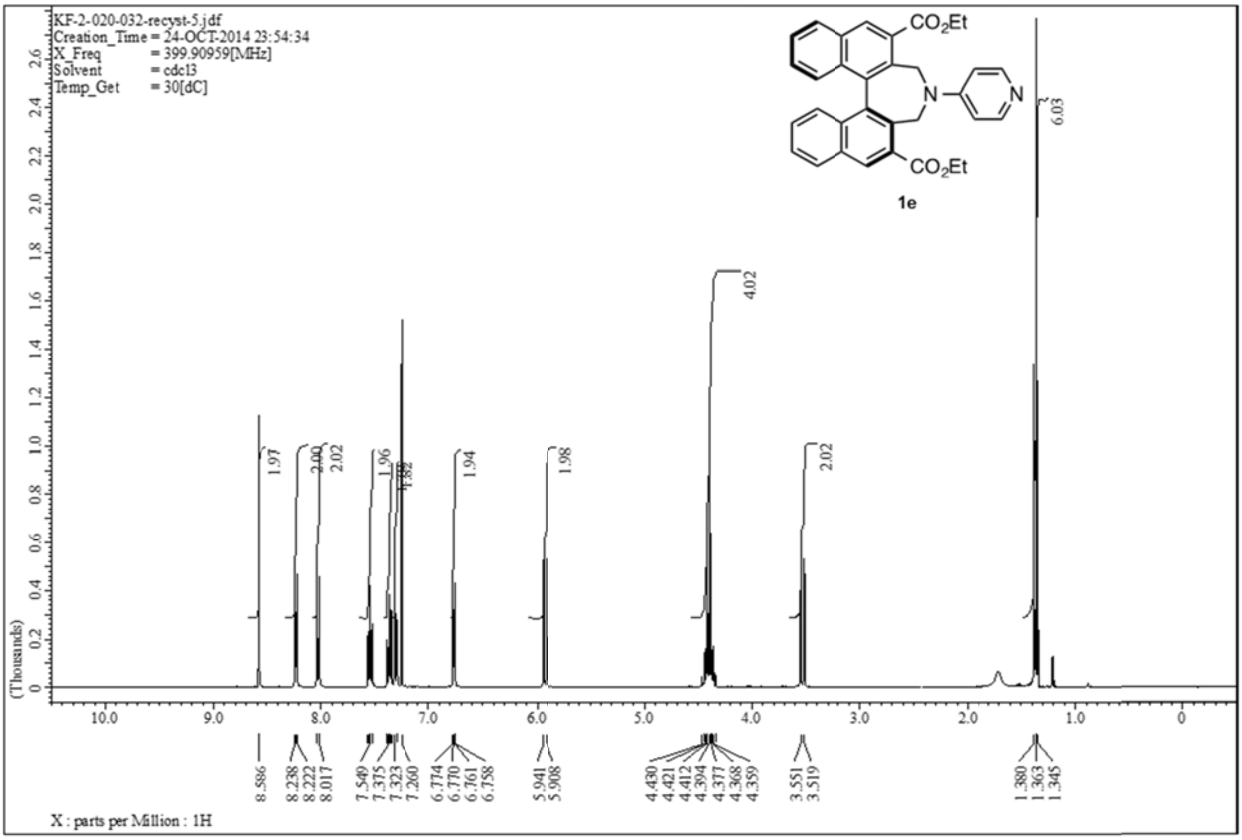

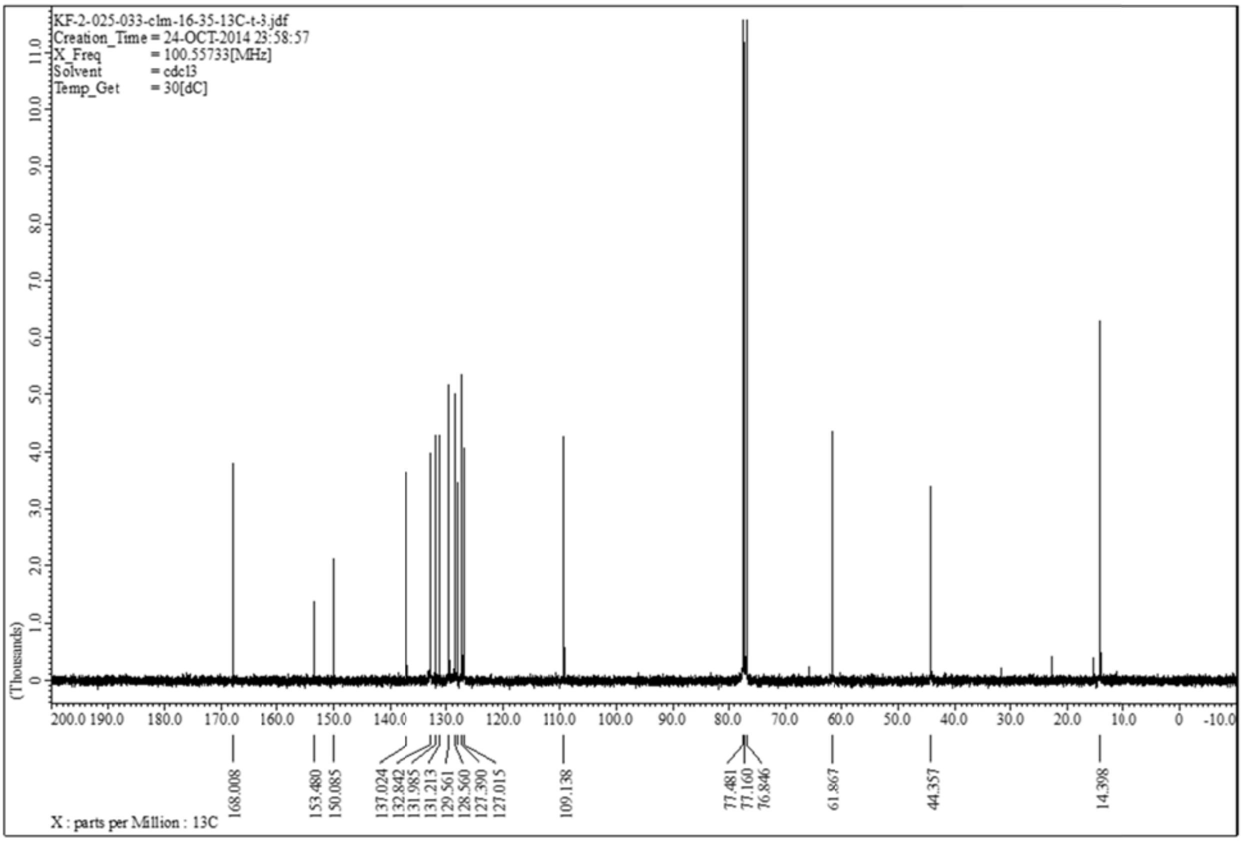

Supplementary Figure 9. ^{1}H and ^{13}C-NMR spectra for catalyst **1e**

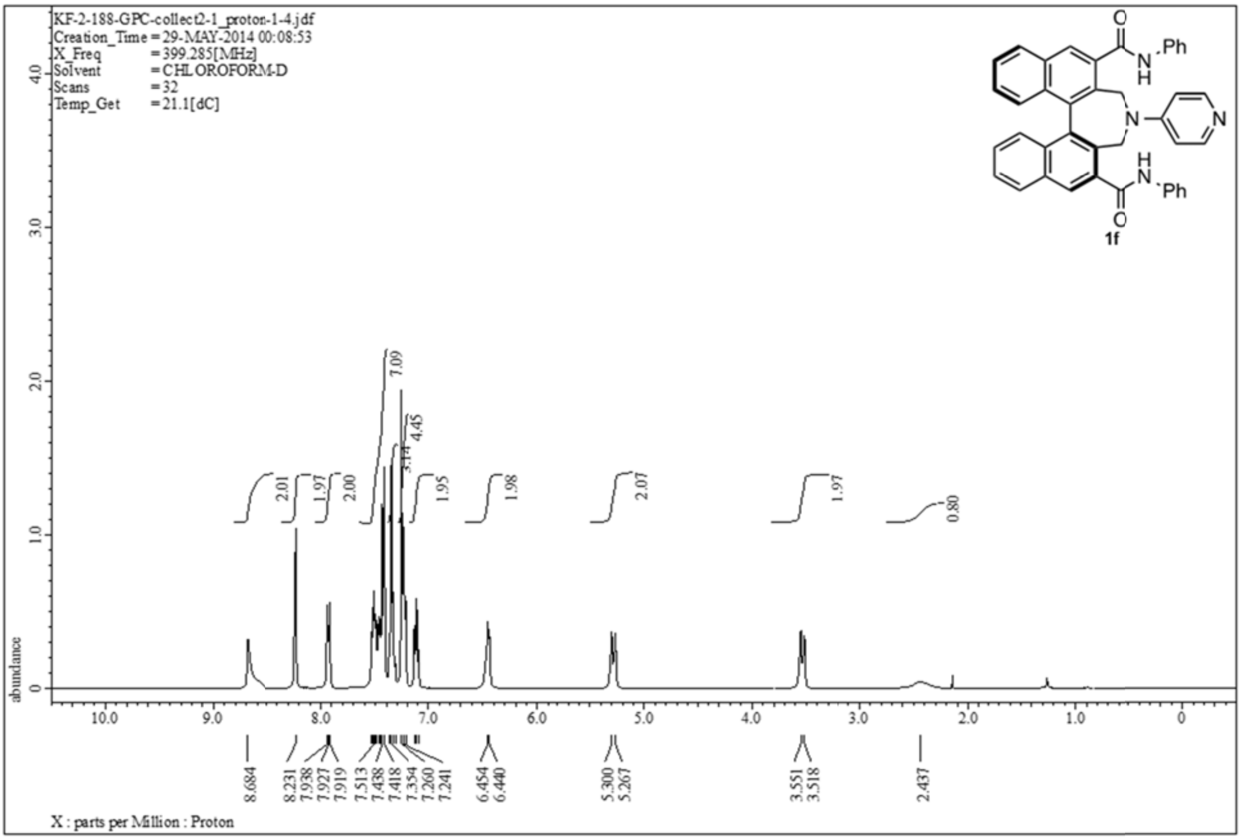

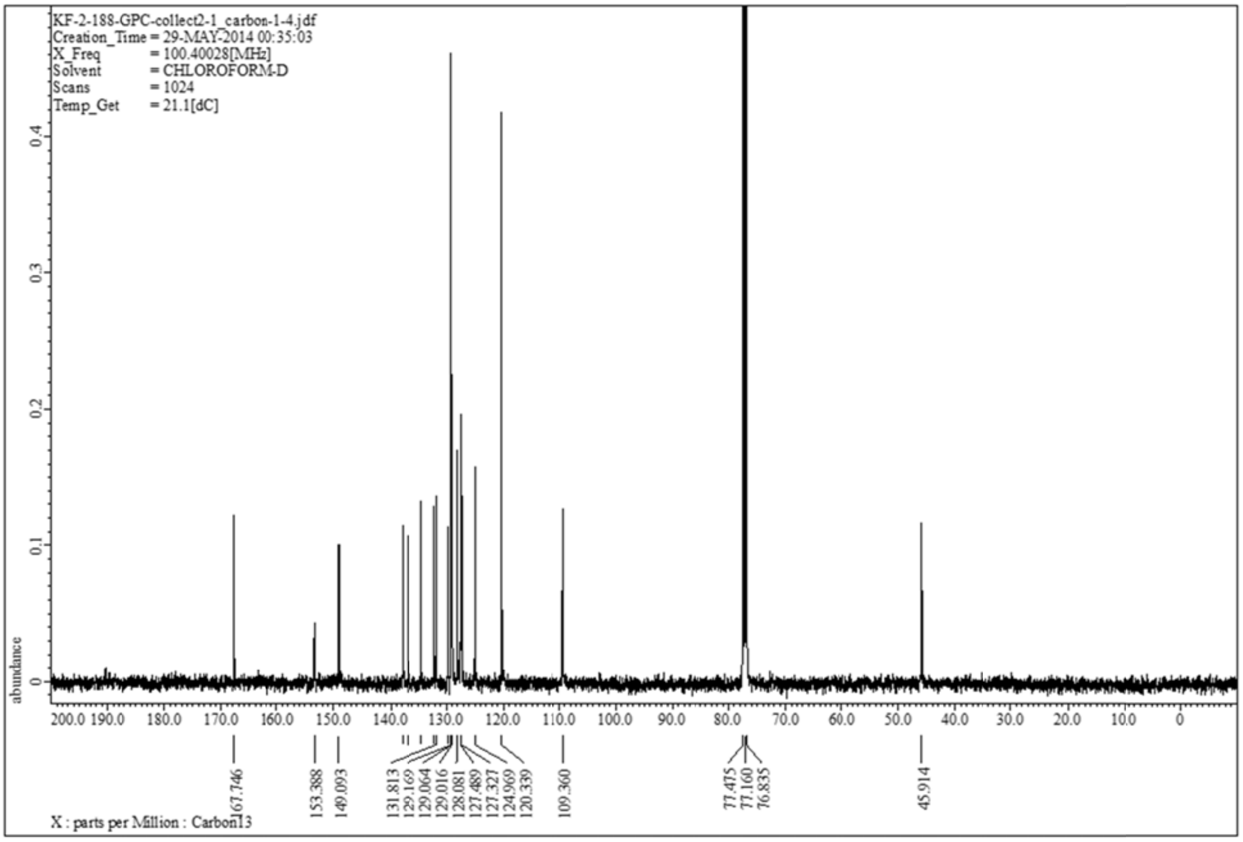

Supplementary Figure 10. ^{1}H and ^{13}C-NMR spectra for catalyst **1f**

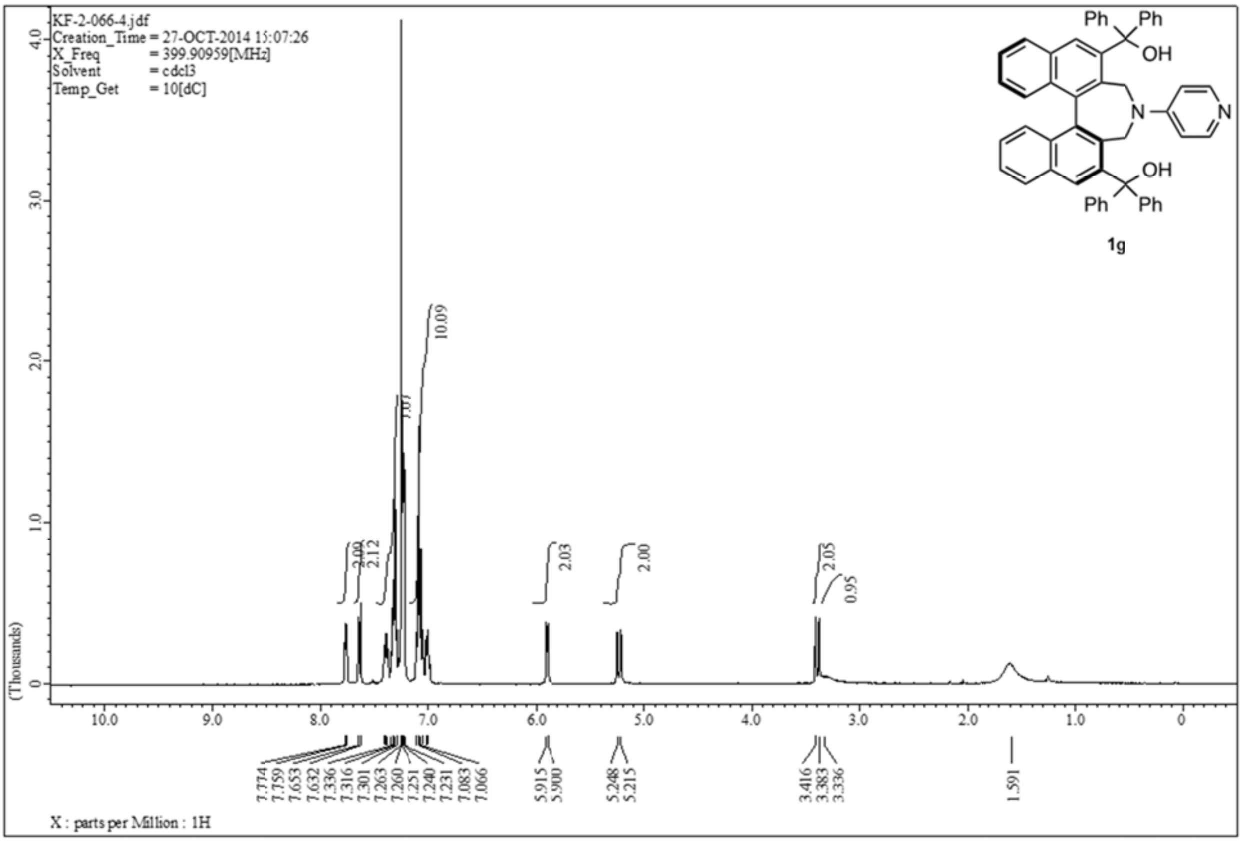

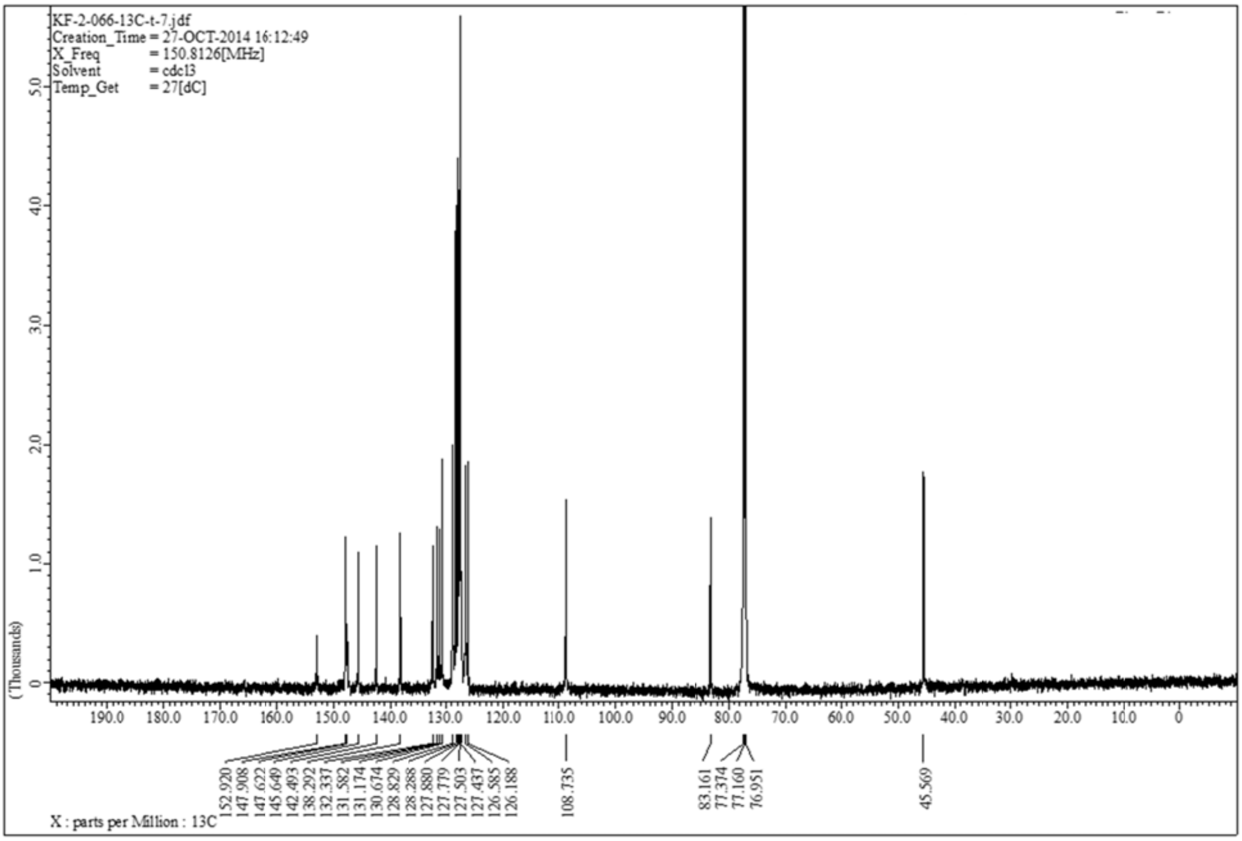

Supplementary Figure 11. ^{1}H and ^{13}C-NMR spectra for catalyst **1g**

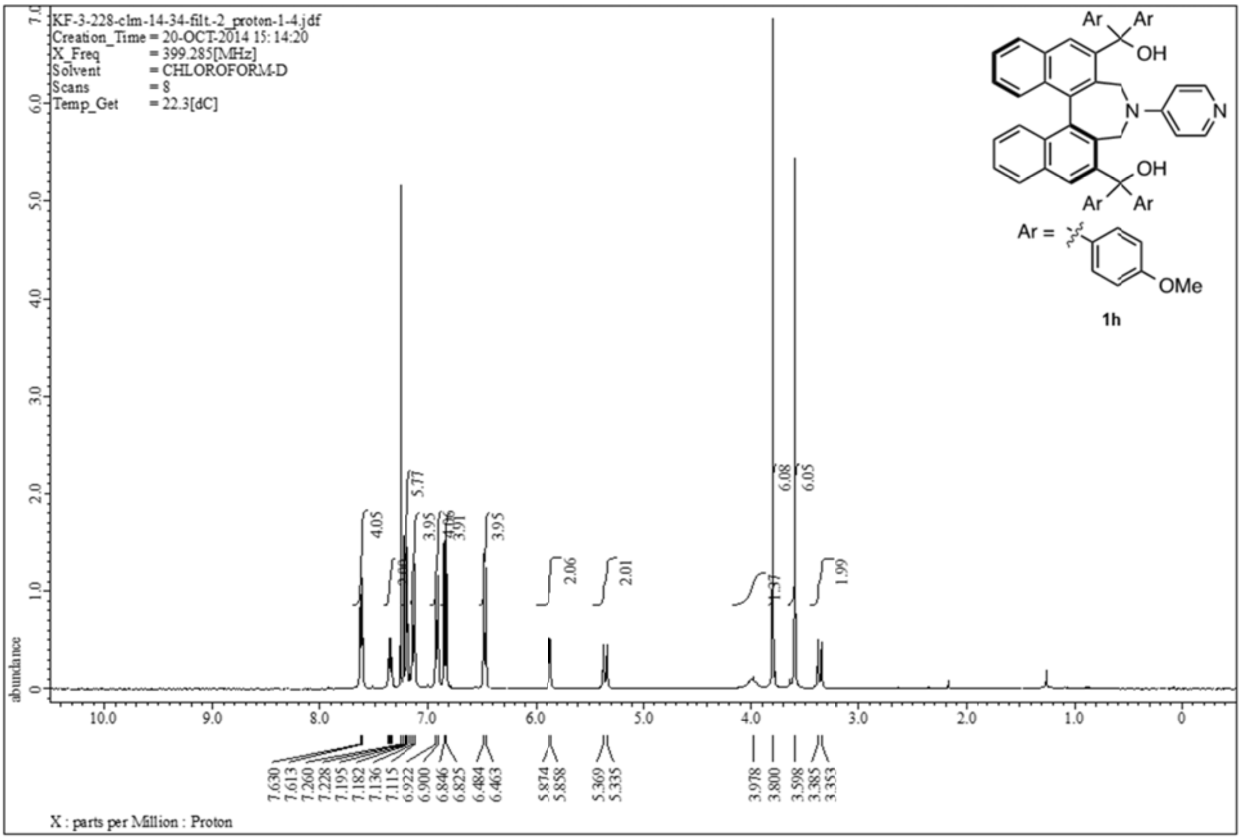

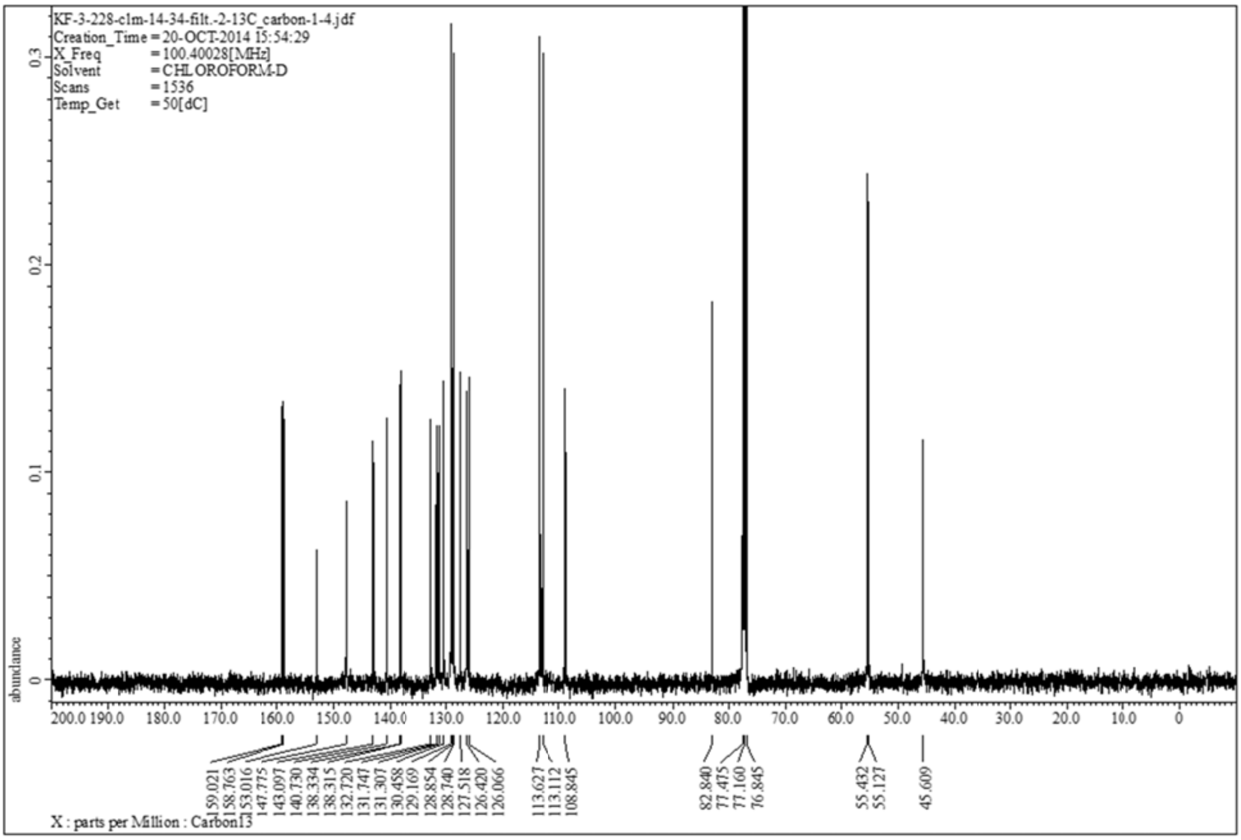

Supplementary Figure 12. ^{1}H and ^{13}C-NMR spectra for catalyst **1h**

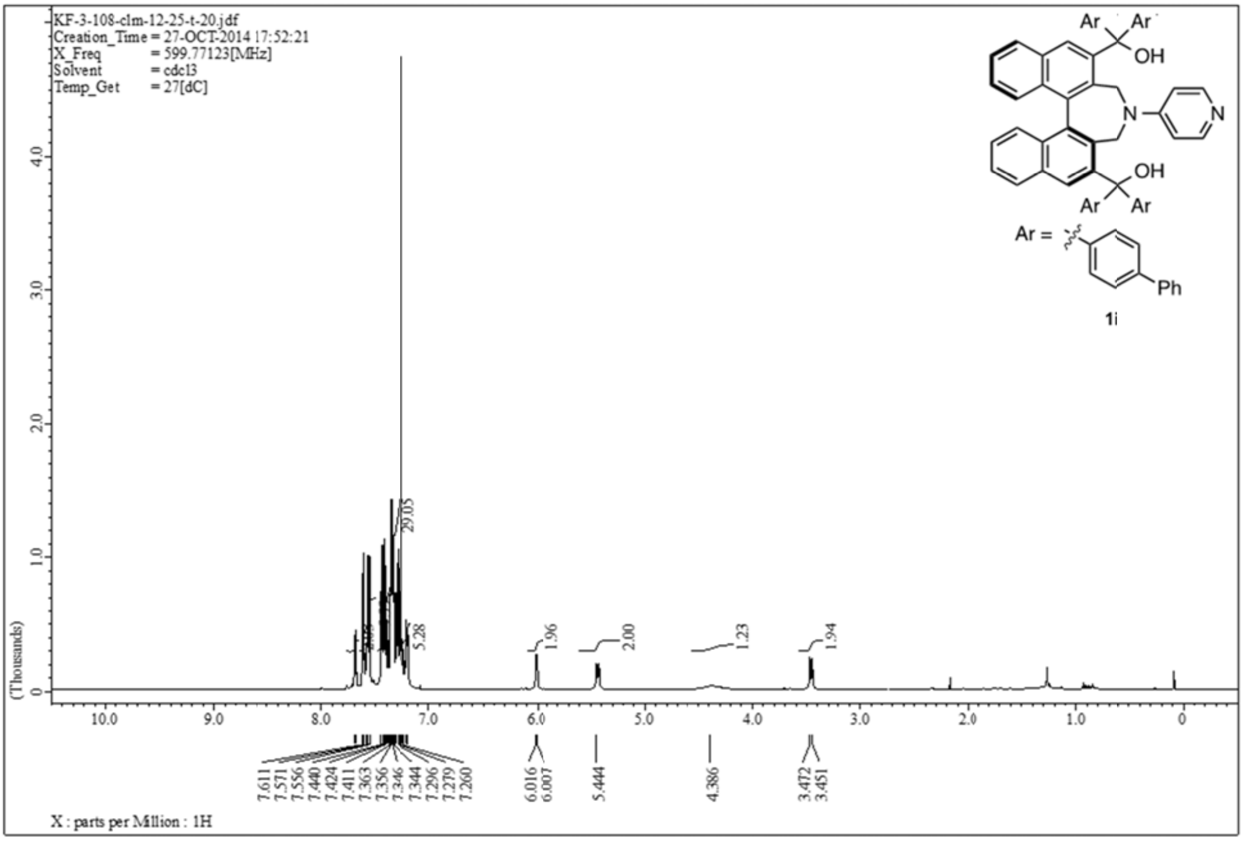

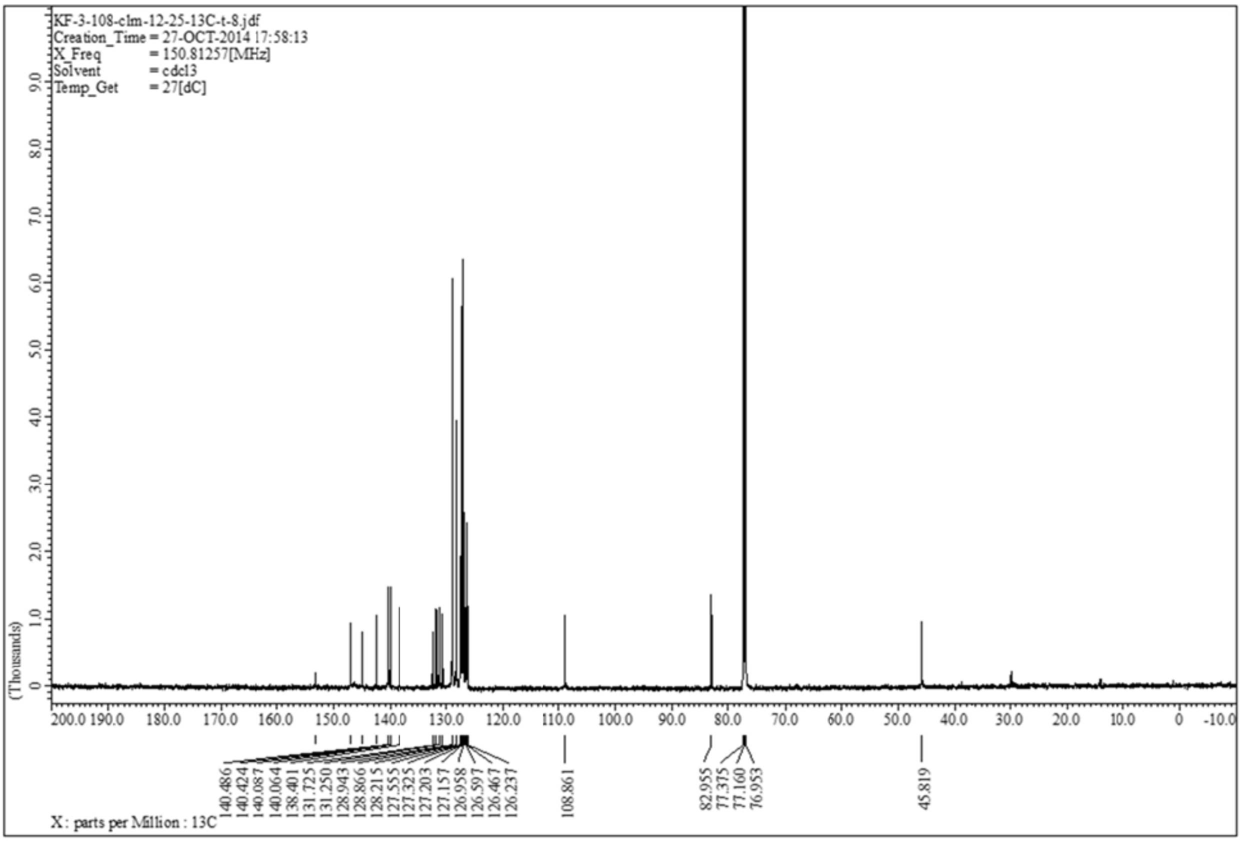

Supplementary Figure 13. ^{1}H and ^{13}C-NMR spectra for catalyst **1i**

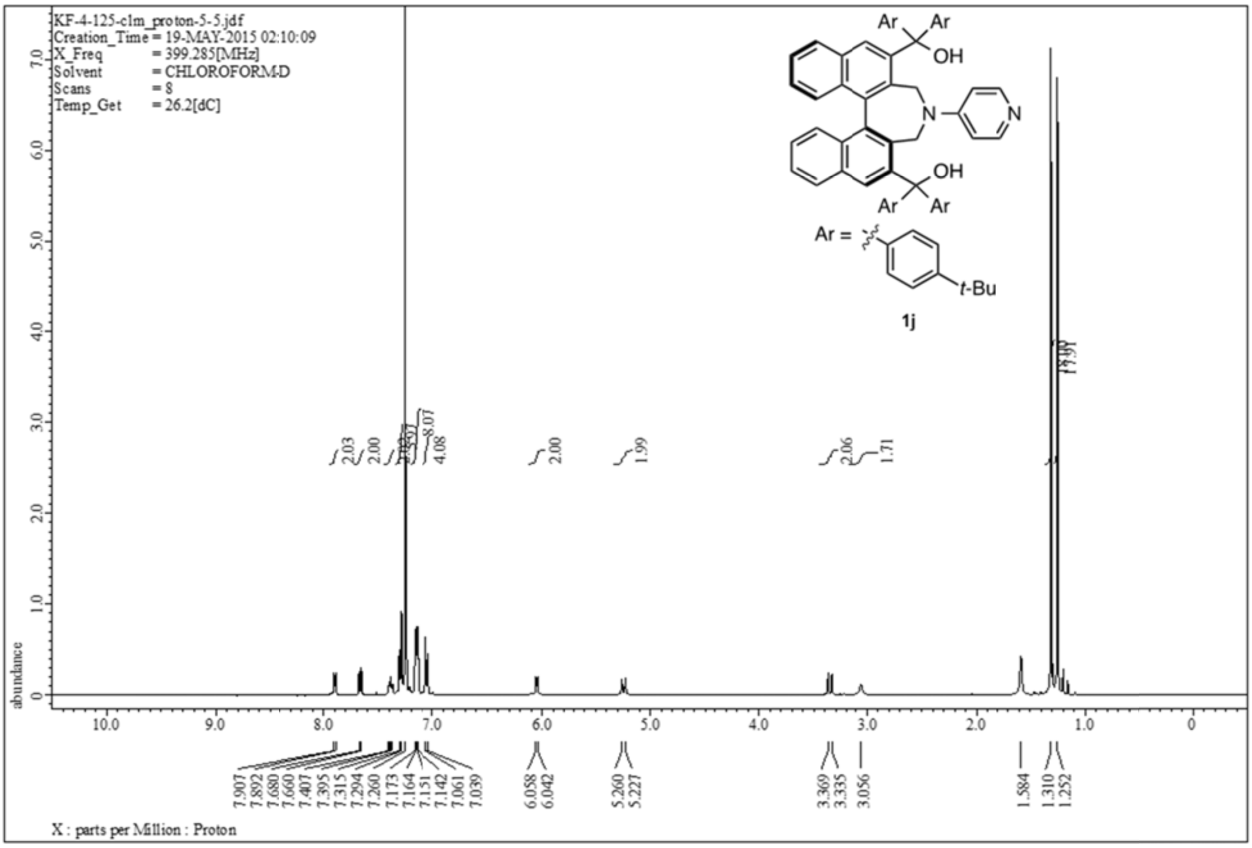

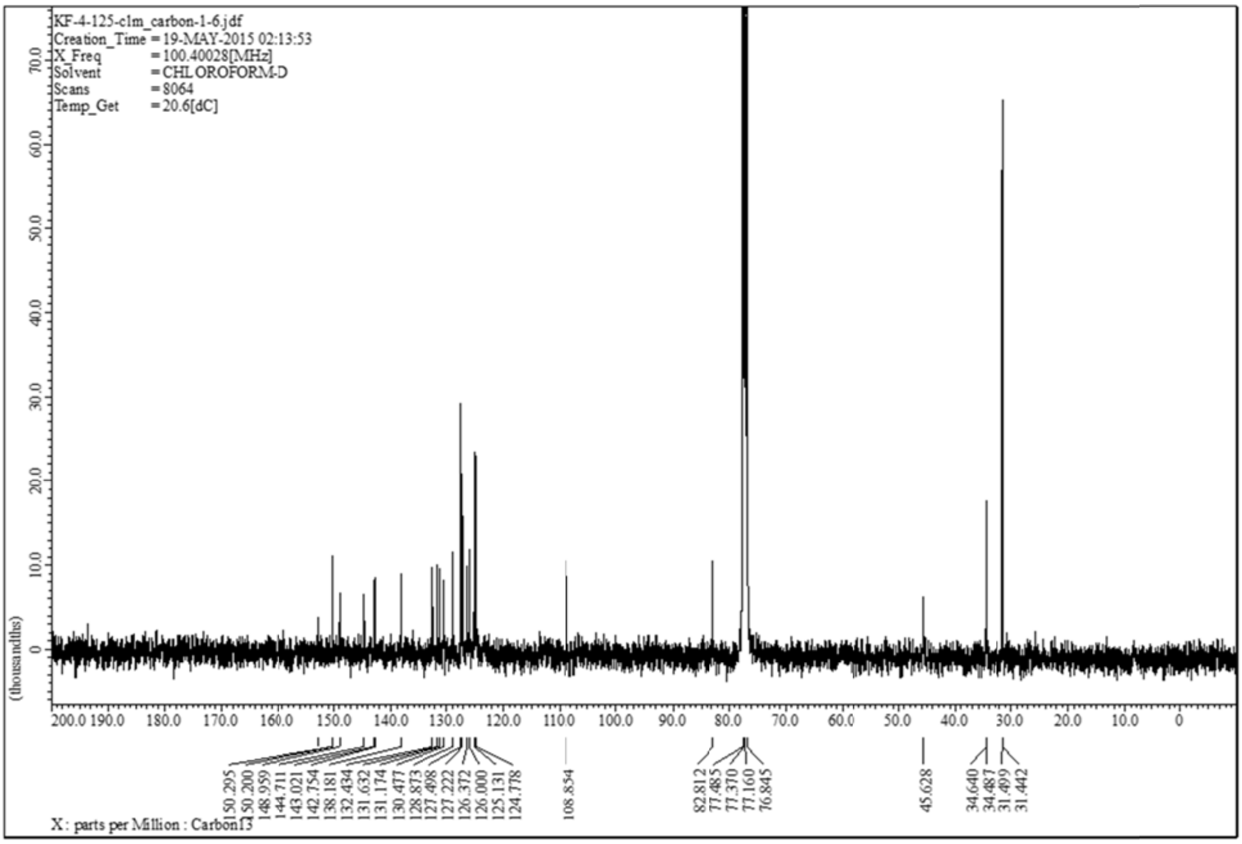

Supplementary Figure 14. ^{1}H and ^{13}C-NMR spectra for catalyst **1j**

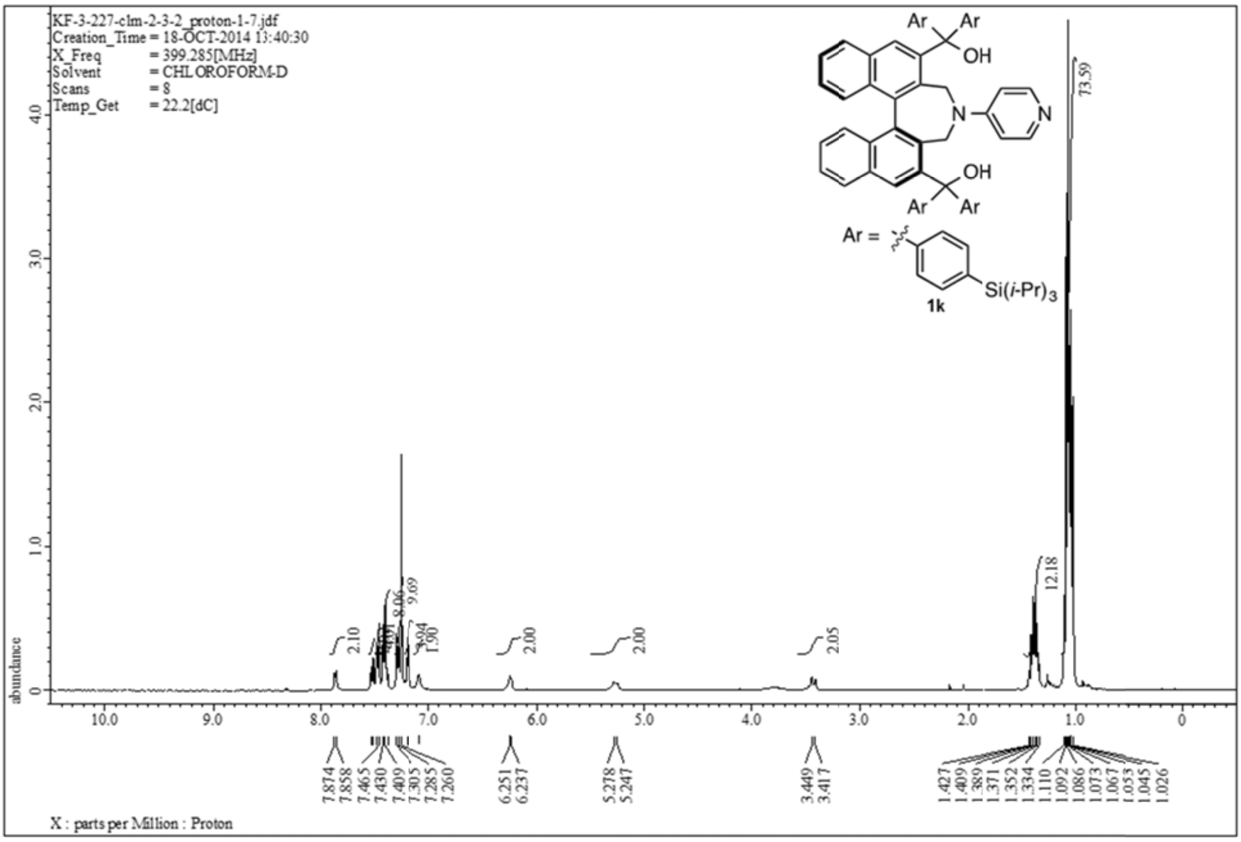

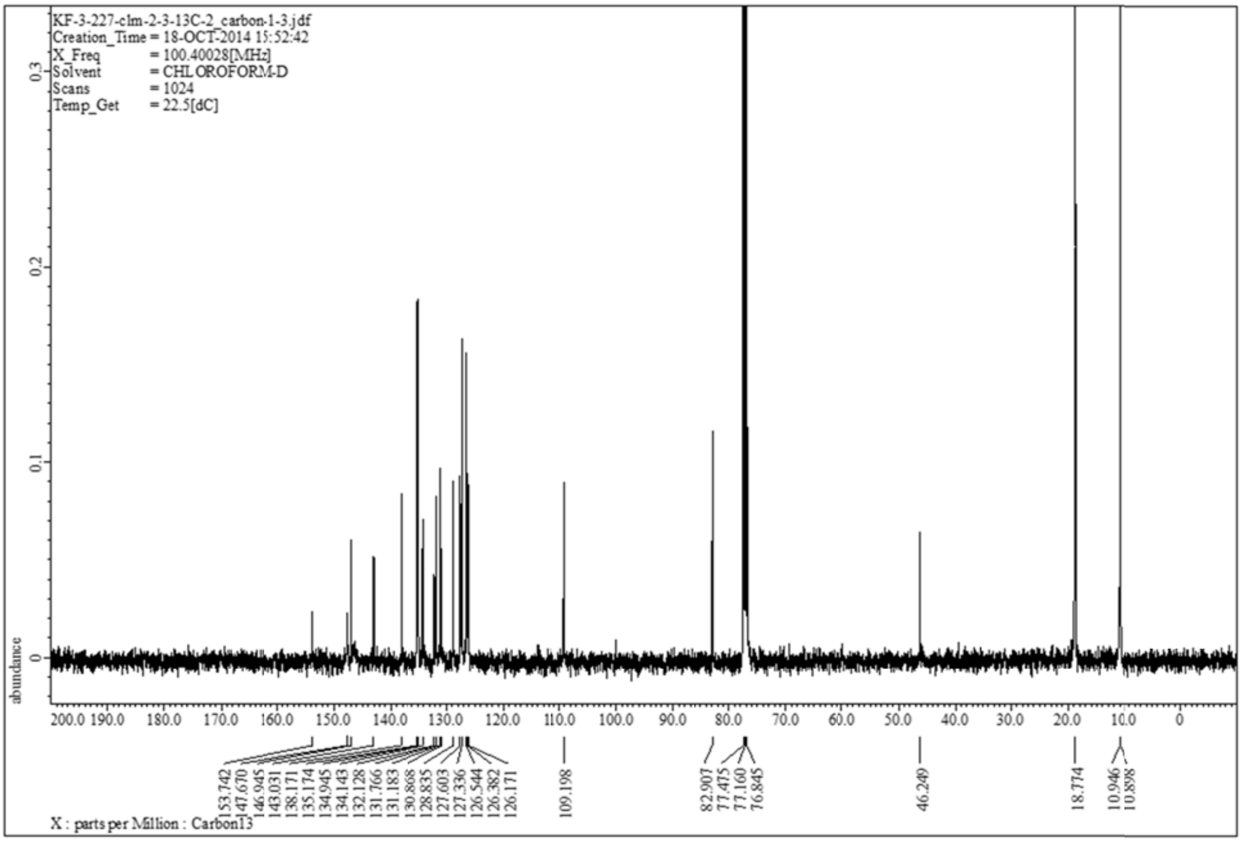

Supplementary Figure 15. ^{1}H and ^{13}C-NMR spectra for catalyst **1k**

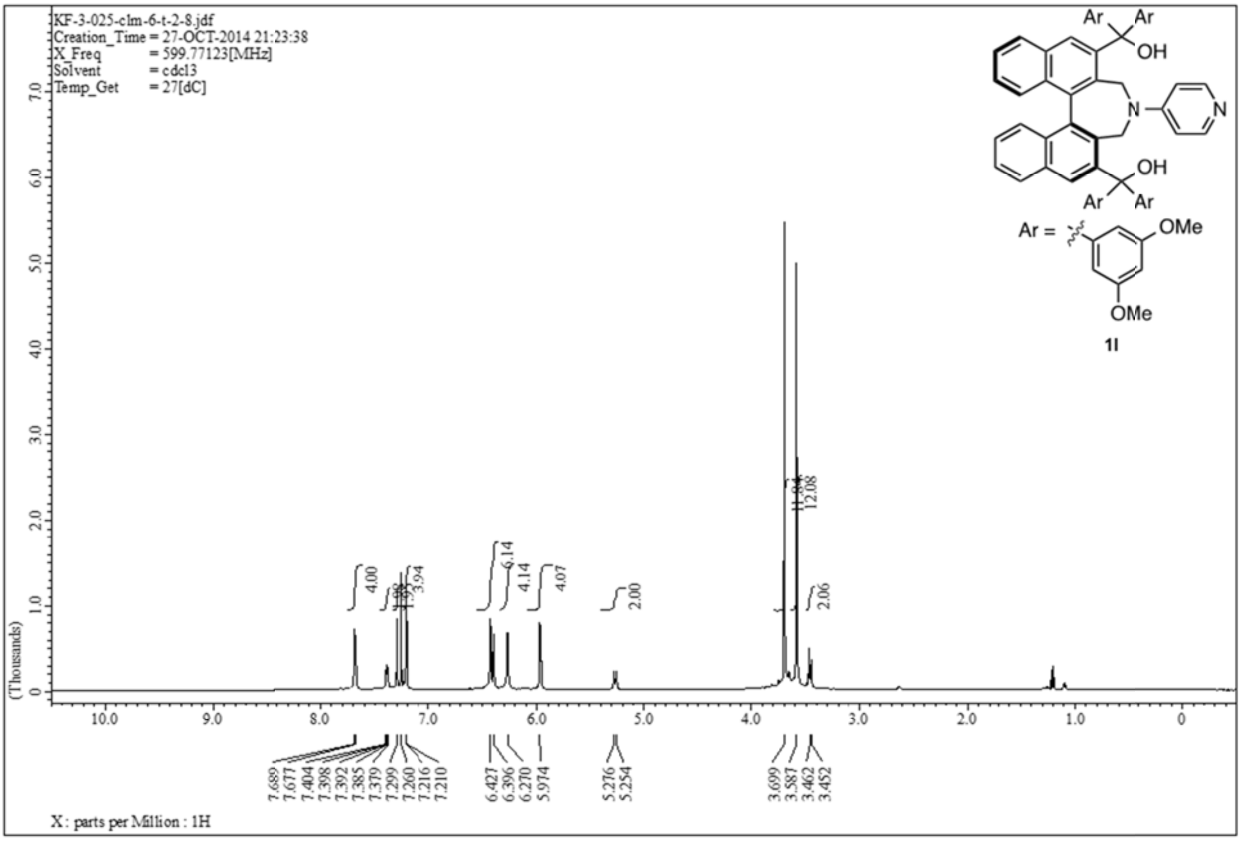

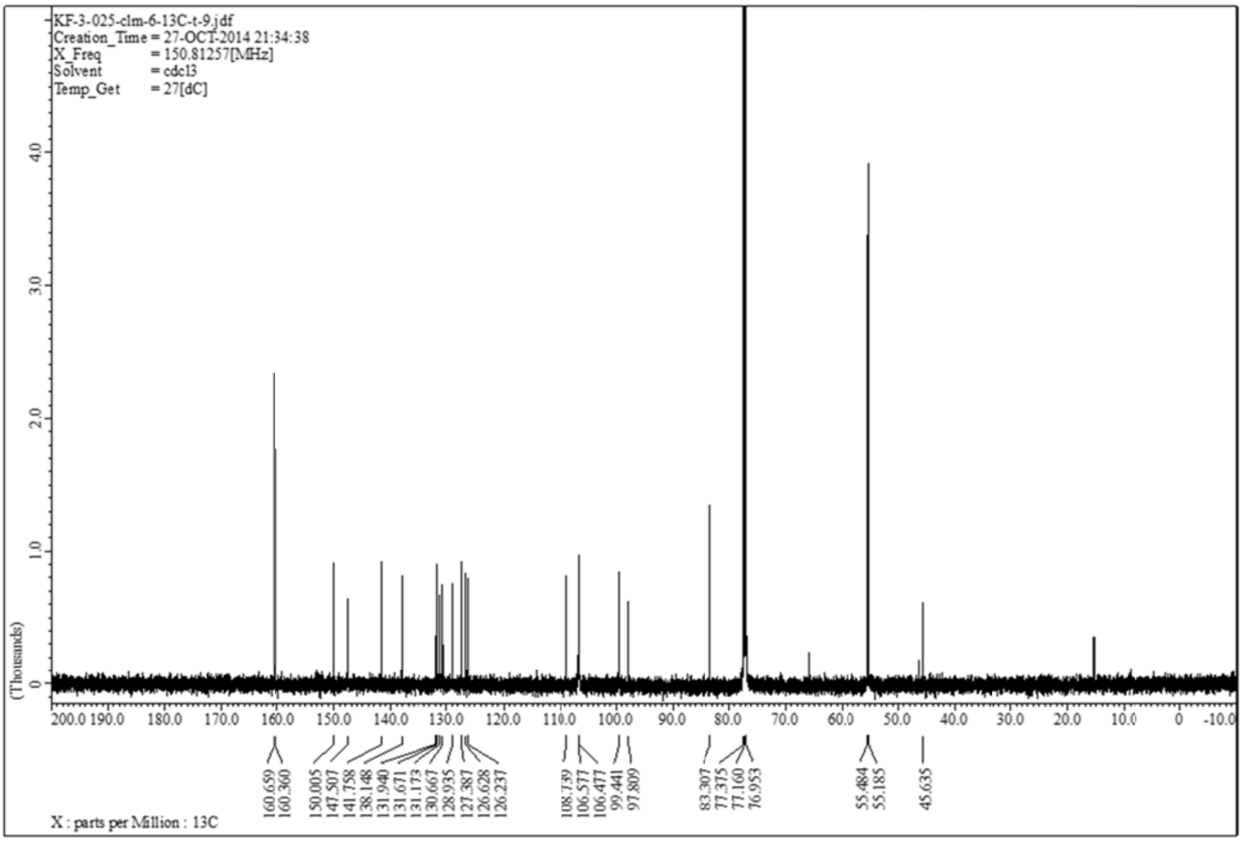

Supplementary Figure 16. ^{1}H and ^{13}C-NMR spectra for catalyst **1l**

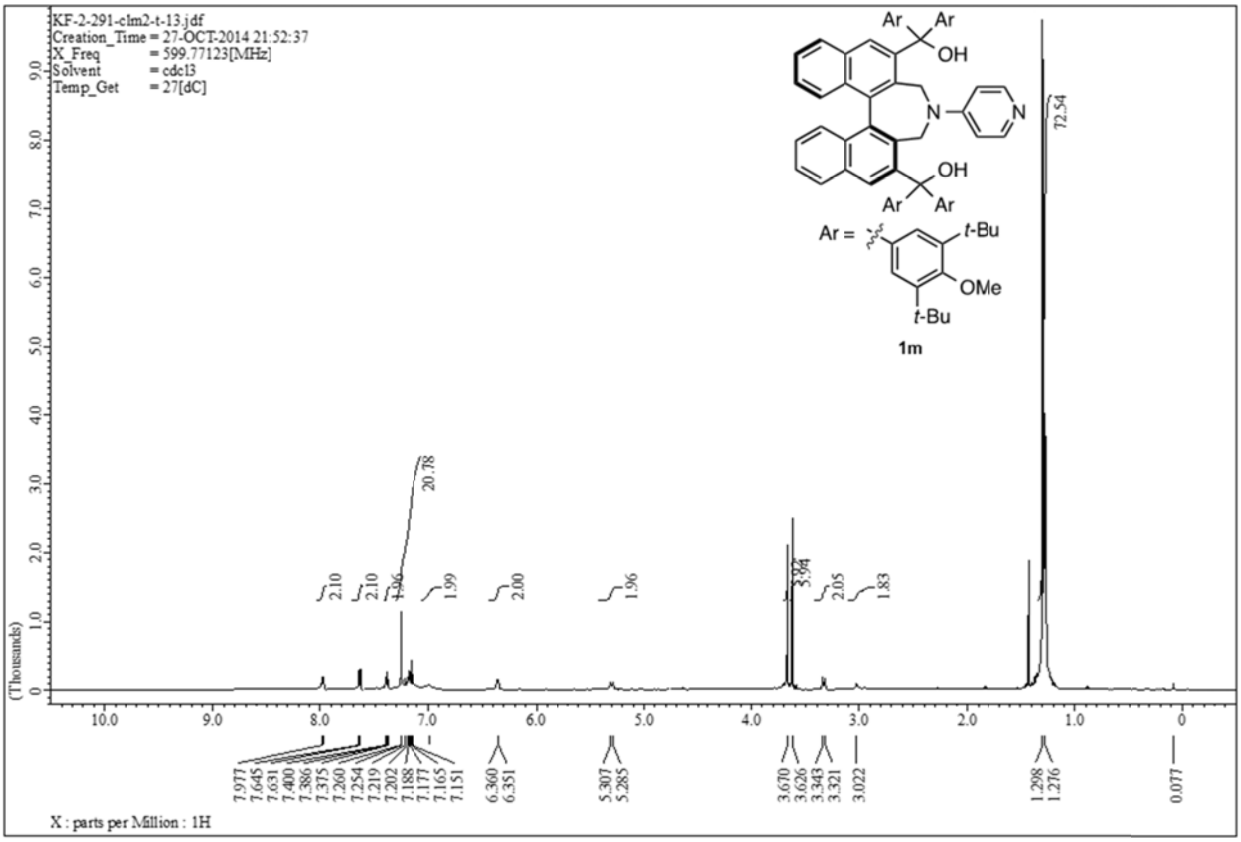

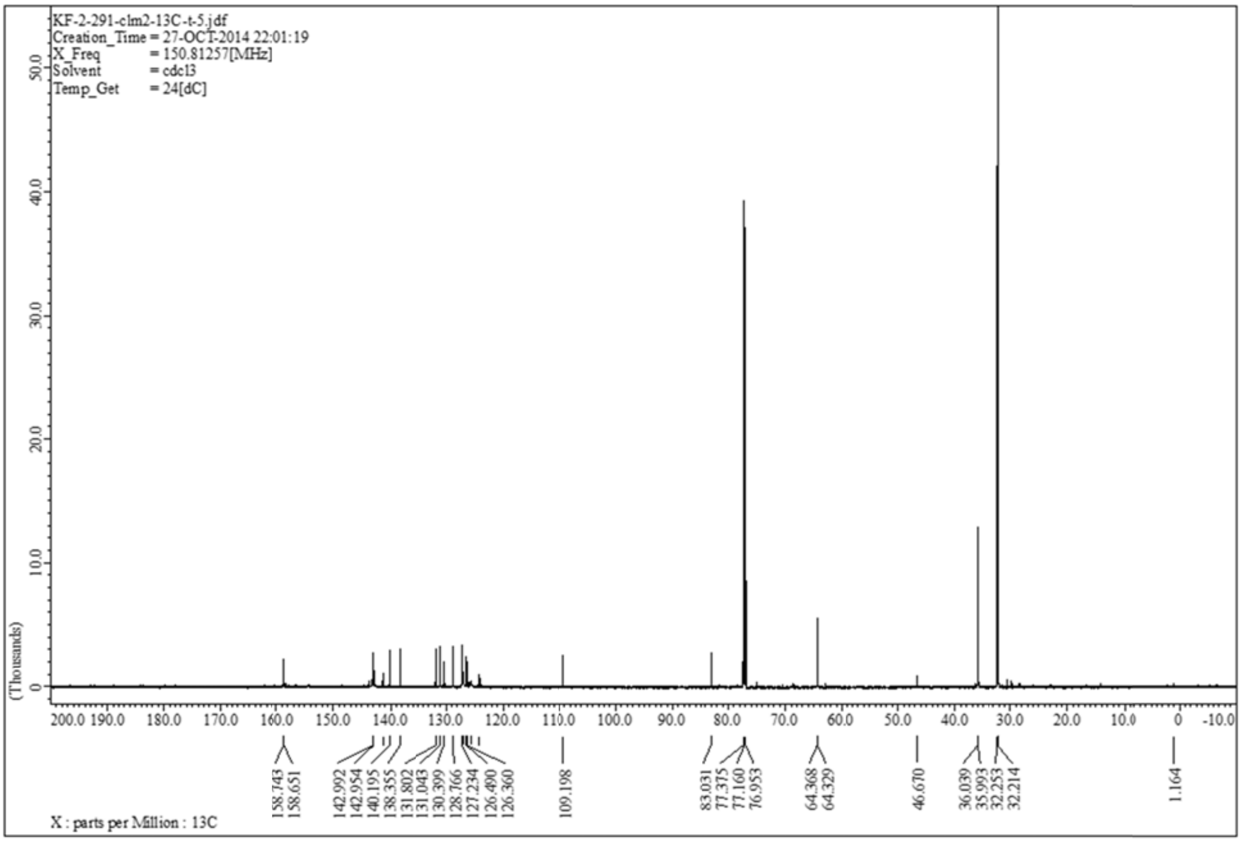

Supplementary Figure 17. ^{1}H and ^{13}C-NMR spectra for catalyst **1m**

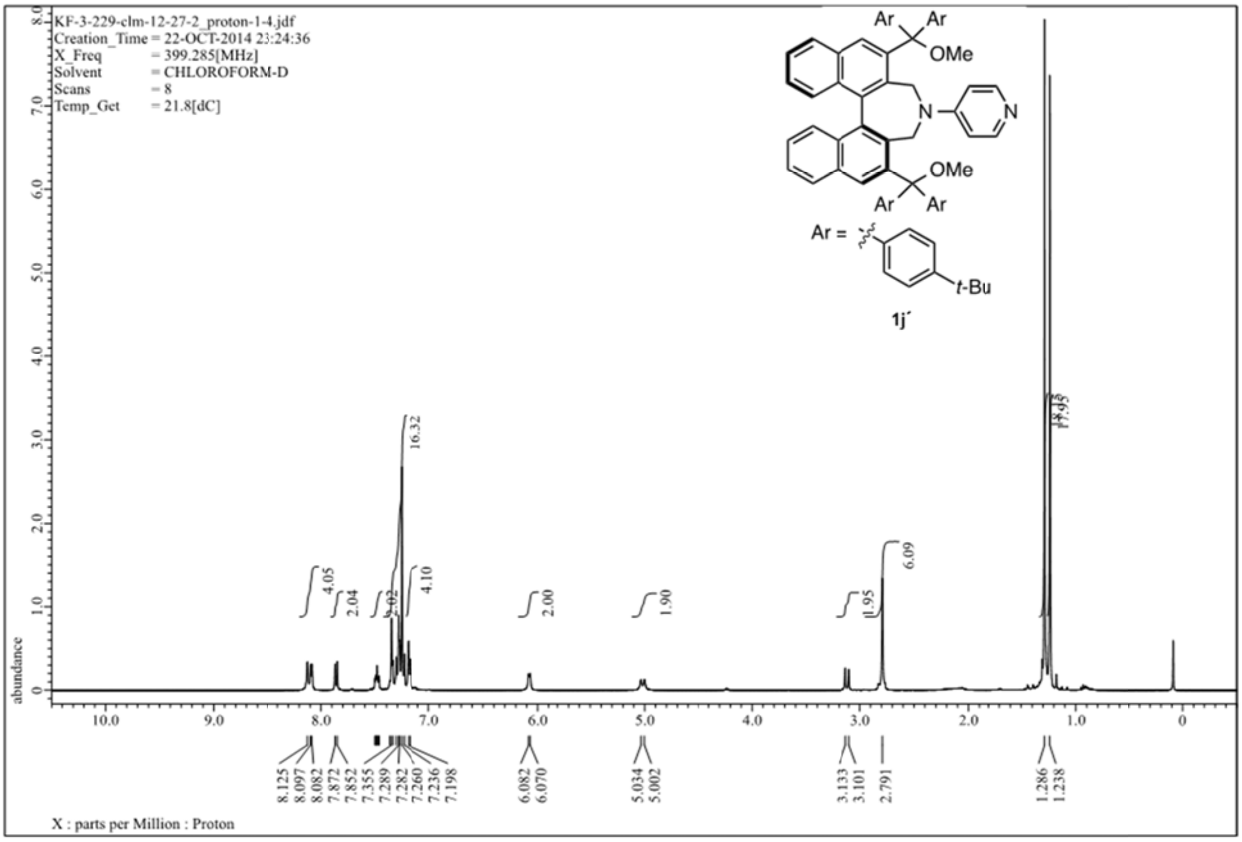

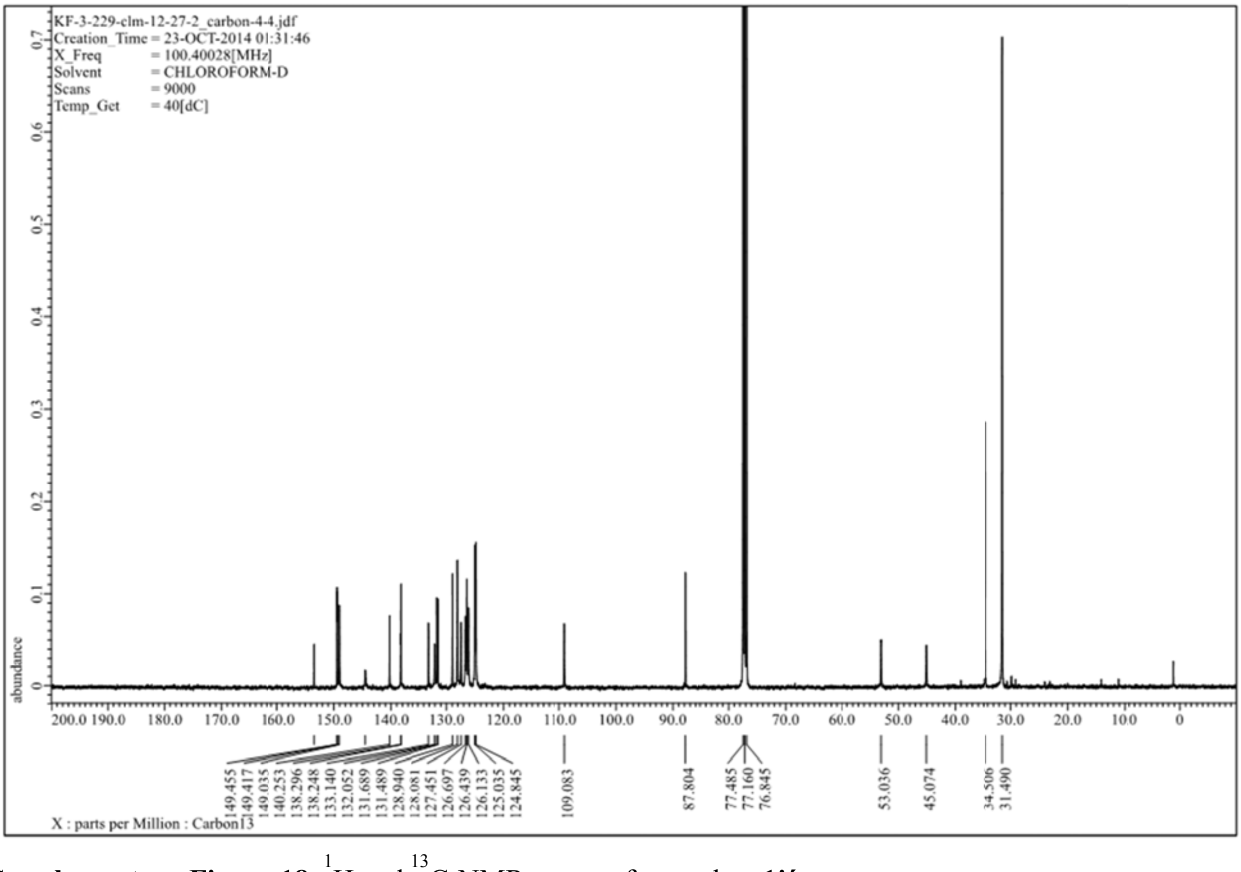

Supplementary Figure 18. ^{1}H and ^{13}C-NMR spectra for catalyst **1j´**

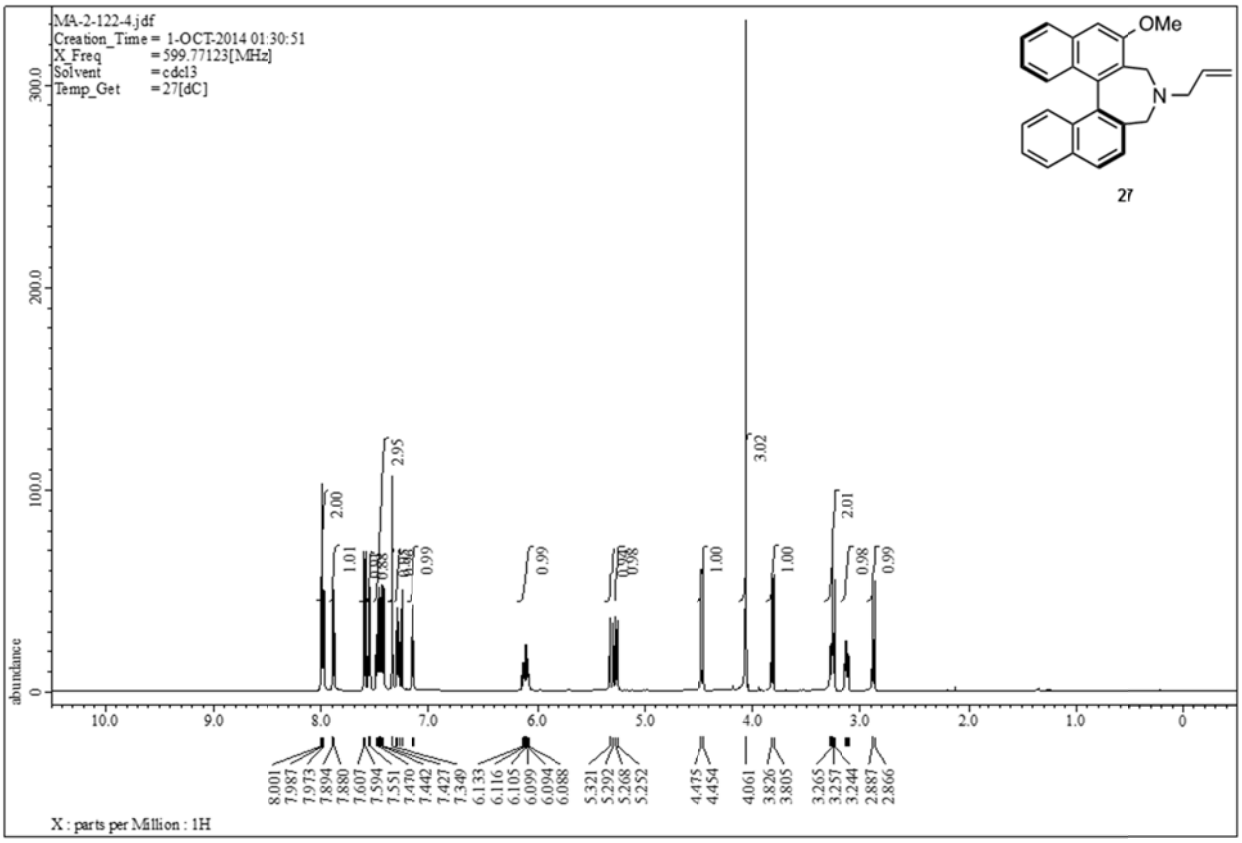

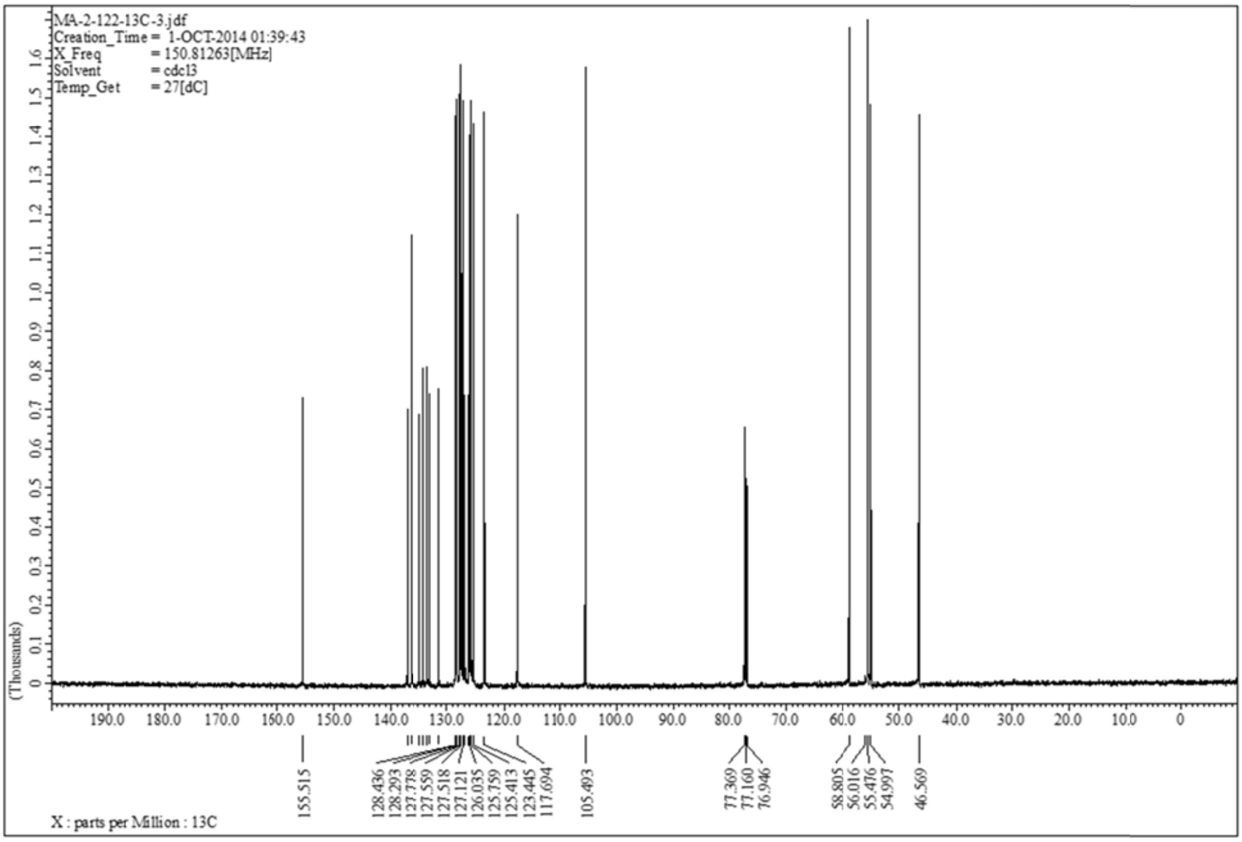

Supplementary Figure 19. ^{1}H and ^{13}C-NMR spectra for **27**

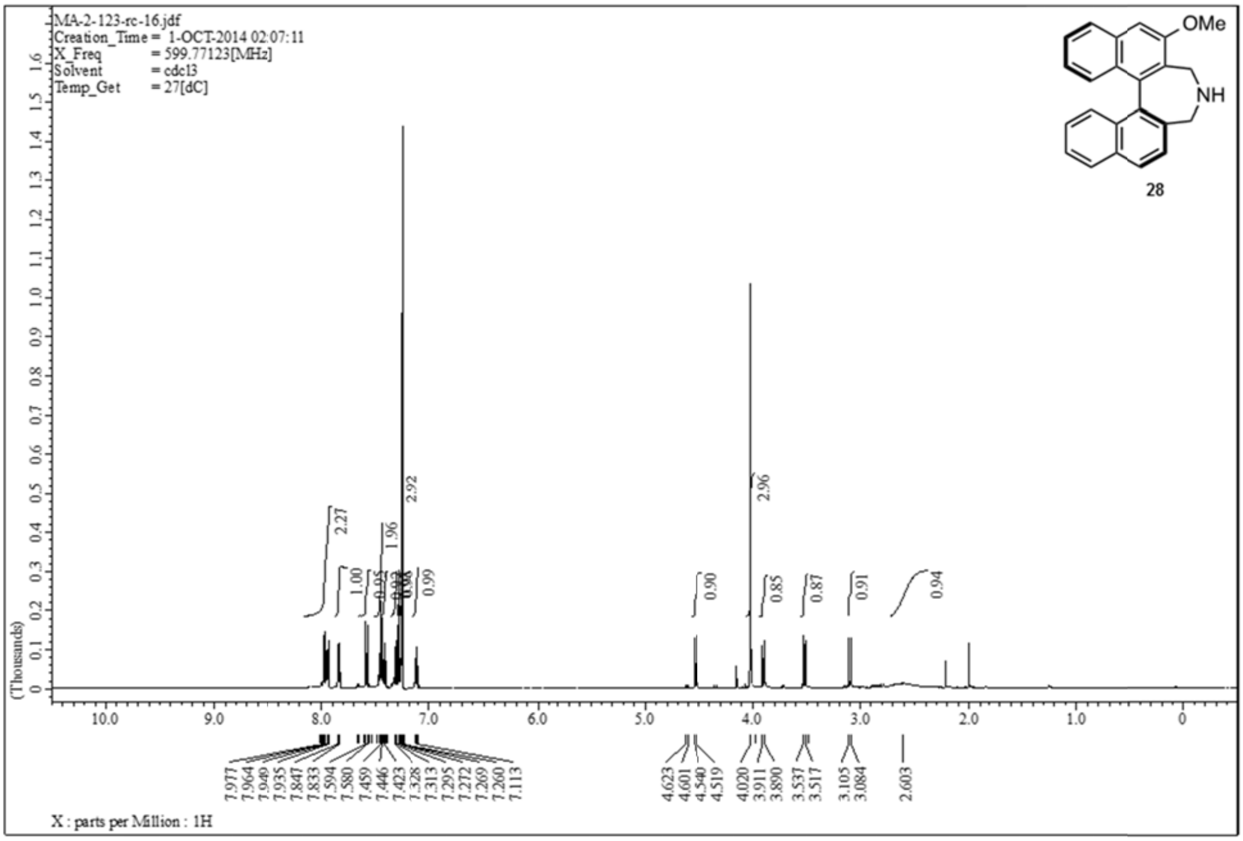

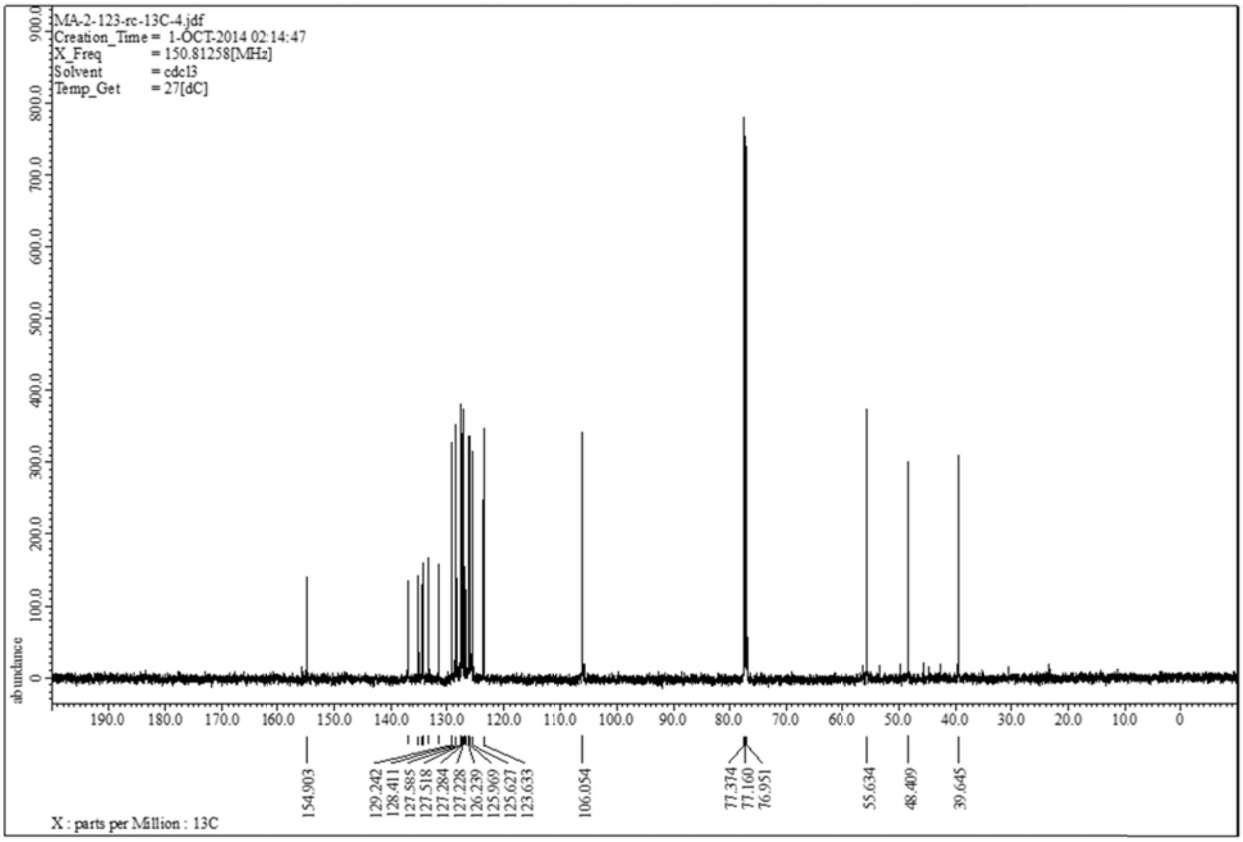

Supplementary Figure 20. ^{1}H and ^{13}C-NMR spectra for **28**

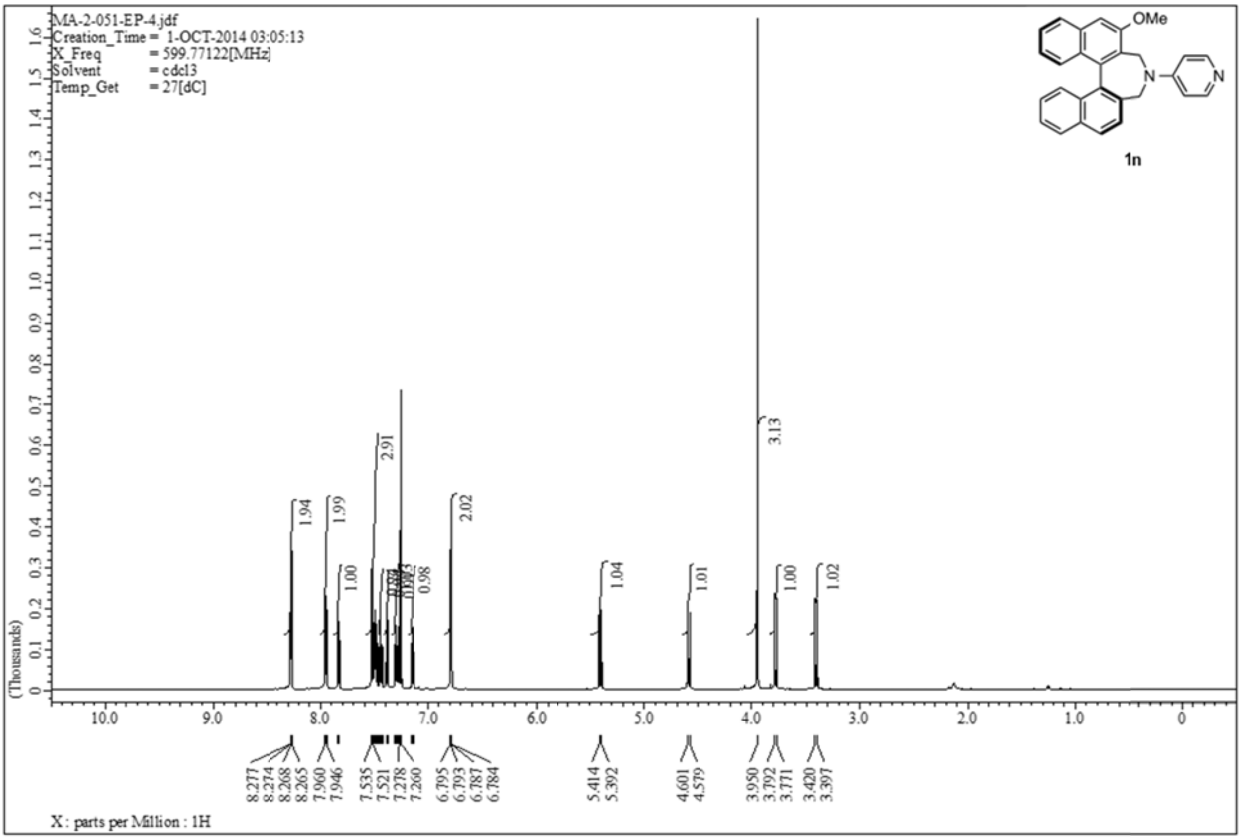

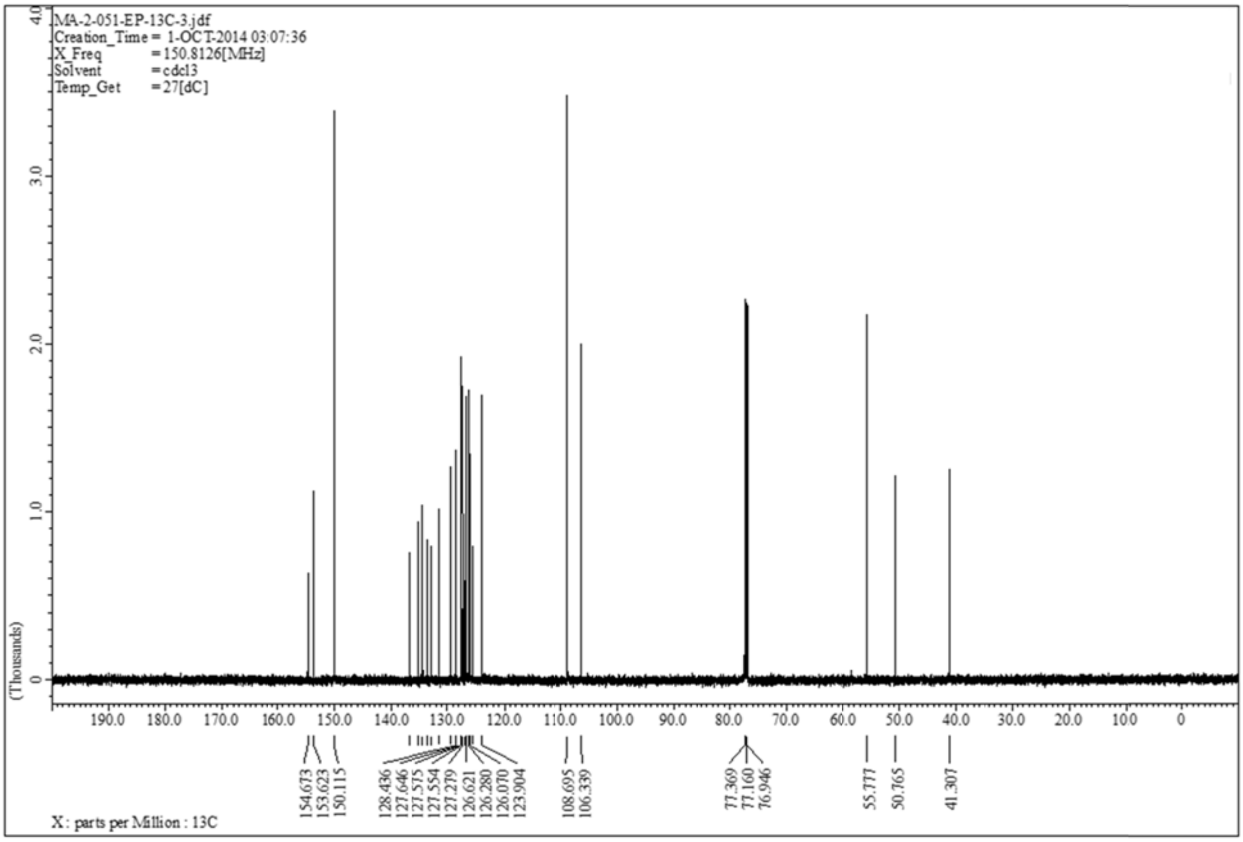

Supplementary Figure 21. ^{1}H and ^{13}C-NMR spectra for catalyst **1n**

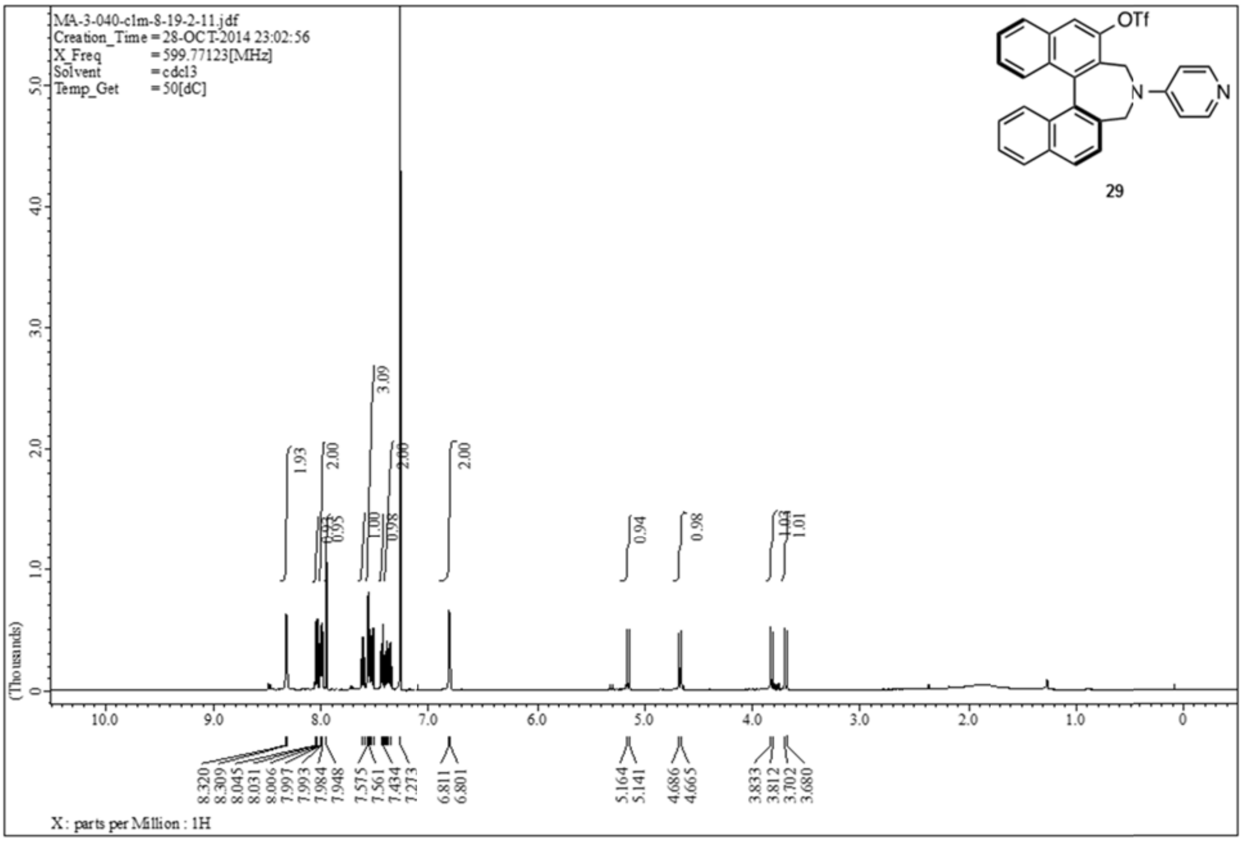

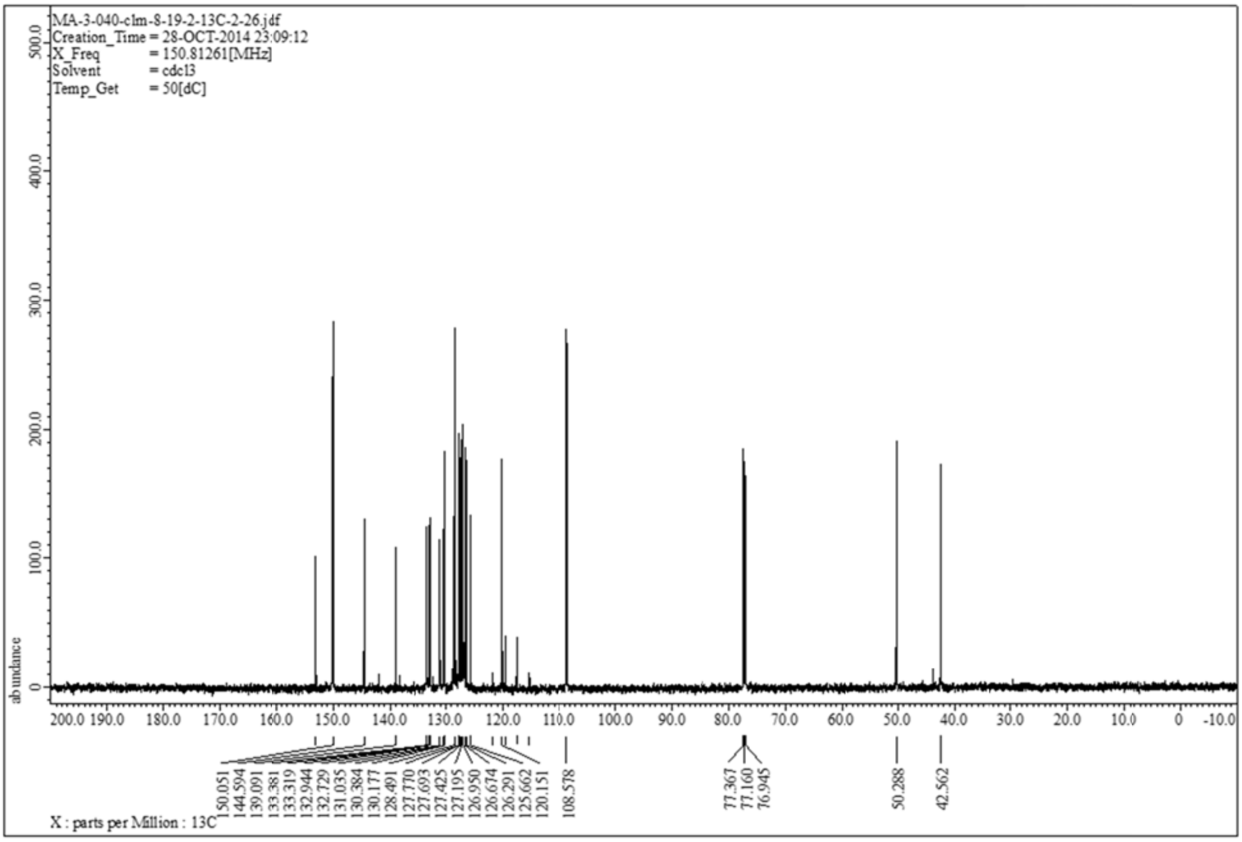

Supplementary Figure 22. ^{1}H and ^{13}C-NMR spectra for **29**

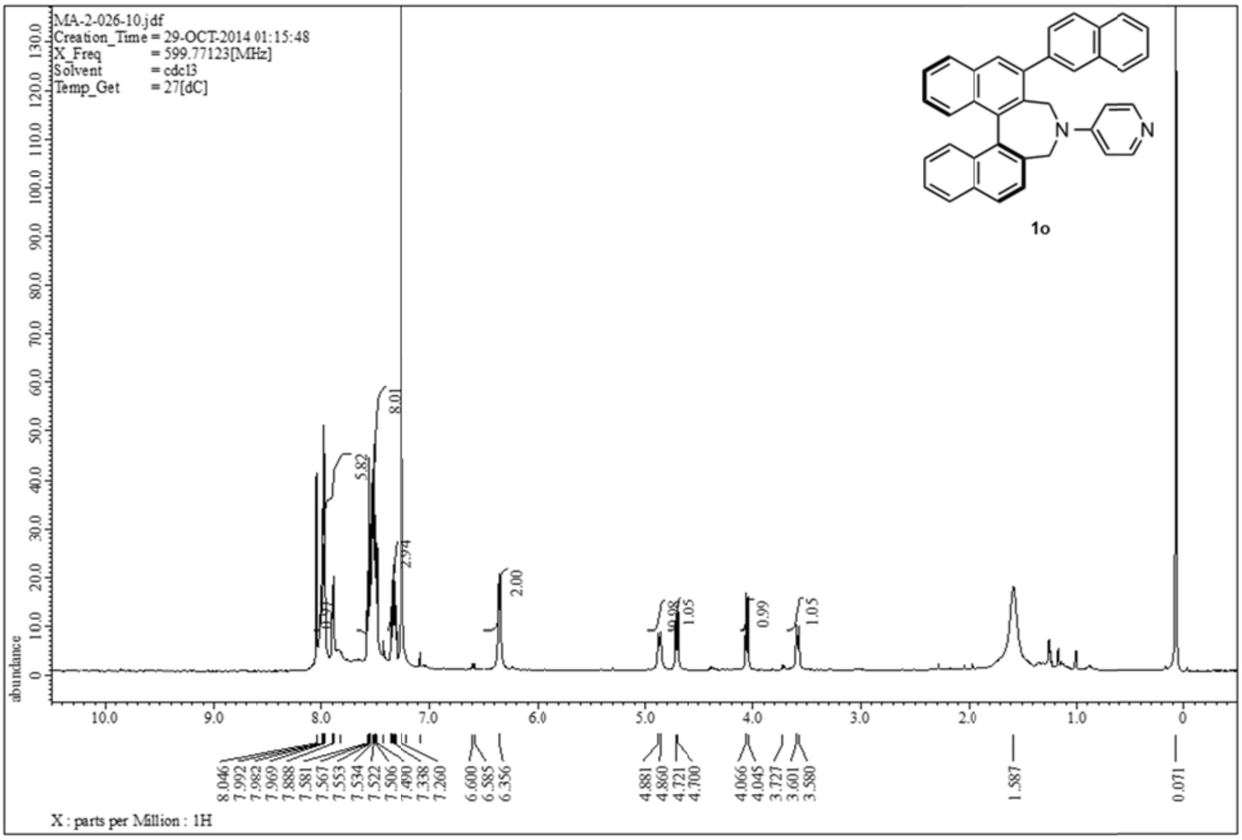

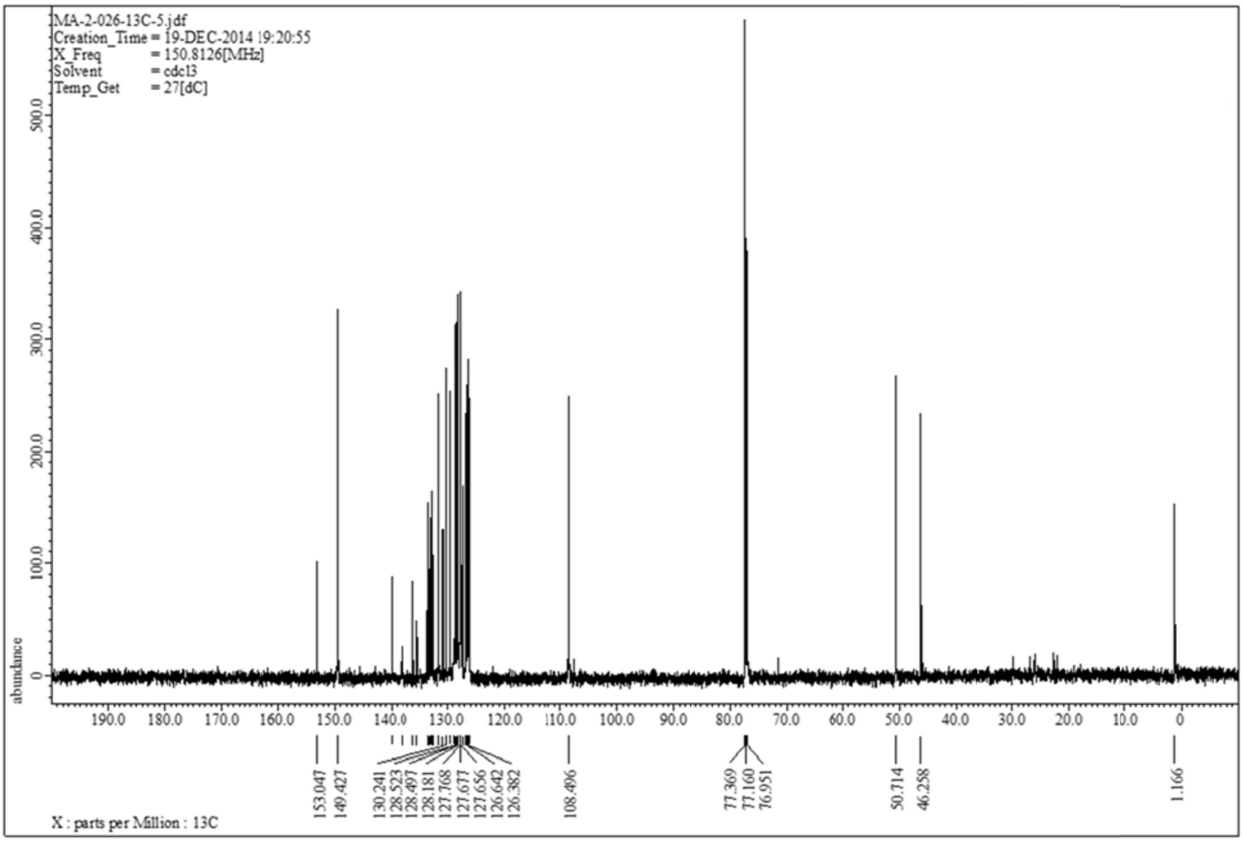

Supplementary Figure 23. ^{1}H and ^{13}C-NMR spectra for catalyst **1o**

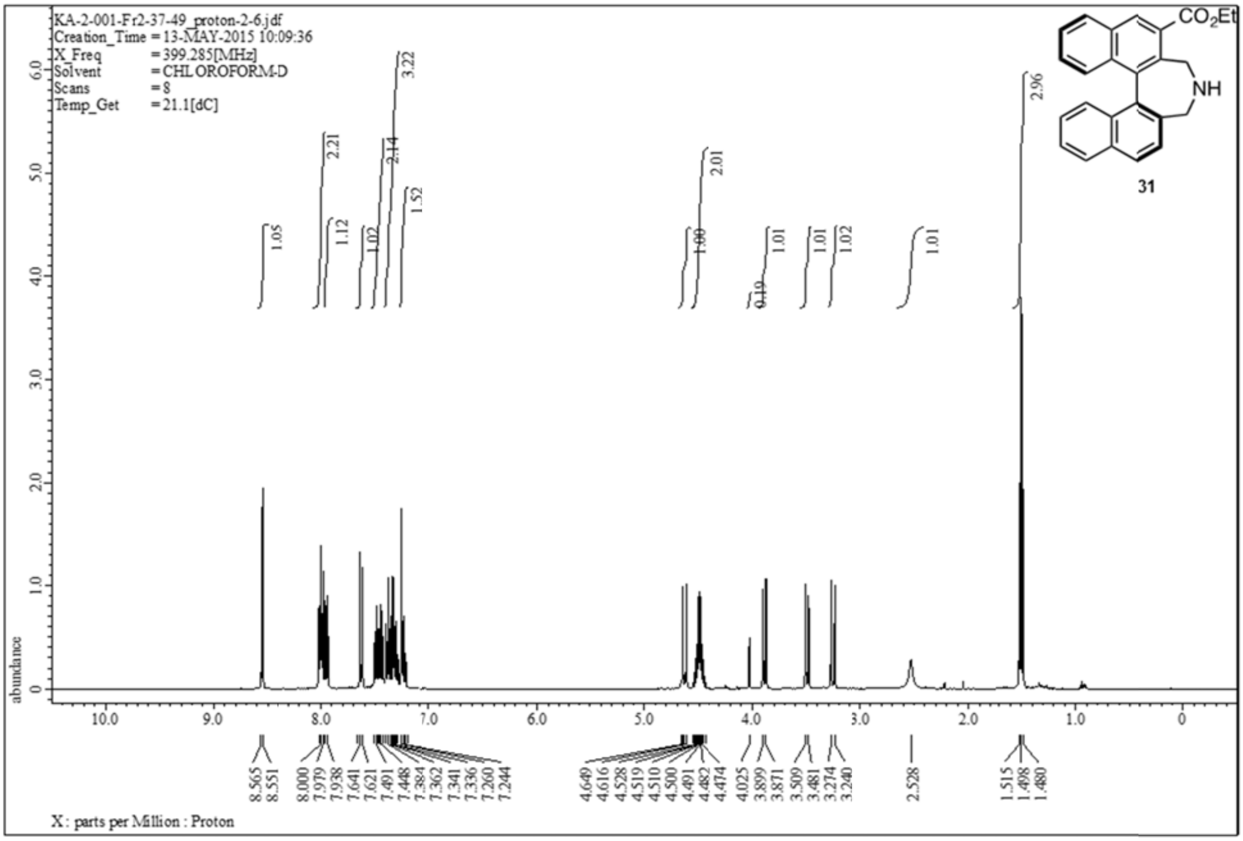

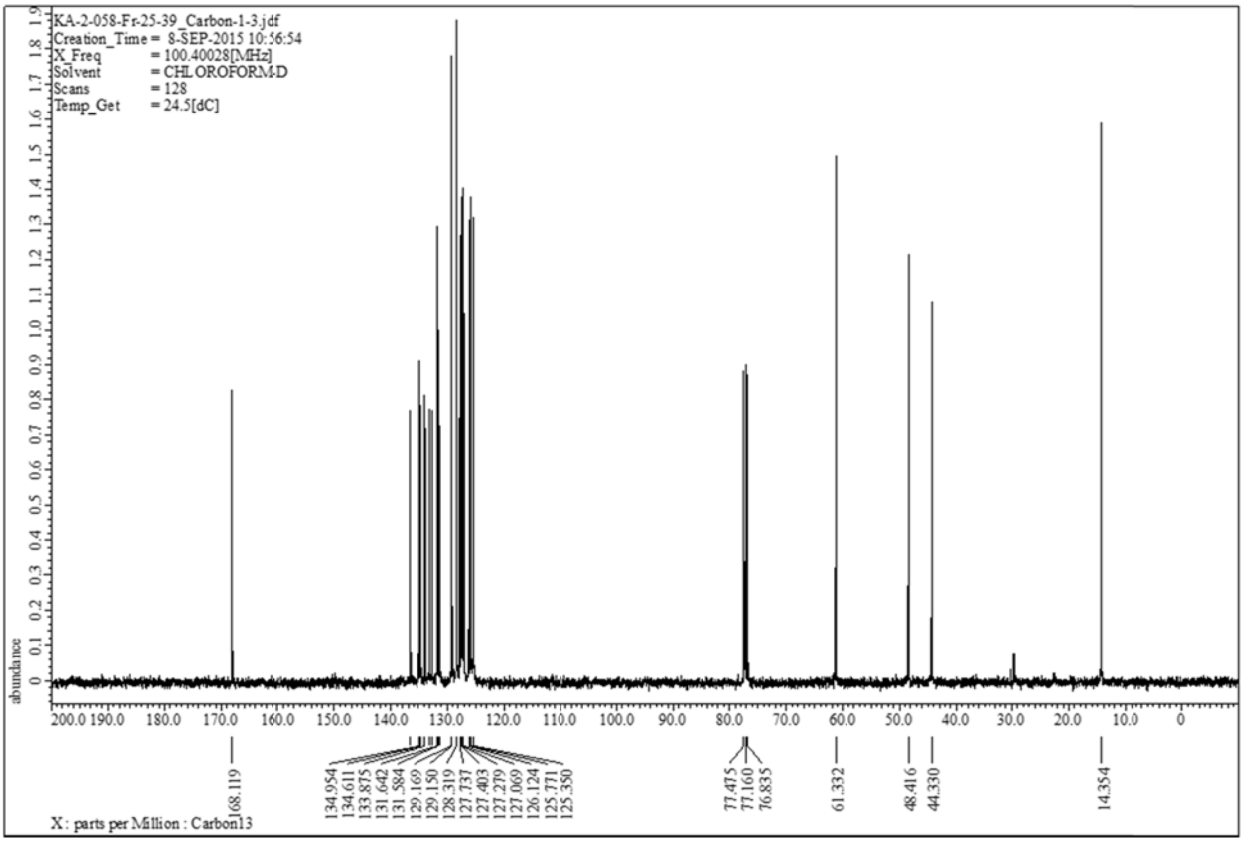

Supplementary Figure 24. ^{1}H and ^{13}C-NMR spectra for **31**

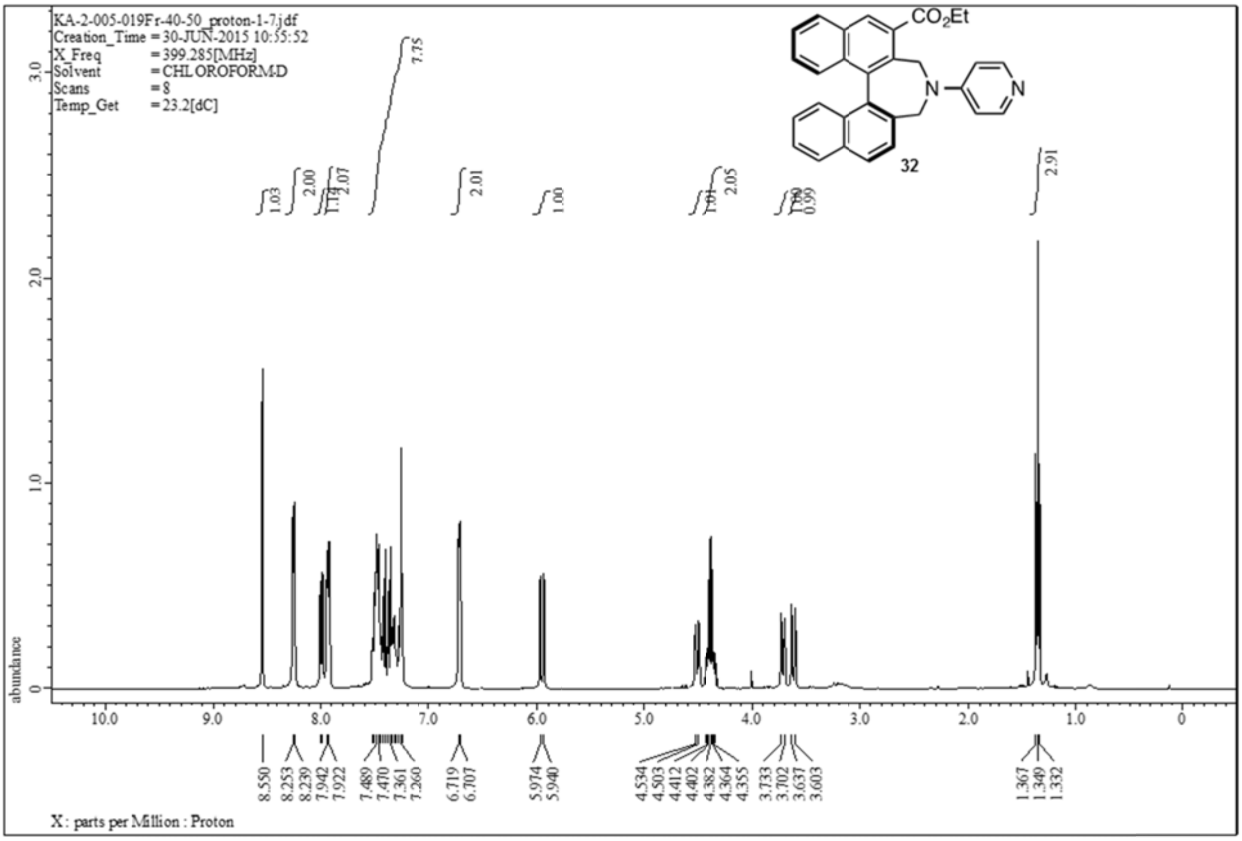

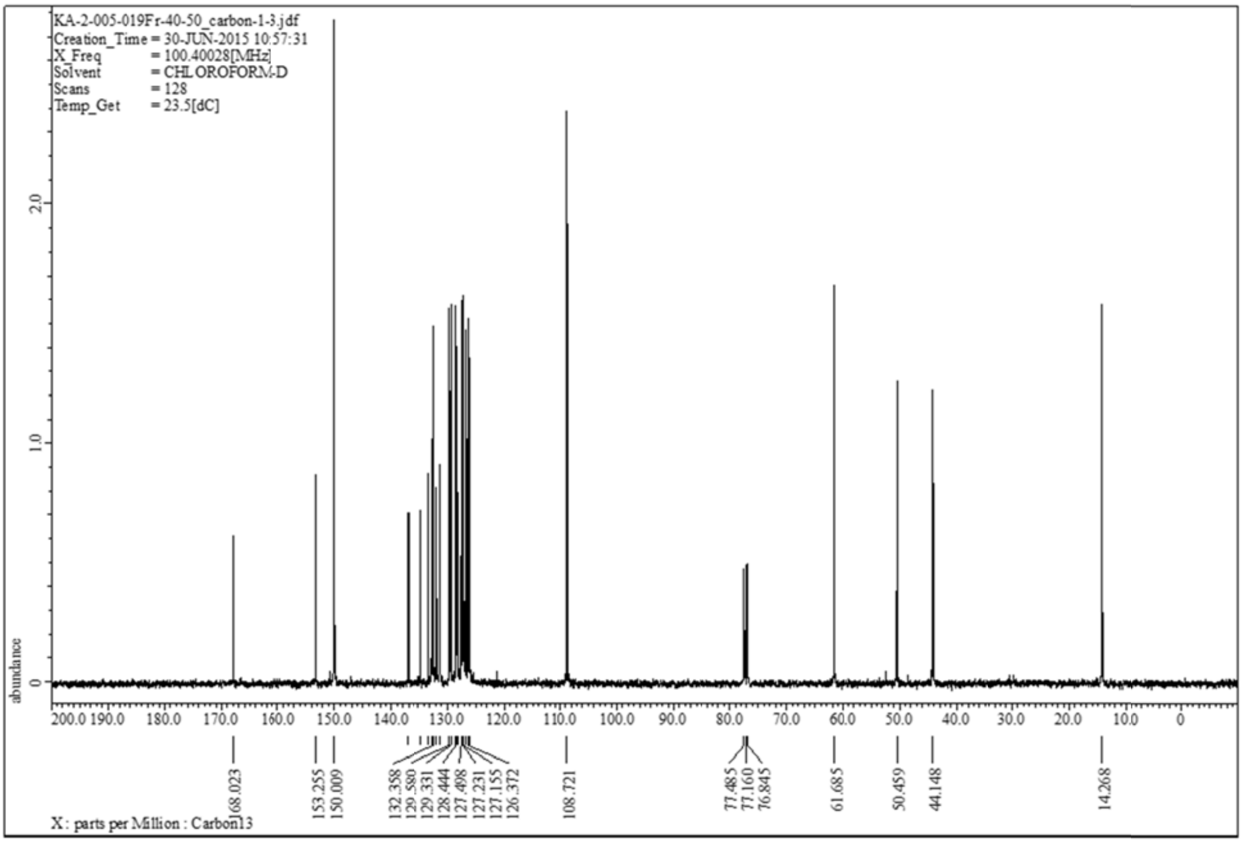

Supplementary Figure 25. ^{1}H and ^{13}C-NMR spectra for **32**

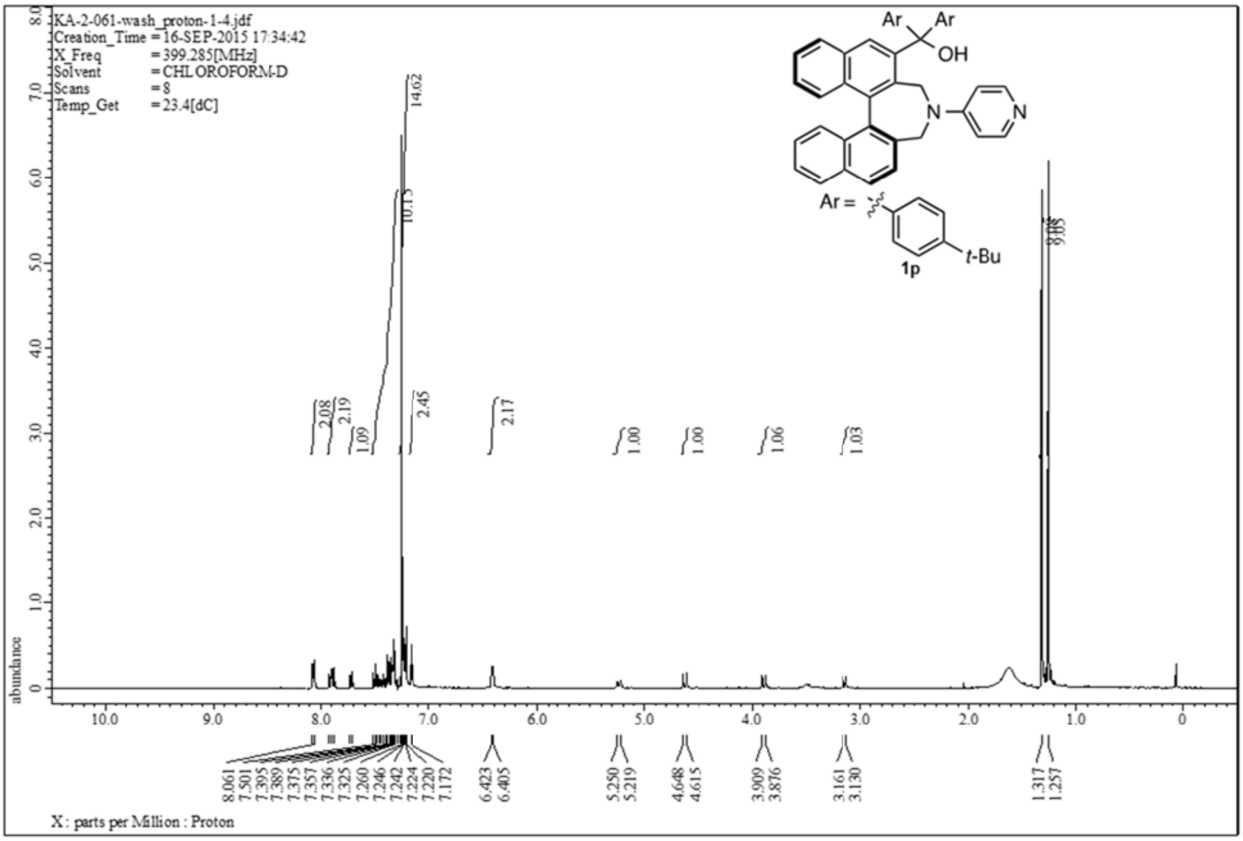

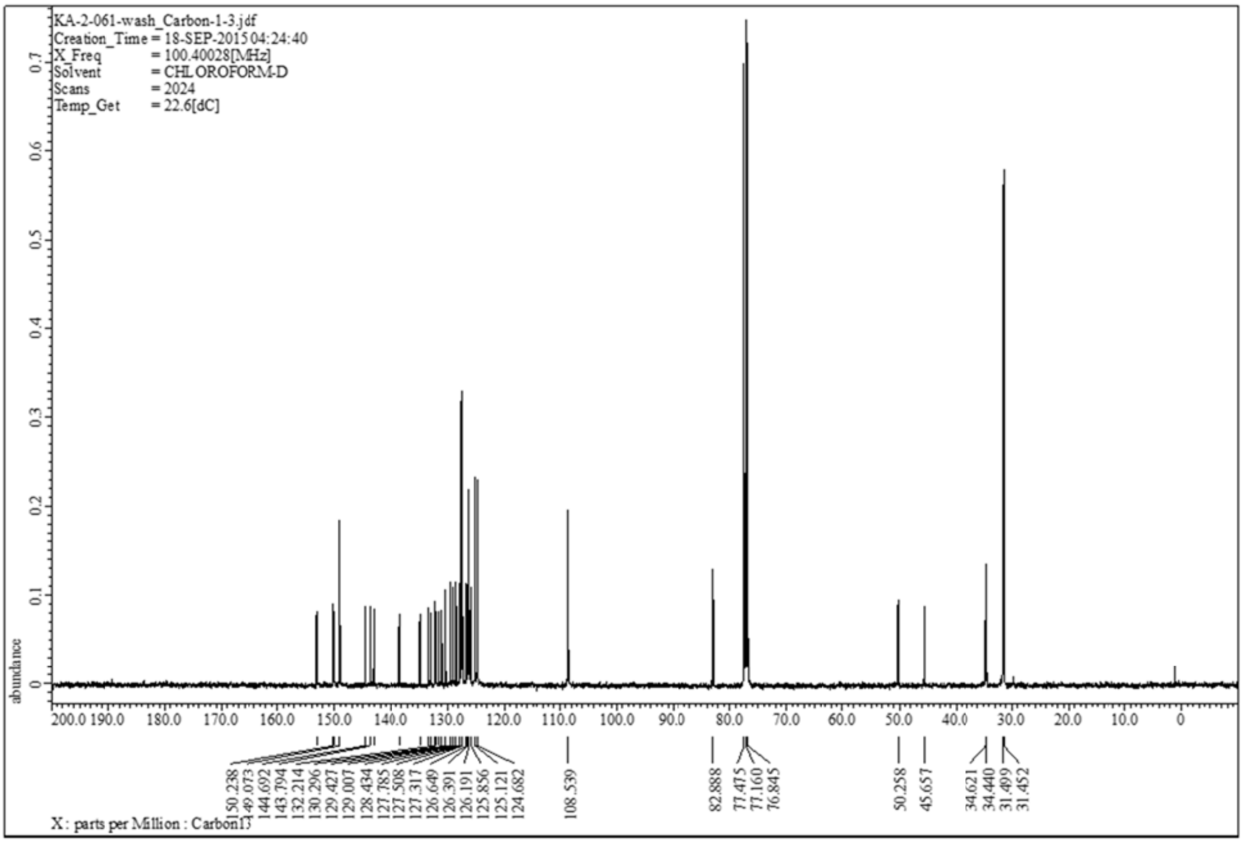

Supplementary Figure 26. ^{1}H and ^{13}C-NMR spectra for **1p**

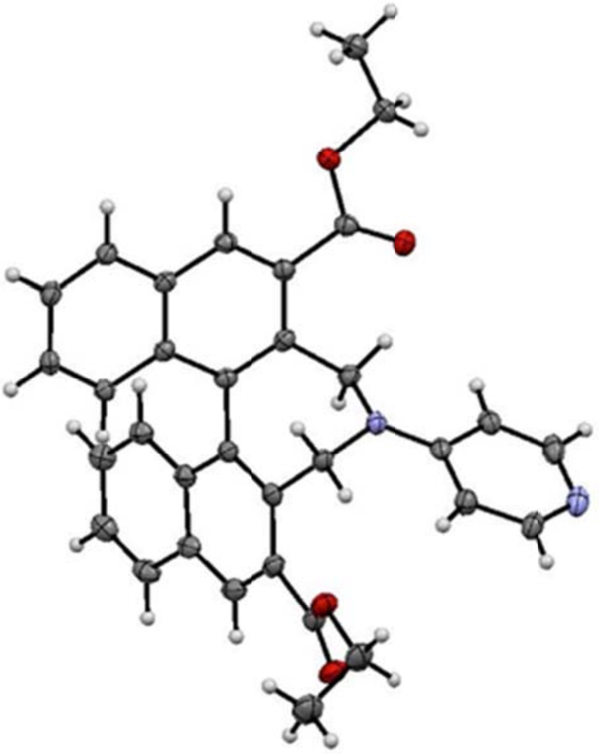

Supplementary Figure 27. X-ray structure of catalyst **1e**

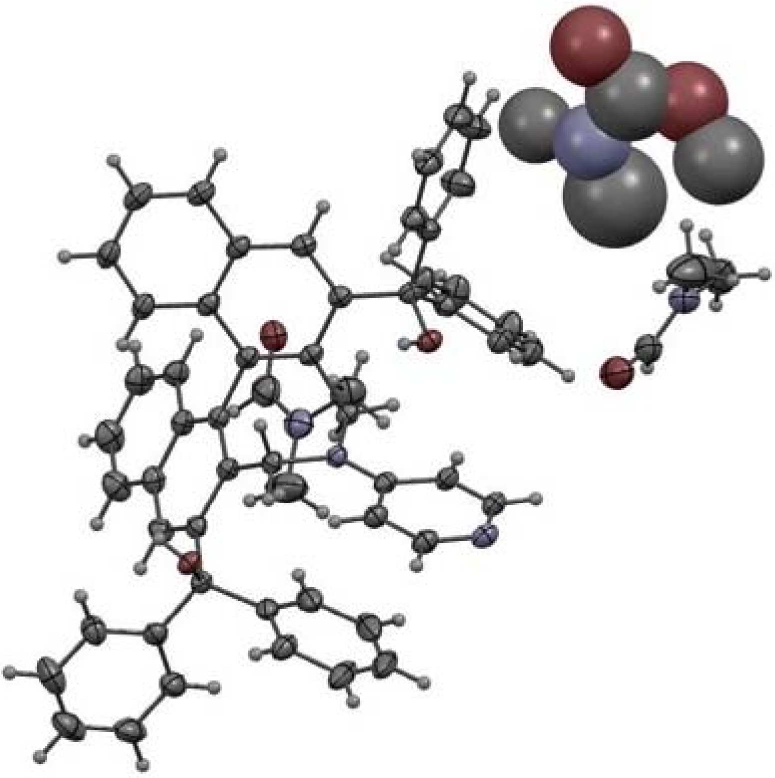

Supplementary Figure 28. X-ray structure of catalyst **1g**

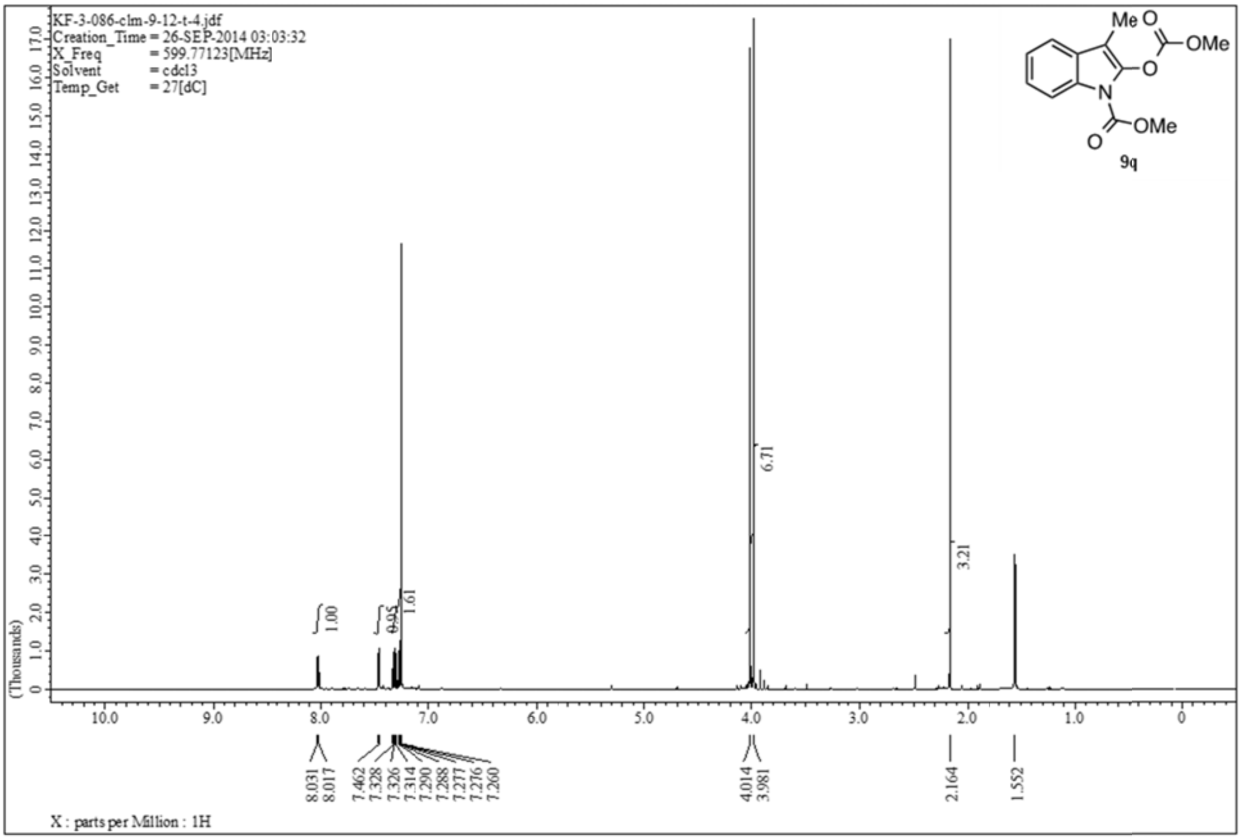

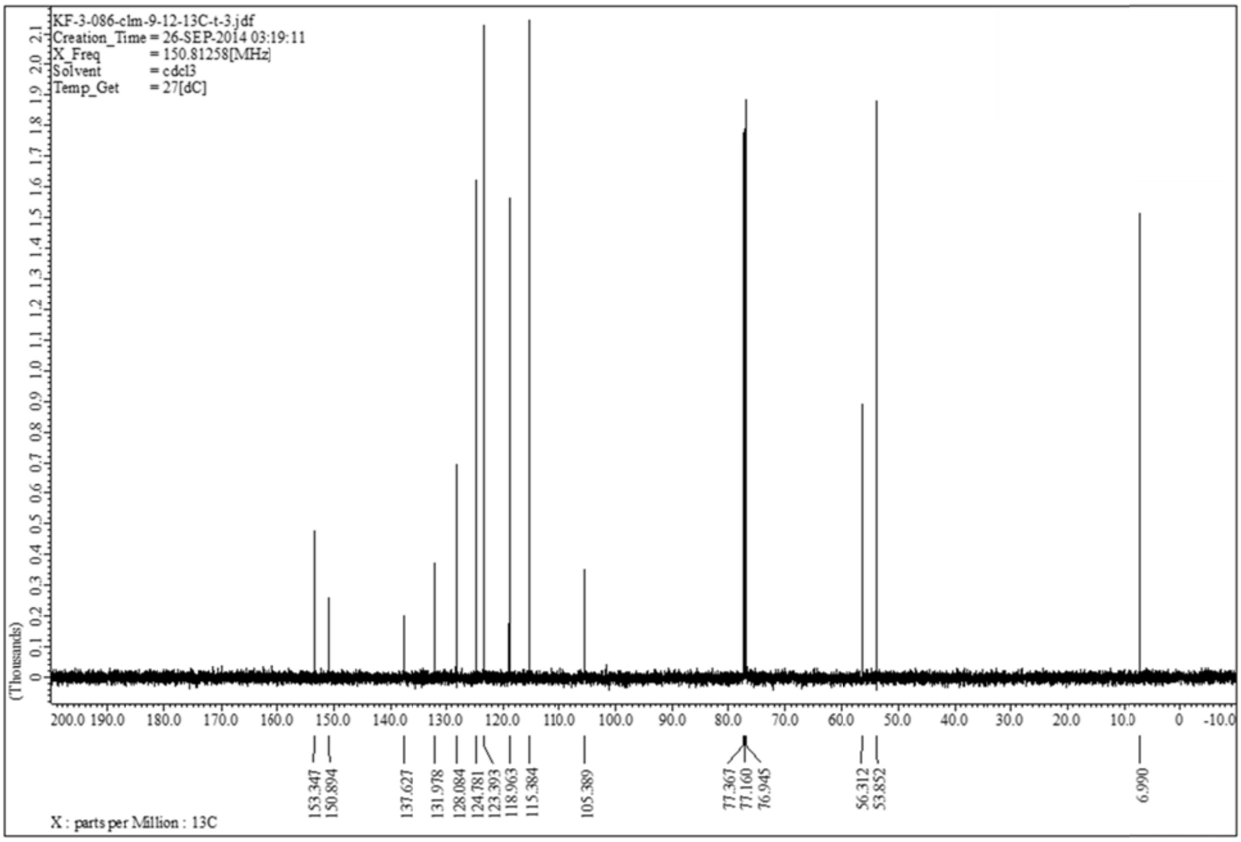

Supplementary Figure 29. ^{1}H and ^{13}C-NMR spectra for substrate **9q**

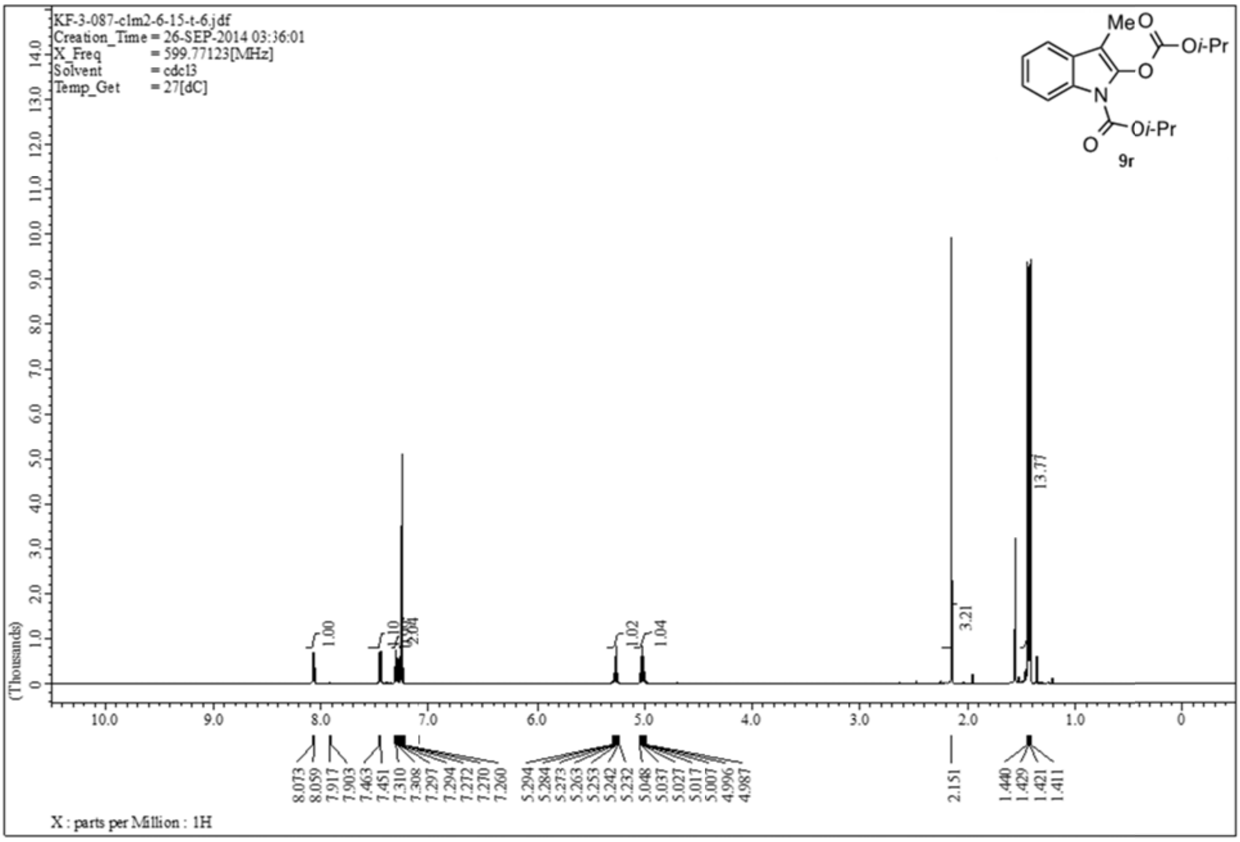

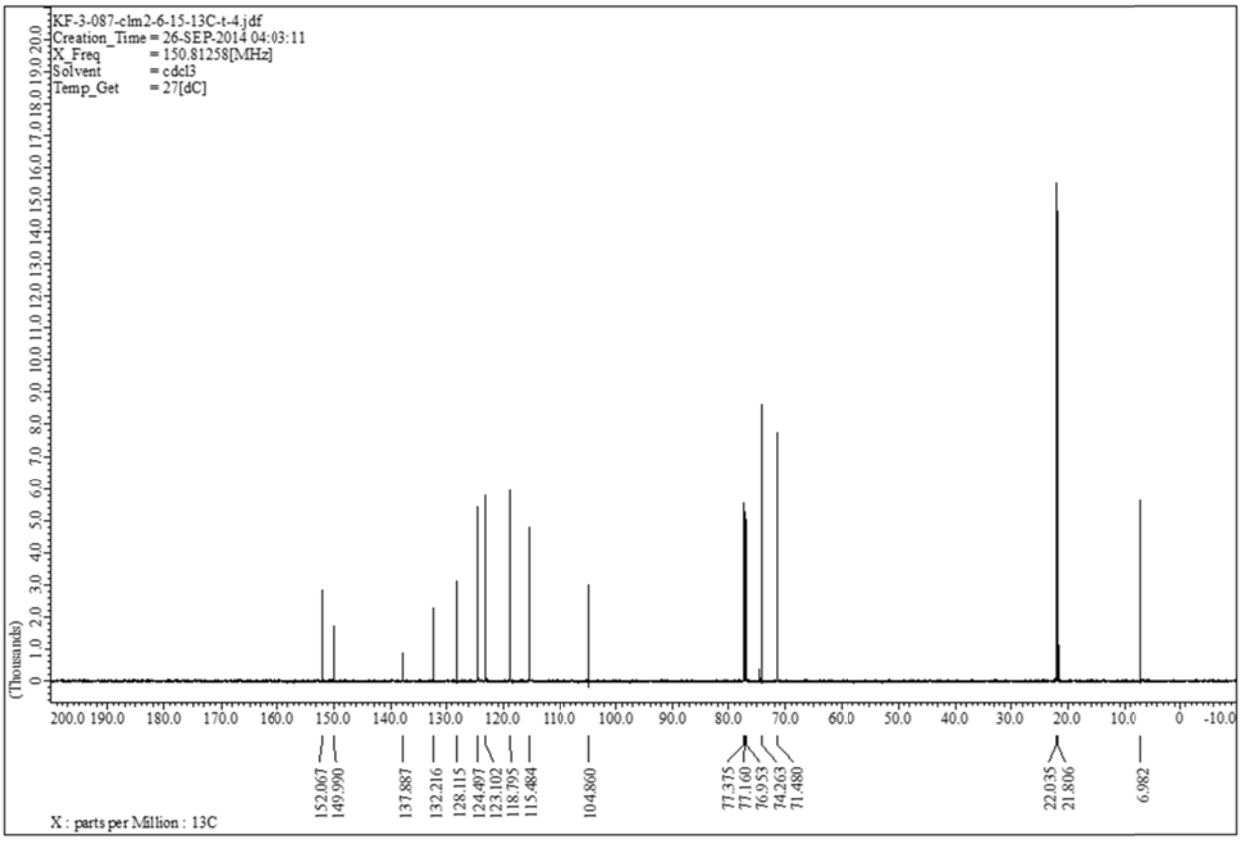

Supplementary Figure 30. ^{1}H and ^{13}C-NMR spectra for substrate **9r**

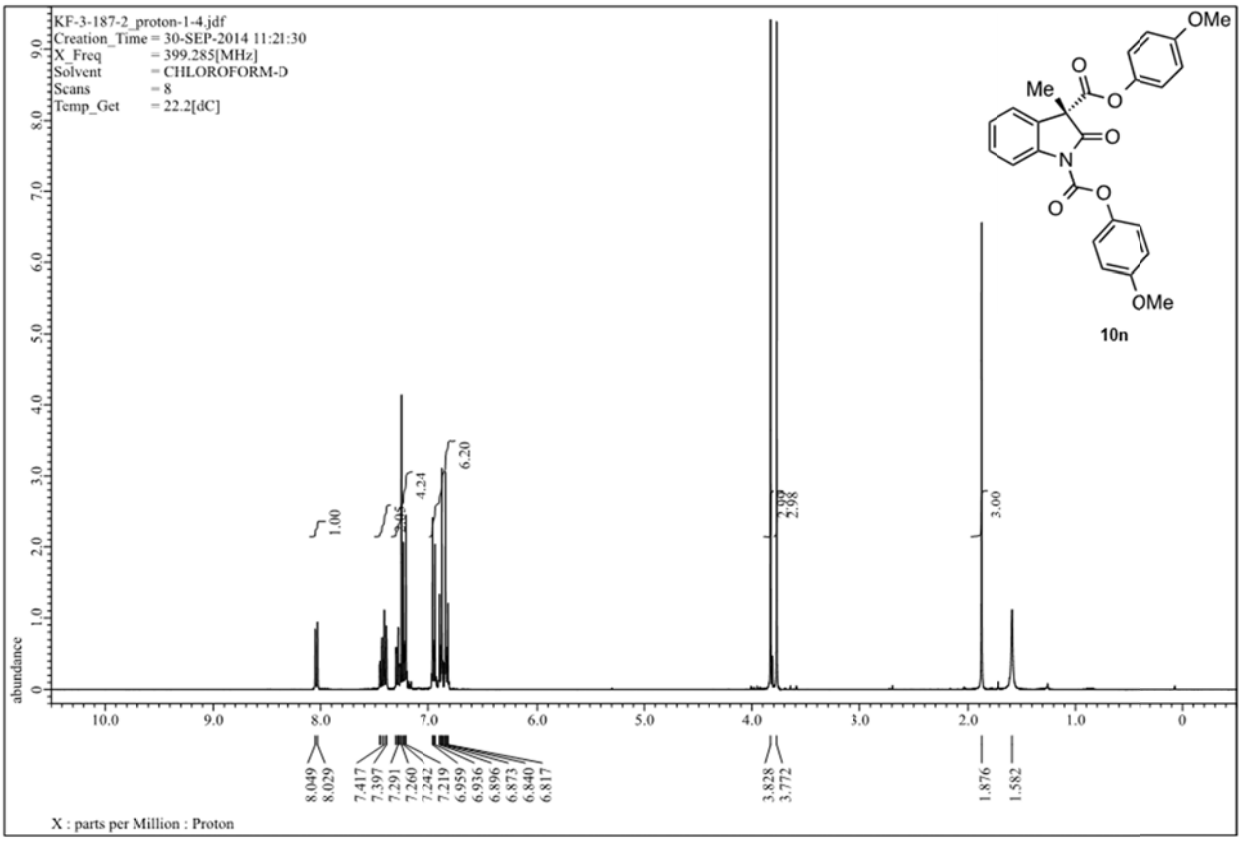

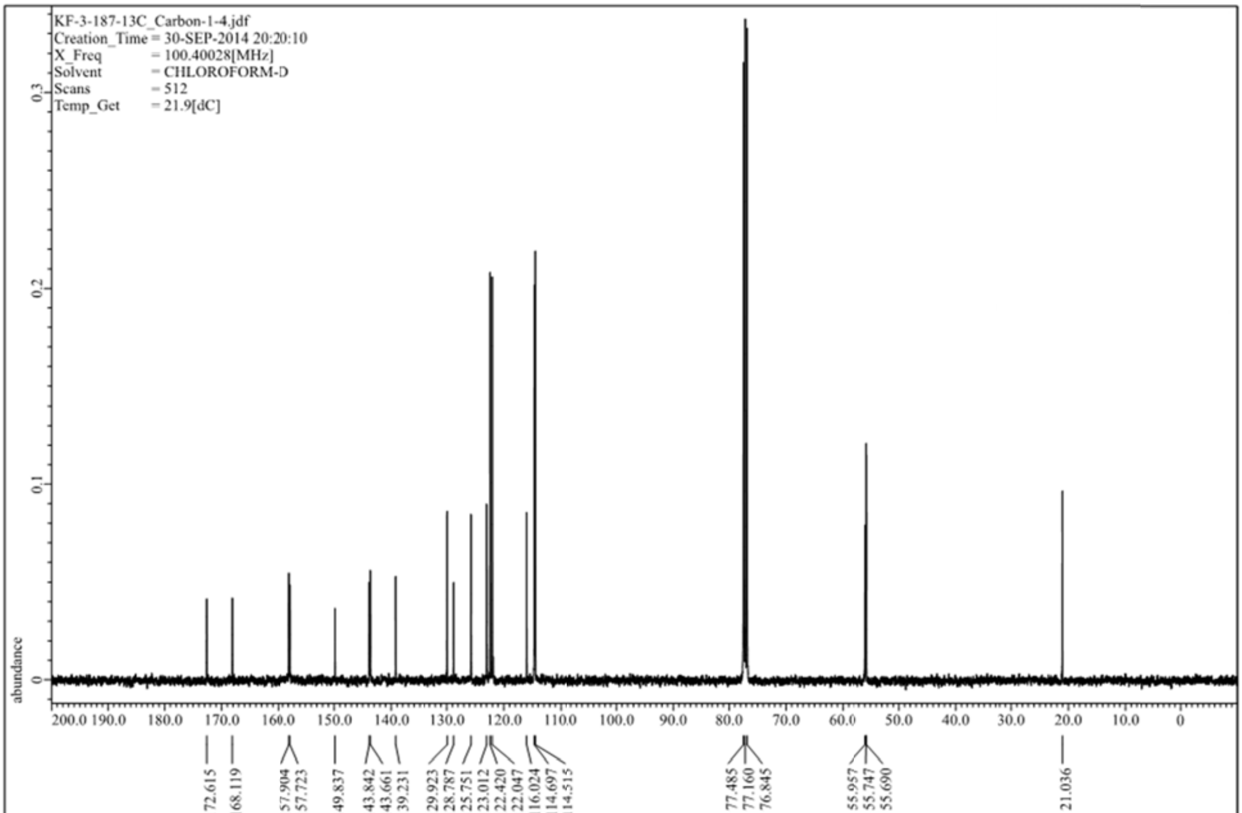

Supplementary Figure 31. ^{1}H and ^{13}C-NMR spectra for product **10n**

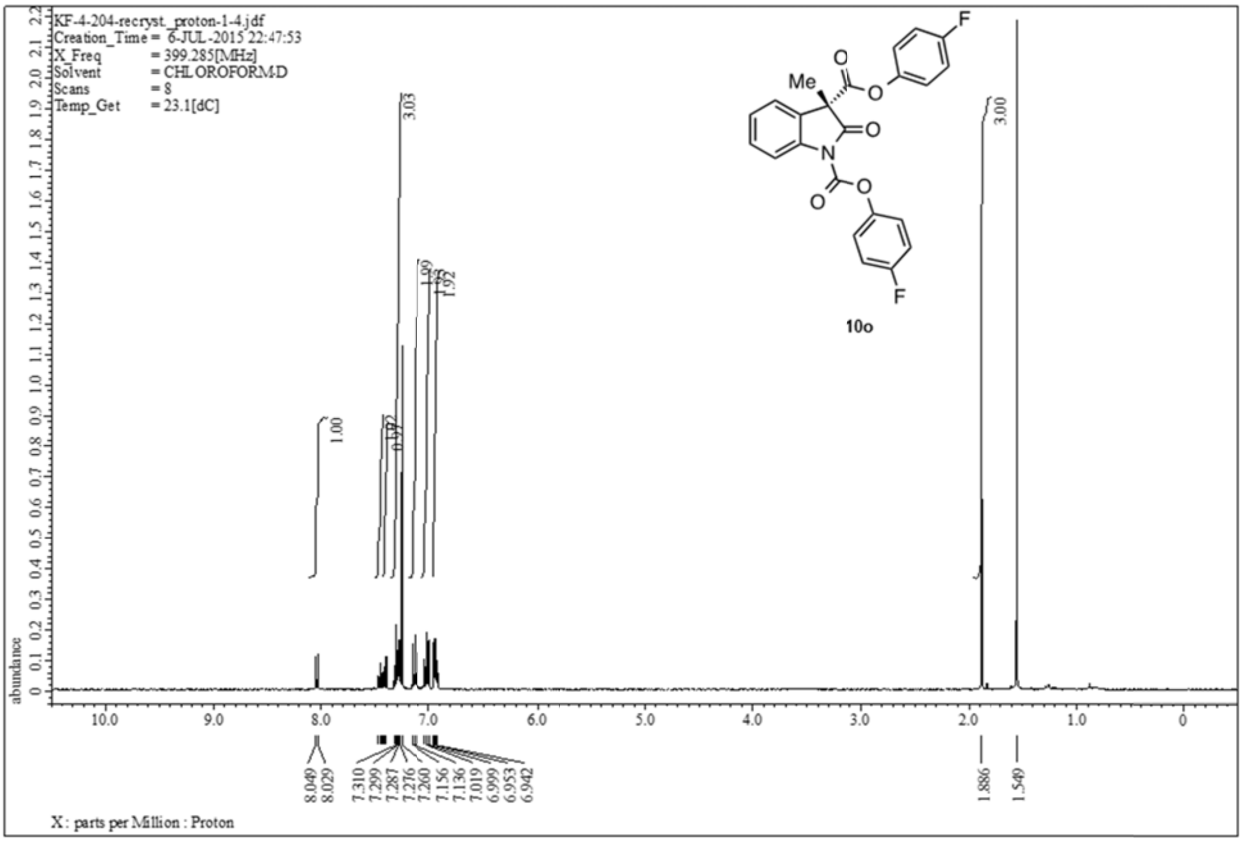

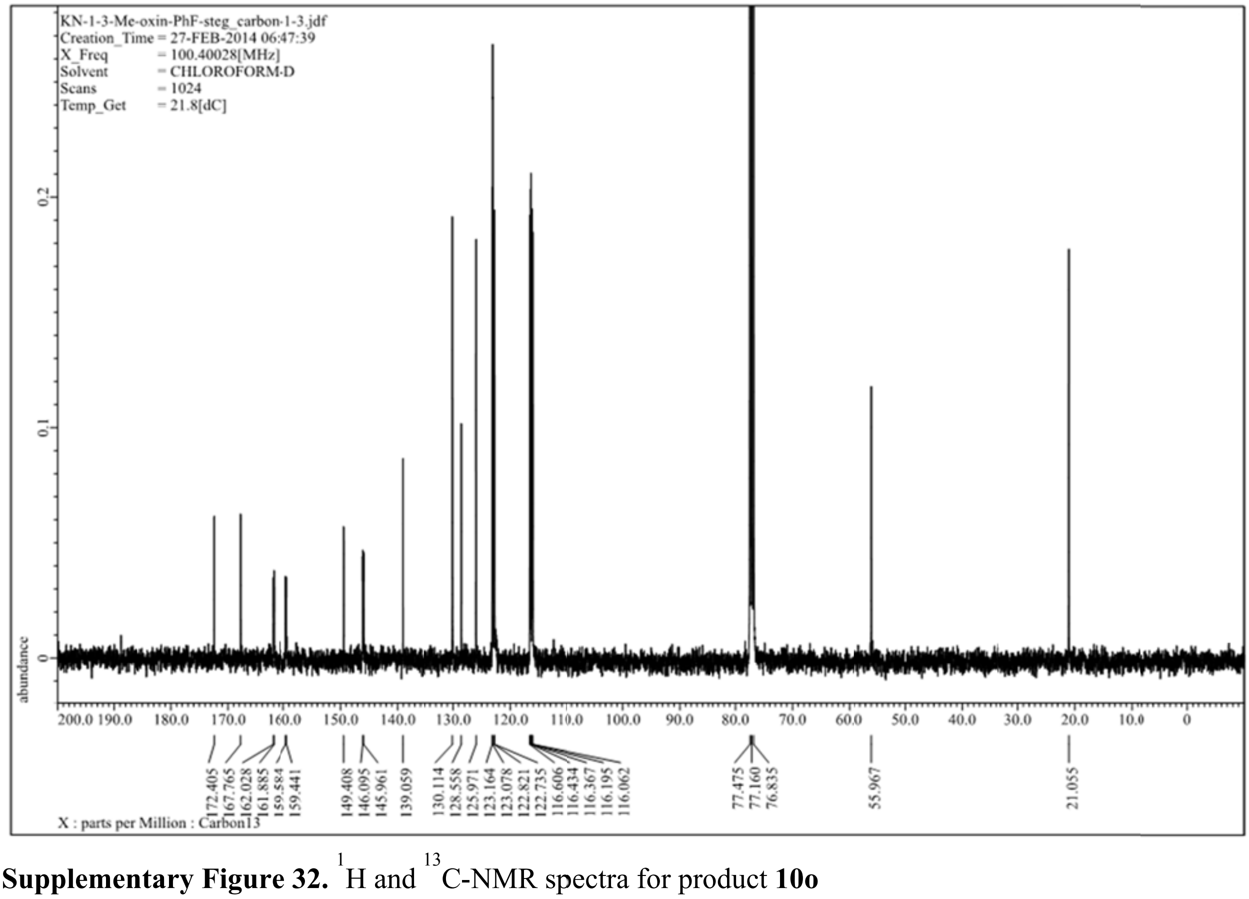

Supplementary Figure 32. ^{1}H and ^{13}C-NMR spectra for product **10o**

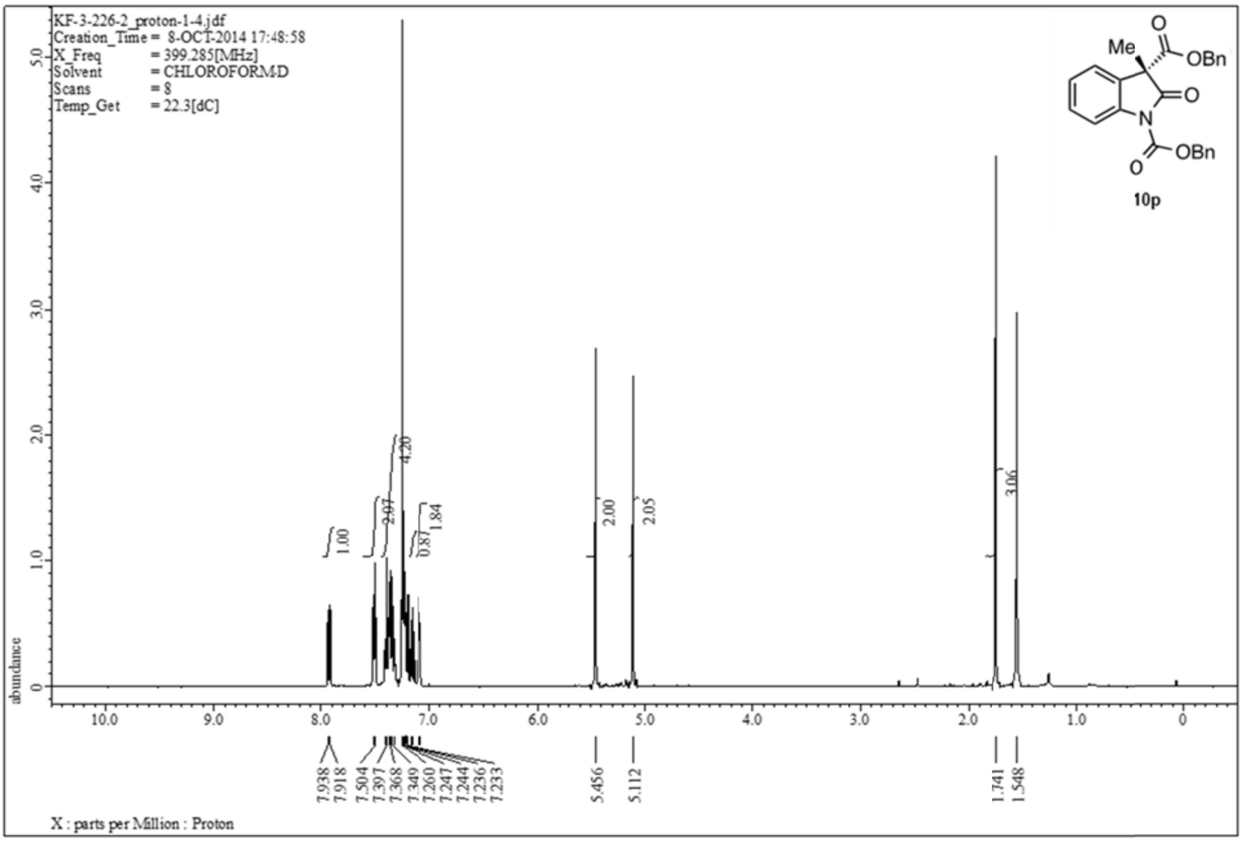

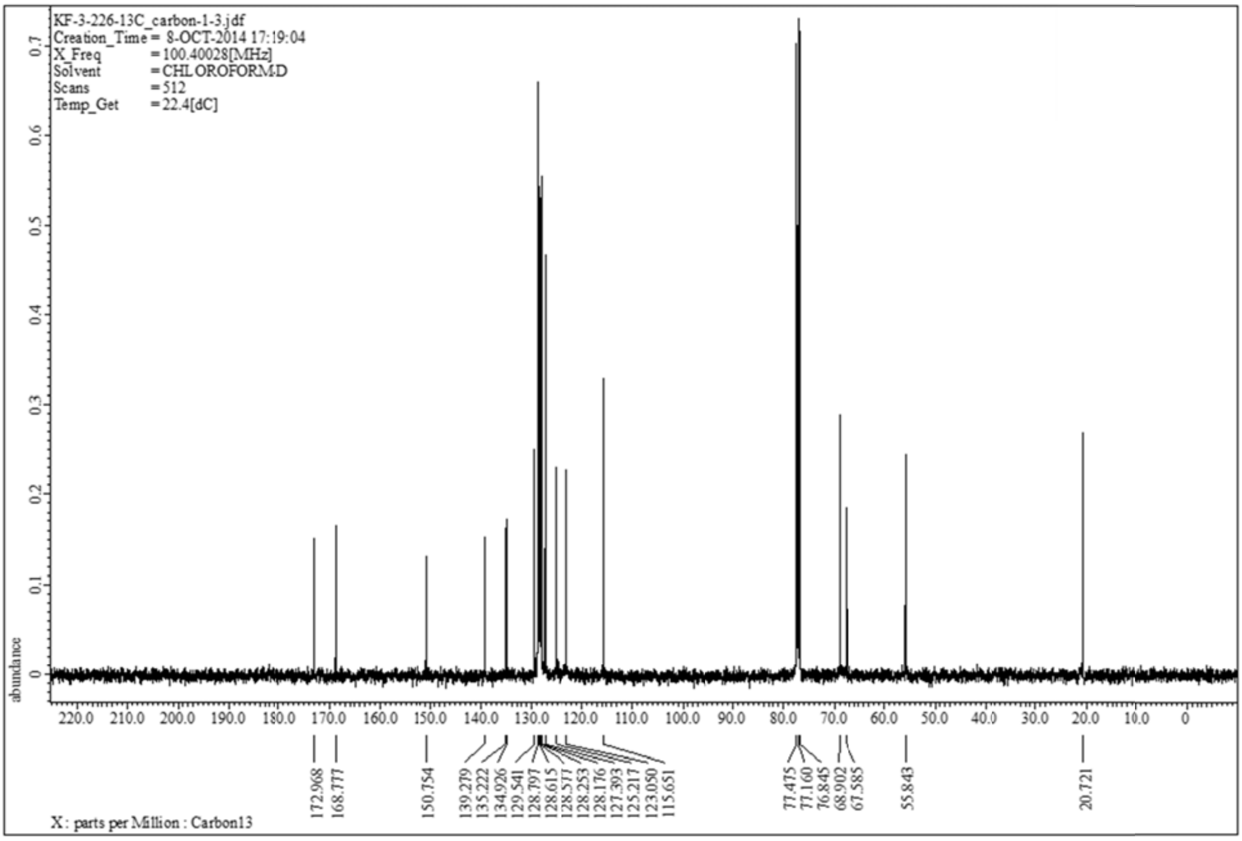

Supplementary Figure 33. ^{1}H and ^{13}C-NMR spectra for product **10p**

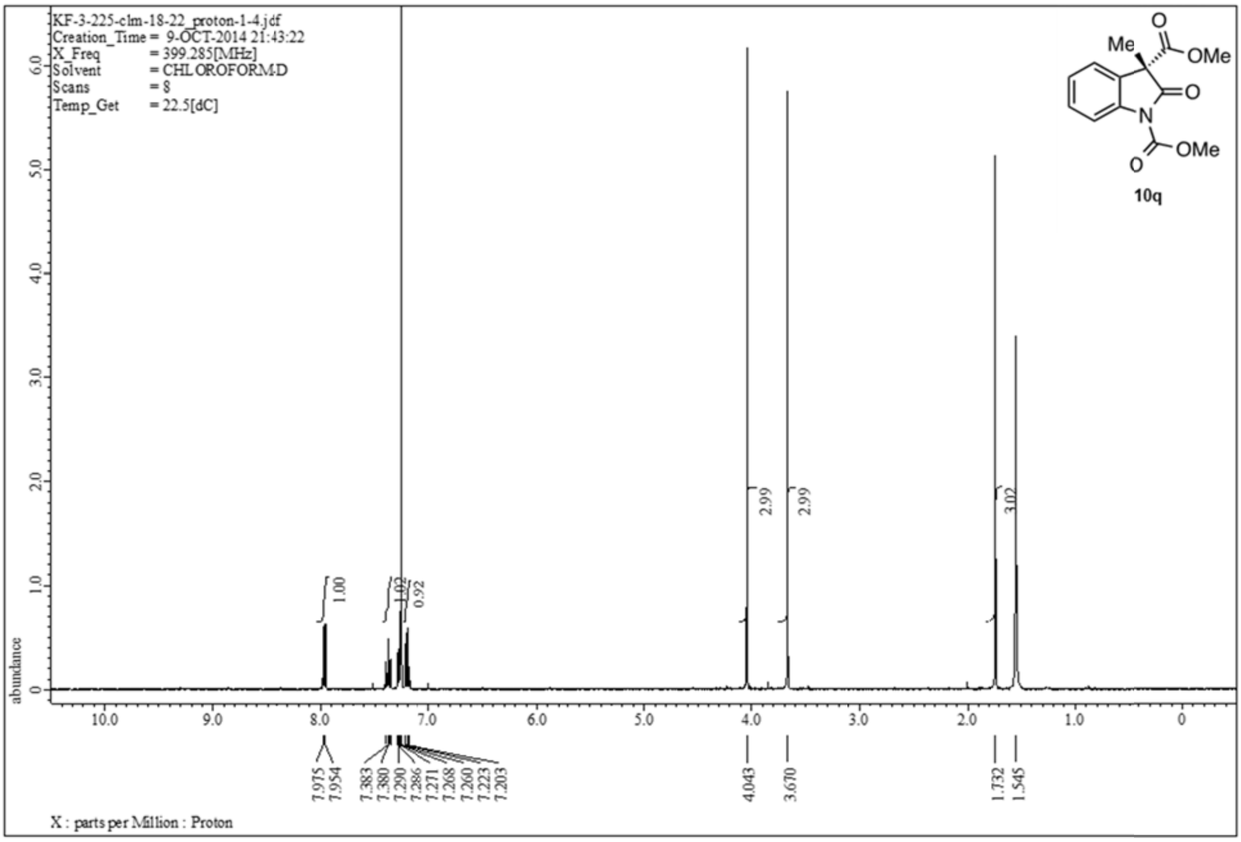

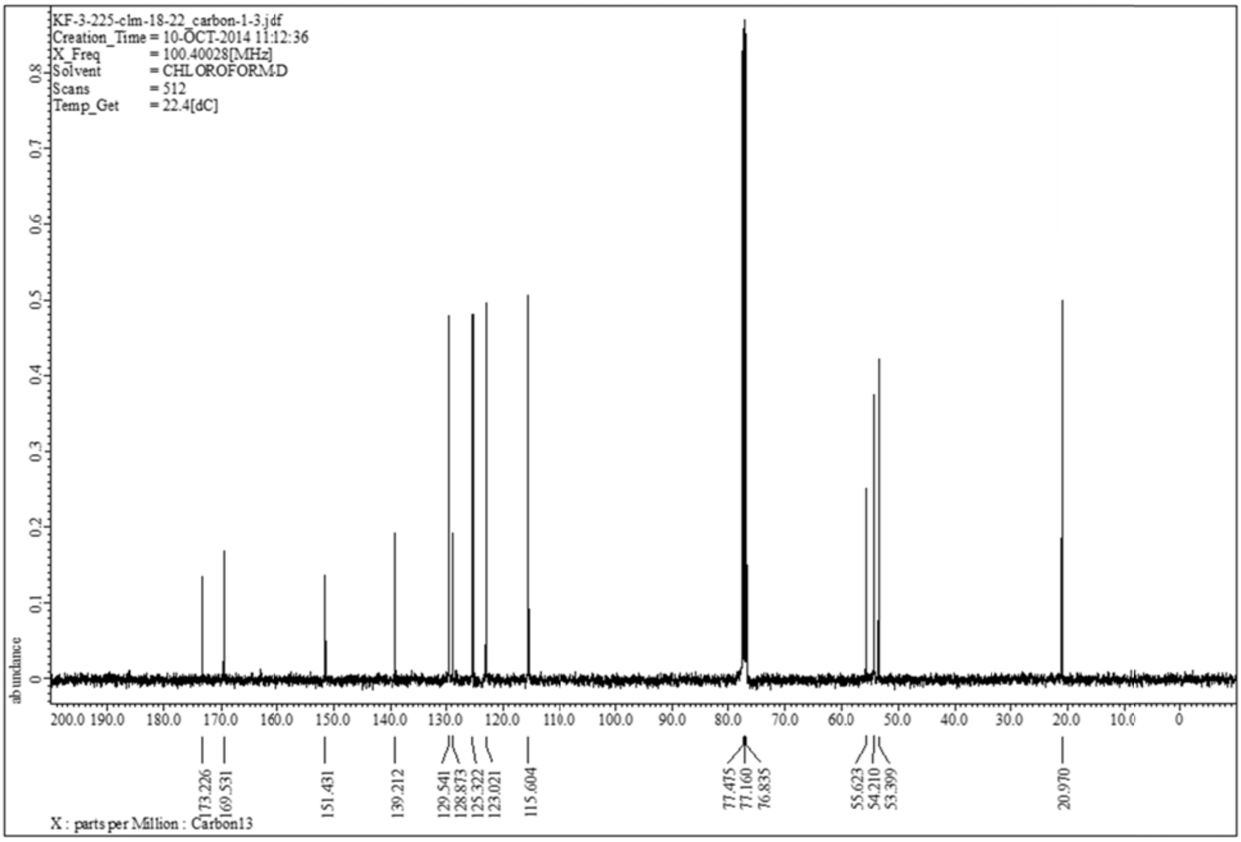

Supplementary Figure 34. ^{1}H and ^{13}C-NMR spectra for product **10q**

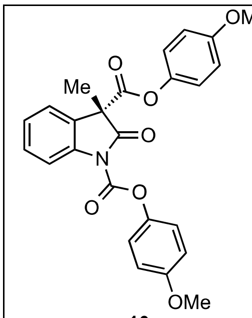

10n

Racemic sample of **10n**				(*S*)-**10n**			

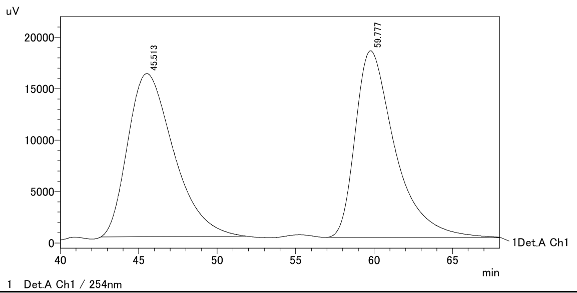

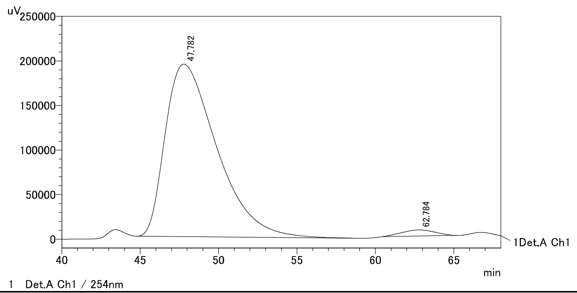

**Peak #**	**Ret. Time**	**Area**	**Area %**	**Peak #**	**Ret. Time**	**Area**	**Area %**
1	45.513	3232619	50.210	1	47.782	44320569	97.881
2	59.777	3205618	49.790	2	62.784	959339	2.119

Supplementary Figure 35. HPLC spectra for product **10n** of Steglich rearrangement with catalyst **1j** (see Supplementary Table 3)

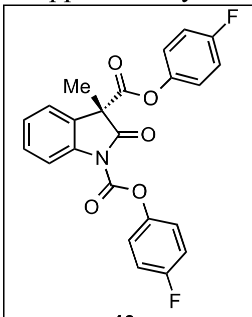

10o

Racemic sample of **10o**				(*S*)-**10o**			

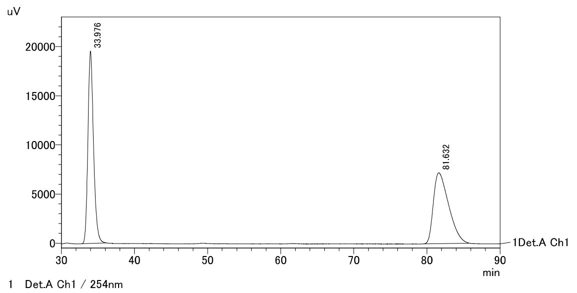

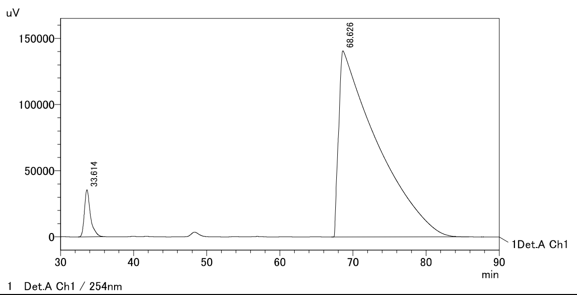

**Peak #**	**Ret. Time**	**Area**	**Area %**	**Peak #**	**Ret. Time**	**Area**	**Area %**
1	33.976	1041862	49.883	1	33.614	2045765	3.826
2	81.632	1046766	50.117	2	68.626	51425941	96.174

Supplementary Figure 36. HPLC spectra for product **10o** of Steglich rearrangement with catalyst **1j** (see Supplementary Table 3)

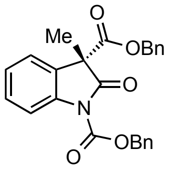

10p

Racemic sample of **10p**	(*S*)-**10p**
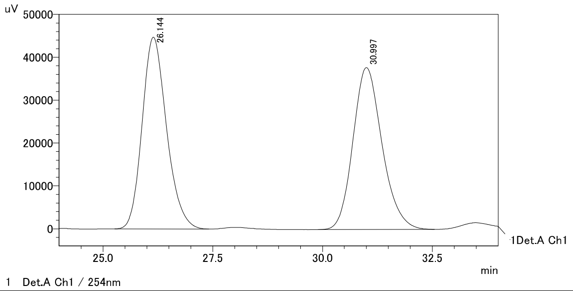	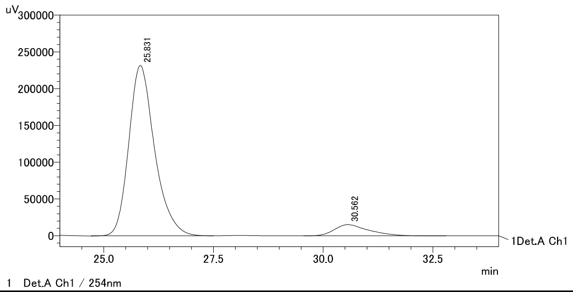

Peak #	Ret. Time	Area	Area %
1	26.144	1729627	49.841
2	30.997	1740677	50.159

Peak #	Ret. Time	Area	Area %
1	25.831	9461752	92.208
2	30.562	799572	7.792

Supplementary Figure 37. HPLC spectra for product **10p** of Steglich rearrangement with catalyst **1j** (see Supplementary Table 3)

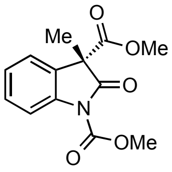

10q

Racemic sample of **10q**	(*S*)-**10q**
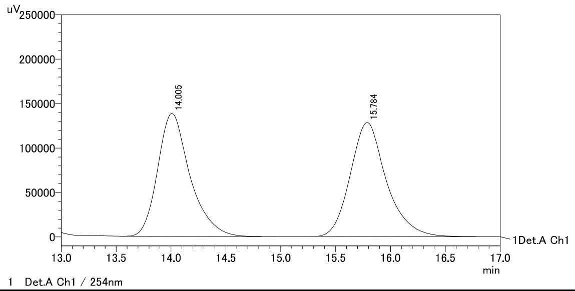	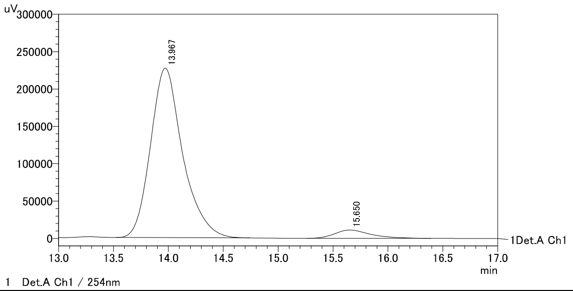

Peak #	Ret. Time	Area	Area %
1	14.005	2832339	49.212
2	15.784	2923020	50.788

Peak #	Ret. Time	Area	Area %
1	13.967	4624433	94.900
2	15.650	248504	5.100

Supplementary Figure 38. HPLC spectra for product **10q** of Steglich rearrangement with catalyst **1j** (see Supplementary Table 3)

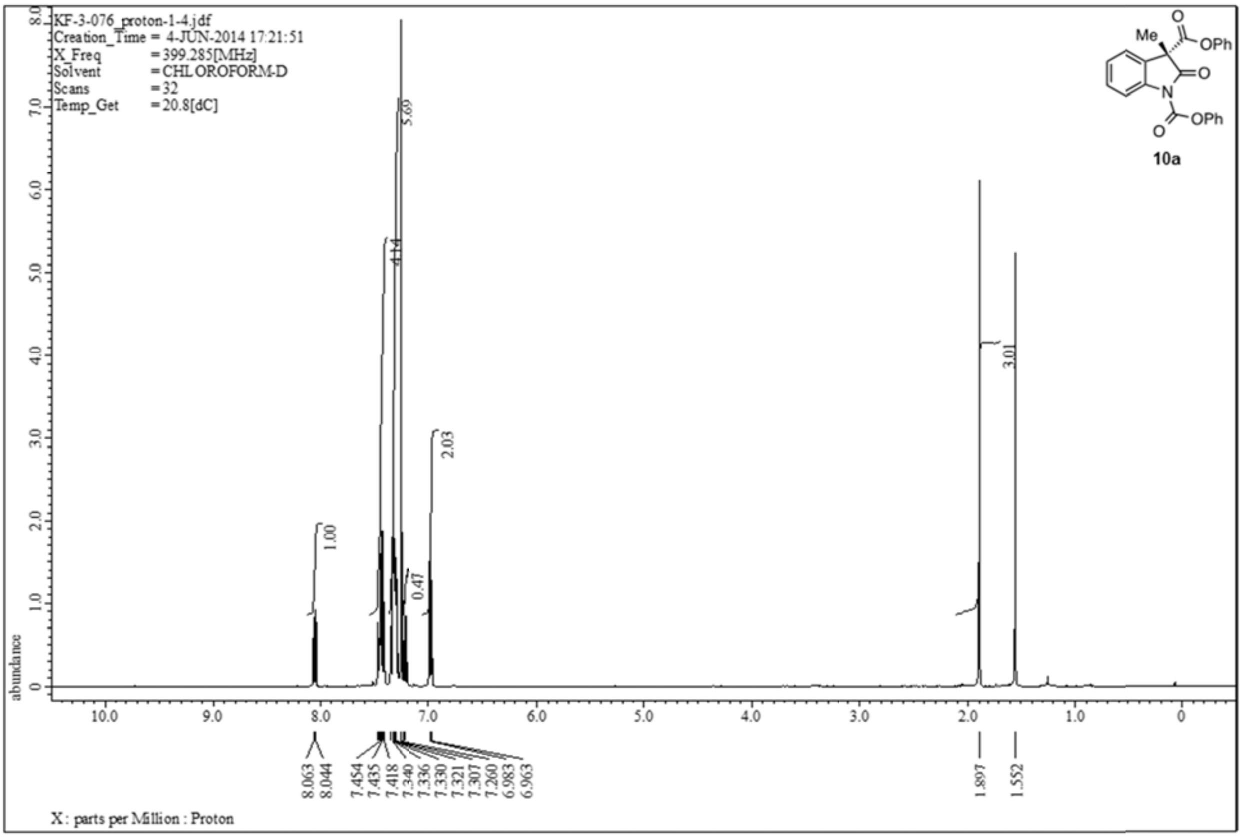

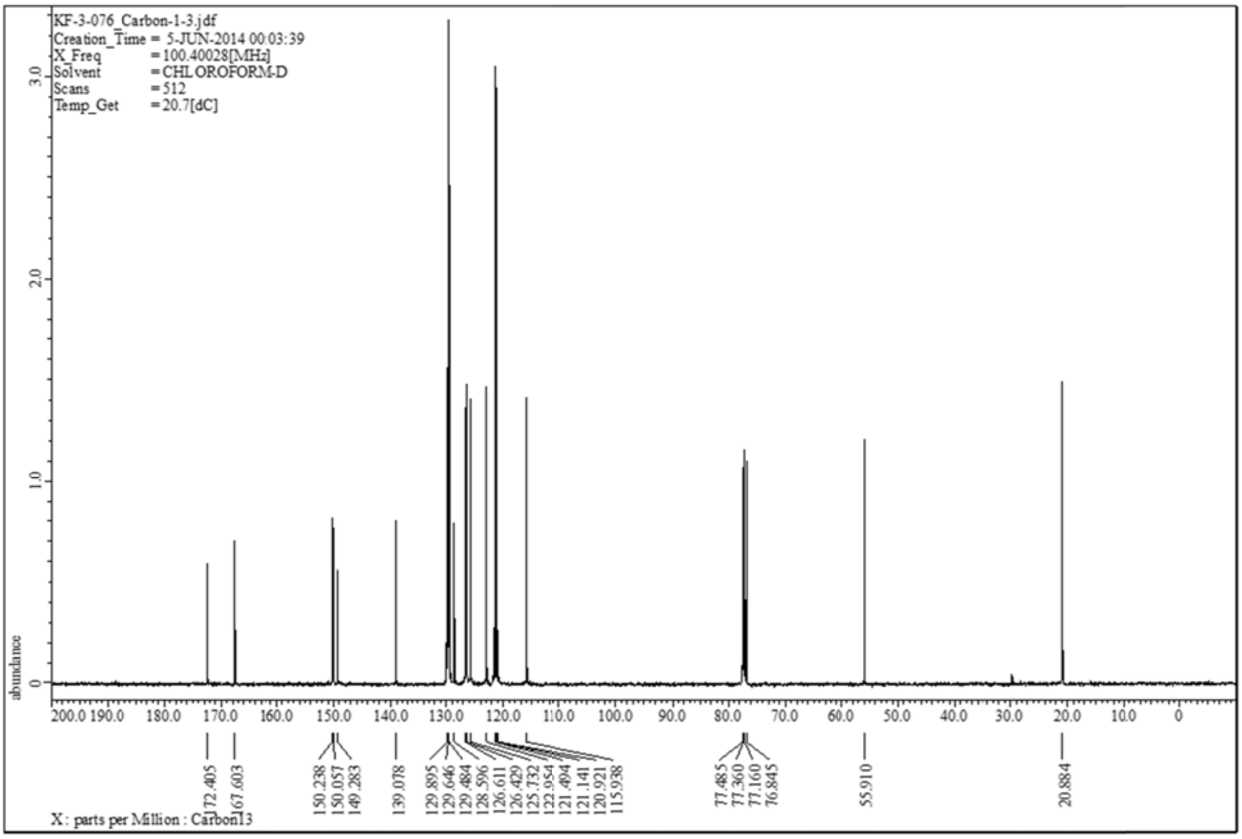

Supplementary Figure 39. ^{1}H and ^{13}C-NMR spectra for product **10a**

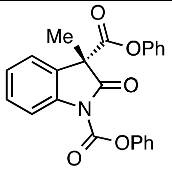

10a

Racemic sample of **10a**	(*S*)-**10a**
A Ch1	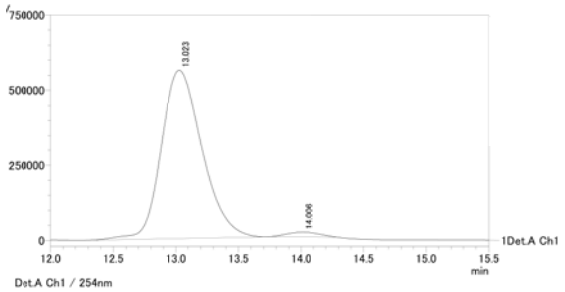

Peak #	Ret. Time	Area	Area %	Peak #	Ret. Time	Area	Area %
1	13.513	102652	50.170	1	13.023	35585727	97.805
2	14.575	90628	49.830	2	14.006	286167	2.195

Supplementary Figure 40. HPLC spectra for product **10a** of Steglich rearrangement on multi-gram scale (see Figure 4b and Supplementary Methods).

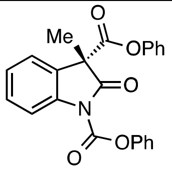

10a

Racemic sample of **10a**	(*S*)-**10a**
A Ch1	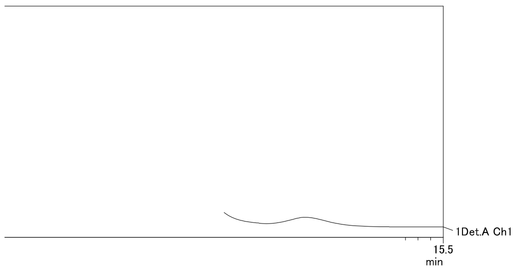

Peak #	Ret. Time	Area	Area %	Peak #	Ret. Time	Area	Area %
1	13.513	102652	50.170	1	13.321	15845757	97.712
2	14.575	90628	49.830	2	14.399	370968	2.288

Supplementary Figure 41. HPLC spectra for product **10a** of Steglich rearrangement with recovered catalyst **1j** (see Supplementary Methods).

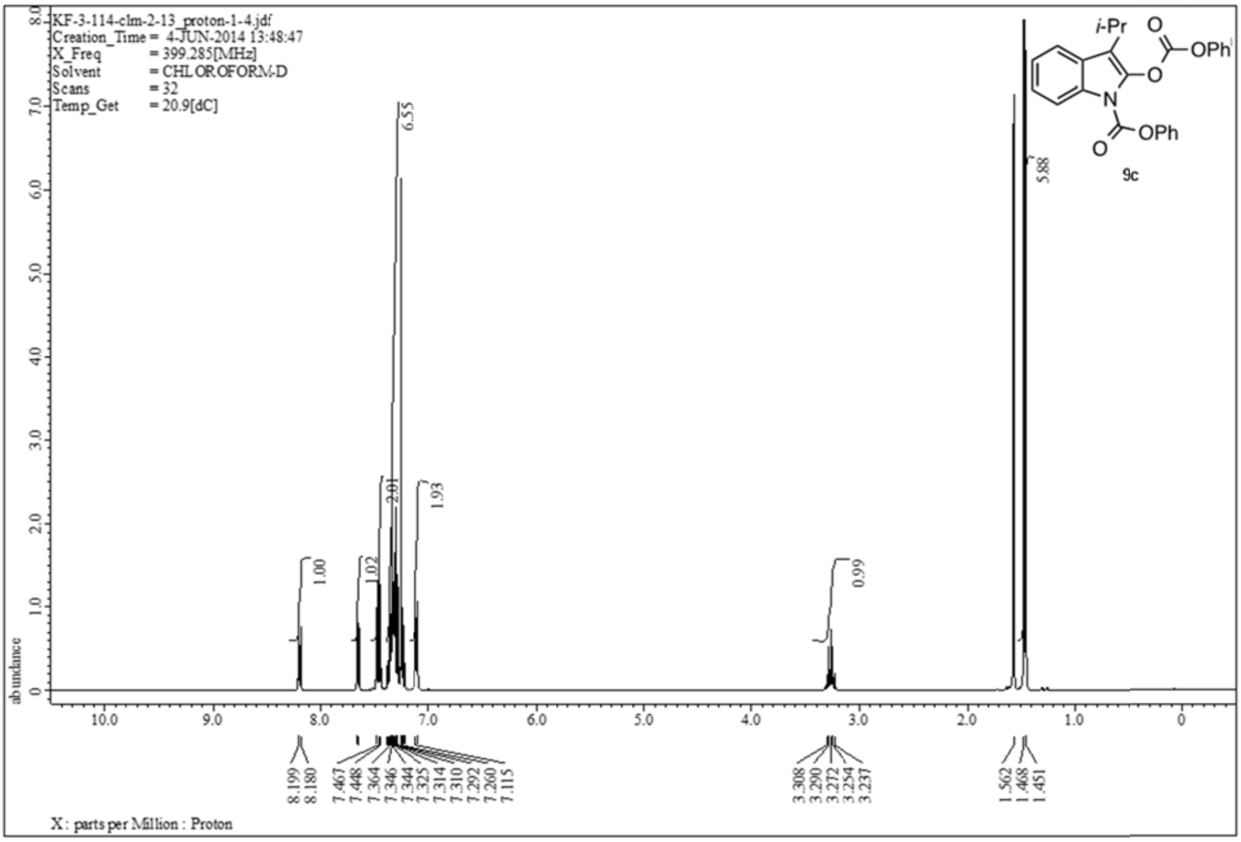

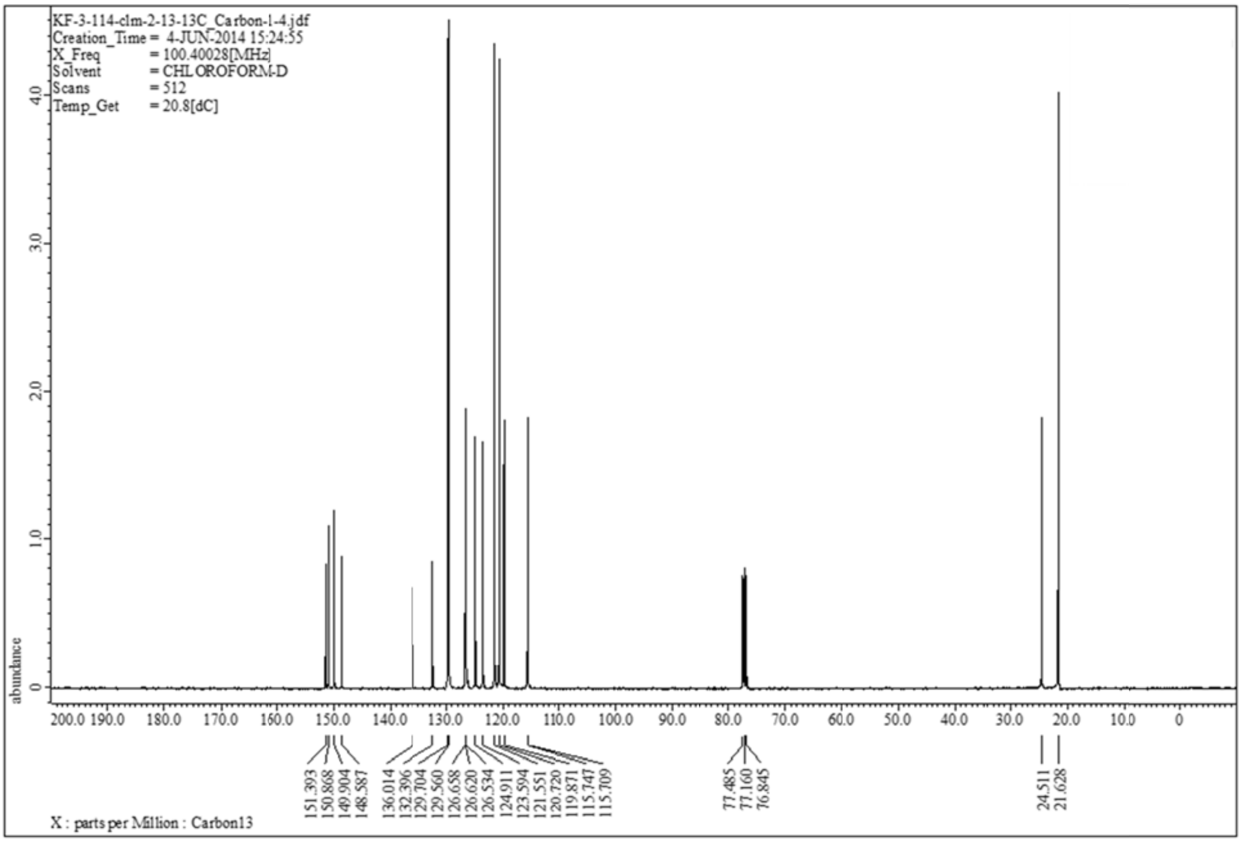

Supplementary Figure 42. ^{1}H and ^{13}C-NMR spectra for substrate **9c**

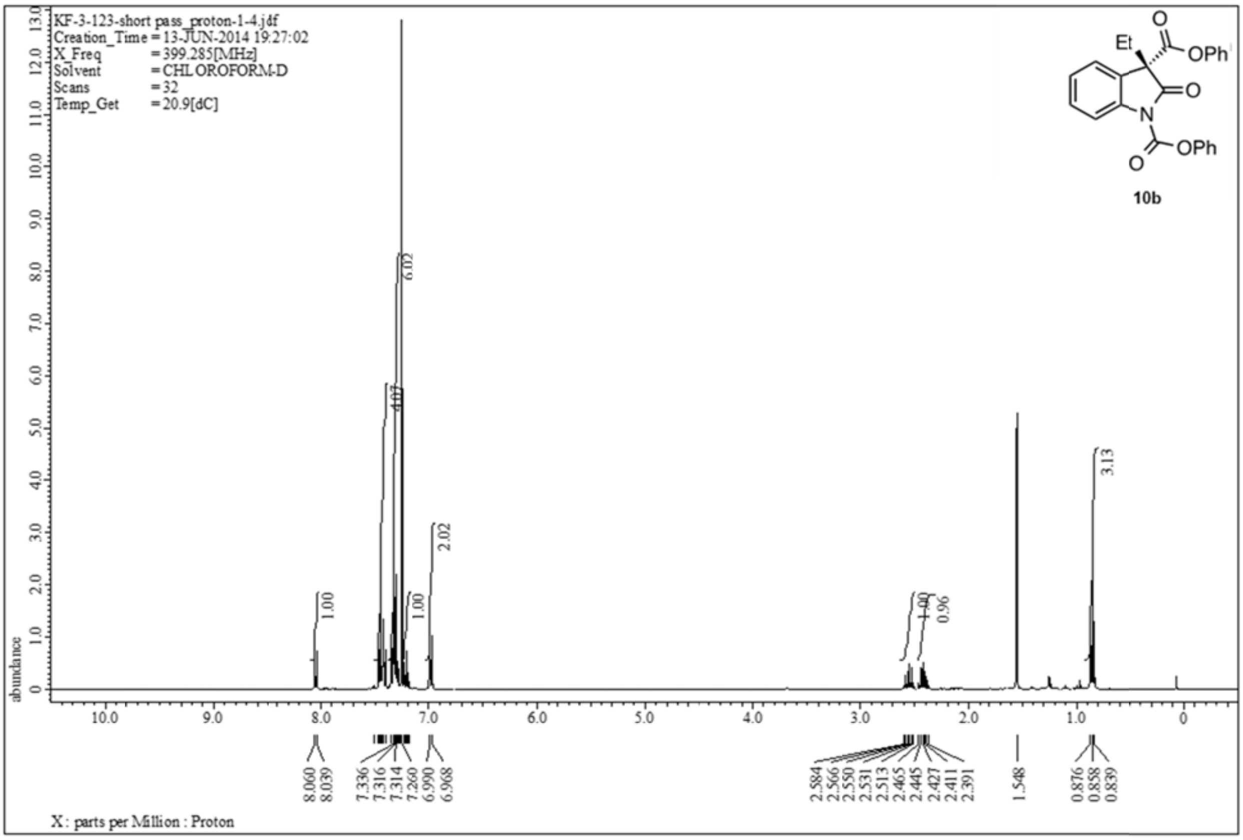

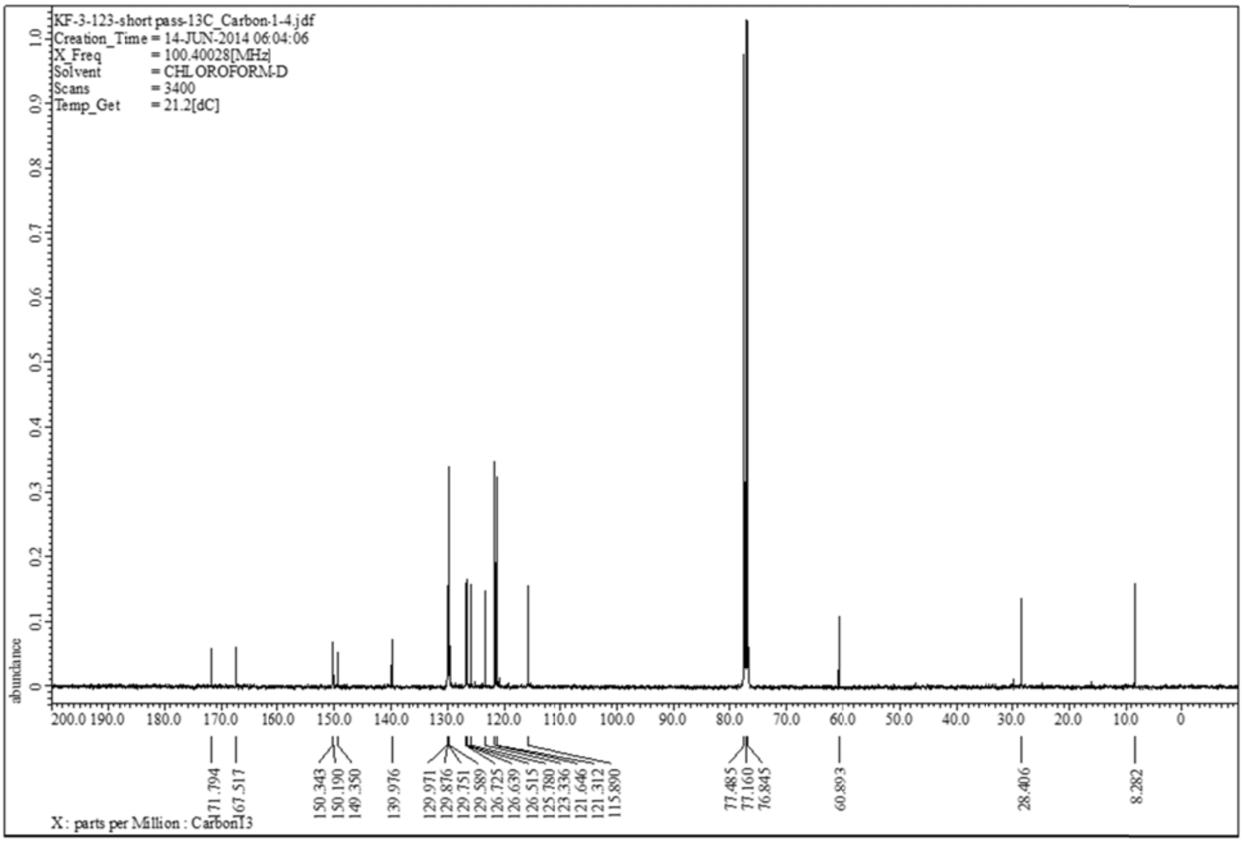

Supplementary Figure 43. ^{1}H and ^{13}C-NMR spectra for product **10b**

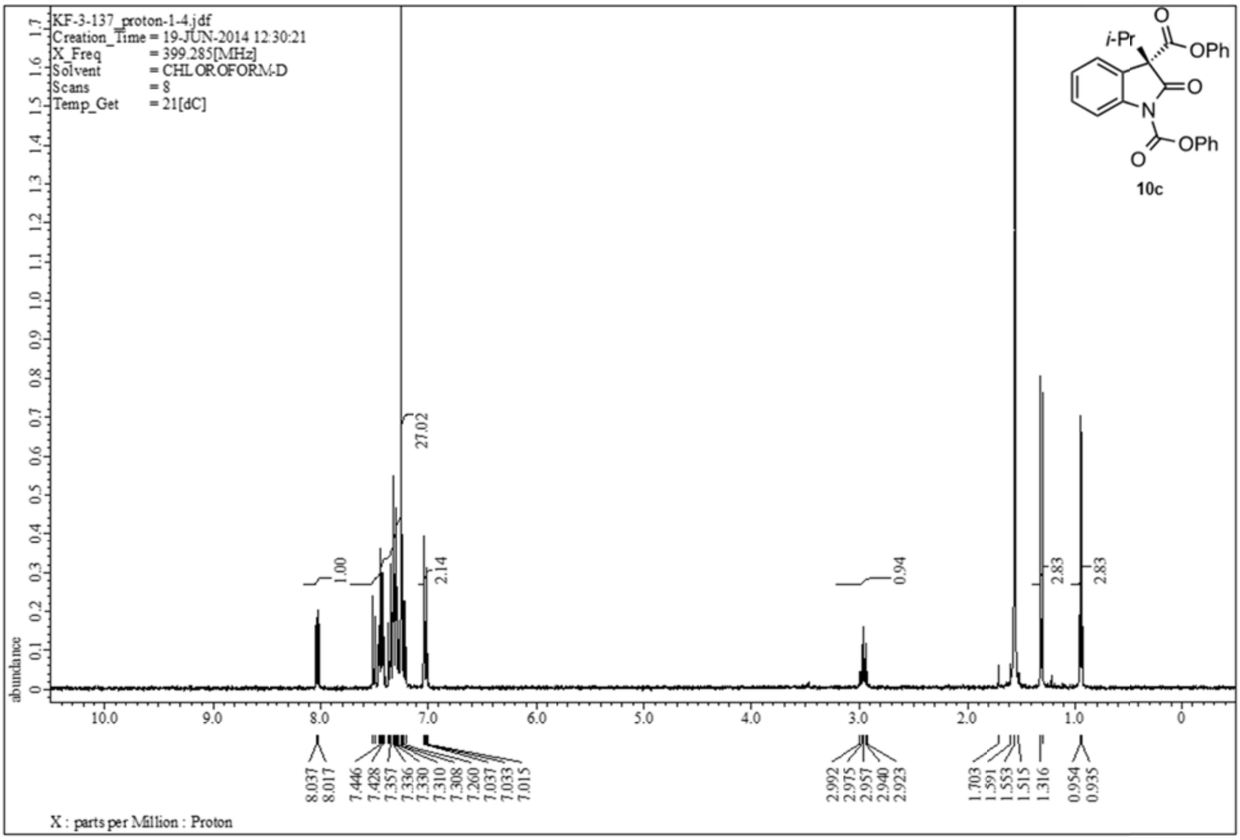

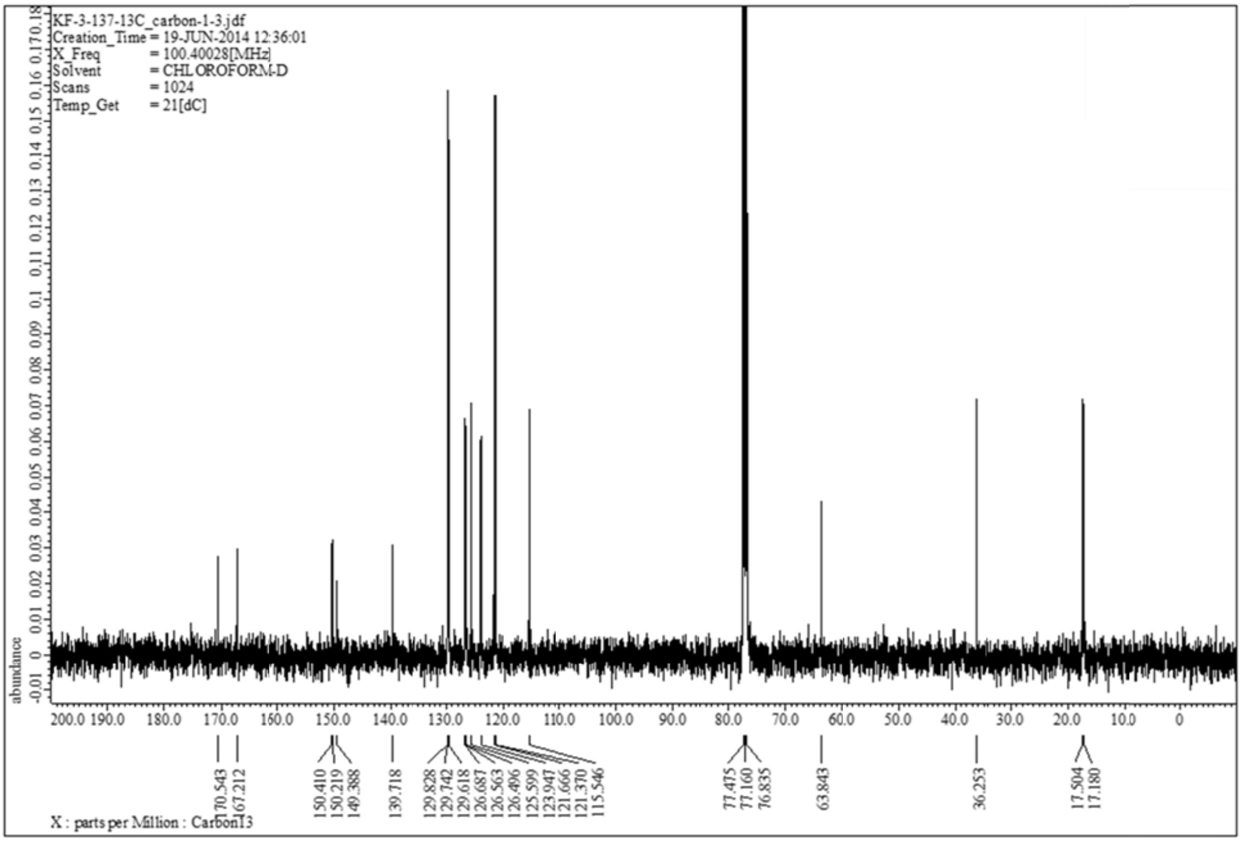

Supplementary Figure 44. ^{1}H and ^{13}C-NMR spectra for product **10c**

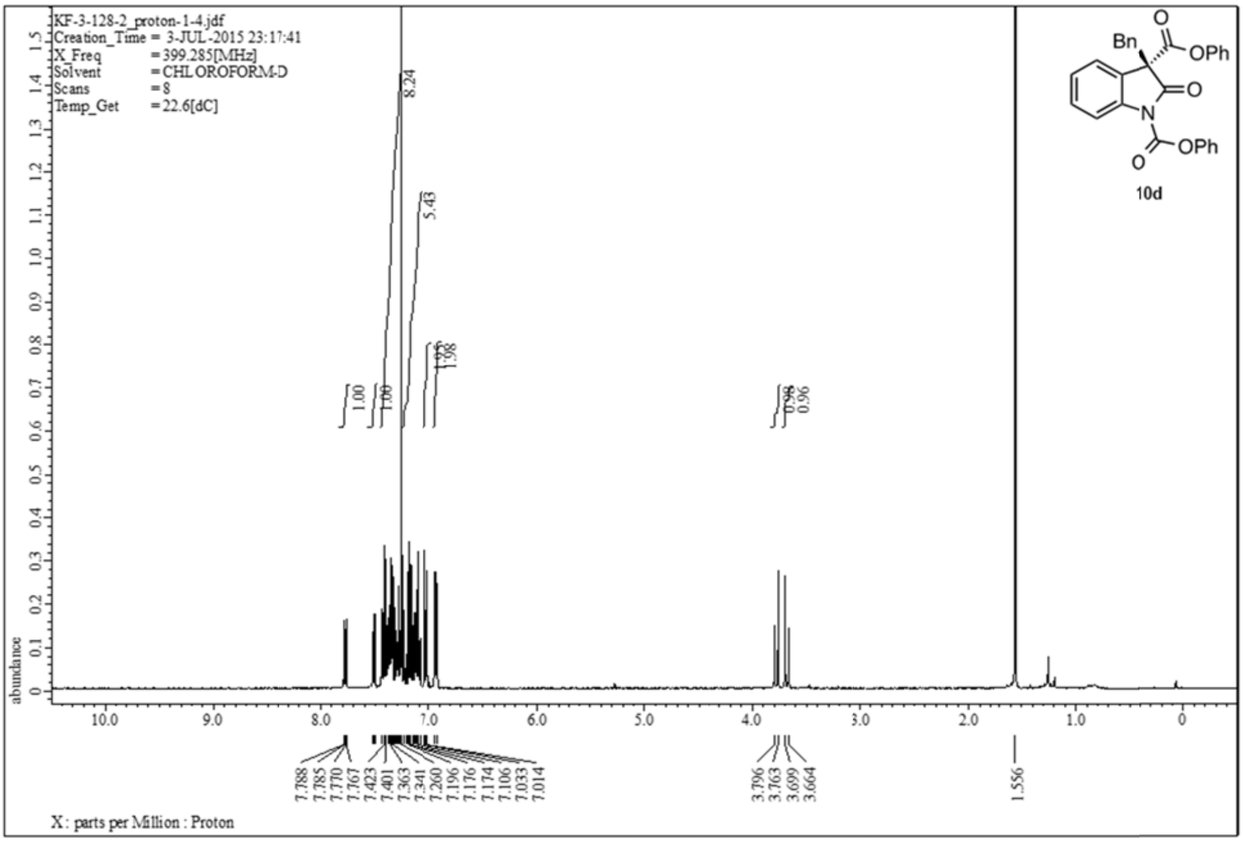

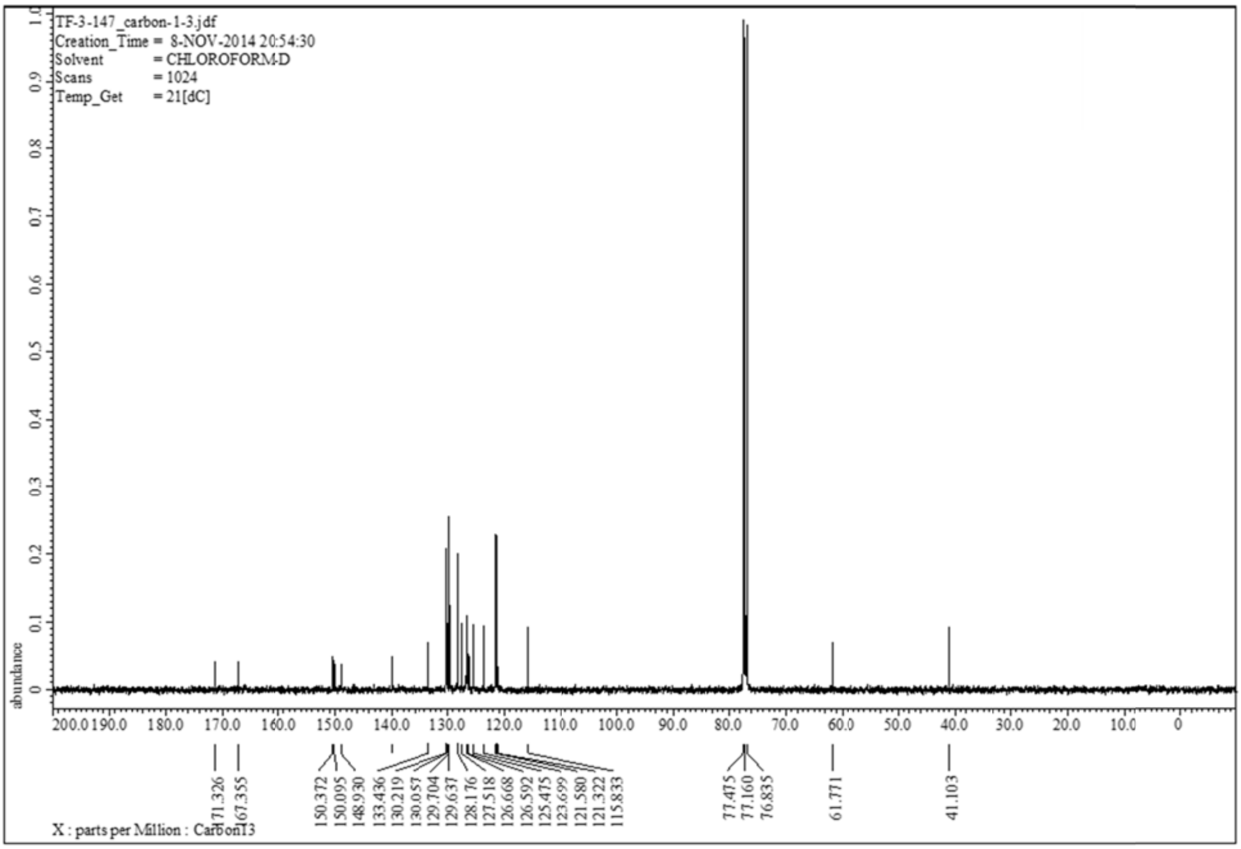

Supplementary Figure 45. ^{1}H and ^{13}C-NMR spectra for product **10d**

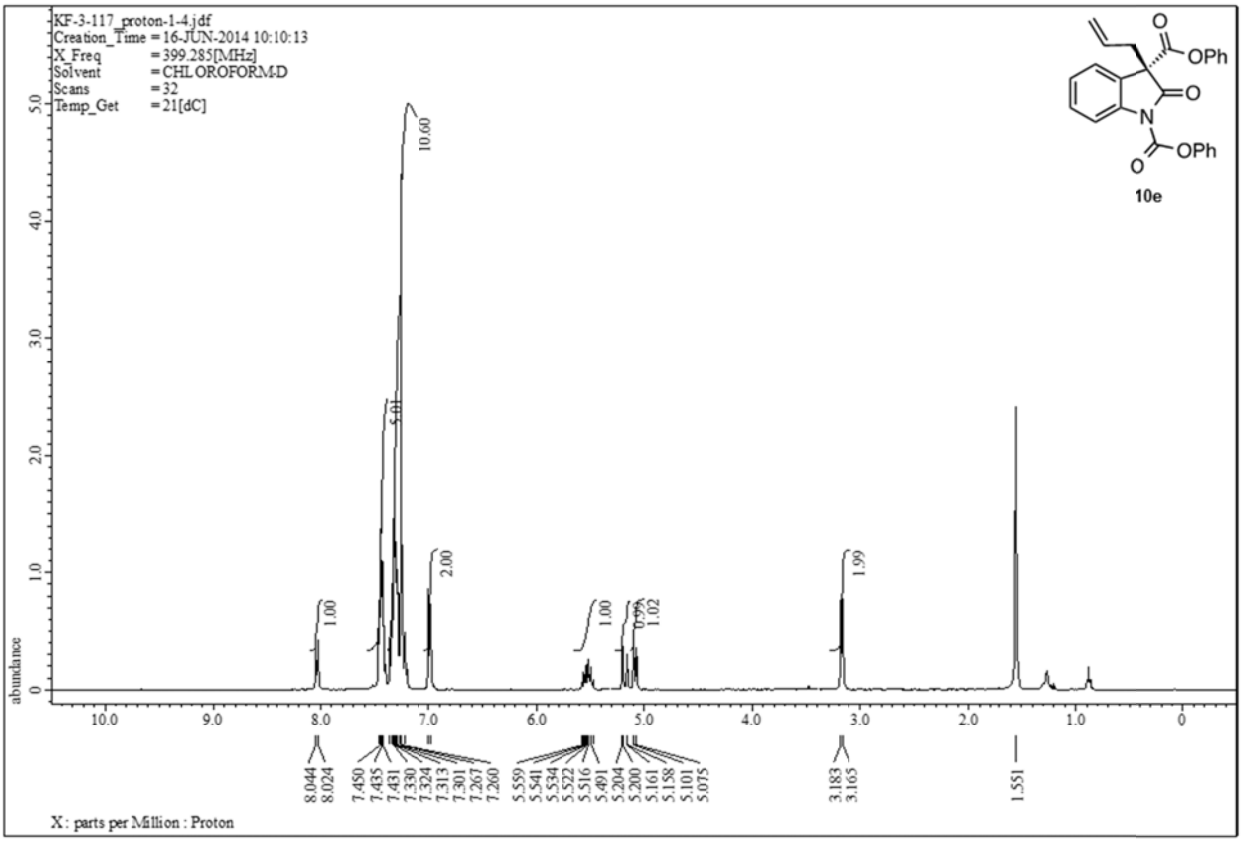

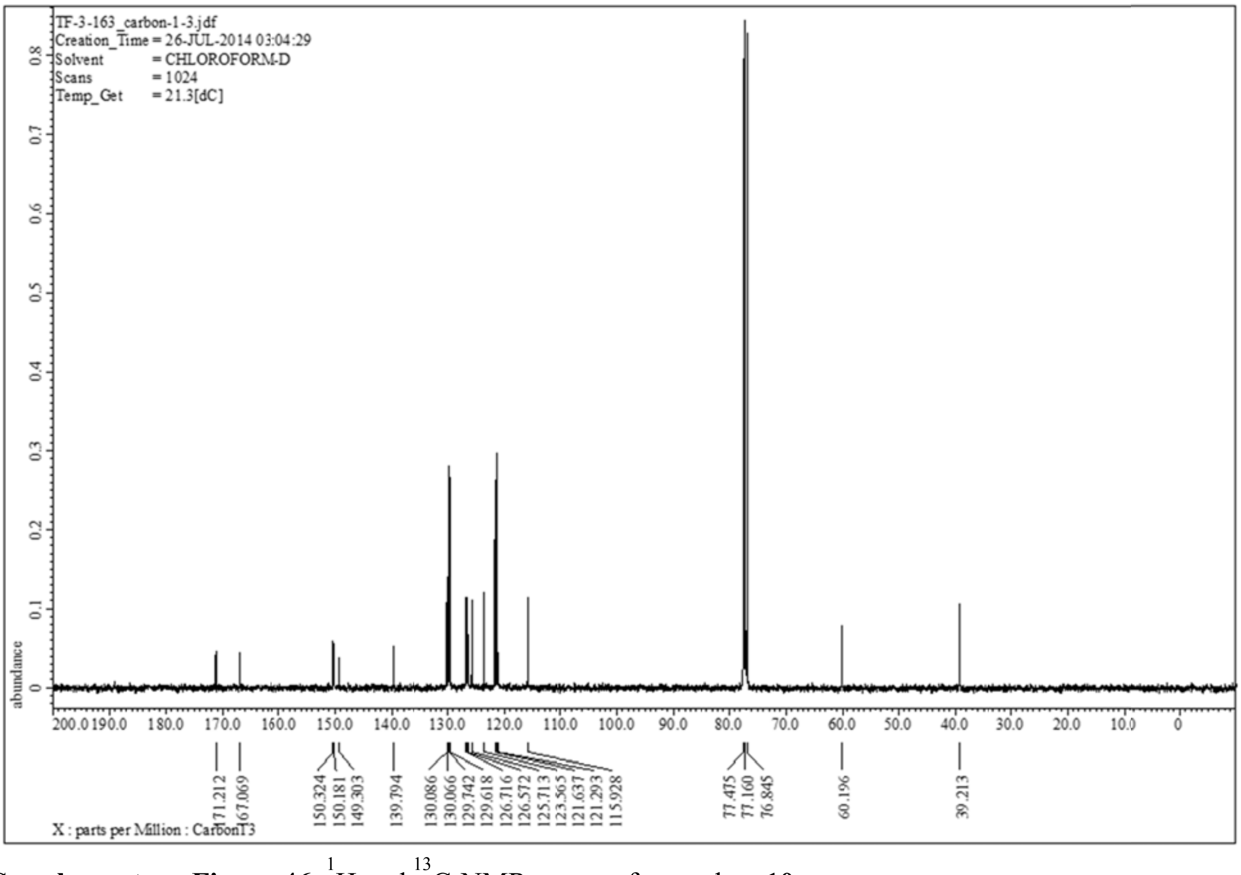

Supplementary Figure 46. ^{1}H and ^{13}C-NMR spectra for product **10e**

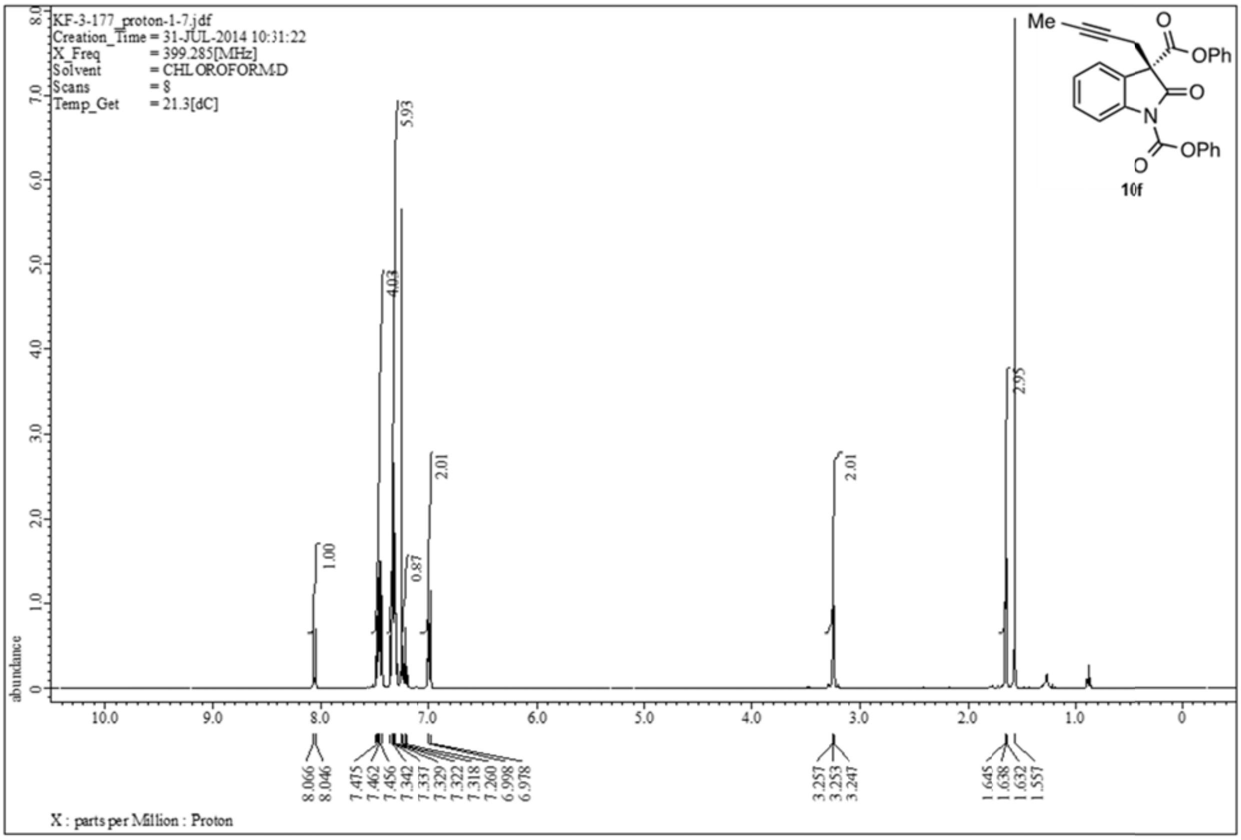

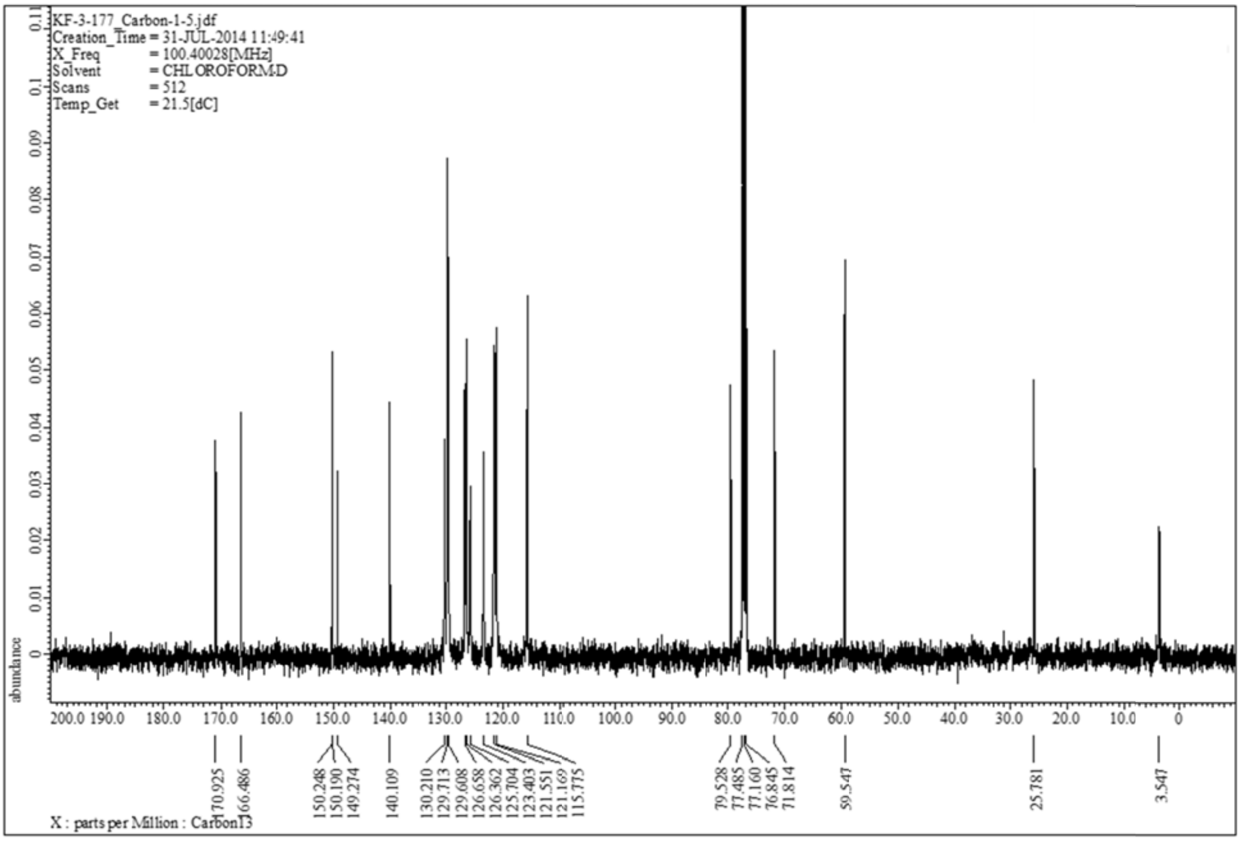

Supplementary Figure 47. ^{1}H and ^{13}C-NMR spectra for product **10f**

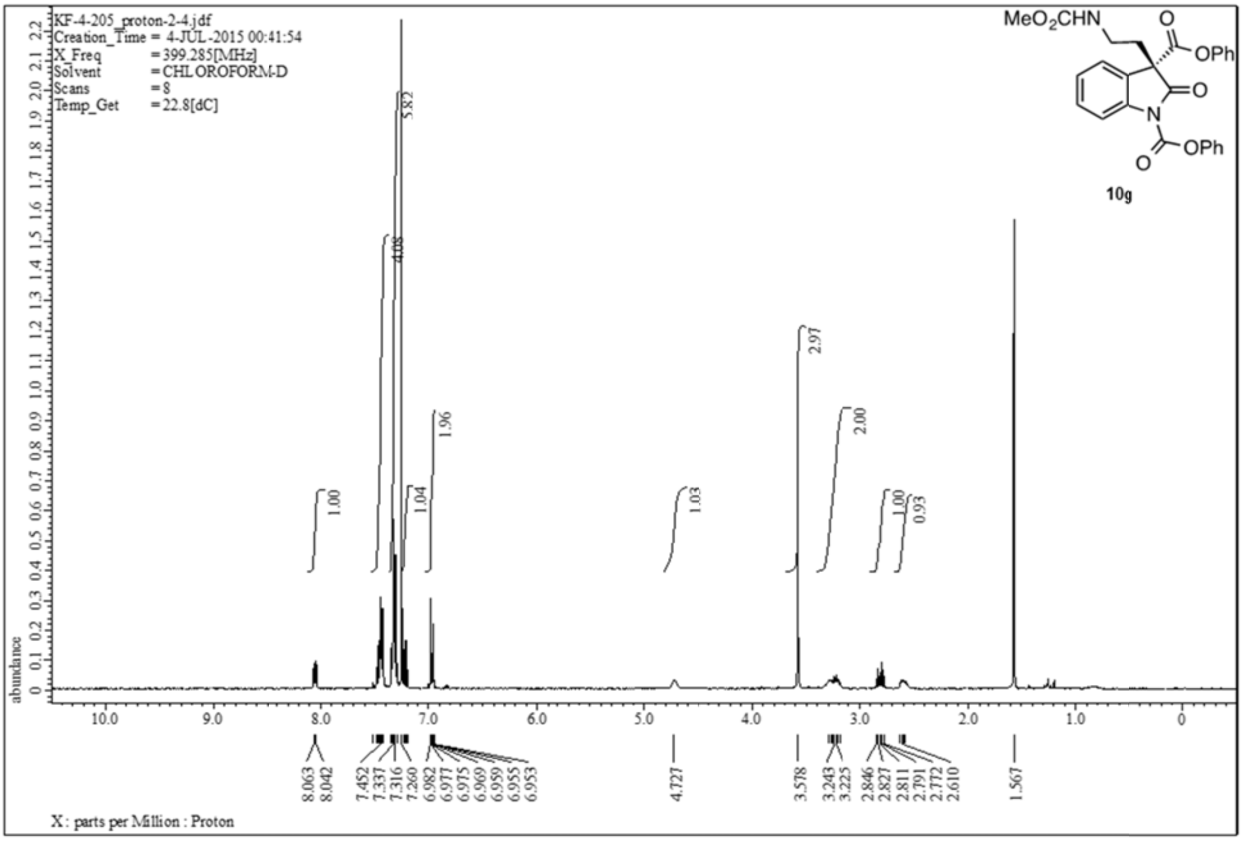

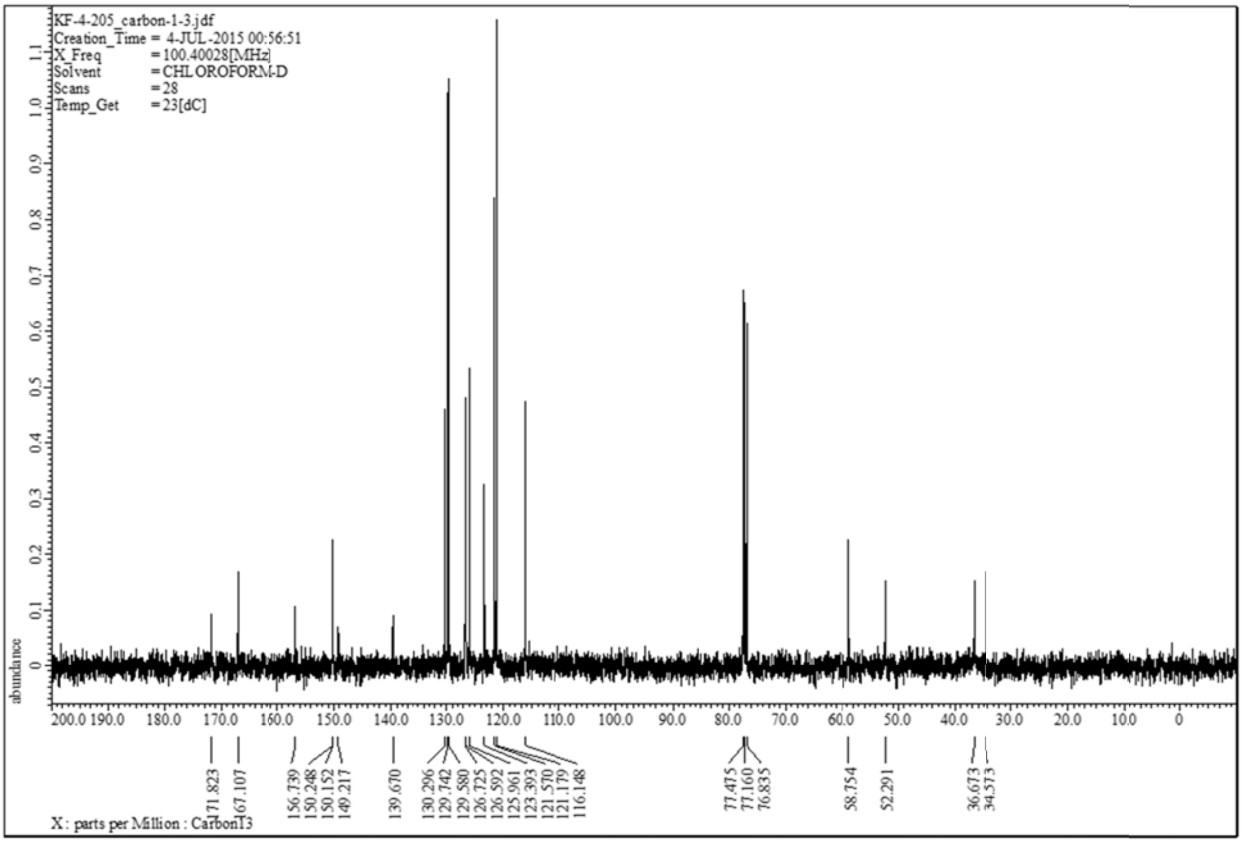

Supplementary Figure 48. ^{1}H and ^{13}C-NMR spectra for product **10g**

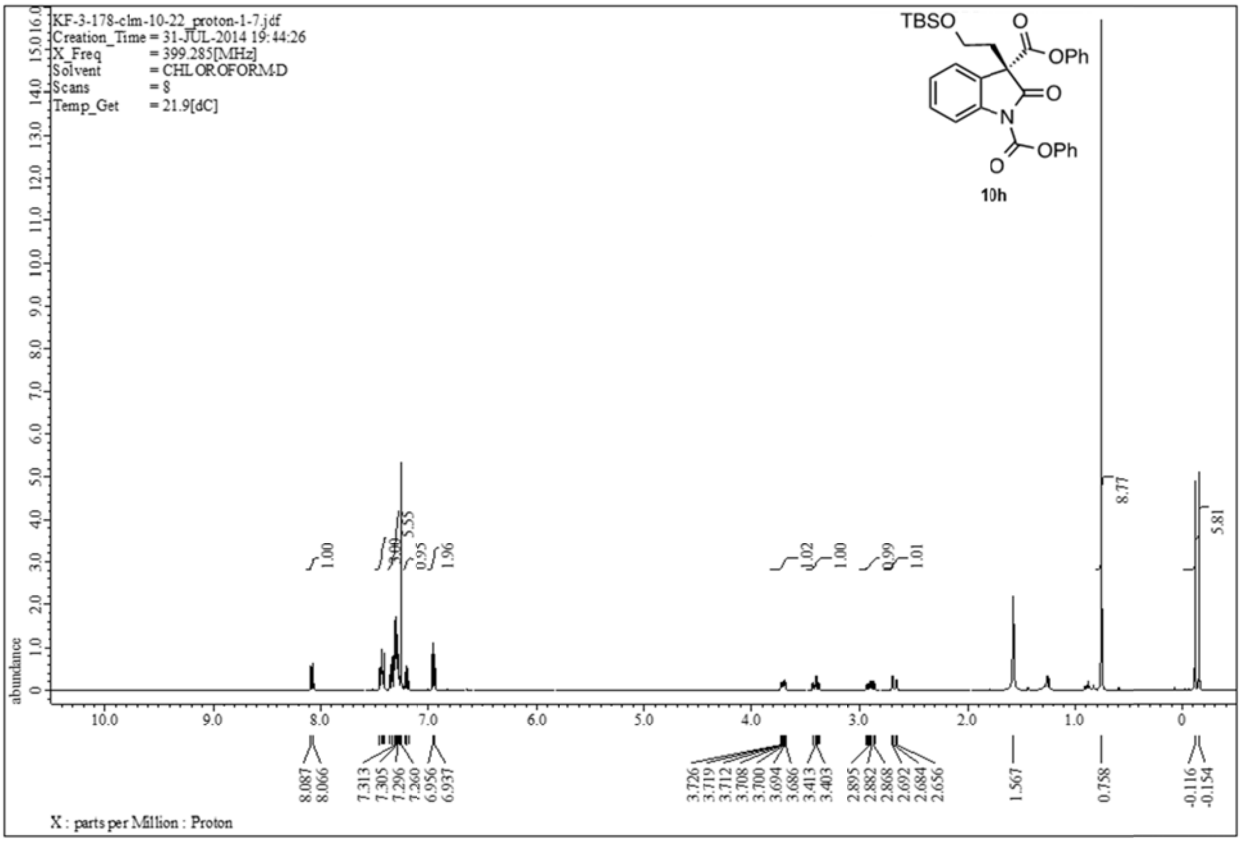

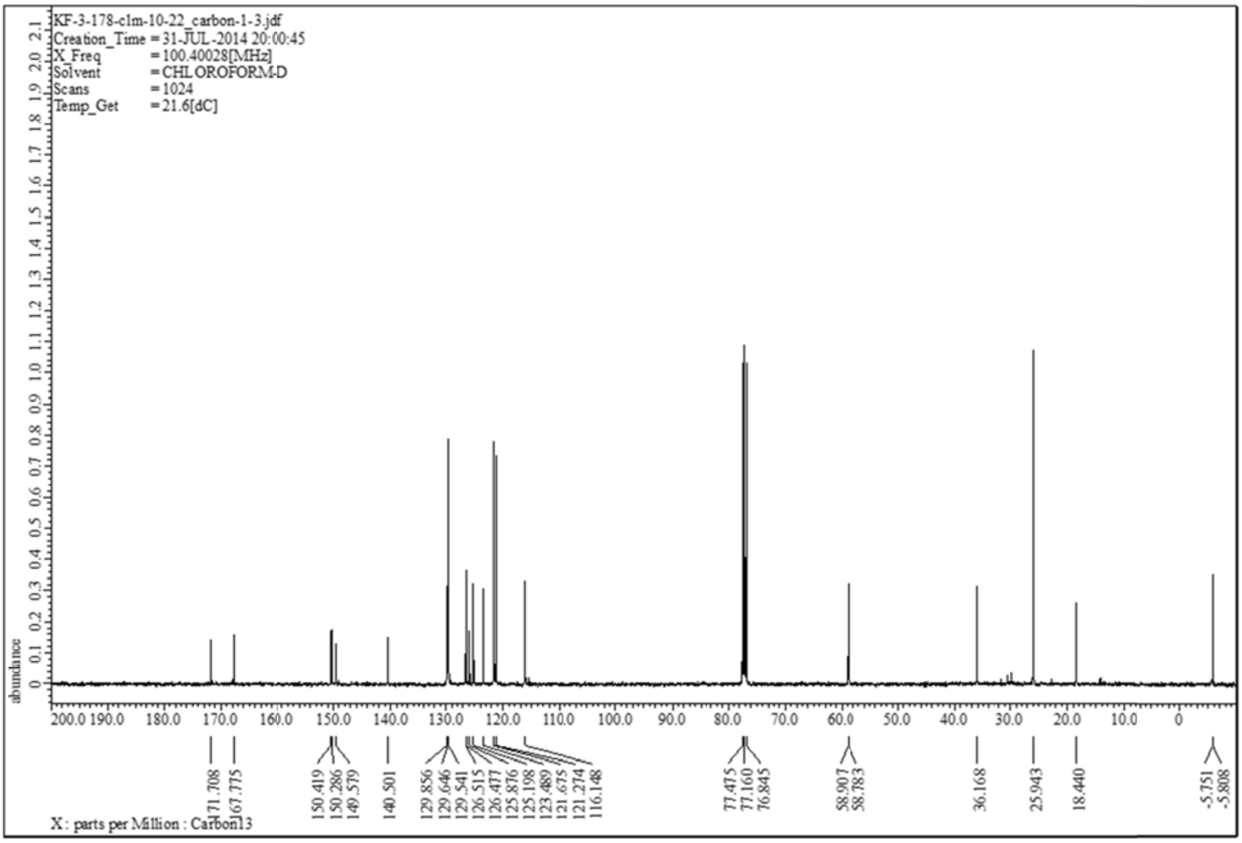

Supplementary Figure 49. ^{1}H and ^{13}C-NMR spectra for product **10h**

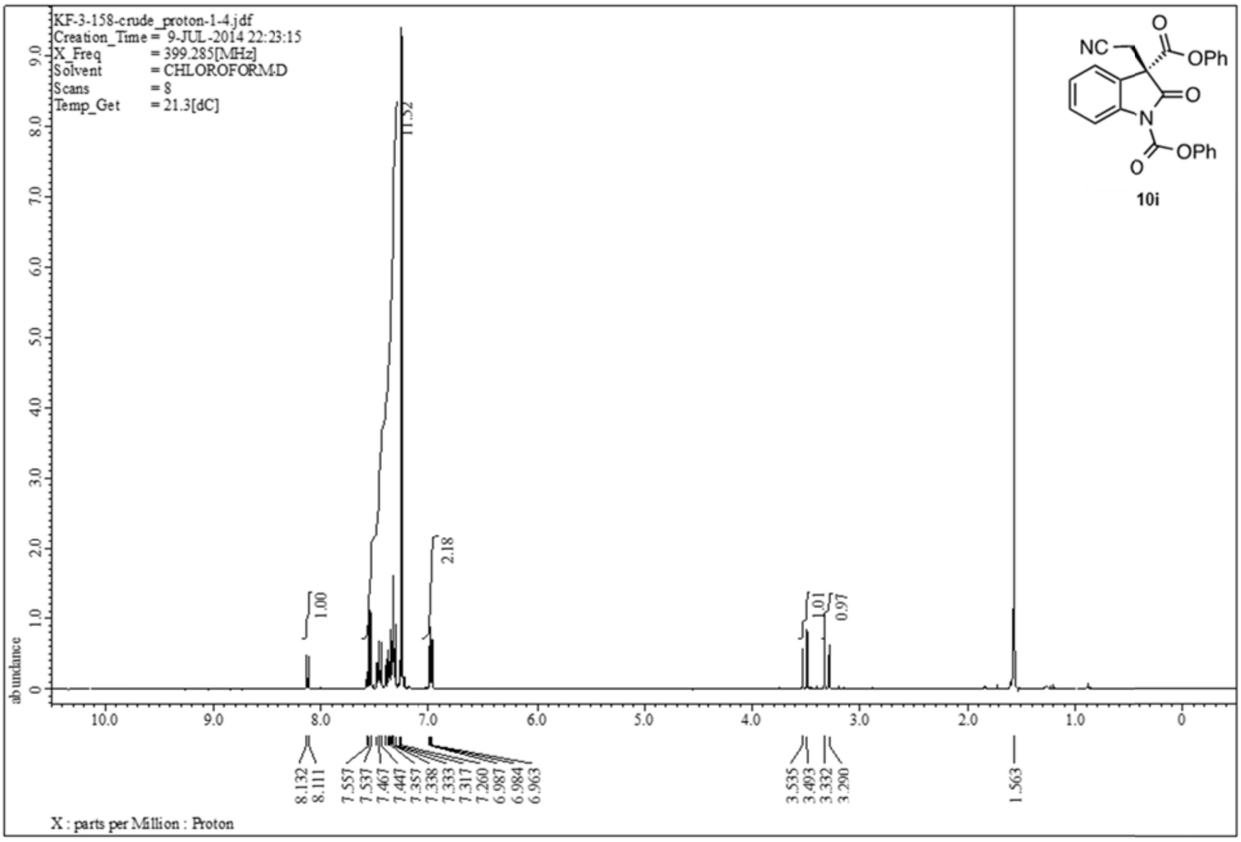

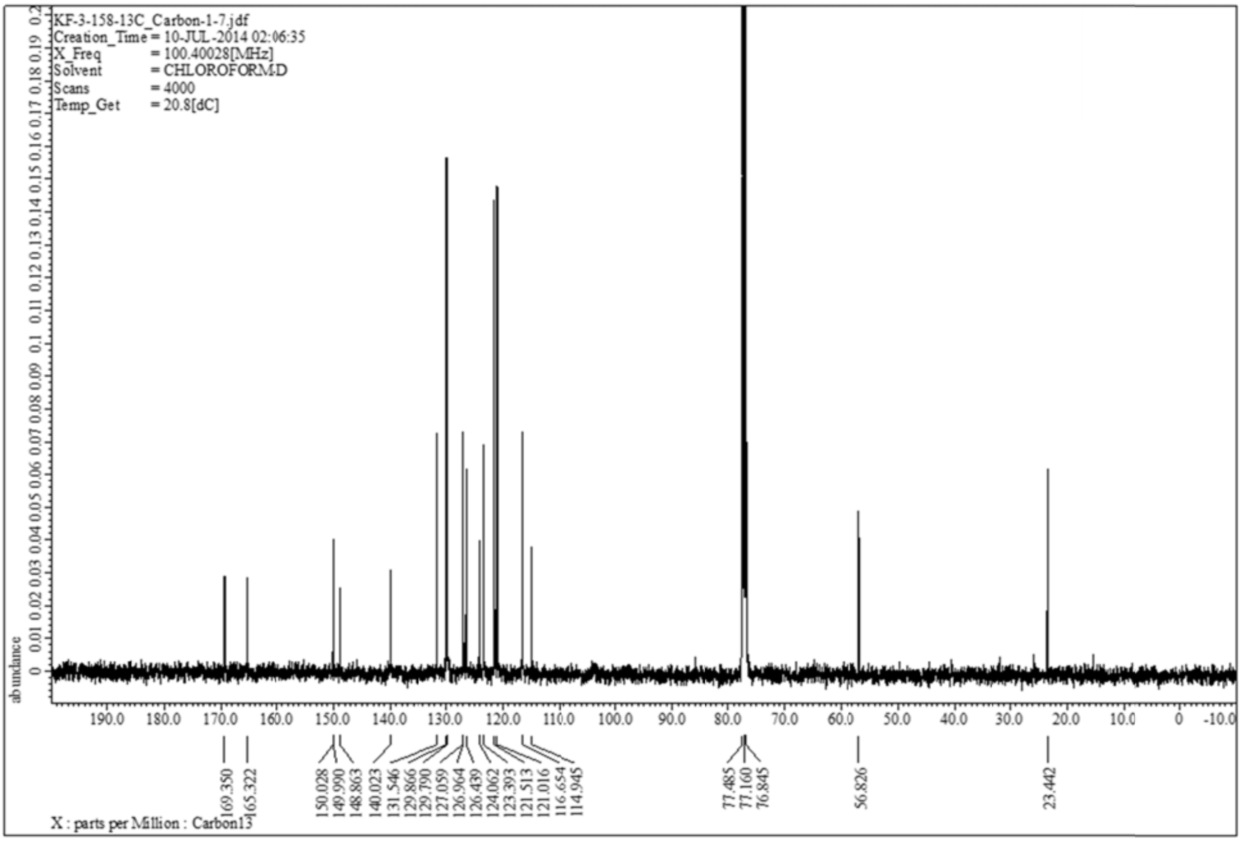

Supplementary Figure 50. ^{1}H and ^{13}C-NMR spectra for product **10i**

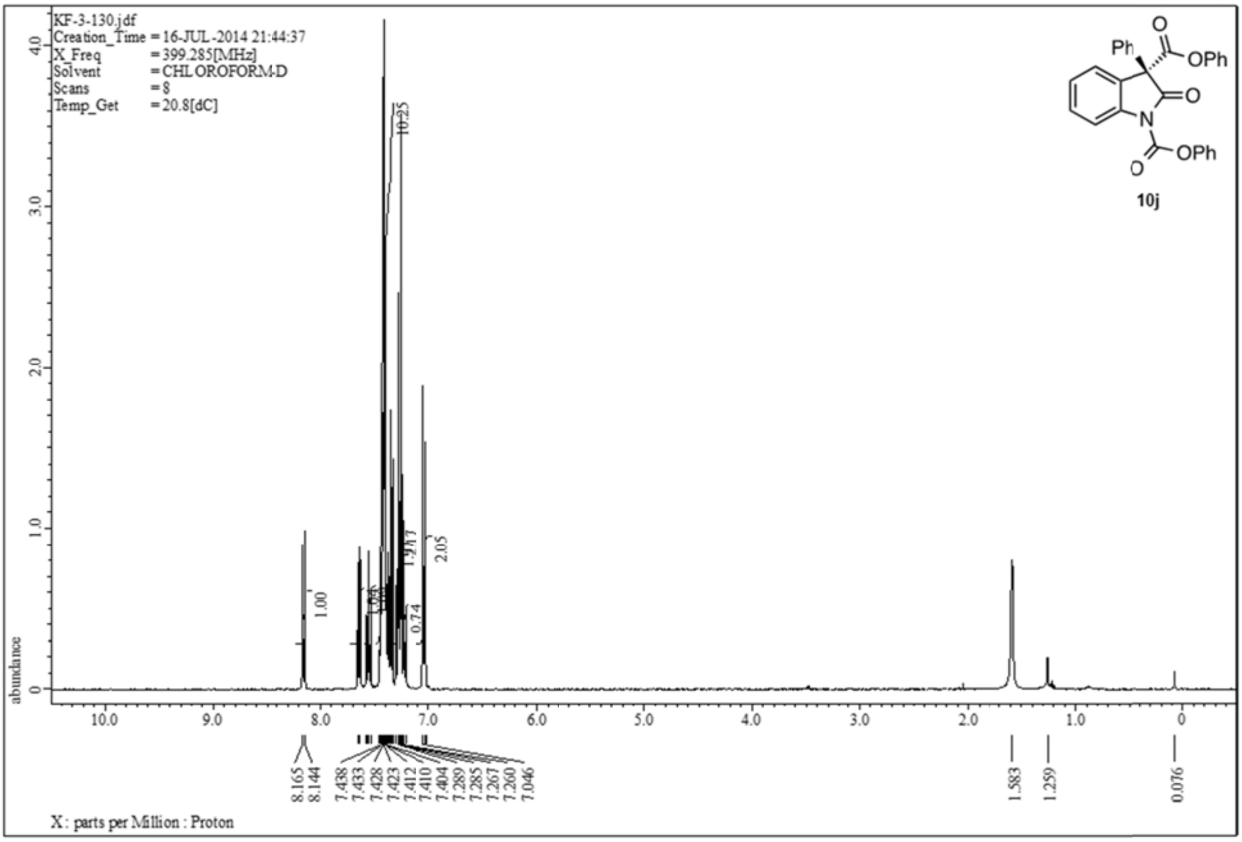

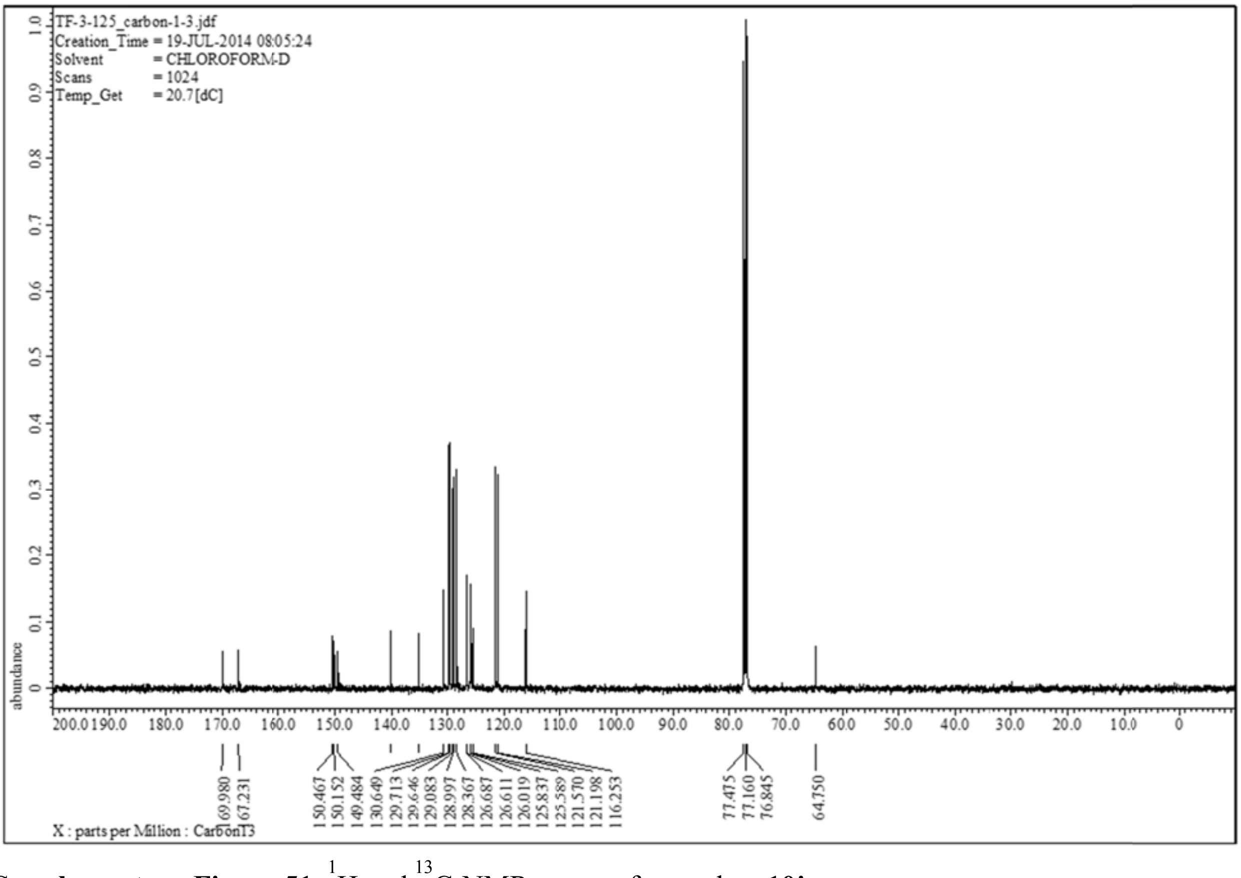

Supplementary Figure 51. ^{1}H and ^{13}C-NMR spectra for product **10j**

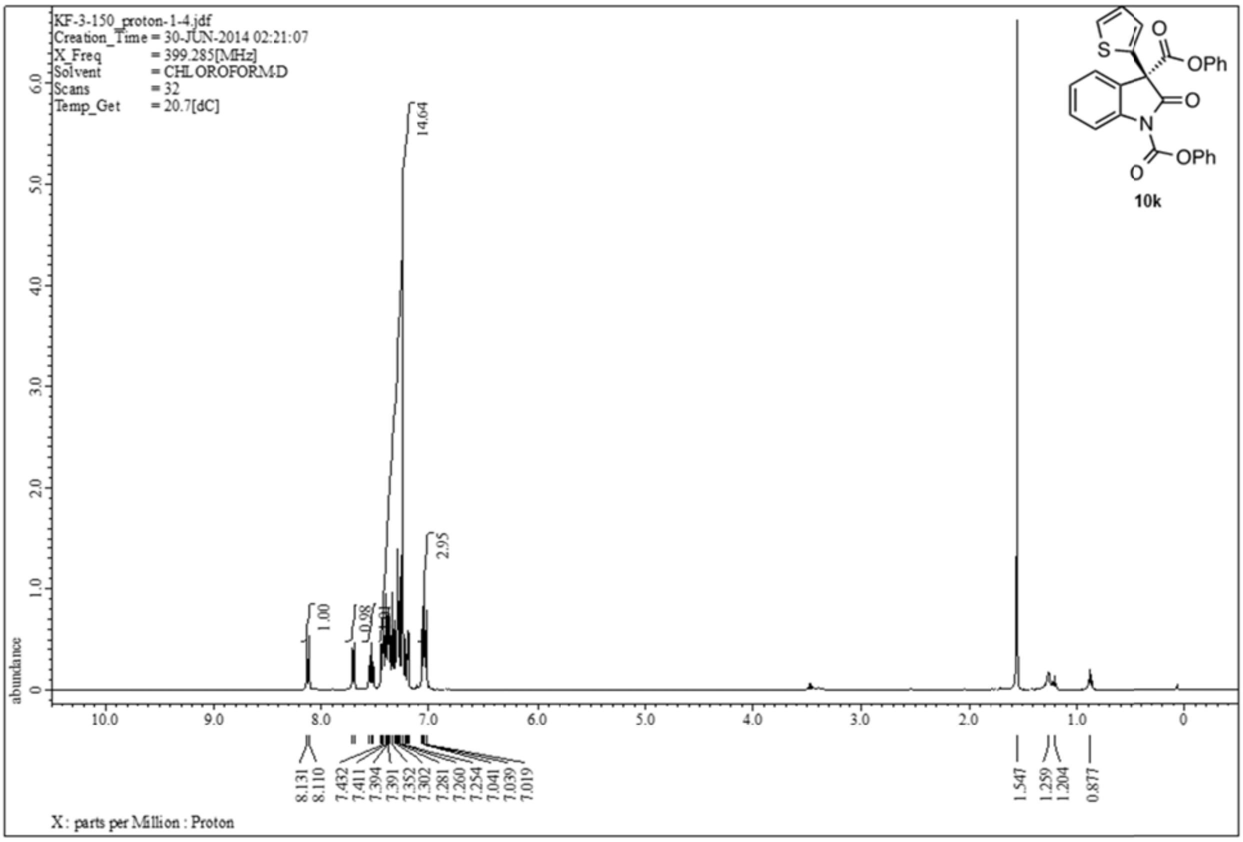

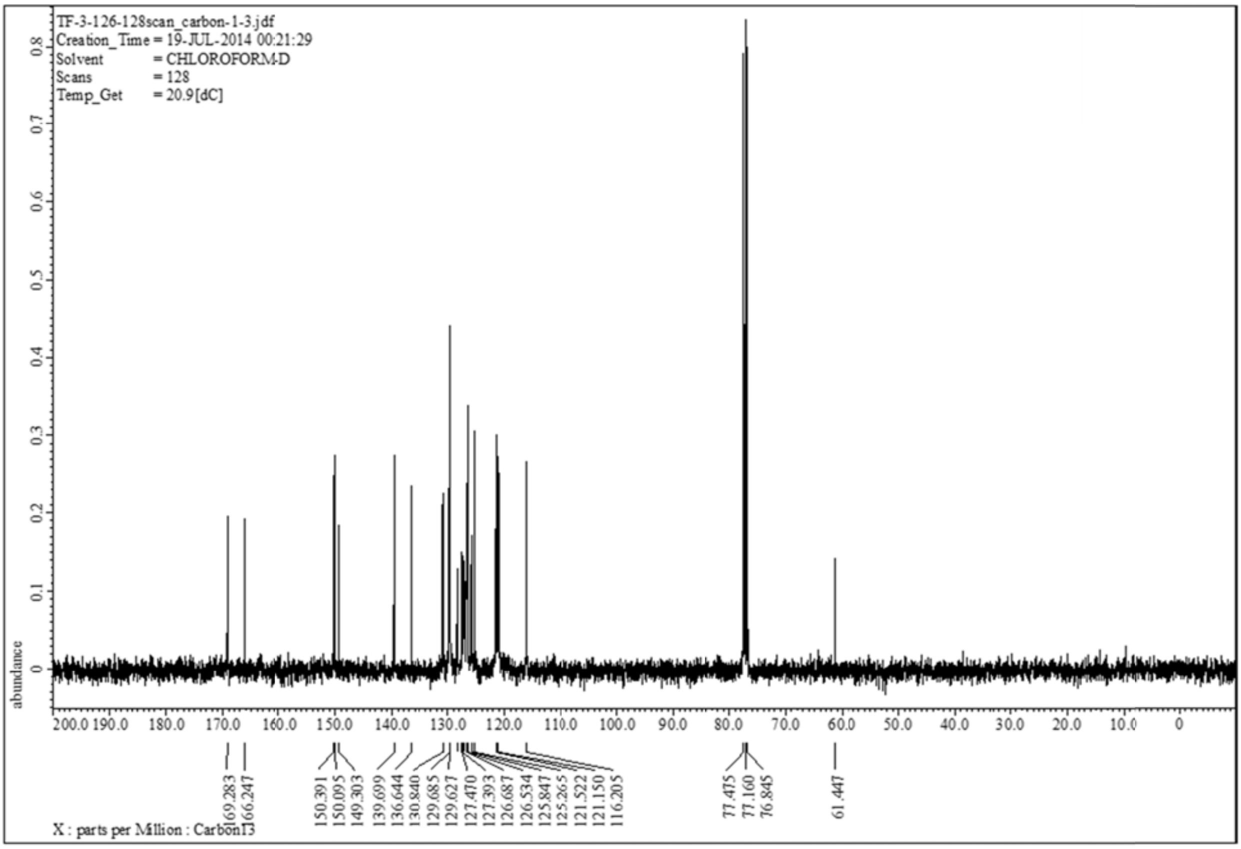

Supplementary Figure 52. ^{1}H and ^{13}C-NMR spectra for product **10k**

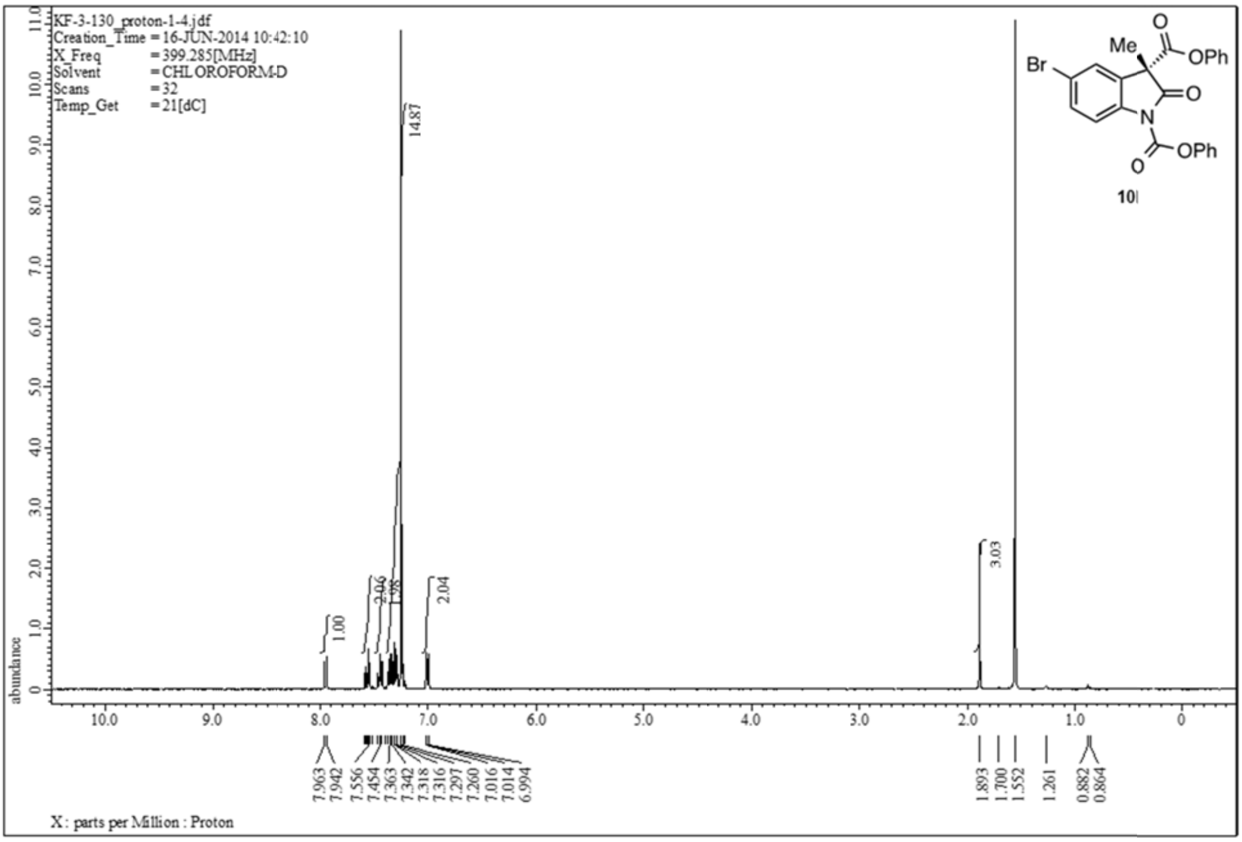

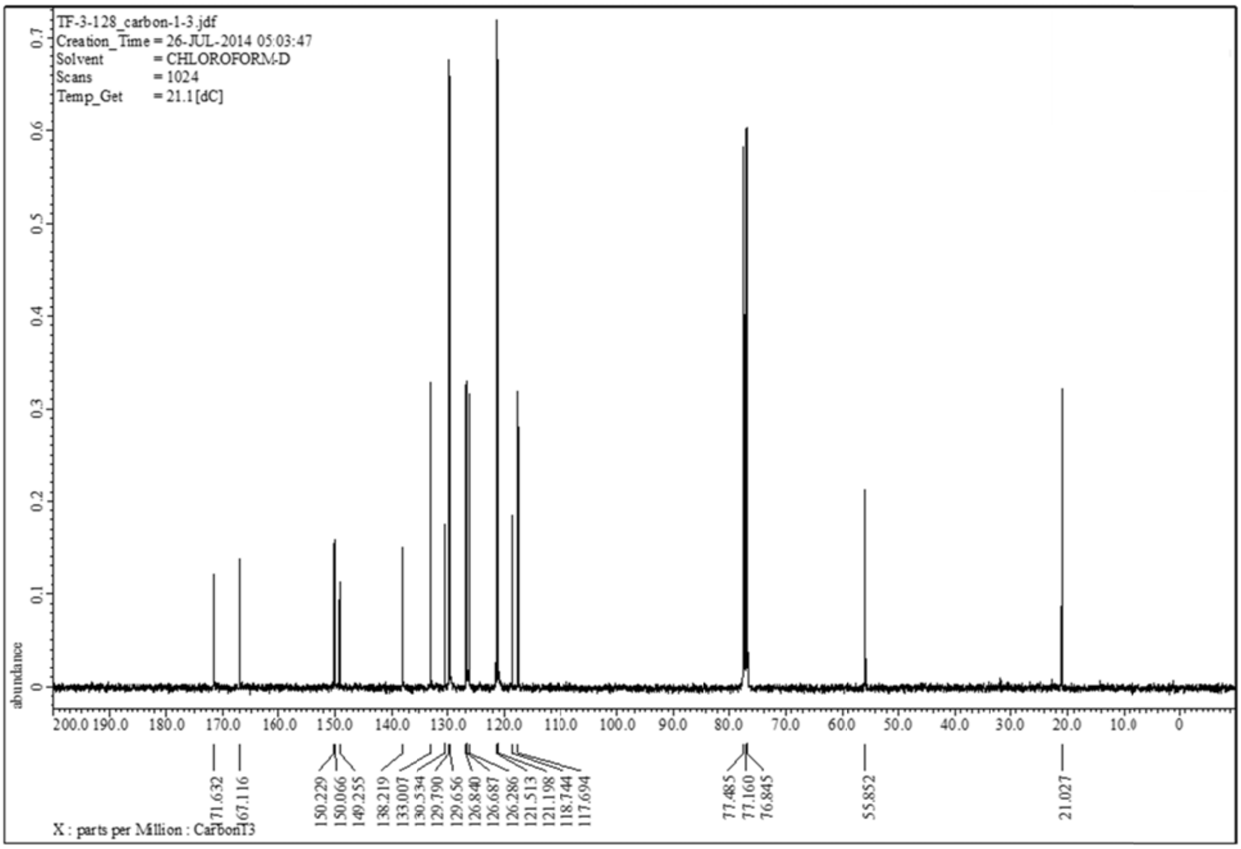

Supplementary Figure 53. ^{1}H and ^{13}C-NMR spectra for product **10l**

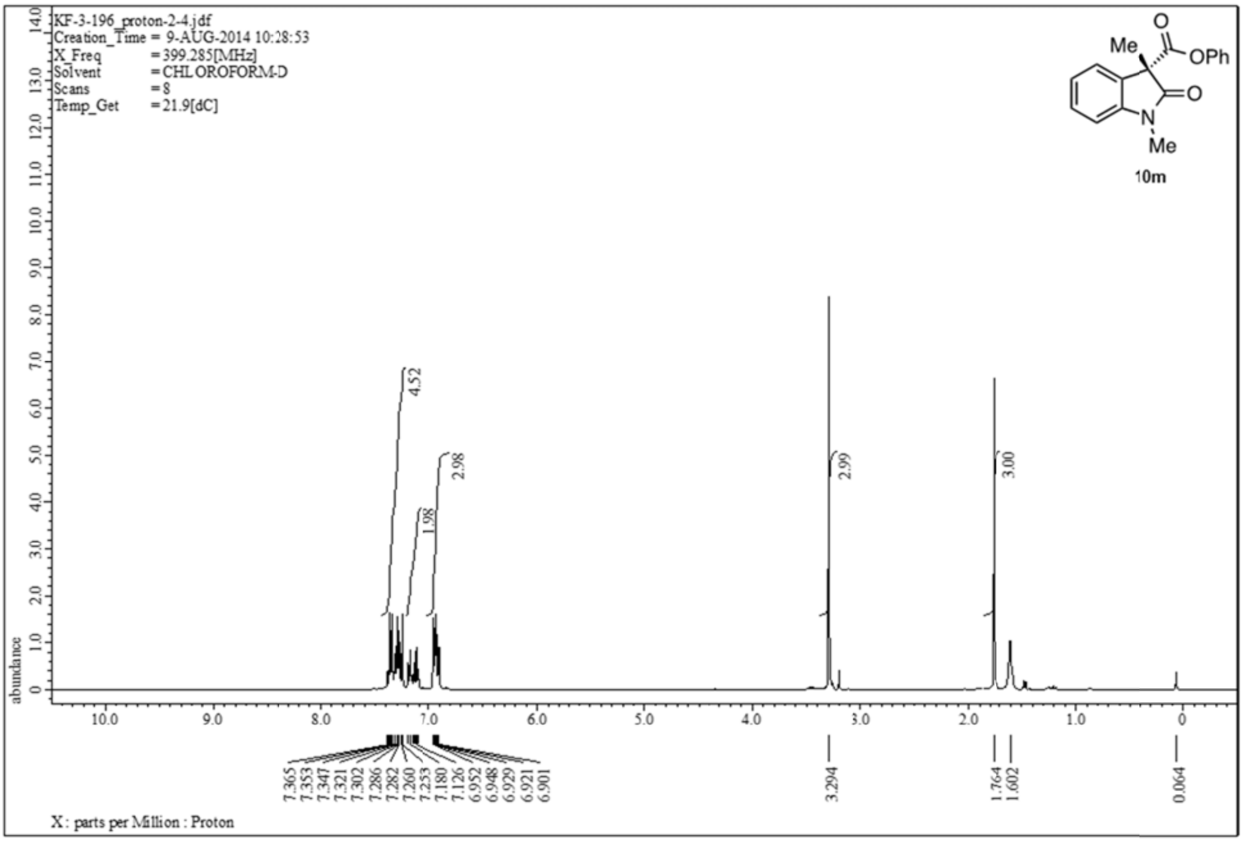

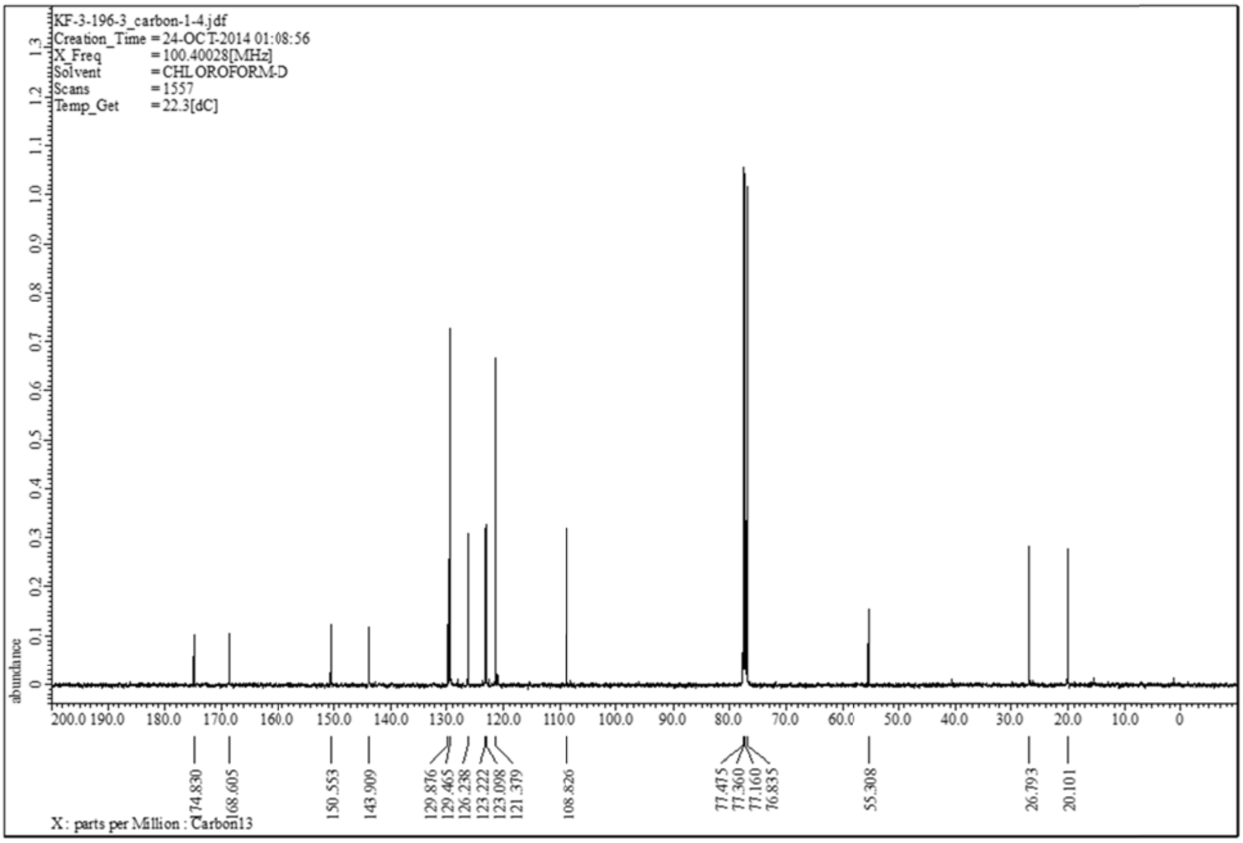

Supplementary Figure 54. ^{1}H and ^{13}C-NMR spectra for product **10m**

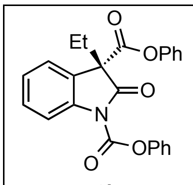

10b

Racemic sample of **10b**	(*S*)-**10b**
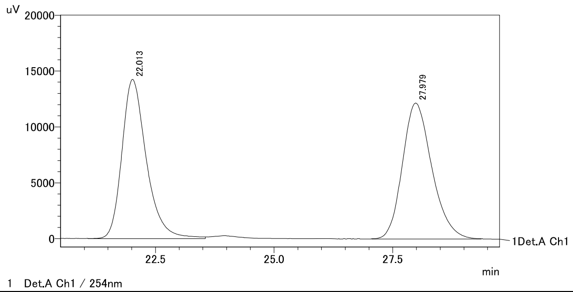	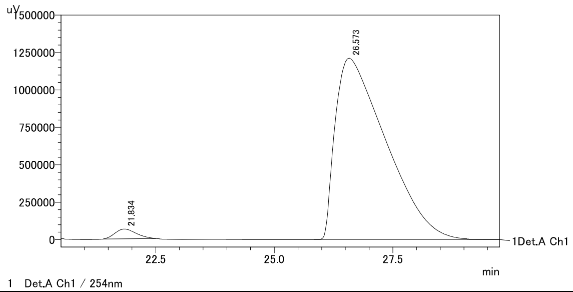

Peak #	Ret. Time	Area	Area %	Peak #	Ret. Time	Area	Area %
1	22.013	514169	49.559	1	21.834	2020595	2.198
2	27.979	523324	50.441	2	26.573	89913781	97.802

Supplementary Figure 55. HPLC spectra for product **10b** of Steglich rearrangement with catalyst **1j** (see Figure 5)

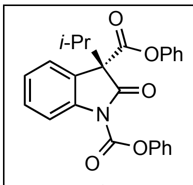

10c

Racemic sample of **10c**	(*S*)-**10c**
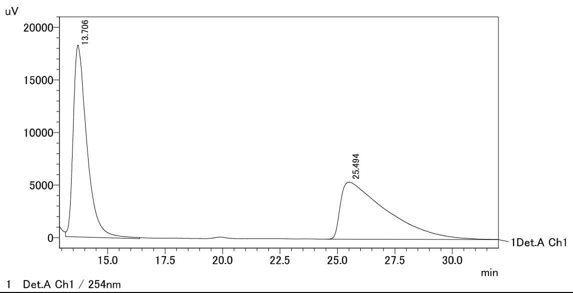	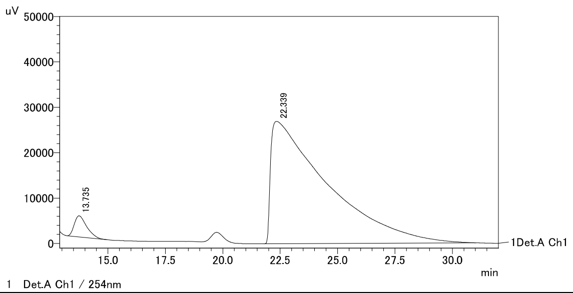

Peak #	Ret. Time	Area	Area %	Peak #	Ret. Time	Area	Area %
1	13.706	756612	49.881	1	13.735	175480	3.656
2	25.494	760216	50.119	2	22.339	4624284	96.344

Supplementary Figure 56. HPLC spectra for product **10c** of Steglich rearrangement with catalyst **1j** (see Figure 5)

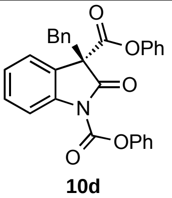

10d

Racemic sample of **10d**	(*S*)-**10d**

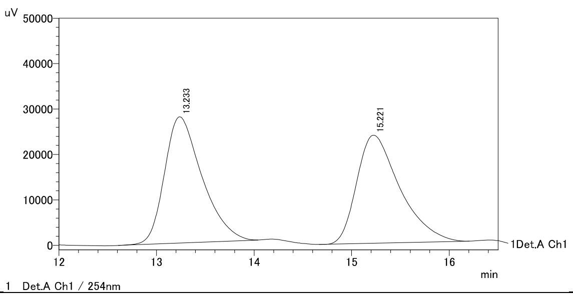

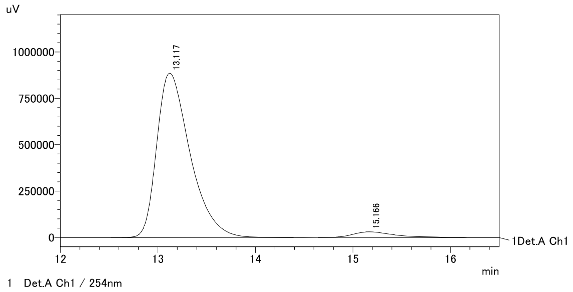

Peak #	Ret. Time	Area	Area %	Peak #	Ret. Time	Area	Area %
1	13.233	743275	50.426	1	13.117	21032544	95.986
2	15.221	730703	49.574	2	15.166	879600	4.014

Supplementary Figure 57. HPLC spectra for product **10d** of Steglich rearrangement with catalyst **1j** (see Figure 5)

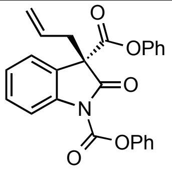

10e

Racemic sample of **10e**	(*S*)-**10e**

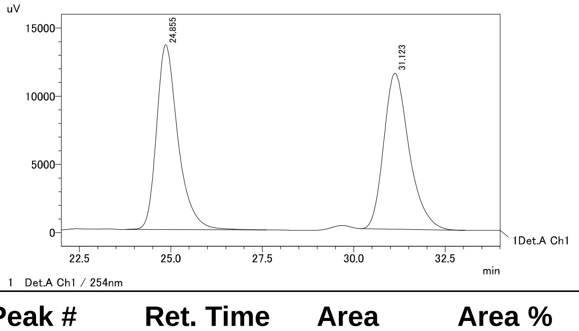

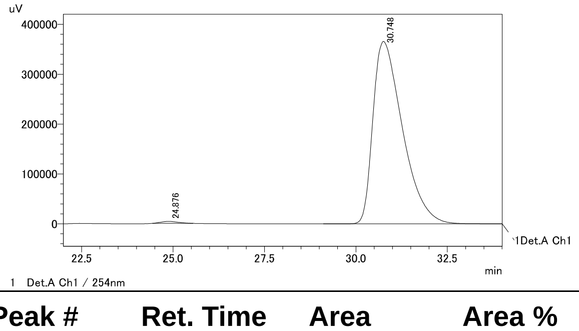

Peak #	Ret. Time	Area	Area %	Peak #	Ret. Time	Area	Area %
1	24.855	555399	50.042	1	24.876	129407	0.627
2	31.123	554476	49.958	2	30.748	20522041	99.373

Supplementary Figure 58. HPLC spectra for product **10e** of Steglich rearrangement with catalyst **1j** (see Figure 5)

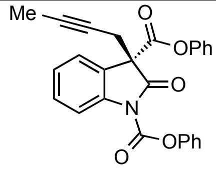

10f

Racemic sample of **10f**				(*S*)-**10f**			

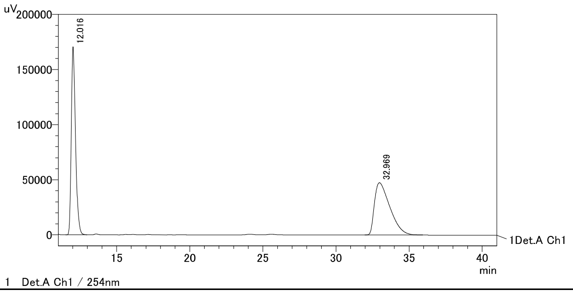

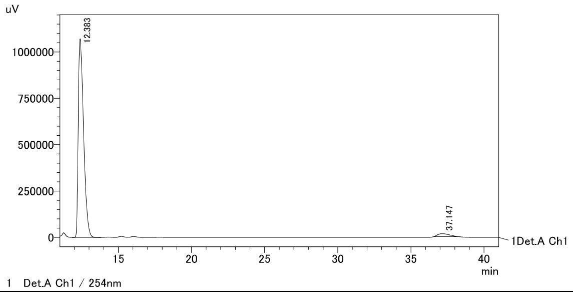

Peak #	Ret. Time	Area	Area %	Peak #	Ret. Time	Area	Area %
1	12.016	3434305	49.724	1	12.383	27117262	96.847
2	32.969	3472435	50.276	2	37.147	882922	3.153

Supplementary Figure 59. HPLC spectra for product **10f** of Steglich rearrangement with catalyst **1j** (see Figure 5)

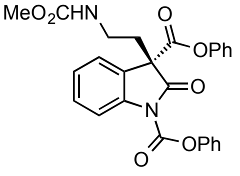

10g

Racemic sample of **10g**				(*S*)-**10g**			

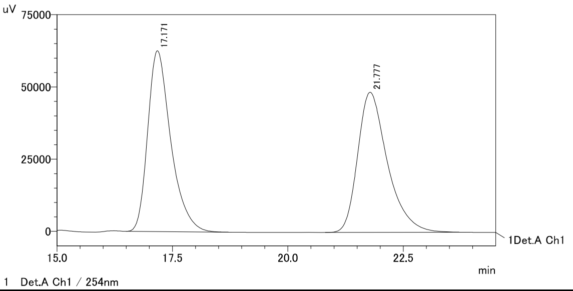

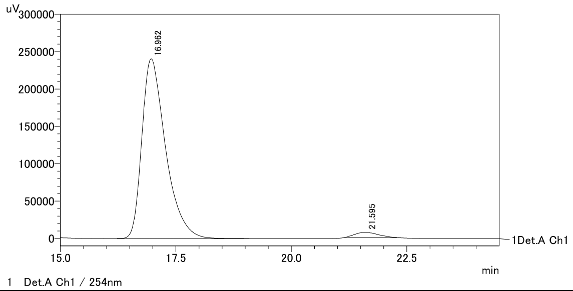

Peak #	Ret. Time	Area	Area %	Peak #	Ret. Time	Area	Area %
1	17.171	2182252	50.054	1	16.962	8578049	97.257
2	21.777	2177545	49.946	2	21.595	241908	2.743

Supplementary Figure 60. HPLC spectra for product **10g** of Steglich rearrangement with catalyst **1j** (see Figure 5)

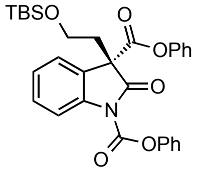

10h

Racemic sample of **10h**				(*S*)-**10h**			

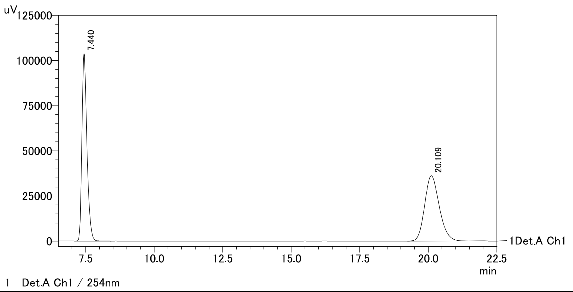

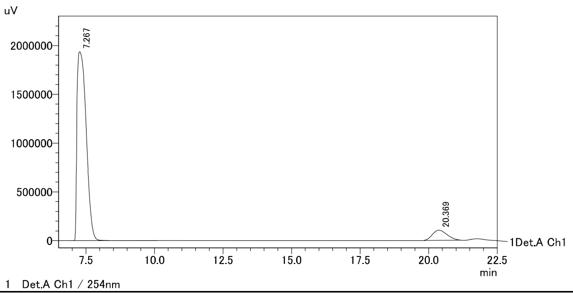

Peak #	Ret. Time	Area	Area %	Peak #	Ret. Time	Area	Area %
1	7.440	1325957	49.786	1	7.267	45012007	92.209
2	20.109	1337333	50.214	2	20.369	3803269	7.791

Supplementary Figure 61. HPLC spectra for product **10h** of Steglich rearrangement with catalyst **1j** (see Figure 5)

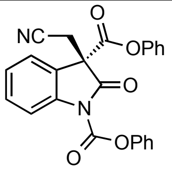

10i

Racemic sample of **10i**				(*S*)-**10i**			

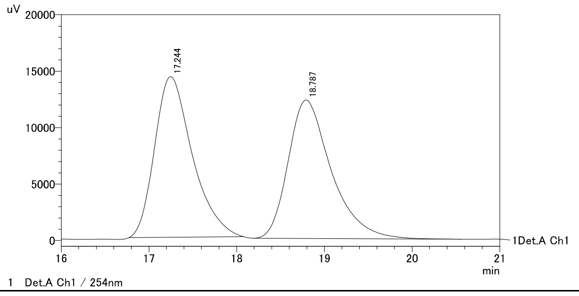

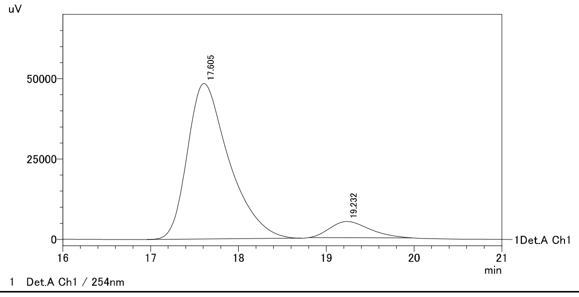

Peak #	Ret. Time	Area	Area %	Peak #	Ret. Time	Area	Area %
1	17.244	424561	50.356	1	17.605	1596012	90.985
2	18.787	418559	49.644	2	19.232	158136	9.015

Supplementary Figure 62. HPLC spectra for product **10i** of Steglich rearrangement with catalyst **1j** (see Figure 5)

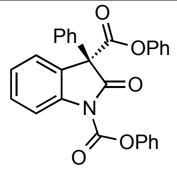

10j

Racemic sample of **10j**

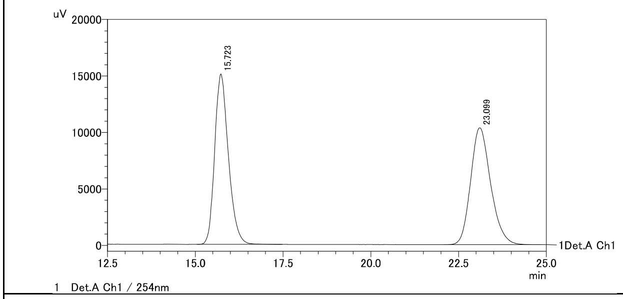

Peak #	Ret. Time	Area	Area %
1	15.723	409768	49.925
2	23.099	410996	50.075

(*S*)-**10j**

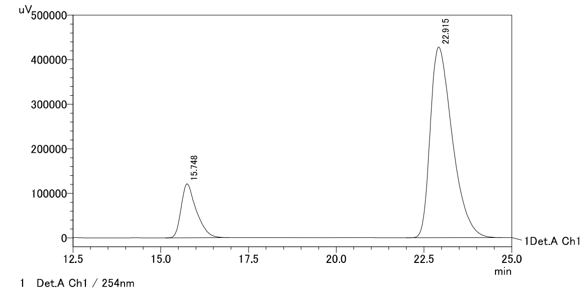

Peak #	Ret. Time	Area	Area %
1	15.748	3645897	16.244
2	22.915	18798408	83.756

Supplementary Figure 63. HPLC spectra for product **10j** of Steglich rearrangement with catalyst **1j** (see Figure 5)

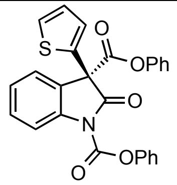

10k

Racemic sample of **10k**

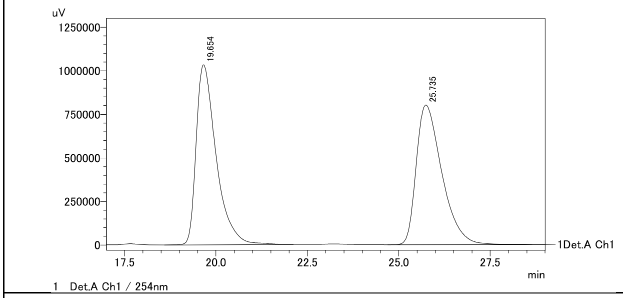

Peak #	Ret. Time	Area	Area %
1	19.654	39311038	50.136
2	25.735	39098419	49.864

(*R*)-**10k**

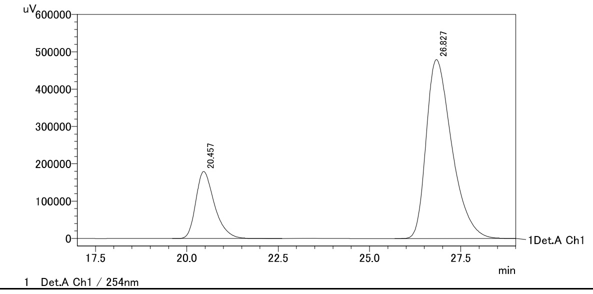

Peak #	Ret. Time	Area	Area %
1	20.457	6376265	21.396
2	26.827	23424552	78.604

Supplementary Figure 64. HPLC spectra for product **10k** of Steglich rearrangement with catalyst **1j** (see Figure 5)

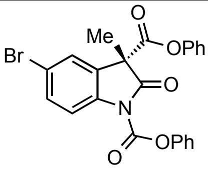

10l

Racemic sample of **10l**				(*S*)-**10l**			

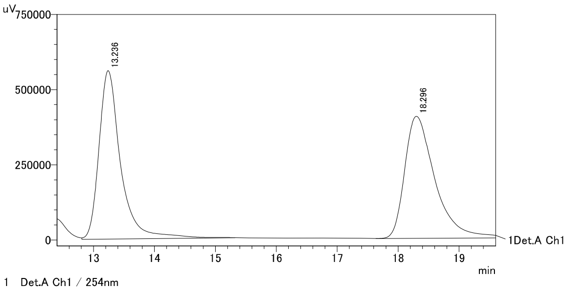

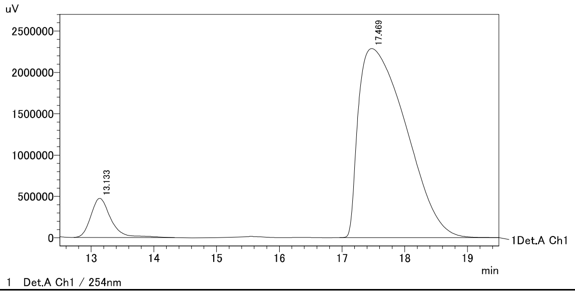

Peak #	Ret. Time	Area	Area %	Peak #	Ret. Time	Area	Area %
1	13.236	13821916	49.348	1	13.133	10516782	8.030
2	18.296	14187024	50.652	2	17.469	120452064	91.970

Supplementary Figure 65. HPLC spectra for product **10l** of Steglich rearrangement with catalyst **1j** (see Figure 5)

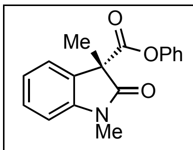

10m

Racemic sample of **10m**				(*S*)-**10m**			

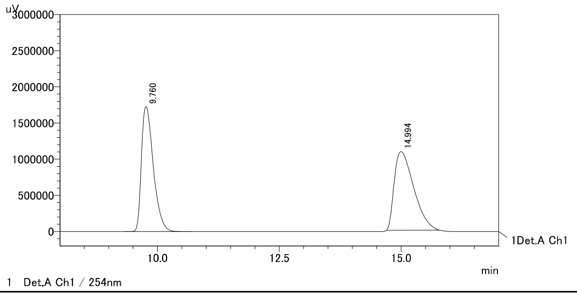

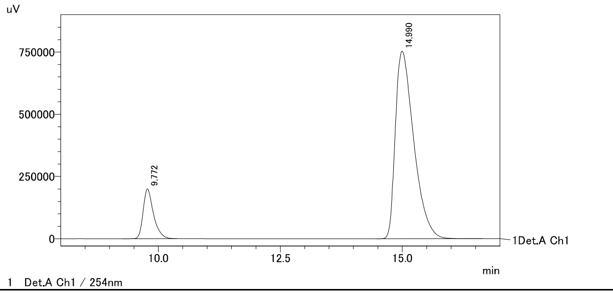

Peak #	Ret. Time	Area	Area %	Peak #	Ret. Time	Area	Area %
1	9.760	29335890	49.263	1	9.772	2945760	12.965
2	14.994	30213315	50.737	2	14.990	19774980	87.035

Supplementary Figure 66. HPLC spectra for product **10m** of Steglich rearrangement with catalyst **1j** (see Figure 5)

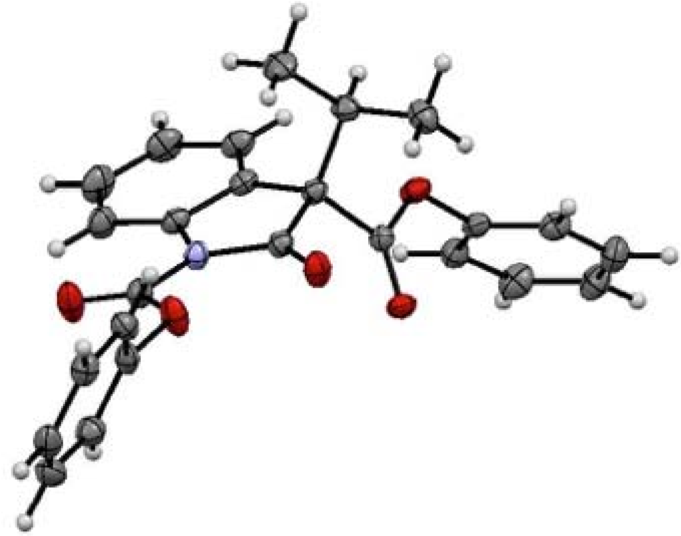

Supplementary Figure 67. X-ray structure of product **10c**

Racemic sample of **10a**, **10n**, **10a´**and **10n´**

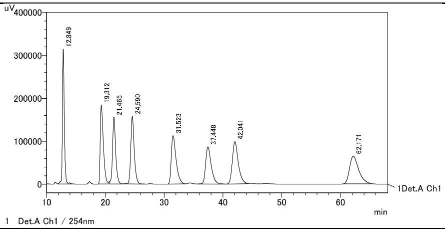

Peak #	Ret. Time	Area	Area %
1	12.849	6776862	13.088
2	19.312	6773022	13.081
3	21.465	5711902	11.032
4	24.590	6483558	12.522
5	31.523	6452442	12.462
6	37.448	5710356	11.029
7	42.041	6934043	13.392
8	62.171	6935636	13.395

(*S*)- **10a**, **10n**, **10a´** and **10n´**

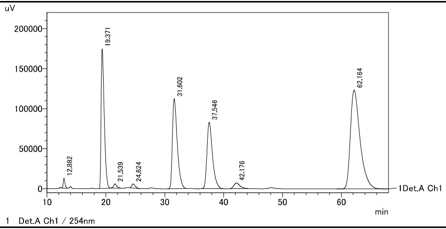

Peak #	Ret. Time	Area	Area %
1	12.882	260894	0.779
2	19.371	6386553	19.065
3	21.539	199224	0.595
4	24.624	212607	0.635
5	31.602	6433427	19.205
6	37.546	5450315	16.270
7	42.176	514876	1.537
8	62.164	14040320	41.914

Racemic sample of **10a**

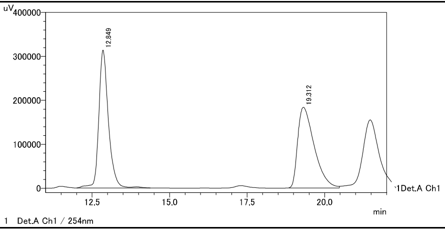

Peak #	Ret. Time	Area	Area %
1	12.849	6776862	50.014
2	19.312	6773022	49.986

(*S*)-**10a**

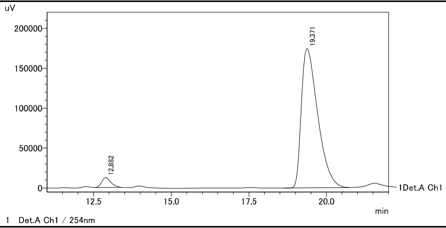

Peak #	Ret. Time	Area	Area %
1	12.882	251216	3.780
2	19.371	6394711	96.220

Racemic sample of **10a´**or **10n´**

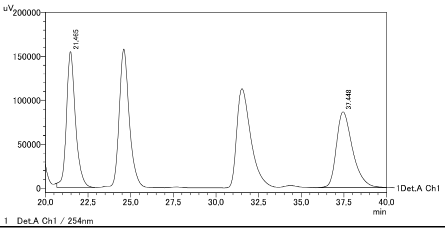

Peak #	Ret. Time	Area	Area %
1	21.465	5711902	50.007
2	37.448	5710356	49.993

(*S*)-**10a´**or **10n´**

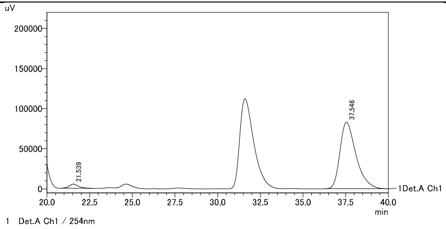

Peak #	Ret. Time	Area	Area %
1	21.539	197158	3.500
2	37.546	5436404	96.500

Racemic sample of **10a´**or **10n´**

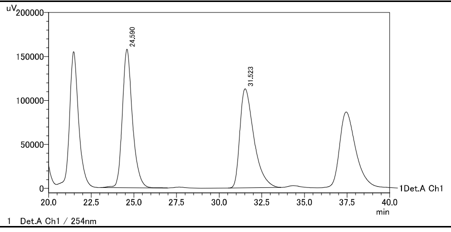

Peak #	Ret. Time	Area	Area %
1	24.590	6483558	50.120

(*S*)-**10a´**or **10n´**

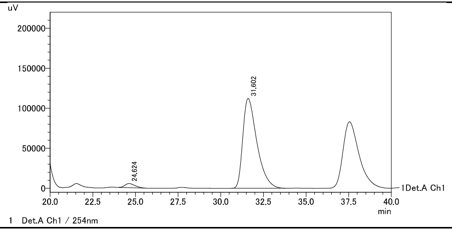

Peak #	Ret. Time	Area	Area %
1	24.624	208941	3.141

2	31.523	6452442	49.880	2	31.602	6442366	96.859
Racemic sample of **10n**				(*S*)-**10n**			
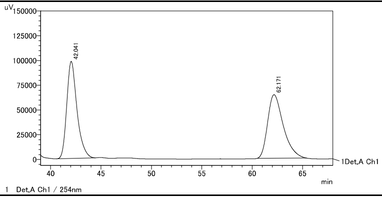				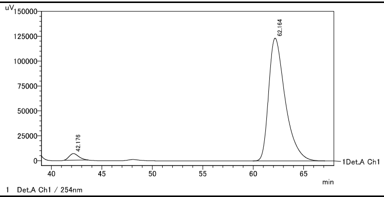			
**Peak #**	**Ret. Time**	**Area**	**Area %**	**Peak #**	**Ret. Time**	**Area**	**Area %**
1	42.041	6934043	49.994	1	42.176	435525	3.011
2	62.171	6935636	50.006	2	62.164	14027941	96.989

Supplementary Figure 68. HPLC spectra for cross over products **10a**, **10n**, **10a´** and **10n´ in Fig. 6**

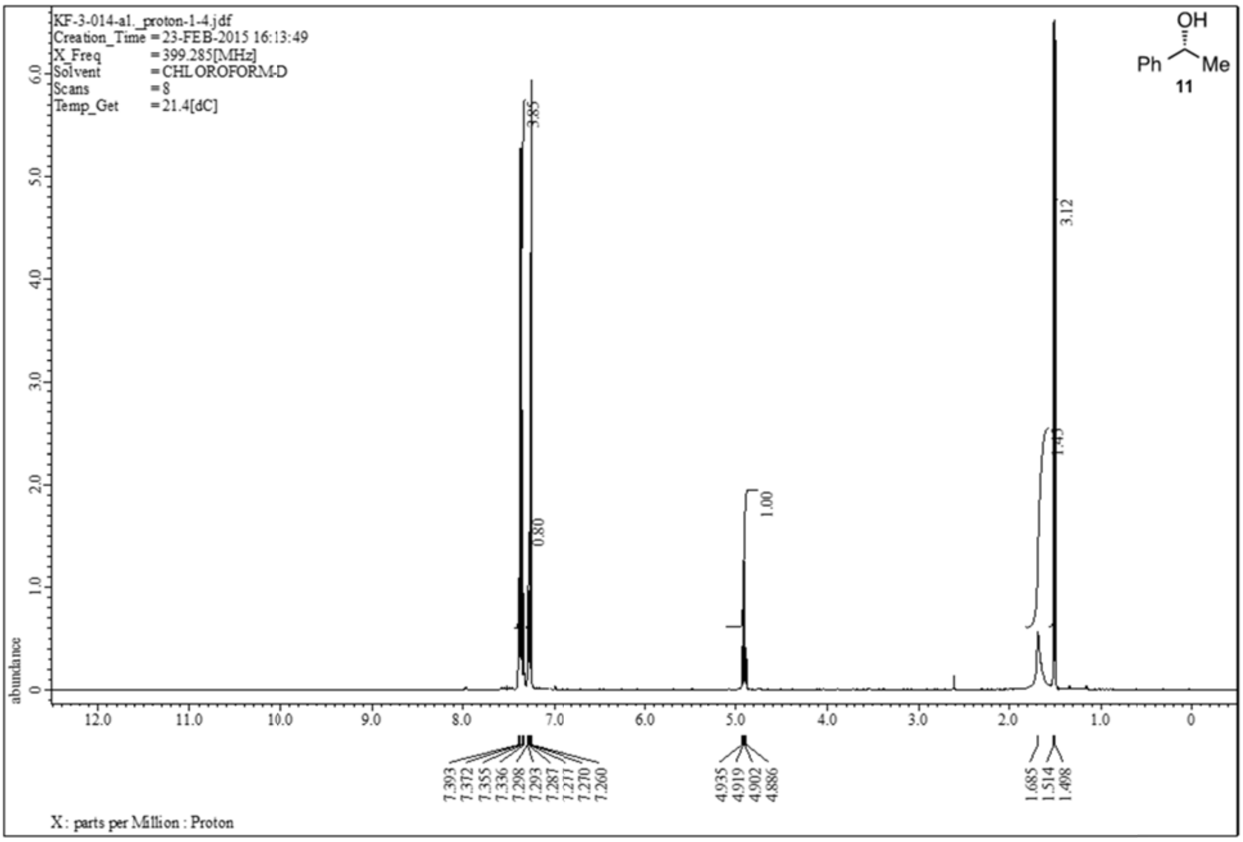

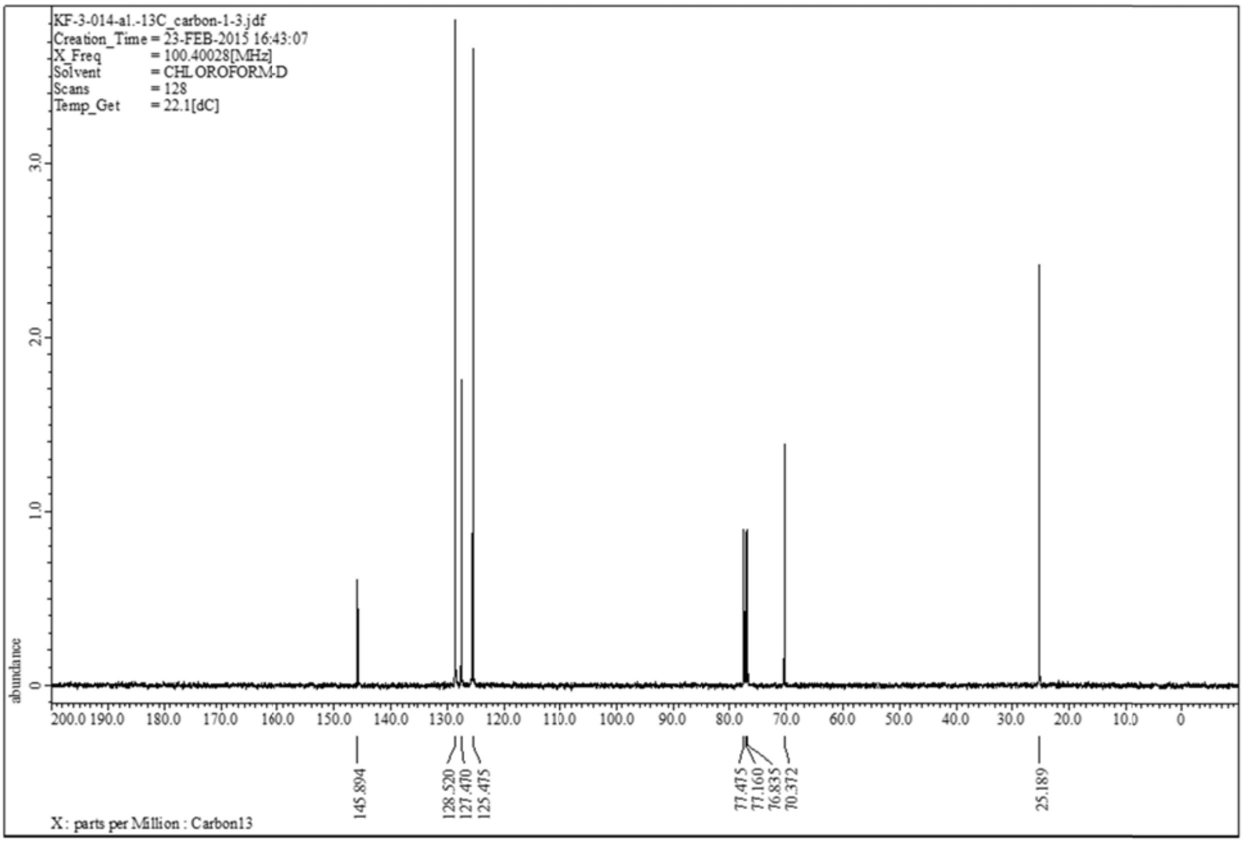

Supplementary Figure 69. ^{1}H and ^{13}C-NMR spectra for product **11**

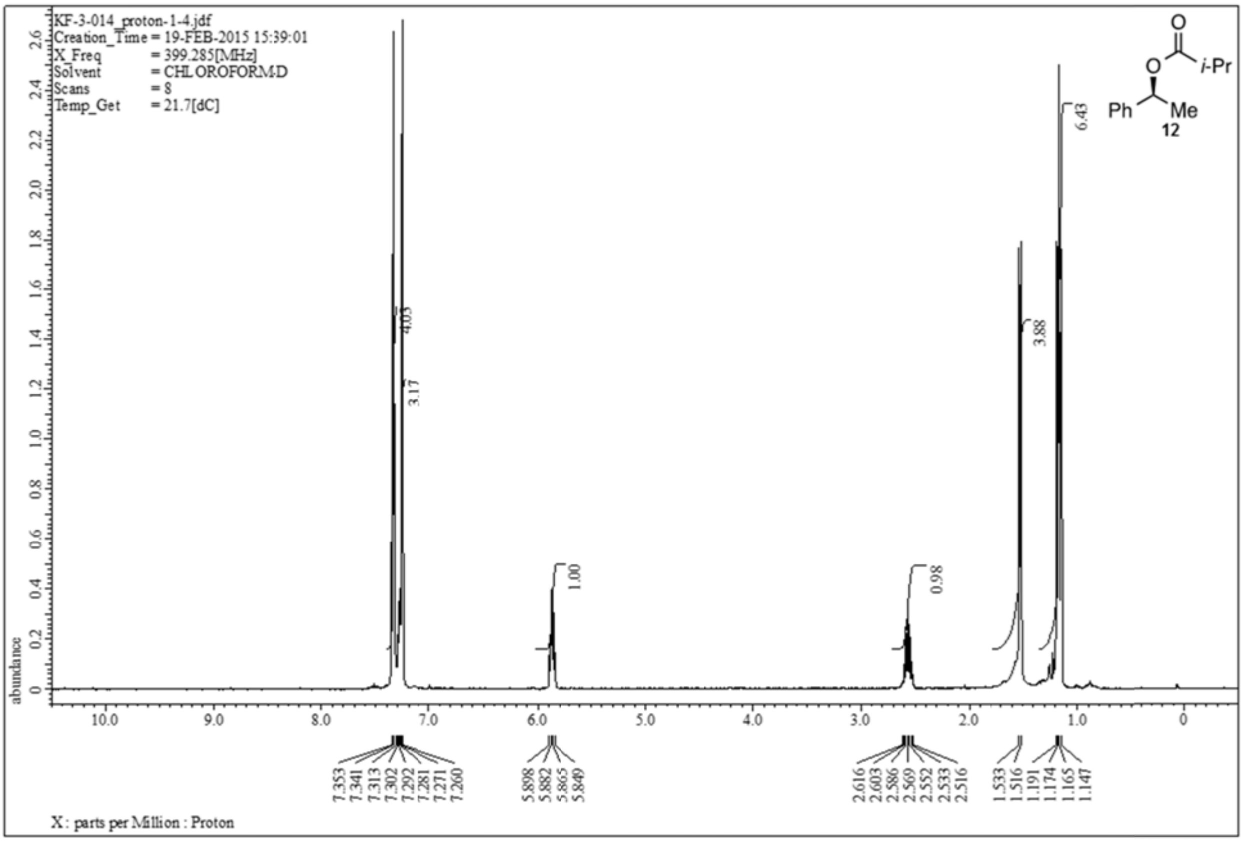

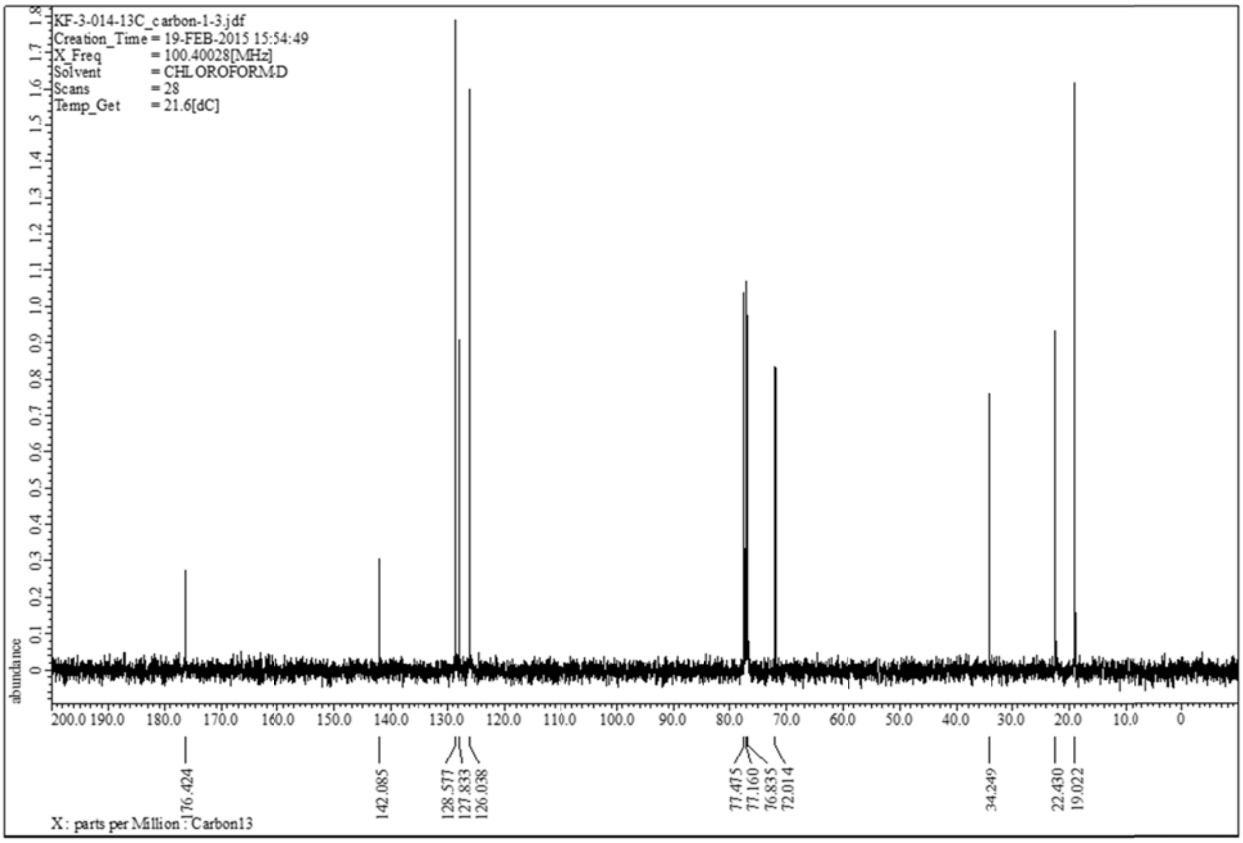

Supplementary Figure 70. ^{1}H and ^{13}C-NMR spectra for product **12**

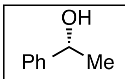

(*R*)-**11**

Racemic sample of **11**	(*R*)-**11**

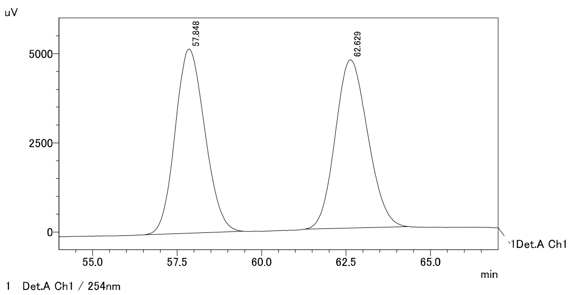

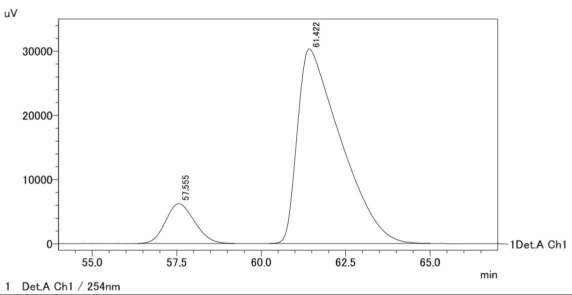

Peak #	Ret. Time	Area	Area %	Peak #	Ret. Time	Area	Area %
1	57.848	316410	50.055	1	57.555	369292	12.022
2	62.629	315721	49.945	2	61.422	2702792	87.974

Supplementary Figure 71. HPLC spectra for substrate **11** of kinetic resolution of secondary carbinol with catalyst **1j** (see Figure 9a)

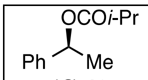

(*S*)-**12**

Racemic sample of **12**	(*R*)-**12**

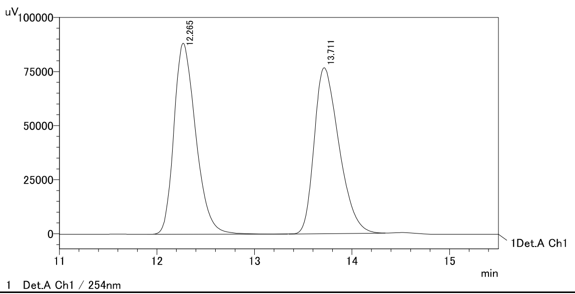

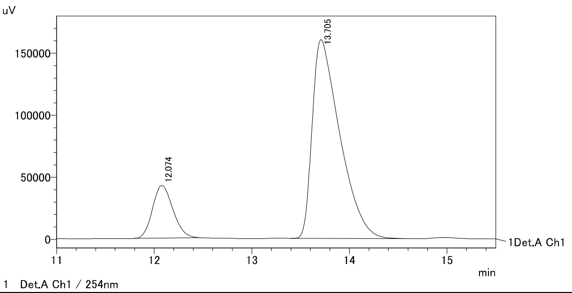

Peak #	Ret. Time	Area	Area %	Peak #	Ret. Time	Area	Area %
1	12.265	1376503	50.322	1	12.074	616839	16.386
2	13.711	1358904	49.678	2	13.705	3147745	83.614

Supplementary Figure 72. HPLC spectra for product **12** of kinetic resolution of secondary carbinol with catalyst **1j** (see Figure 9a)

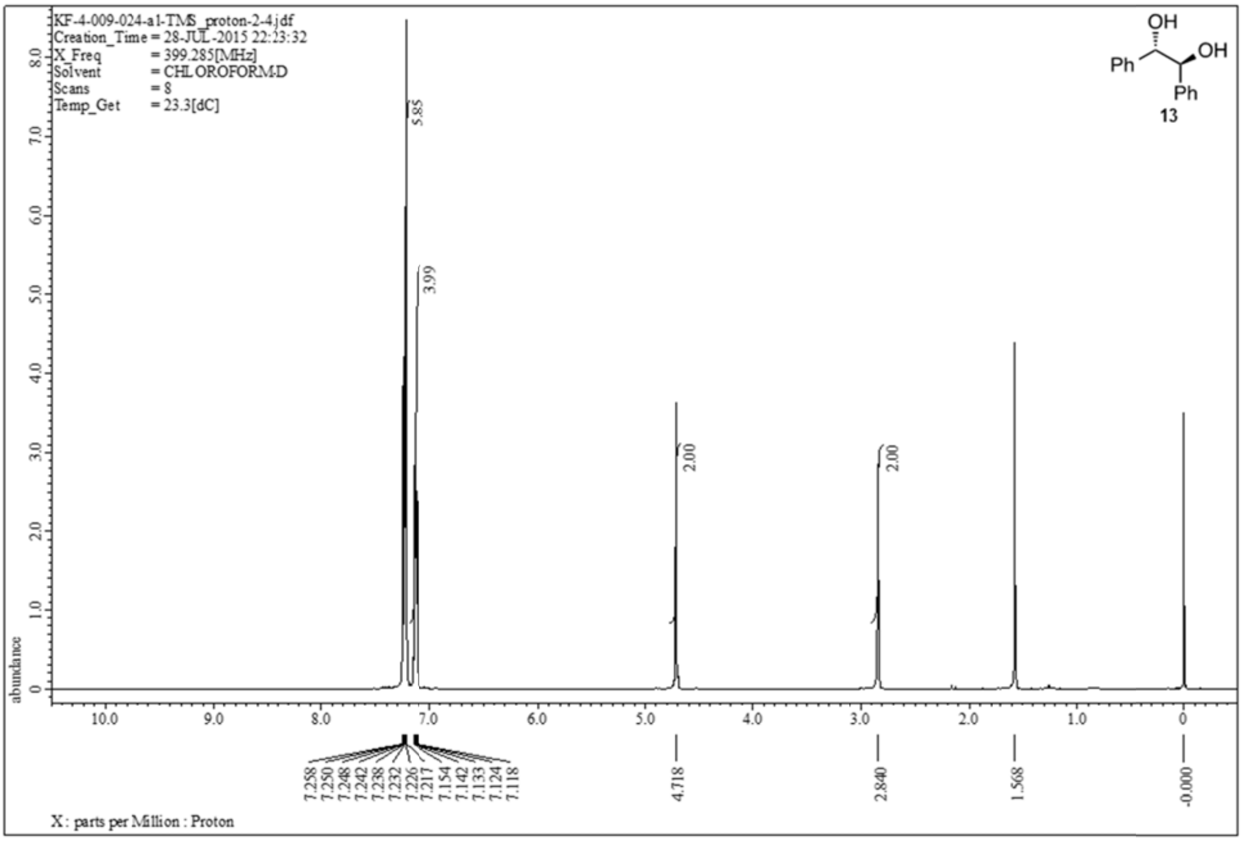

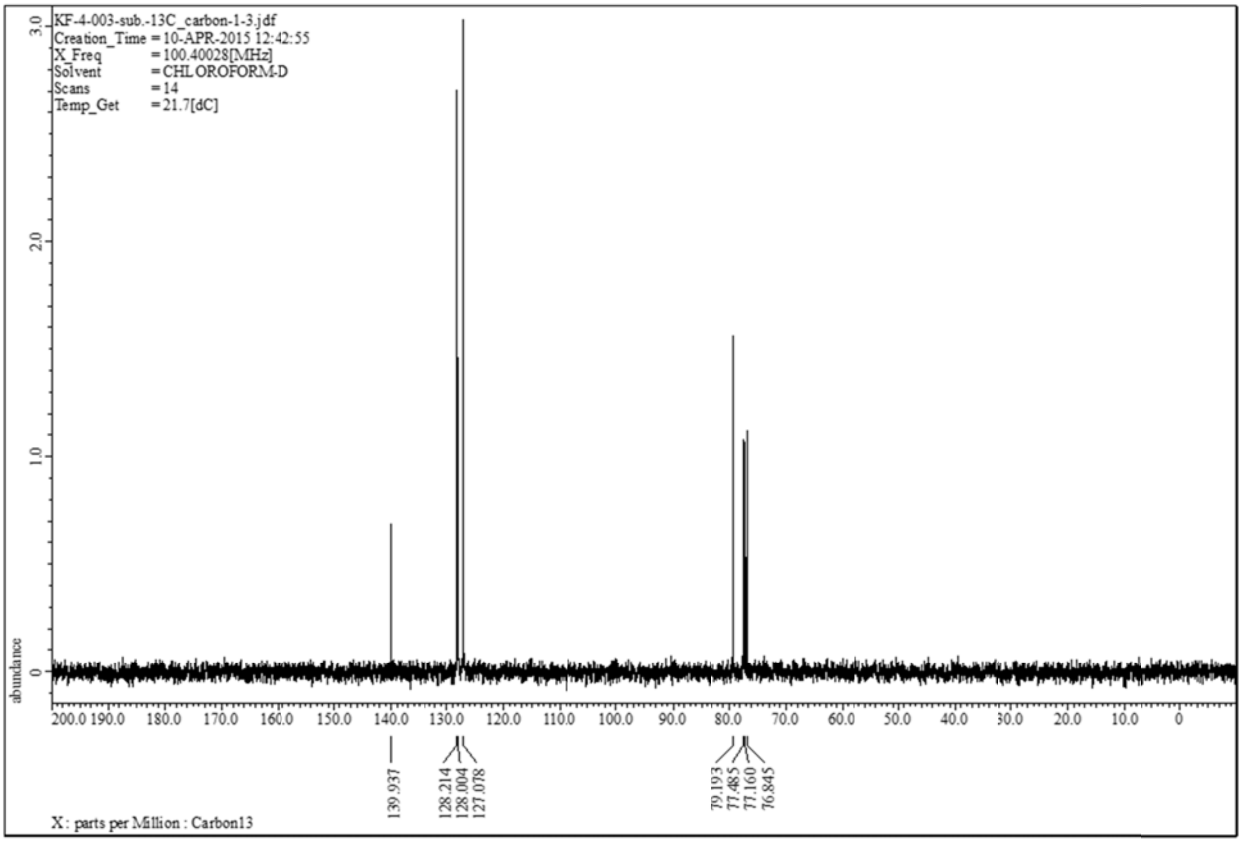

Supplementary Figure 73. ^{1}H and ^{13}C-NMR spectra for product **13**

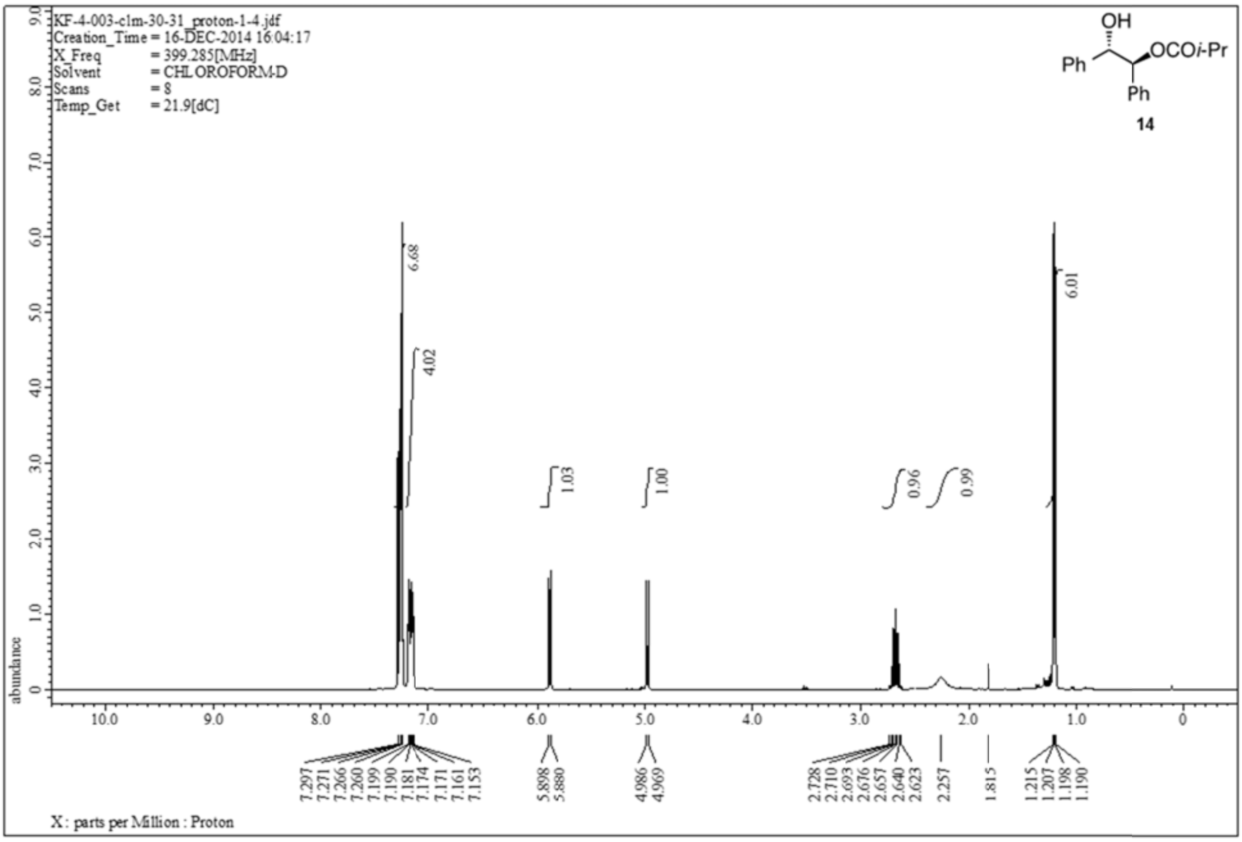

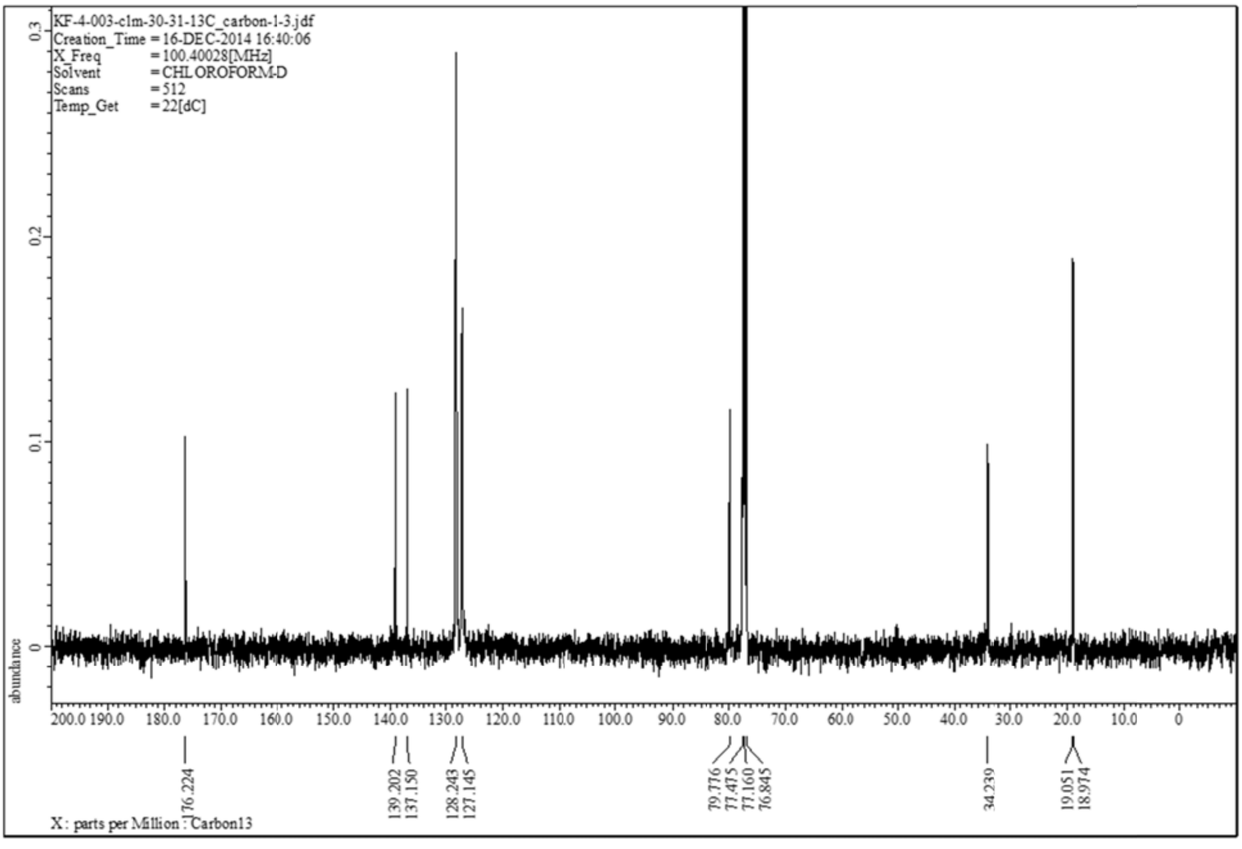

Supplementary Figure 74. ^{1}H and ^{13}C-NMR spectra for product **14**

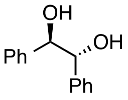

(1*R*, 2*R*)-**13**

Racemic sample of **13**

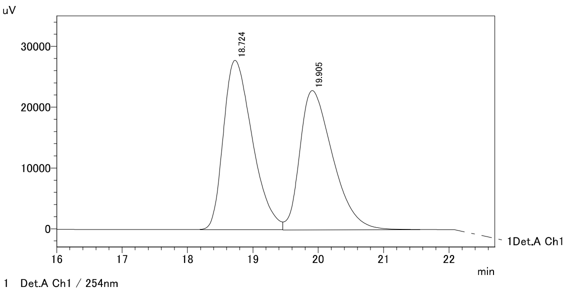

ピ ク ポ

Peak #	Ret. Time	Area	Area %
1	18.724	627240	49.798
2	19.905	632317	50.202

(1*S*, 2*R*)-**13**

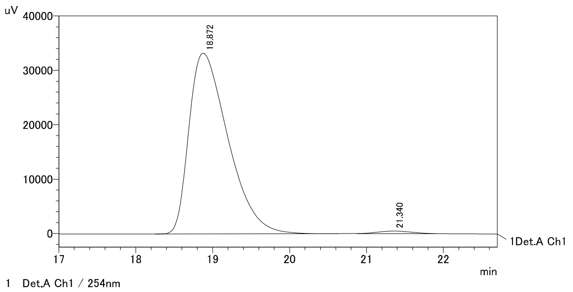

ピ ク ポ

Peak #	Ret. Time	Area	Area %
1	18.872	1172564	98.787
2	21.340	14395	1.213

Supplementary Figure 75. HPLC spectra for substrate **13** of kinetic resolution of racemic acyclic 1,2-diol with catalyst **1g** (see Figure 9b)

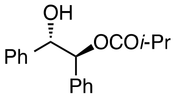

(1*S*, 2*S*)-**14**

Racemic sample of **14**

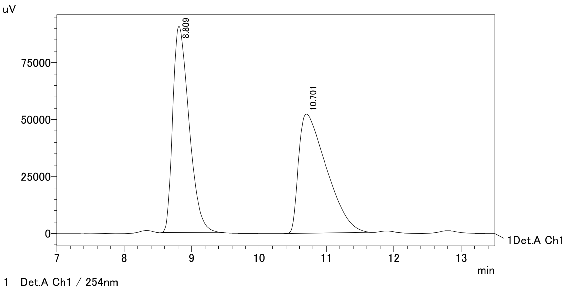

ピ ク ポ

Peak #	Ret. Time	Area	Area %
1	8.809	1527948	50.365
2	10.701	1505824	49.635

(1*S*, 2*R*)-**14**

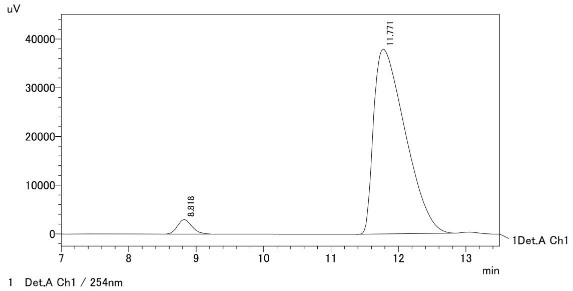

ピ ク ポ

Peak #	Ret. Time	Area	Area %
1	8.818	43462	3.406
2	11.771	1232480	96.594

Supplementary Figure 76. HPLC spectra for product **14** of kinetic resolution of racemic acyclic 1,2-diol with catalyst **1g** (see Figure 9b)

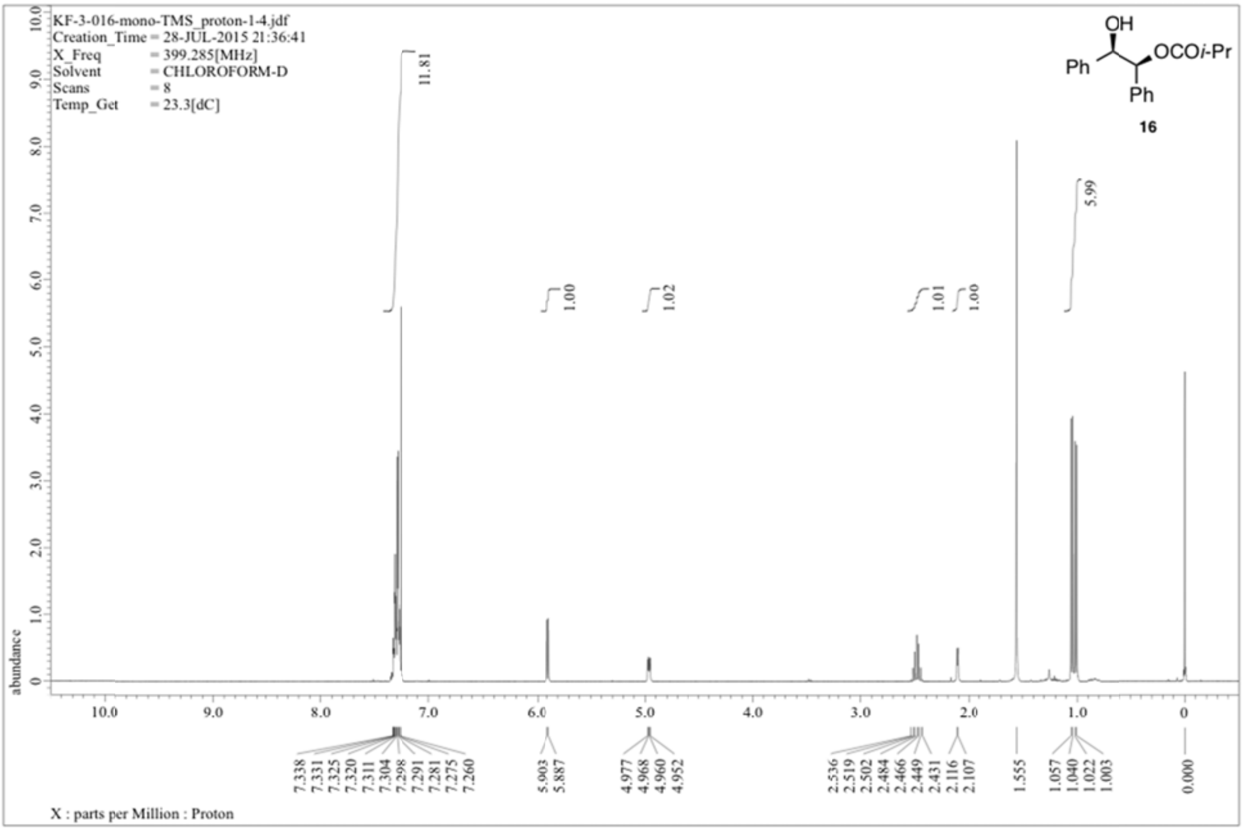

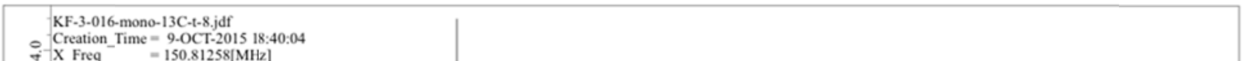

Supplementary Figure 77. ^{1}H and ^{13}C-NMR spectra for product **16**

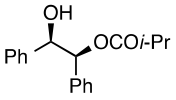

(1*S*, 2*R*)-**16**

Racemic sample of **16**

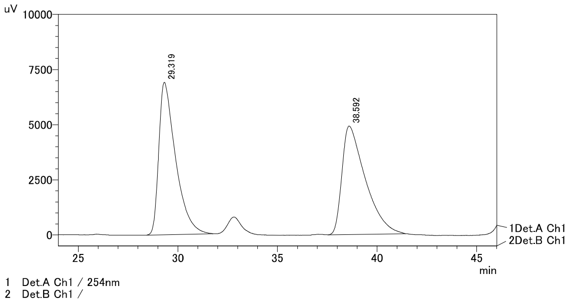

Peak #	Ret. Time	Area	Area %
1	29.319	405052	50.262
2	38.592	400827	49.738

(1*S*, 2*R*)-**16**

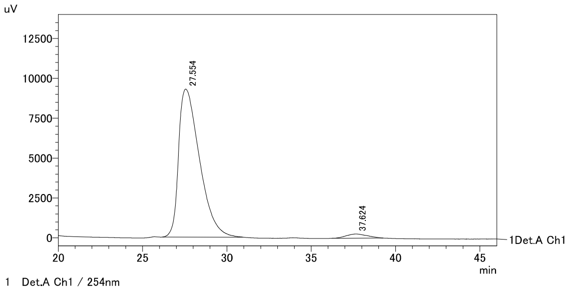

Peak #	Ret. Time	Area	Area %
1	27.554	800955	97.230
2	37.624	22819	2.770

Supplementary Figure 78. HPLC spectra for product **16** of kinetic resolution of desymmetrization of acyclic *meso*-1,2-diol with catalyst **1g** (see Figure 9c)

Supplementary Table 1. The screening of the solvent for the explore of optimal reaction conditions

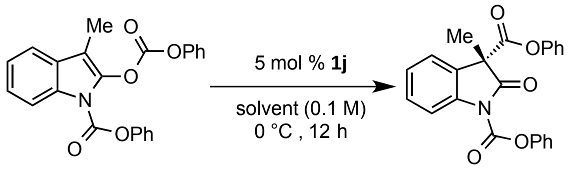

9a **10a**

Entry	Solvent	Conv (%)[b]	er[c]
1	THF	>98	98:2
2	Et_2O	>98	98:2
3	CPME	>98	97:3
4	acetone	>98	95:5
5	EtOAc	>98	98:2
6	CH_2Cl_2	>98	97:3
7	toluene	>98	98:2
8	hexane	74	93:7
9	*t*-amyl alcohol	88	64:36

[a]Reactions were performed on 0.10 mmol scale under Ar atmosphere. [b]Conversions were determined by ^{1}H NMR analysis of unpurified reaction mixture. [c]Enantioselectivities were determined by HPLC analysis.

We conducted several screening in order to explore the optimal reaction conditions. After screening of catalyst (Figure 4a), we carried out screening of solvent (Supplementary Table 1). Various solvents could be used in these reaction to afford the product **10a** with complete conversions (>98% conv) and a high enantioselectivity (>95:5 er, entries 1–7) except in the case of hexane and *t*-amyl alcohol (entries 8 and 9). In addition, the *t*-amyl alcohol significantly decreased the enantioselectivity (64:36 er) which might be stem largely from inhibition of hydrogen bonding network between the catalyst **1j** and substrate **9a** in the transition state. Since the highest enantiomeric ratio (98:2 er) was achieved in the reaction in THF (entry 1), we selected THF as an optimal solvent.

Supplementary Table 2. The screening of the reaction temperature for the explore of optimal reaction conditions

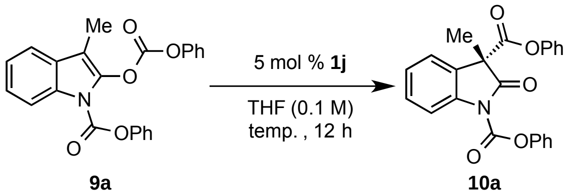

Entry	Temp. (°C)	Conv (%)[b]	er[c]
1	25	>98	96:4
2	0	>98	97:3
3	–20	>98	98:2
4	–40	>98	98:2
5	–60	97	99:1
6	–78	39	99:1

[a-c]See footnotes a-c of Supplementary Table 1.

Next, we also carried out the reaction at various temperature (Supplementary Table 2). The reaction at above –40 °C proceeded smoothly (>98% conv; >96:4 er; entries 1–4), whereas below –60 °C decreased the reaction conversions with maintaining almost the same enantiomeric ratios (99:1 er, entries 5 and 6). Interestingly, synthetically useful enantioselectivity (96:4 er) was also maintained even if the reaction at 25 °C. The catalyst **1j**, however, could be used and tolerated in various solvents and the reaction temperature to deliver the desired product **10a** with a high enantiomeric ratio. Judging from practicality and efficiency of the reaction, the reaction at –20 °C was determined to be optimal in the enantioselective Steglich rearrangement of oxindoles.

Supplementary Table 3. The screening of the migrating and *N*-protected group for the explore of optimal reaction conditions

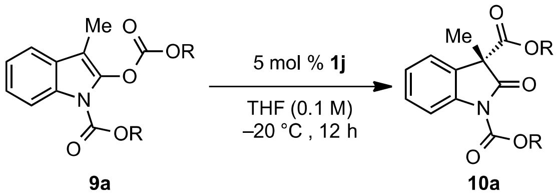

entry	Product (R =)	Conv (%)[b]	er[c]
1	**10a**: Ph	>98	98:2
2	**10n**: 4-MeO-C_6H_4	>98	98:2
3	**10o**: 4-F-C_6H_4	>98	96:4
4	**10p**: Bn	94	92:8
5	**10q**: Me	82	95:5
6	**10r**: *i*-Pr	<2	—

[a-c]See footnotes a-c of SupplementaryTable 1.

We carried out the reaction with different migrating group (Supplementary Table 3). As a result, phenyl carbonate gave the best enantioselectivity (98:2 er, entry 1). Therefore phenyl carbonate thought to be the

optimal migrating group, and was used for further screening. The procedures and the compounds data were shown in the later part of this supporting information.

Supplementary Table 4. The screening of the catalyst loading for the explore of optimal reaction conditions

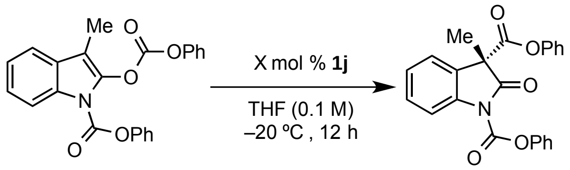

9a **10a**

Entry	X (mol %)	Conv (%)[b]	er[c]
1	5	>98	98:2
2	3	>98	98:2
3	1	>98	98:2
4	0.5	>98	98:2
5	0.3	73	98:2
6	0.1	15	98:2

[a-c]See footnotes a-c of Supplementaly Table 1.

We carried out the reaction in the presence of various catalyst loading (Supplementary Table 4). The reaction with at least 0.5 mol % of catalyst **1j** also proceeded smoothly, and the desired product **10a** was obtained in >98% conv with 98:2 er (entries 1–4). The use of less than 0.3 mol % of **1j** in the reaction decreased the conversion of **9a** after 12 h but still maintaining high enantioselectivities (entries 5 and 6).

Supplementary Table 5. The screening of the concentration of substrate for the explore of optimal reaction conditions

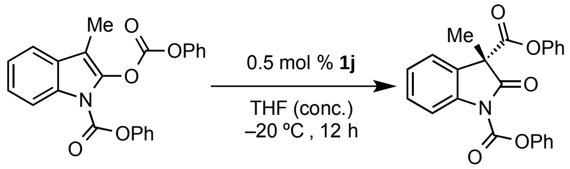

9a **10a**

Entry	Conc. (M)	Conv (%)[b]	er[c]
1	0.1	71	98:2
2	0.2	92	98:2
3	0.4	>98	98:2
4[d]	0.4	>98	98:2

[a-c]See footnotes a-c of Supplementary Table 1. [d]The reaction was carried out in THF (0.4 M) at -20 °C for 5 h.

Further tuning of the reaction conditions, concentration of substrate **9a** and reaction time, allowed to find the optimal reaction conditions to afford **10a** within 5 hours (>98% conv; 98:2 er; entry 4).

Supplementary Table 6. The time course analysis of the enantioselective Steglich rearrangement of oxindole catalyzed by **1j**, **1j´**and DMAP for kinetic experiments

catalyst **1j**

time (h)	0.33	0.67	1	2	3	4	5
conversion (%)	34.8	55.2	69.0	91.4	97.5	100	100
ln(1-conv/100)	0.428	0.803	1.17	2.45	3.69	–	–
kinetic constant (k, h^{-1})	1.22						

DMAP

time (h)	0.33	0.67	1	2	3	4	5
conversion (%)	0	1.7	2.2	4.4	6.8	9.4	13.5
ln(1-conv/100)	0	0.0171	0.0222	0.0450	0.0704	0.0987	0.1450
kinetic constant (k, h^{-1})	7.47×10^{-2}						

catalyst **1j´**

time (h)	0.33	0.67	1	2	3	4	5
conversion (%)	2.3	4.7	6.4	12.4	19.5	24.3	33.2
ln(1-conv/100)	0.0233	0.0481	0.0661	0.132	0.217	0.278	0.404
kinetic constant (k, h^{-1})	2.62×10^{-2}						

Supplementary Table 7. Cartesian coordinates of calculation results.

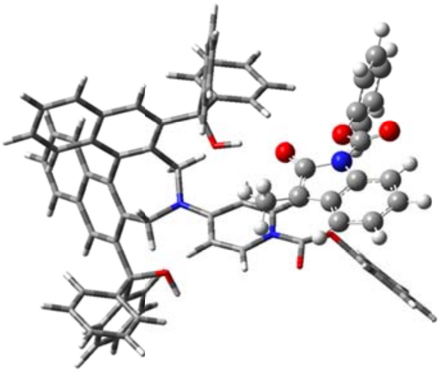

TS-I

Thermal Free Energies = -3620.205898 A.U.

Center Number	Atomic Number	Atomic Type	Coordinates (Angstroms) X	Y	Z
1	7	0	-1.720045	0.343494	0.023893
2	6	0	-1.762828	-0.955341	0.734540
3	6	0	-2.918775	0.673857	-0.771863
4	6	0	-2.259812	-2.047920	-0.205066
5	6	0	-4.182510	0.334396	0.001469
6	6	0	-3.619018	-2.036893	-0.514847
7	6	0	-4.155708	-2.950315	-1.486004
8	6	0	-3.283962	-3.938645	-2.035405

9	6	0	-1.918516	-3.953589	-1.652088
10	6	0	-1.379395	-3.023216	-0.792368
11	6	0	-5.007830	1.346704	0.600577
12	6	0	-6.132037	0.954306	1.293644
13	6	0	-6.456738	-0.409811	1.503448
14	6	0	-5.617977	-1.422350	0.946270
15	6	0	-4.500936	-1.015569	0.138708
16	6	0	-7.580393	-0.782656	2.288919
17	6	0	-7.851004	-2.106587	2.546908
18	6	0	-6.998030	-3.111689	2.033126
19	6	0	-5.911448	-2.780229	1.253935
20	6	0	-5.496453	-2.899554	-1.959542
21	6	0	-5.954125	-3.804341	-2.892335
22	6	0	-5.097866	-4.807982	-3.403042
23	6	0	-3.788625	-4.867886	-2.984193
24	6	0	-0.560336	1.019788	-0.146034
25	6	0	-0.351928	1.934196	-1.219325
26	6	0	0.893280	2.455344	-1.453329
27	6	0	1.762537	1.408683	0.456858
28	6	0	0.543309	0.860847	0.741510
29	7	0	1.955890	2.165065	-0.659320
30	6	0	3.314162	2.639603	-1.124288
31	8	0	3.329371	3.463748	-2.026598
32	8	0	4.121341	2.684357	0.011533
33	6	0	4.859289	3.837286	0.287697
34	6	0	5.725709	4.421463	-0.635805
35	6	0	4.746366	4.336108	1.585850
36	6	0	6.472228	5.533430	-0.242822
37	6	0	5.508453	5.440586	1.967381
38	6	0	6.370498	6.046411	1.051967
39	1	0	-0.770246	-1.186045	1.094583
40	1	0	-2.431170	-0.868982	1.596617
41	1	0	-2.889822	1.732144	-0.997288
42	1	0	-2.901067	0.116525	-1.717229
43	1	0	-1.281907	-4.713554	-2.090963
44	1	0	-6.788450	1.699287	1.729213
45	1	0	-8.213795	0.002004	2.695807
46	1	0	-8.708264	-2.382728	3.154705
47	1	0	-7.199785	-4.155244	2.258956
48	1	0	-5.263736	-3.561699	0.874345
49	1	0	-6.163153	-2.131502	-1.584613
50	1	0	-6.981167	-3.743900	-3.242335
51	1	0	-5.472763	-5.519244	-4.133942
52	1	0	-3.112796	-5.619684	-3.384597
53	1	0	-1.140634	2.178699	-1.917588
54	1	0	1.124187	3.106334	-2.286015
55	1	0	2.625404	1.268956	1.089190
56	1	0	0.456293	0.267219	1.640908
57	1	0	5.812636	4.010720	-1.632939
58	1	0	4.067119	3.853303	2.282003
59	1	0	7.148038	5.994398	-0.958290
60	1	0	5.423366	5.826961	2.979701
61	1	0	6.962813	6.908298	1.346808
62	6	0	-4.681925	2.850745	0.443288
63	6	0	0.137154	-3.035051	-0.470391
64	8	0	0.552649	-1.698475	-0.762849

65	1	0	1.527952	-1.595486	-0.701515
66	6	0	0.377411	-3.388870	1.018083
67	6	0	1.523617	-2.918866	1.678430
68	6	0	-0.507806	-4.216239	1.723759
69	6	0	1.764021	-3.259583	3.011034
70	1	0	2.241266	-2.289515	1.160912
71	6	0	-0.264447	-4.558803	3.055965
72	1	0	-1.397444	-4.596465	1.231007
73	6	0	0.871521	-4.078233	3.705912
74	1	0	2.656431	-2.884726	3.504130
75	1	0	-0.967794	-5.199260	3.582396
76	1	0	1.062561	-4.340273	4.743332
77	6	0	0.917475	-4.009789	-1.382912
78	6	0	1.291582	-3.579895	-2.664957
79	6	0	1.258925	-5.310206	-0.990762
80	6	0	1.991234	-4.423194	-3.526994
81	1	0	1.033582	-2.574111	-2.978058
82	6	0	1.957247	-6.156991	-1.855154
83	1	0	0.989703	-5.666903	-0.002120
84	6	0	2.328171	-5.717407	-3.125484
85	1	0	2.276827	-4.065917	-4.513118
86	1	0	2.217540	-7.160026	-1.526718
87	1	0	2.877686	-6.374119	-3.794835
88	8	0	-3.297283	2.984057	0.836658
89	1	0	-3.162785	3.906182	1.107999
90	6	0	-5.513903	3.733685	1.399067
91	6	0	-6.591767	4.520180	0.976062
92	6	0	-5.163214	3.755068	2.760290
93	6	0	-7.299264	5.308934	1.887287
94	1	0	-6.881234	4.526303	-0.068855
95	6	0	-5.867277	4.543907	3.668242
96	1	0	-4.335999	3.141379	3.103069
97	6	0	-6.939787	5.326351	3.234147
98	1	0	-8.129894	5.915510	1.536367
99	1	0	-5.578447	4.545512	4.715969
100	1	0	-7.488808	5.943490	3.940157
101	6	0	-4.862266	3.298379	-1.025182
102	6	0	-4.013415	4.265644	-1.579519
103	6	0	-5.894211	2.783492	-1.821819
104	6	0	-4.188670	4.706224	-2.892054
105	1	0	-3.195477	4.667567	-0.990127
106	6	0	-6.074144	3.225968	-3.133912
107	1	0	-6.558716	2.026877	-1.415963
108	6	0	-5.221579	4.188772	-3.675042
109	1	0	-3.514675	5.453886	-3.301833
110	1	0	-6.878848	2.809934	-3.734304
111	1	0	-5.357386	4.529152	-4.697845
112	6	0	6.231573	0.671518	-1.354183
113	6	0	3.998807	0.773183	-2.042728
114	6	0	4.087514	-0.141146	-0.926604
115	8	0	3.217014	-0.866012	-0.427390
116	6	0	2.963021	0.563573	-3.115403
117	1	0	2.052858	0.117192	-2.703517
118	1	0	3.335485	-0.105839	-3.902648
119	1	0	2.703025	1.516276	-3.592914
120	6	0	5.370069	1.157270	-2.358910

121	6	0	7.598155	0.922360	-1.366490
122	6	0	5.897773	1.905066	-3.412564
123	6	0	7.273410	2.159421	-3.443544
124	1	0	7.695969	2.734479	-4.263585
125	6	0	8.107814	1.678507	-2.430884
126	1	0	9.174517	1.882690	-2.466962
127	1	0	8.242576	0.534250	-0.589758
128	1	0	5.245467	2.286308	-4.192423
129	7	0	5.442785	-0.070256	-0.429172
130	6	0	5.950536	-0.589437	0.750324
131	8	0	7.128943	-0.725353	0.993852
132	8	0	4.949005	-0.883808	1.631841
133	6	0	5.290356	-1.517110	2.826422
134	6	0	4.893800	-0.896407	4.008430
135	6	0	5.913704	-2.764420	2.838239
136	6	0	5.124337	-1.537497	5.227336
137	1	0	4.413514	0.075847	3.961206
138	6	0	6.144578	-3.391492	4.062151
139	1	0	6.214588	-3.228634	1.905940
140	6	0	5.750966	-2.784418	5.257522
141	1	0	4.816506	-1.056971	6.152002
142	1	0	6.632577	-4.362064	4.079172
143	1	0	5.933011	-3.280781	6.206525

Supplementary Table 8. Cartesian coordinates of calculation results.

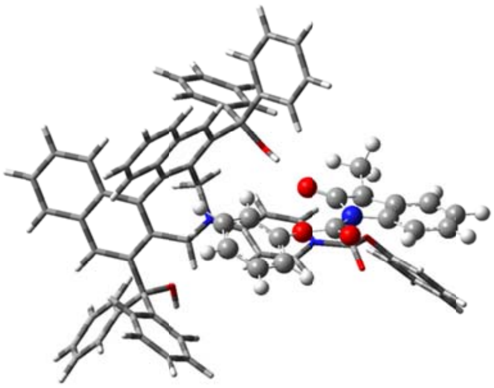

TS-II

Thermal Free Energies = -3620.200904 A.U.

Center Number	Atomic Number	Atomic Type	Coordinates (Angstroms) X	Y	Z
1	7	0	-1.220106	-0.483495	-0.893117
2	6	0	-1.586879	0.750048	-1.633615
3	6	0	-2.094750	-0.856158	0.238383
4	6	0	-1.785909	1.893306	-0.640459
5	6	0	-3.551742	-0.573070	-0.070077
6	6	0	-2.905229	1.816430	0.188160
7	6	0	-3.052879	2.706800	1.305972
8	6	0	-2.080579	3.736826	1.481839

9	6	0	-1.022857	3.860020	0.544402
10	6	0	-0.842046	2.974046	-0.496043
11	6	0	-4.501600	-1.614776	-0.344156
12	6	0	-5.809612	-1.260539	-0.592602
13	6	0	-6.232349	0.091662	-0.653988
14	6	0	-5.282965	1.133911	-0.424205
15	6	0	-3.937495	0.763614	-0.083377
16	6	0	-7.575806	0.424981	-0.973942
17	6	0	-7.966724	1.738694	-1.092221
18	6	0	-7.021078	2.773803	-0.902305
19	6	0	-5.714709	2.480934	-0.577666
20	6	0	-4.085566	2.583156	2.276656
21	6	0	-4.159458	3.449003	3.346512
22	6	0	-3.208982	4.485873	3.502092
23	6	0	-2.189837	4.622793	2.586868
24	6	0	0.042083	-0.986321	-0.934006
25	6	0	0.610317	-1.691378	0.160373
26	6	0	1.944893	-2.008124	0.156977
27	6	0	2.196156	-1.213074	-2.028718
28	6	0	0.881168	-0.842317	-2.076836
29	7	0	2.741596	-1.745174	-0.903246
30	6	0	4.244709	-1.934278	-0.693130
31	8	0	4.567868	-2.596883	0.281431
32	8	0	4.775579	-2.098536	-1.980284
33	6	0	5.660624	-3.134847	-2.263658
34	6	0	6.678388	-3.554394	-1.403751
35	6	0	5.517568	-3.701091	-3.532883
36	6	0	7.542028	-4.564931	-1.831352
37	6	0	6.396252	-4.700845	-3.948040
38	6	0	7.410883	-5.140873	-3.096177
39	1	0	-0.795409	0.980226	-2.333684
40	1	0	-2.504393	0.562994	-2.197363
41	1	0	-1.945793	-1.912994	0.427612
42	1	0	-1.798893	-0.299875	1.138995
43	1	0	-0.335839	4.688583	0.671745
44	1	0	-6.553341	-2.027684	-0.777306
45	1	0	-8.286887	-0.381441	-1.136678
46	1	0	-8.995070	1.984221	-1.342739
47	1	0	-7.327753	3.809877	-1.017377
48	1	0	-5.001591	3.285613	-0.441448
49	1	0	-4.815924	1.787974	2.177636
50	1	0	-4.953326	3.332647	4.079450
51	1	0	-3.281654	5.164909	4.347141
52	1	0	-1.444779	5.406311	2.700482
53	1	0	0.050230	-1.888656	1.063320
54	1	0	2.456729	-2.445683	1.004026
55	1	0	2.867276	-1.098749	-2.866856
56	1	0	0.505206	-0.410911	-2.993769
57	1	0	6.785334	-3.102590	-0.427794
58	1	0	4.719507	-3.349935	-4.180186
59	1	0	8.332241	-4.896838	-1.162945
60	1	0	6.282419	-5.136496	-4.937319
61	1	0	8.094636	-5.922252	-3.416172
62	6	0	5.819617	-0.133256	1.684825
63	6	0	4.828549	0.129234	-0.418208
64	6	0	3.854954	0.488212	0.598499

65	8	0	2.724823	0.973263	0.472191
66	6	0	4.787678	0.850208	-1.745394
67	1	0	3.760425	1.108838	-2.019449
68	1	0	5.225543	0.245346	-2.545354
69	1	0	5.352682	1.791439	-1.695339
70	6	0	6.083460	-0.091388	0.301639
71	6	0	6.815267	-0.373368	2.623620
72	6	0	7.388952	-0.282486	-0.150165
73	6	0	8.405179	-0.520392	0.781140
74	1	0	9.426546	-0.663328	0.437941
75	6	0	8.117750	-0.571662	2.147810
76	1	0	8.916476	-0.755930	2.861286
77	1	0	6.591793	-0.386305	3.681694
78	1	0	7.612182	-0.247883	-1.212948
79	7	0	4.434417	0.142569	1.872596
80	6	0	3.804215	0.120749	3.110034
81	8	0	4.378288	0.141298	4.176843
82	8	0	2.458326	0.024026	2.947192
83	6	0	1.582161	0.224908	4.012912
84	6	0	0.491436	1.048664	3.733987
85	6	0	1.716570	-0.414176	5.245012
86	6	0	-0.485823	1.241441	4.710178
87	1	0	0.433163	1.526336	2.761176
88	6	0	0.732180	-0.206607	6.213866
89	1	0	2.574796	-1.043331	5.441638
90	6	0	-0.366334	0.614665	5.953070
91	1	0	-1.334989	1.885215	4.497909
92	1	0	0.829470	-0.694692	7.180006
93	1	0	-1.125194	0.766975	6.715740
94	6	0	-4.093939	-3.106328	-0.319547
95	6	0	0.306560	3.195804	-1.519268
96	8	0	1.106515	2.015216	-1.619260
97	1	0	1.574385	1.798513	-0.785268
98	6	0	-0.260247	3.404823	-2.940875
99	6	0	0.547533	3.095688	-4.045168
100	6	0	-1.532353	3.942628	-3.172846
101	6	0	0.088710	3.307036	-5.344657
102	1	0	1.536869	2.687589	-3.872792
103	6	0	-1.990935	4.158310	-4.475464
104	1	0	-2.173973	4.193524	-2.334086
105	6	0	-1.183978	3.838568	-5.566400
106	1	0	0.729463	3.059092	-6.187171
107	1	0	-2.982950	4.574026	-4.632355
108	1	0	-1.541241	4.002683	-6.579602
109	6	0	1.191524	4.414115	-1.144154
110	6	0	2.304580	4.247812	-0.306987
111	6	0	0.897654	5.706022	-1.603830
112	6	0	3.098374	5.338346	0.052606
113	1	0	2.567426	3.268805	0.079128
114	6	0	1.690051	6.796964	-1.242593
115	1	0	0.047156	5.866526	-2.257907
116	6	0	2.797077	6.618146	-0.413386
117	1	0	3.957577	5.180813	0.699410
118	1	0	1.441702	7.786281	-1.618525
119	1	0	3.418712	7.465142	-0.135212
120	8	0	-2.918582	-3.194200	-1.155732

121	1	0	-2.853174	-4.112717	-1.462494
122	6	0	-5.168570	-4.017594	-0.952340
123	6	0	-5.994505	-4.867455	-0.207559
124	6	0	-5.309265	-4.004300	-2.351262
125	6	0	-6.935219	-5.683159	-0.842570
126	1	0	-5.903482	-4.902520	0.872335
127	6	0	-6.245834	-4.819120	-2.984138
128	1	0	-4.679073	-3.344725	-2.939641
129	6	0	-7.063856	-5.664287	-2.230457
130	1	0	-7.562792	-6.339272	-0.245314
131	1	0	-6.337222	-4.792913	-4.066791
132	1	0	-7.793311	-6.302205	-2.722113
133	6	0	-3.758347	-3.552192	1.121455
134	6	0	-2.720572	-4.462989	1.358919
135	6	0	-4.500163	-3.088273	2.216753
136	6	0	-2.430625	-4.898065	2.652908
137	1	0	-2.116713	-4.821460	0.531468
138	6	0	-4.215220	-3.526216	3.511318
139	1	0	-5.302511	-2.374086	2.057821
140	6	0	-3.178460	-4.432774	3.735185
141	1	0	-1.615790	-5.599053	2.813296
142	1	0	-4.800795	-3.149684	4.345822
143	1	0	-2.950808	-4.767897	4.743242

All calculations were performed with the Gaussian 03 program[1]. Geometries were fully optimized and characterized by frequency calculation using the the B3LYP density functional theory (DFT)[2, 3] with the 6-31G(d) basis set. Free energies (298.15 K, 1 atm) were initially computed for the gas phase. Weak hydrogen bond (between the benzene ring of **I** and the enolate **II**), C-H···O. However, the interaction of **TS-I** is stronger than that of **TS-II** (2.5 kcal/mol vs. 1.0 kcal/mol), which were evaluated by second order perturbation theory analysis in NBO at the M062X/6-31g** level.

Supplementary Methods

General

Nuclear Magnetic Resonance (NMR) spectra were recorded on Varian 600 MR (^{1}H 600 MHz, ^{13}C 150 MHz), Varian 400 MR (^{1}H 400 MHz, ^{13}C 100 Mz) and JEOL ECS-400 (^{1}H 400 MHz, ^{13}C 100 Mz) spectrometer. Chemical shifts for ^{1}H NMR were reported in parts per million (ppm) relative to residual $CHCl_3$ in $CDCl_3$ (δ 7.26 ppm). Data is reported as follows: chemical shift, multiplicity (s = singlet, d = doublet, t = triplet, q = quartet, quin =quintet, sext = sextet, sep = septet, b = broad, m = multiplet), coupling constants, and integration. Chemical shifts for ^{13}C NMR were reported in parts per million (ppm) relative to $CDCl_3$ (δ 77.16 ppm) with complete proton decoupling. Infrared (IR) spectra were recorded on JASCO FT/IR-4100 spectrophotometer and Varian 7000e FT-IR spectrometer, Vmax in cm^{-1}. High-resolution mass spectrometry was performed on a JEOL JMS-700 MStation (FAB-MS and EI-MS), Agilent 6520 Accurate Mass Q-TOF LC/MS (ESI-MS) and Thermo Fisher Scientific EXACTIVE Plus (ESI-MS). Optical rotations were measured on a JASCO DIP-1000. Melting point was recorded on a SANSYO SMP-300. Enantiomeric ratios were determined by analytical liquid chromatography (HPLC) Shimadzu chromatograph (DAICEL CHIRALPAK® AS-3 (4.6 × 150 mm), DAICEL CHIRALPAK® AD-H (4.6 × 250 mm) in comparison with authentic racemic materials. Column chromatography was performed with silica gel 60 N (spherical, neutral, 40–50 μm) purchased from Kanto Chemical. All experiments were carried out under argon atmosphere unless otherwise noted.

Materials

All reagents were obtained commerically available and used as received unless otherwise noted. MeCN, CH_2Cl_2, toluene, and Et_3N were distilled over CaH_2. Dry tetrahydrofuran [THF] and dry Et_2O were purchased from Wako Pure Chemical Industries, Ltd. and used without further purification. Dry dimethylformamide [DMF] and 3-methyl-2-oxindole were purchased from Sigma-ALDRICH Japan and used without further purification. (*S*)-(-)-1,1′-Bi-2-naphthol [(*S*)-BINOL] (>99% ee) was purchased from KANTO CHEMICAL CO.,INC. and used without further purification. (±)-1-Phenylethanol **11** was purchased from NAKARAI TESQUE, INC. and used without further purification. (±)-Hydrobenzoin **5** was purchased from Tokyo Chemical Industry Co., Ltd. and used without further purification. **18**[4], **19**[5], **23**[5], **24**[5], **3–4**[6], **6–7**[6], **25**[7], **30**[6], **31**[8], **9a**[9], **9b**[10], **9d–i**[10], **9j**[9, 10], **9k–l**[10], **9m**[9], **9n–o**[10], **9p**[10, 11] and **15**[12] were prepared by reported procedures. All of new compounds were descried spectral data at following. **2**, **3**[13], **5**[6] was synthesized by modified known method.

Synthesis of catalyst 1a–1p

Synthesis of Catalyst 1a

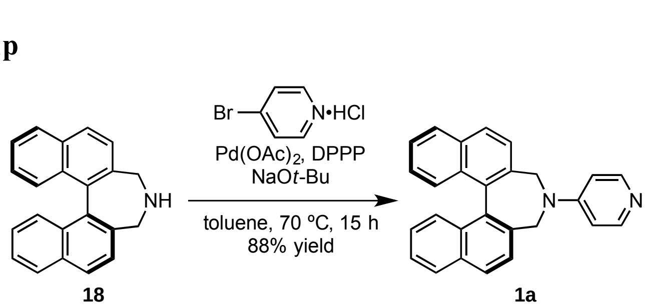

(*S*)-4-(pyridin-4-yl)-4,5-dihydro-3*H*-dinaphtho[2,1-*c*:1′,2′-*e*]azepine (1a)

To a suspension of **18**[4] (52.4 mg, 0.177 mmol), 4-bromopyridine hydrochloride (69.0 mg, 0.355 mmol), NaO*t*-Bu (68.0 mg, 0.708 mmol) and 1,3-bis(diphenylphosphino)propane [DPPP] (2.90 mg, 7.03 μmol) in toluene (1.61 mL) was added $Pd(OAc)_2$ (1.60 mg, 7.13 μmol) at room temperature. The reaction mixture was

warmed up to 70 °C and stirred for 15 h. After being cooled to room temperature, saturated aqueous $NaHCO_3$ (30 mL) was added to reaction mixture and extracted with toluene (10 mL × 2). The organic layer was washed with saturated aqueous $NaHCO_3$ (5 mL × 2), dried over $MgSO_4$, and concentrated *in vacuo*. The purification of the crude product by flash column chromatography on silica gel (eluent: CH_2Cl_2/MeOH = 5/1 to 2/1, v/v) gave **1a** (58.0 mg, 0.156 mmol, 88% yield) as a colorless solid.

^{1}H NMR (600 MHz, $CDCl_3$) δ 8.30 (d, *J* = 6.6 Hz, 2H), 7.96 (d, *J* = 8.4 Hz, 4H), 7.57–7.47 (m, 6H), 7.32 (td, *J* = 7.8, 1.4 Hz, 2H), 6.80 (d, *J* = 6.6 Hz, 2H), 4.67 (d, *J* = 12.6 Hz, 2H), 3.89 (d, *J* = 12.6 Hz, 2H); ^{13}C NMR (150 MHz, $CDCl_3$) δ 153.5, 150.3, 135.0, 133.5, 132.9, 131.5, 129.4, 128.5, 127.6, 127.4, 126.3, 126.1, 108.6, 50.7; IR (KBr) 1593, 1505, 1232, 827, 760 cm^{-1}; HRMS (FAB$^+$) calculated for $C_{27}H_{21}N_2$ [M+H]$^+$ 373.1699, found 373.1693; mp 261.0–261.7 °C; $[\alpha]^{22}_D$ –439.8 (*c* 1.15, $CHCl_3$, (*S*)-configuration).

An overview for synthesis of catalyst 1b

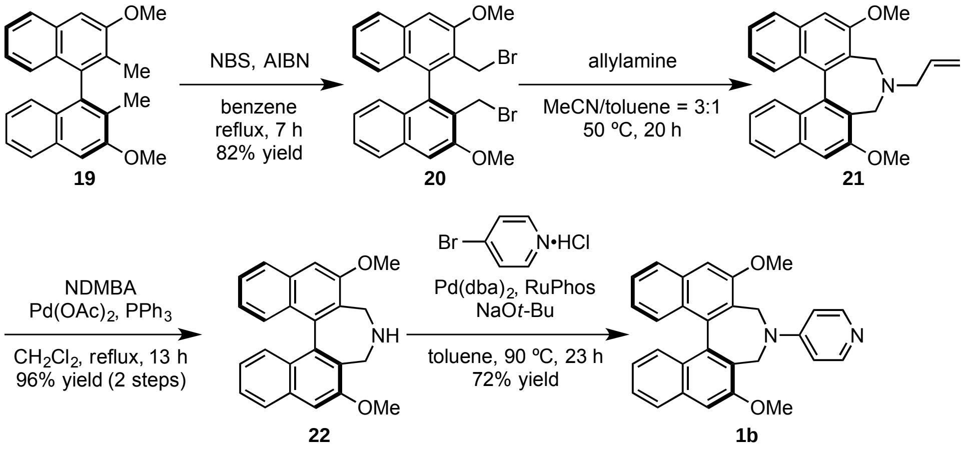

Synthesis of 20

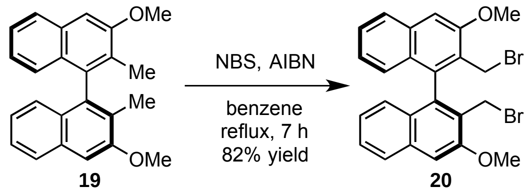

(*S*)-2,2′-bis(bromomethyl)-3,3′-dimethoxy-1,1′-binaphthalene (20)

To a solution of **19**[5] (6.19 g, 18.1 mmol) and *N*-bromosuccinimide [NBS] (7.08 g, 39.8 mmol) in benzene (90.5 mL) was added azobisisobutyronitrile [AIBN] (297 mg, 1.81 mmol) at room temperature. The reaction mixture was warmed up to refluxed temperature and stirred for 7 h. After being cooled to room temperature, water (30 mL) was added to reaction mixture. The resulting solution was extracted with toluene (30 mL × 2), and the organic layer was washed with saturated aqueous $NaHCO_3$ (20 mL × 2), and brine (20 mL × 2), dried over $MgSO_4$ and concentrated *in vacuo*. The purification of the crude product by recrystallizition from CH_2Cl_2/hexane gave **20** (7.39 g, 14.8 mmol, 82% yield) as a colorless solid.

^{1}H NMR (400 MHz, $CDCl_3$) δ 7.81 (d, *J* = 8.2 Hz, 2H), 7.44 (ddd, *J* = 8.2, 6.9, 1.5 Hz, 2H), 7.33 (s, 2H), 7.11 (ddd, *J* = 8.2, 6.9, 1.5 Hz, 2H), 6.99 (bd, *J* = 8.2 Hz, 2H), 4.34 (d, *J* = 9.4 Hz, 2H), 4.28 (d, *J* = 9.4 Hz, 2H), 4.11 (s, 6H); ^{13}C NMR (100 MHz, $CDCl_3$) δ 155.7, 136.5, 134.8, 127.8, 127.4, 127.4, 126.8, 124.3, 106.5, 56.0, 27.9; IR (KBr) 3060, 2935, 1599, 1170, 750 cm^{-1}; HRMS (FAB$^+$) [M+H]$^+$ calculated for $C_{24}H_{21}Br_2O_2$ 500.9883, found 500.9875; mp 189.9–190.5 °C; $[\alpha]^{20}_D$ +163.5 (*c* 0.22, $CHCl_3$, (*S*)-configuration).

Synthesis of 21

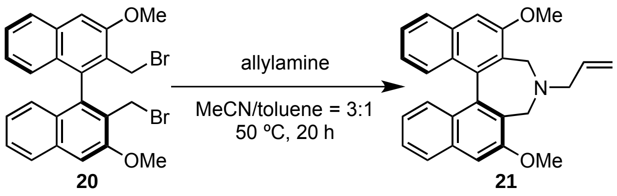

(*S*)-4-allyl-2,2'-dimethoxy-4,5-dihydro-3H-dinaphtho[2,1-c:1′,2′-e]azepine (21)

To a solution of **20** (1.58 g, 3.16 mmol) in MeCN (31.6 mL) and toluene (10.5 mL) was added allylamine (781 µL, 10.4 mmol) at room temperature. The reaction mixture was warmed up to 50 °C and stirred for 20 h. After being cooled to room temperature, water (50 mL) was added to reaction mixture. The resulting solution was extracted with EtOAc (50 mL × 2), and the organic layer was washed with water (30 mL × 2) and brine (10 mL × 2), dried over $MgSO_4$ and concentrated *in vacuo*. The crude product was directly used for the following reaction without further purification. The analytical data of **21** was obtained by the purification of the crude product by flash column chromatography on silica gel (eluent: hexane/EtOAc = 3/1 to 2/1, v/v) as a colorless solid.

^{1}H NMR (400 MHz, $CDCl_3$) δ 7.83 (d, *J* = 8.3 Hz, 2H), 7.41 (ddd, *J* = 8.3, 6.8, 1.3 Hz, 2H), 7.34 (d, *J* = 8.3 Hz, 2H), 7.28 (s, 2H), 7.10 (ddd, *J* = 8.3, 6.8, 1.3 Hz, 2H), 6.08–5.98 (m, 1H), 5.23–5.14 (m, 2H), 4.36 (d, *J* = 12.4 Hz, 2H), 4.02 (s, 6H), 3.25 (dd, *J* = 13.2, 6.7 Hz, 1H), 2.98 (dd, *J* = 13.2, 6.7 Hz, 1H), 2.78 (d, *J* = 12.4 Hz, 2H); ^{13}C NMR (100 MHz, $CDCl_3$) δ 155.5, 137.1, 136.6, 134.2, 127.7, 127.2, 127.0, 126.3, 126.1, 123.5, 117.5, 105.6, 59.2, 55.6, 46.7; IR (KBr) 2950, 2827, 1596, 1226, 752 cm^{-1}; HRMS (FAB^+) calculated for $C_{27}H_{26}NO_2$ $[M+H]^+$ 396.1958, found 396.1961; mp 158.0–158.7 °C; $[\alpha]^{20}_D$ +438.8 (*c* 0.23, $CHCl_3$, (*S*)-configuration).

Synthesis of 22

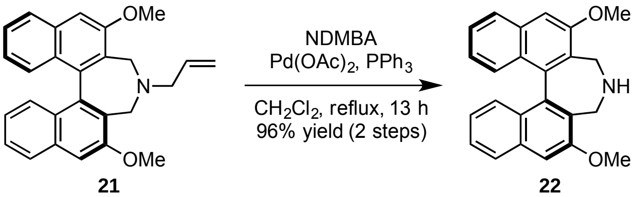

(*S*)-2,2'-dimethoxy-4,5-dihydro-3H-dinaphtho[2,1-c:1′,2′-e]azepine (22)

To a solution of **21** (1.21 g, 3.06 mmol), *N*,*N′*-dimethylbarbituric acid [NDMBA] (1.43 g, 9.15 mmol), PPh_3 (160 mg, 0.608 mmol) in CH_2Cl_2 (30.4 mL) was added $Pd(OAc)_2$ (34.5 mg, 0.153 mmol) at room temperature. The reaction mixture was warmed up to refluxed temperature and stirred for 13 h. After being cooled to room temperature, saturated aqueous $NaHCO_3$ (30 mL) was added to reaction mixture. The resulting solution was extracted with EtOAc (30 mL × 2) and the organic layer was washed with saturated aqueous $NaHCO_3$ (10 mL × 2) and brine (10 mL × 2), dried over $MgSO_4$ and concentrated *in vacuo*. The purification of the crude product by flash column chromatography on silica gel (eluent: EtOAc to EtOAc + 1% Et_3N) gave **22** (1.08 g, 3.04 mmol, 96% yield based on **20**) as a pale yellow solid.

^{1}H NMR (400 MHz, $CDCl_3$) δ 7.83 (d, *J* = 8.3 Hz, 2H), 7.41 (ddd, *J* = 8.3, 6.8, 1.3 Hz, 2H), 7.34 (d, *J* = 8.3 Hz, 2H), 7.29 (s, 2H), 7.11 (ddd, *J* = 8.3, 6.8, 1.3 Hz, 2H), 4.47 (d, *J* = 12.0 Hz, 2H), 4.03 (s, 6H), 3.04 (d, *J* = 12.0 Hz, 2H), 1.69 (bs, 1H); ^{13}C NMR (100 MHz, $CDCl_3$) δ 155.0, 137.0, 134.1, 127.6, 127.4, 127.2, 127.0, 126.0, 123.5, 106.0, 55.6, 40.1; IR (KBr) 2934, 1595, 1231, 832, 748 cm^{-1}; HRMS (FAB^+) $[M+H]^+$ calculated for $C_{24}H_{22}NO_2$ 356.1645, found 356.1655; mp 101.8–102.4 °C; $[\alpha]^{20}_D$ +388.5 (*c* 0.20, $CHCl_3$, (*S*)-configuration).

Synthesis of 1b

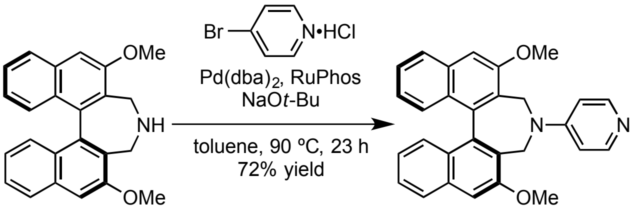

(*S*)-2,6-dimethoxy-4-(pyridin-4-yl)-4,5-dihydro-3H-dinaphtho[2,1-c:1′,2′-e]azepine (1b)

To a solution of **22** (733 mg, 2.06 mmol), 4-bromopyridine hydrochloride (801 mg, 4.12 mmol), NaO*t*-Bu (1.19 g, 12.4 mmol), 2-dicyclohexylphosphino-2′,6′-di-i-propoxy-1,1'-biphenyl [RuPhos] (77.2 mg, 0.165 mmol) in toluene (20.6 mL) was added bis(dibenzylideneacetone)palladium(0) [Pd(dba)$_2$] (47.4 mg, 82.4 μmol) at room temperature. The reaction mixture was warmed up to 90 °C and stirred for 23 h. After being cooled to room temperature, water (20 mL) was added to reaction mixture, and passed through a short pad of celite. The resulting solution was extracted with toluene (30 mL × 2), and the organic layer was washed with brine (10 mL × 2), dried over $MgSO_4$ and concentrated *in vacuo*. The purification of the crude product by flash column chromatography on silica gel (eluent: EtOAc to EtOAc + 2% Et_3N) gave **1b** (639 mg, 1.48 mmol, 72% yield) as a pale yellow solid.

^{1}H NMR (400 MHz, $CDCl_3$) δ 8.25 (d, *J* = 6.6 Hz, 2H), 7.83 (d, *J* = 8.0 Hz, 2H), 7.44 (t, *J* = 8.0 Hz, 2H), 7.38 (d, *J* = 8.0 Hz, 2H), 7.28 (s, 2H), 7.15 (t, *J* = 8.0 Hz, 2H), 6.85 (d, *J* = 6.6 Hz, 2H), 5.38 (d, *J* = 12.6 Hz, 2H), 3.96 (s, 6H), 3.36 (d, *J* = 12.6 Hz, 2H); ^{13}C NMR (100 MHz, $CDCl_3$) δ 154.7, 154.0, 149.7, 137.2, 134.3, 127.7, 127.3, 126.9, 126.6, 125.4, 123.9, 108.9, 106.4, 55.8, 41.4; IR (KBr) 3421, 3003, 2937, 1507, 749 cm^{-1}; HRMS (FAB$^+$) calculated for $C_{29}H_{25}N_2O_2$ [M+H]$^+$ 433.1910, found 433.1907; mp 154.3–155.3 °C; $[\alpha]^{20}_D$ −293.7 (*c* 0.20, $CHCl_3$, (*S*)-configuration).

Synthesis of 1c

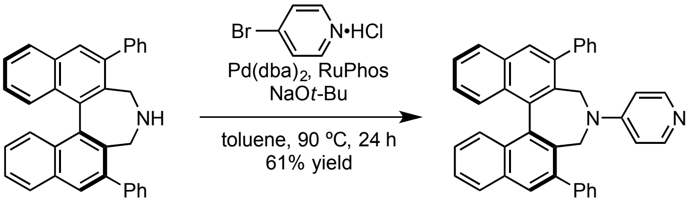

(*S*)-2,6-diphenyl-4-(pyridin-4-yl)-4,5-dihydro-3H-dinaphtho[2,1-c:1′,2′-e]azepine (1c)

To a solution of **23**[5] (58.0 mg, 0.130 mmol), 4-bromopyridine hydrochloride (50.9 mg, 0.262 mmol), NaO*t*-Bu (75.1 mg, 0.781 mmol), RuPhos (4.90 mg, 10.5 μmol) in toluene (1.30 mL) was added Pd(dba)$_2$ (3.00 mg, 5.22 μmol) at room temperature. The reaction mixture was warmed up to 90 °C and stirred for 24 h. After being cooled to room temperature, water (2 mL) was added to reaction mixture, and then passed through a short pad of celite. The resulting solution was extracted with toluene (5 mL × 2) and the organic layer was washed with brine (5 mL × 2), dried over $MgSO_4$ and concentrated *in vacuo*. The purification of the crude product by flash column chromatography on silica gel (eluent: hexane/EtOAc = 5/1 + 1% Et_3N to EtOAc + 1% Et_3N, v/v) gave **1c** (41.8 mg, 79.7 μmol, 61% yield) as a colorless solid.

^{1}H NMR (400 MHz, $CDCl_3$) δ 7.98–7.93 (m, 6H), 7.56–7.49 (m, 4H), 7.43–7.27 (m, 12H), 6.16 (d, *J* = 6.8 Hz, 2H), 4.88 (d, *J* = 13.0 Hz, 2H), 3.66 (d, *J* = 13.0 Hz, 2H); ^{13}C NMR (100 MHz, $CDCl_3$) δ 152.4, 149.6, 140.9, 140.0, 136.4, 132.9, 131.3, 130.8, 130.0, 129.8, 128.6, 128.5, 127.8, 127.6, 126.6, 126.3, 108.5, 45.9; IR (KBr) 3053, 2925, 2852, 2360, 1593, 703 cm^{-1}; HRMS (FAB$^+$) calculated for $C_{39}H_{29}N_2$ [M+H]$^+$ 525.2325,

found 525.2325; mp 143.5–144.0 °C; $[\alpha]^{20}_{D}$ −170.3 (*c* 0.20, $CHCl_3$, (*S*)-configuration).

Synthesis of 1d

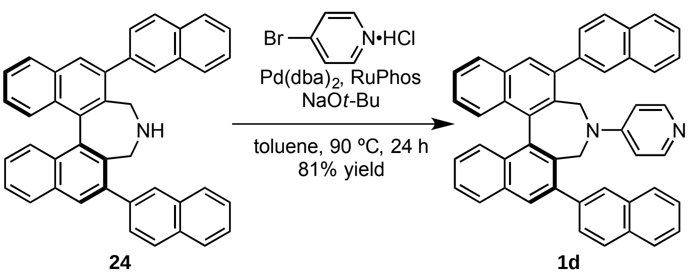

(*S*)-2,6-di(naphthalen-2-yl)-4-(pyridin-4-yl)-4,5-dihydro-3H-dinaphtho[2,1-c:1′,2′-e]azepine (1d)

To a solution of **24**[5] (201 mg, 0.367 mmol), 4-bromopyridine hydrochloride (143 mg, 0.734 mmol), NaO*t*-Bu (212 mg, 2.21 mmol), RuPhos (13.7 mg, 29.4 μmol) in toluene (3.70 mL) was added $Pd(dba)_2$ (8.60 mg, 15.0 μmol) at room temperature. The reaction mixture was warmed up to 90 °C and stirred for 24 h. After being cooled to room temperature, $CHCl_3$ (10 mL) and water (5 mL) was added to reaction mixture, and then passed through a short pad of celite. The resulting solution was extracted with $CHCl_3$ (5 mL × 2) and the organic layer was washed with brine (10 mL×2), dried over $MgSO_4$ and concentrated *in vacuo*. The purification of the crude product by recrystallization from $CHCl_3$/hexane gave **1d** (187 mg, 0.299 mmol, 81% yield) as a colorless solid.

^{1}H NMR (400 MHz, $CDCl_3$) δ 8.07 (s, 2H), 7.99 (d, *J* = 8.4 Hz, 2H), 7.89 (d, *J* = 8.0 Hz, 4H), 7.83 (bd, *J* = 5.6 Hz, 4H), 7.57–7.49 (m, 10H), 7.36 (t, *J* = 7.4 Hz, 4H), 6.05 (d, *J* = 5.6 Hz, 2H), 4.97 (bd, *J* = 13.2 Hz, 2H), 3.79 (bd, *J* = 13.2 Hz, 2H); ^{13}C NMR (150 MHz, $CDCl_3$) δ 152.4, 149.5, 139.9, 136.4, 133.3, 133.0, 132.6, 131.4, 130.9, 130.4, 128.9, 128.6, 128.2, 127.8, 126.8, 126.7, 126.5, 126.4, 108.6, 46.3; IR (KBr) 3053, 2866, 2359, 1589, 749 cm^{-1}; HRMS (FAB$^+$) calculated for $C_{47}H_{33}N_2$ $[M+H]^+$ 625.2638, found 625.2656; mp >280 °C; $[\alpha]^{20}_{D}$ −173.6 (*c* 0.20, $CHCl_3$, (*S*)-configuration).

An overview for synthesis of catalyst 1e

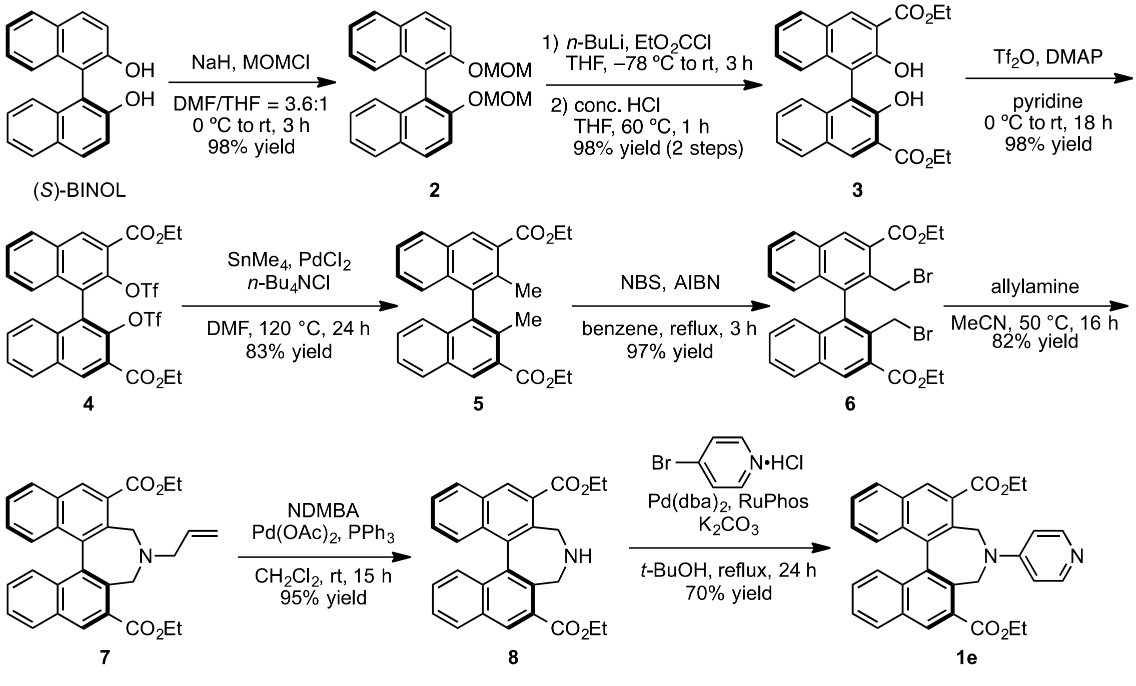

Synthesis of 2

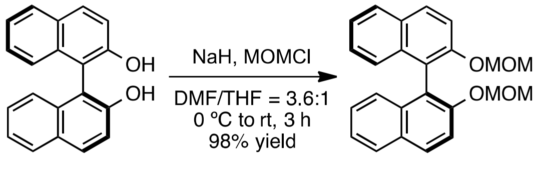

(*S*)-BINOL 2

(*S*)-2,2'-Bis(methoxymethoxy)-1,1'-binaphthalene (2)[13]

We modified solvent, substrate concentration, and purification method of known procedure. [13]

To a suspension of NaH (60% dispersion in paraffin liquid, 15.4 g, 385 mmol) in DMF (77.0 mL) was added dropwise solution of (*S*)-BINOL (50.0 g, 175 mmol) in THF (40.0 mL) and DMF (65.5 mL) for 10 min at 0 ºC. The resulting solution was warmed up to room temperature and stirred for 5 min at room temperature, then chloromethyl methyl ether [MOMCl] (29.2 mL, 384 mmol) was added dropwise for 40 min at room temperature. The reaction mixture was stirred for 3 h. After stirring for 3 h, the reaction mixture was poured into cold water (200 mL). The resulting solution was extracted with EtOAc (300 mL × 3), washed with brine (300 mL), dried over Mg_2SO_4 and concentrated *in vacuo*. The purification of the crude product by recrystallization from toluene/hexane gave **2** (64.5 g, 172 mmol, 98% yield) as a colorless solid.

^{1}H NMR (400 MHz, $CDCl_3$) δ 7.96 (d, *J* = 9.0 Hz, 2H), 7.88 (d, *J* = 8.3 Hz, 2H), 7.58 (d, *J* = 9.0 Hz, 2H), 7.35 (ddd, *J* = 8.3, 6.8, 1.3 Hz), 7.23 (ddd, *J* = 8.3, 6.8, 1.4 Hz, 2H), 7.16 (d, *J* = 8.3 Hz, 2H), 5.10 (d, *J* = 6.8 Hz, 2H), 4.99 (d, *J* = 6.8 Hz, 2H), 3.15 (s, 6H); ^{13}C NMR (100 MHz, $CDCl_3$) δ 152.8, 134.1, 130.0, 129.5, 128.0, 126.4, 125.7, 124.2, 121.4, 117.4, 95.3, 56.0; IR (KBr) 2952, 1622, 1593, 1507, 1478, 1241, 1089, 1041 cm^{-1}.

Synthesis of 3

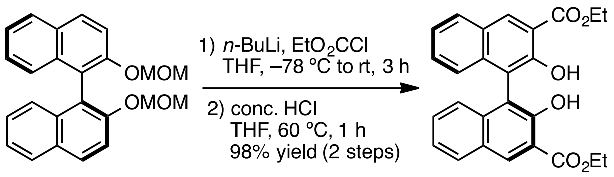

2 3

(*S*)-Diethyl 2,2'-dihydroxy-[1,1'-binaphthalene]-3,3'-dicarboxylate (3)[6]

We modified substrate concentration and purification method of known procedure.[13]

To a solution of **2** (29.5 g, 78.8 mmol) in THF (200 mL) was added dropwise solution of *n*-BuLi (1.58 M, 125 mL, 197 mmol) in hexane for 10 min at –78 ºC. The reaction solution was warmed up to room temperature and stirred for 1 h. After stirring for 1 h, the reaction suspension was re-cooled to –78 ºC and Et_2CO_2Cl was carefully added dropwise for 20 min at –78 ºC. The reaction mixture was warmed up room temperature and stirred for 2 h, then water was carefully added to quench the reaction. After removal water using syringe from the mixture, 6 M aqueous HCl (200 mL, 1.20 mol) was added to resulting solution. The reaction solution was heated to 60 ºC and sirred for 1 h. Afetr being cooled to room temperature, the resulting solid was collected by filtration and washed with copious hexane. After drying of the solid, **3** (33.1 g, 76.9 mmol, 98% yield based on **2**) was isolated as a yellow solid.

^{1}H NMR (400 MHz, $CDCl_3$) δ 10.81 (s, 2H), 8.69 (s, 2H), 7.94–7.92 (m, 2H), 7.36–7.32 (m, 4H), 7.16–7.14 (m, 2H), 4.49–4.55 (m, 4H), 1.56 (t, *J* = 7.0 Hz, 6H); ^{13}C NMR (100 MHz, $CDCl_3$) δ 170.3, 154.2, 137.3, 132.9, 129.9, 129.5, 127.3, 124.8, 124.0, 117.1, 114.5, 62.0, 14.4; IR (KBr) 2921, 1840, 1665, 1580, 1072, 846, 680 cm^{-1}; $[\alpha]^{21}_{D}$ –112.9 (*c* 1.04, CH_2Cl_2, (*S*)-configuration) [lit.[6] $[\alpha]^{27}_{D}$ +168.5 (*c* 1.0, CH_2Cl_2, (*R*)-configuration)].

Synthesis of 4

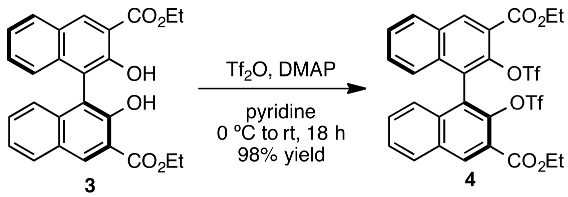

3 4

(*S*)-Diethyl 2,2'-bis(((trifluoromethyl)sulfonyl)oxy)-[1,1'-binaphthalene]-3,3'-dicarboxylate (4)[6]

To a solution of **3** (14.6 g, 33.9 mmol) and DMAP (828 mg, 6.78 mmol) in pyridine (188 mL) was added dropwise trifluoromethanesulfonic anhydride [Tf_2O] (17.3 mL, 103 mmol) for 10 min at 0 ºC. The reaction mixture was warmed up to room temperature and stirred for 18 h at room temperature. The resulting solution was poured carefully into cold water and extracted with Et_2O (200 mL × 3), washed with 6 M aqueous HCl (200 mL × 3), dried over $MgSO_4$, and concentration *in vacuo*. Subsequently, the resulting solid was subsequently thoroughly washed with MeOH. After filtration and drying of the solid, **4** (23.0 g, 33.1 mmol, 98% yield) was isolated as a colorless solid.

^{1}H NMR (400 MHz, $CDCl_3$) δ 8.75 (s, 2H), 8.08 (d, *J* = 8.2 Hz, 2H), 7.63 (t, *J* = 7.5 Hz, 2H), 7.45 (t, *J* = 7.7 Hz 2H), 7.19 (d, *J* = 8.6 Hz, 2H), 4.62–4.54 (m, 2H), 4.47–4.39 (m, 2H), 1.46 (t, *J* = 7.0 Hz, 6H); ^{13}C NMR (100 MHz, $CDCl_3$) δ 164.6, 142.7, 135.8, 134.9, 131.5, 129.8, 129.6, 128.3, 127.7, 125.0, 124.1, 118.0 (q, $J_{C\text{-}F}$ = 1275.5), 62.6, 14.2; IR (KBr) 2900, 2359, 1987, 1836, 1750, 1587, 1498, 693 cm^{-1}; $[\alpha]^{21}_D$ +116.2 (*c* 1.03, CH_2Cl_2, (*S*)-configuration) [lit.[6] $[\alpha]^{27}_D$ –159.2 (*c* 1.0, CH_2Cl_2, (*R*)-configuration)].

Synthesis of 5

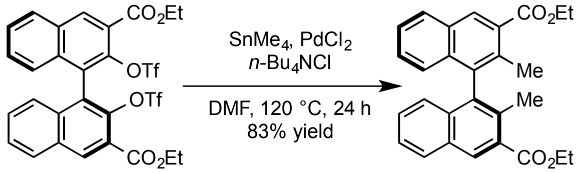

4 5

(*S*)-Diethyl-2,2′-dimethyl-1,1′-binaphthyl-3,3′-dicarboxylate (5)[6]

Initially, Negishi cross coupling (with Me_2Zn) was conducted in according literature,[6] but we modified this procedure to Migita-Kosugi-Stille cross coupling (with Me_4Sn) because Me_2Zn is costly and pyrophoric reagent, and unsuitable for scale up.

Small scale reaction (2.00 mmol scale)

To a solution of **4**[6] (139 mg, 2.00 mmol), LiCl (86.5 mg, 2.04 mmol), *n*-Bu_4NCl (223 mg, 0.803 mmol) and Me_4Sn (83.0 μL, 0.600 mmol) in DMF (2.70 mL) was added $PdCl_2$ (3.70 mg, 20.9 μmol) at room temperature. The reaction mixture was heated at 120 ºC and stirred for 24 h, then the reaction mixture was under reduced pressure to removed excess Me_4Sn, and the resulting suspension was filtered through a short pad of silica gel with copious washing Et_2O. The resulting solution was extracted with Et_2O (10 mL × 3). The organic layer was washed with water (5 mL × 3), dried over $MgSO_4$ and concentrated *in vacuo*. The purification of the crude product by flash column chromatography on silica gel (eluent: hexane/EtOAc = 8/1, v/v) gave **5** (75.1 mg, 0.176 mmol, 88% yield) as a pale yellow solid.

Large scale reaction (32.0 mmol scale)

To a solution of **4**[6] (22.2 g, 32.0 mmol), LiCl (6.85 g, 162 mmol), *n*-Bu_4NCl (25.3 g, 91.0 mmol) and Me_4Sn (9.00 mL, 65.0 mmol) in DMF (175 mL) was added $PdCl_2$ (397 mg, 2.24 mol) at room temperature. The

reaction mixture was heated at 120 ºC and stirred for 20 h, then the reaction mixture was under reduced pressure to removed excess Me_4Sn, and the resulting suspension was filtered through a short pad of silica gel with copious washing Et_2O. The resulting solution was extracted with Et_2O (150 mL × 3). The organic layer was washed with water (150 mL × 3), dried over $MgSO_4$ and concentrated *in vacuo*. The purification of the crude product by flash column chromatography on silica gel (eluent: hexane/Et_2O = 5/1, v/v) gave **5** (11.3 g, 26.5 mmol, 83% yield) as a pale yellow solid.

^{1}H NMR (400 MHz, $CDCl_3$) δ 8.53 (s, 2H), 7.93 (d, *J* = 8.4 Hz, 2H), 7.45 (t, *J* = 7.3 Hz, 2H), 7.29 (ddd, *J* = 8.4, 7.3, 1.4 Hz, 2H), 6.96 (d, *J* = 8.4 Hz, 2H), 4.47 (q, *J* = 7.2 Hz, 4H), 2.22 (s, 6H), 1.48 (t, *J* = 7.2 Hz, 6H); ^{13}C NMR (100 MHz, $CDCl_3$) δ 168.6, 136.9, 134.2, 133.9, 131.2, 129.9, 129.1, 128.5, 126.0, 125.9, 61.3, 18.1, 14.4; IR (KBr) 2927, 1926, 1726, 1622, 1202, 1062, 905, 788 cm^{-1}; $[\alpha]^{23}_D$ −23.4 (*c* 0.76, CH_2Cl_2, (*S*)-configuration) [lit.[6] $[\alpha]^{27}_D$ +74.2 (*c* 0.76, CH_2Cl_2, (*R*)-configuration)].

Synthesis of 6

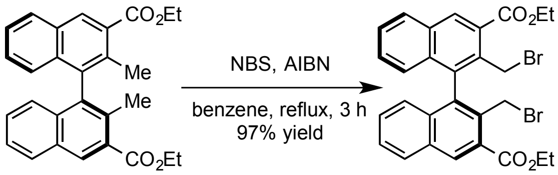

5 **6**

(*S*)-Diethyl 2,2′-bis(bromomethyl)-1,1′-binaphthyl-3,3′-dicarboxylate (6)[6]

To a suspension of **5**[6] (11.3 g, 26.5 mmol), NBS (10.4 g, 58.4 mmol) in benzene (73.0 mL) was added AIBN (442 mg, 2.69 mmol) at room temperature. The reaction mixture was heated to refluxed temperature and stirred for 2 h, and then additional AIBN (218 mg, 1.22 mmol) was added to the reaction mixture at room temperature. After addition was completed, the reaction mixture was stirred for 1 h at refluxed temperature. After being cooled to room temperature, the reaction mixture was poured into water (100 mL) and extracted with Et_2O (100 mL × 3). The organic layer was washed with brine (50 mL), dried over $MgSO_4$ and concentrated *in vacuo*. The crude product was passed through a short pad of silica gel (eluent: hexane/Et_2O = 3/1, v/v) to give a pale yellow solid (15.1 g, 25.8 mmol, 97% yield). This crude product was directly used for the following reaction without further purification. The analytical data of **6** was obtained by the purification of the crude product by flash column chromatography on silica gel (eluent: hexane/Et_2O = 5/1 to 3/1, v/v) as a pale yellow solid.

^{1}H NMR (400 MHz, $CDCl_3$) δ 8.66 (s, 2H), 8.01 (d, *J* = 8.5 Hz, 2H), 7.56 (ddd, *J* = 8.5, 7.2, 1.0 Hz, 2H), 7.36 (ddd, *J* = 8.5, 7.2, 1.6 Hz, 2H), 7.02 (d, *J* = 7.2 Hz, 2H), 4.80 (d, *J* = 9.8 Hz, 2H), 4.66 (d, *J* = 9.8 Hz, 2H), 4.52 (q, *J* = 7.2 Hz, 4H), 1.51 (t, *J* = 7.2 Hz, 6H); ^{13}C NMR (100 MHz, $CDCl_3$) δ 167.2, 135.8, 133.5, 133.4, 133.2, 132.1, 129.0, 128.9, 128.4, 127.7, 127.1, 61.7, 30.0, 14.2; IR (KBr) 2980, 1716, 1425, 1242, 1057, 800, 752 cm^{-1}; $[\alpha]^{23}_D$ −48.9 (*c* 0.64, CH_2Cl_2, (*S*)-configuration) [lit.[6] $[\alpha]^{24}_D$ +96.7 (*c* 0.64, CH_2Cl_2, (*R*)-configuration)].

Synthesis of 7

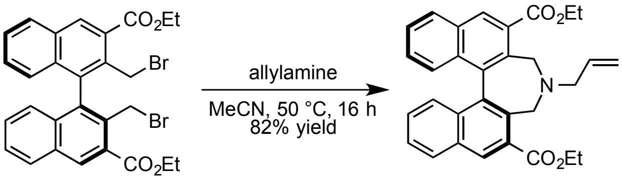

6 **7**

(*S*)-Diethyl 4-allyl-4,5-dihydro-3H-dinaphtho[2,1-c:1′,2′-e]azepine-2,6-dicarboxylate (7)[6]

To a solution of **6**[3] (23.4 g, 40.0 mmol) in MeCN (400 mL) was added allylamine (8.90 mL, 119 mmol) at 0 ºC. The reaction mixture was heated to 50 ºC and stirred for 16 h, then poured into water (300 mL). The resulting solution was extracted with CH_2Cl_2 (100 mL × 3). The organic layer was dried over $MgSO_4$ and concentrated *in vacuo*. The purification of the crude product by flash column chromatography on silica gel (eluent: hexane/EtOAc = 9/2, v/v) gave **7** (15.7 g, 32.7 mmol, 82% yield) as a colorless foam.

^{1}H NMR (400 MHz, $CDCl_3$) δ 8.58 (s, 2H), 8.03 (d, J = 8.0 Hz, 2H), 7.50 (t, J = 8.0 Hz, 2H), 7.32 (t, J = 8.0 Hz, 2H), 7.24 (d, J = 8.0 Hz, 2H), 6.05-5.90 (m, 1H) 5.18-5.08 (m, 2H), 4.80 (d, J = 12.9 Hz, 2H), 4.53 (q, J = 7.1 Hz, 4H), 3.30 (dd, J = 13.1, 5.6 Hz, 1H), 3.01-2.89 (m, 3H), 1.49 (td, J = 7.1, 2.1 Hz, 6H); ^{13}C NMR (100 MHz, $CDCl_3$) δ 168.0, 136.9, 136.5, 132.7, 132.0, 131.7, 129.3, 128.8, 128.0, 127.2, 126.3, 117.1, 61.4, 58.9, 49.8, 14.4; IR (KBr) 2979, 2790, 1721, 1443, 1241, 1204, 1056, 754 cm^{-1}; $[\alpha]^{22}_D$ +202.6 (*c* 1.00, CH_2Cl_2, (*S*)-configuration) (lit.[6] $[\alpha]^{27}_D$ −431.5 [*c* 1.0, CH_2Cl_2, (*R*)-configuration]).

Synthesis of 8

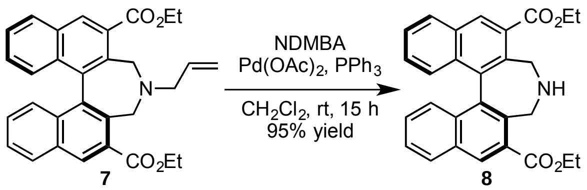

(*S*)-Diethyl 4,5-dihydro-3H-dinaphtho[2,1-c:1′,2′-e]azepine-2,6-dicarboxylate (8)

To a solution of **7**[6] (15.7 g, 32.7 mmol), NDMBA (10.3 g, 66.0 mmol) and Ph_3P (1.84 g, 7.02 mmol) in CH_2Cl_2 (110 mL) was added $Pd(OAc)_2$ (377 mg, 1.68 μmol) at room temperature. The reaction mixture was stirred for 15 h at room temperature, then water (100 mL) was added. The resulting solution was extracted with CH_2Cl_2 (100 mL × 3). The organic layer was washed saturated aqueous $NaHCO_3$ (100 mL × 3), dried over $MgSO_4$ and concentrated *in vacuo*. The purification of the crude product by flash column chromatography on silica gel (eluent: EtOAc to EtOAc/MeOH = 30/1, v/v) gave **8** (13.7 g, 31.2 mmol, 95% yield) as a pale yellow foam.

^{1}H NMR (400 MHz, $CDCl_3$) δ 8.58 (s, 2H), 8.01 (d, J = 8.0 Hz, 2H), 7.49 (dt, J = 8.0, 1.2 Hz, 2H), 7.31 (dt, J = 8.0, 1.2 Hz, 2H), 7.42 (d, J = 8.0 Hz, 2H), 4.78 (d, J = 12.5 Hz, 2H), 4.48 (qd, J = 7.1, 3.1 Hz, 4H), 3.22 (d, J = 12.5 Hz, 2H), 2.41 (bs, 1H), 1.48 (t, J = 7.1 Hz, 6H); ^{13}C NMR (100 MHz, $CDCl_3$) δ 168.1, 136.7, 133.6, 132.7, 132.2, 131.7, 129.4, 128.5, 128.0, 127.3, 126.3, 61.5, 44.0, 14.5; IR (KBr) 2984, 1722, 1242, 1137, 1053, 909, 773, cm^{-1}; HRMS (FAB$^+$) calculated for $C_{28}H_{26}NO_4$ $[M+H]^+$ 440.1856, found 440.1857; $[\alpha]^{20}_D$ +433.4 (*c* 0.53, CH_2Cl_2, (*S*)-configuration).

Synthesis of 1e

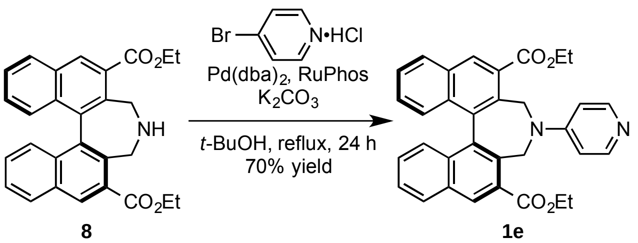

(*S*)-Diethyl 4-(pyridin-4-yl)-4,5-dihydro-3H-dinaphtho[2,1-c:1′,2′-e]azepine-2,6-dicarboxylate (1e)

Small scale reaction (0.200 mmol scale)

To a suspension of **8** (87.9 mg, 0.200 mmol), 4-bromopyridine hydrochloride (77.7 mg, 0.400 mmol), RuPhos (7.70 mg, 16.5 mmol) and potassium carbonate (167 mg, 1.21 mmol) in *t*-BuOH (2.00 mL) was added $Pd(dba)_2$ (4.70 mg, 8.17 μmol) at room temperature. The reaction mixture was heated to refluxed

temperature and vigorously stirred for 24 h, and then water (3 mL) was added and filtered off. After filtrate was concentrated *in vacuo*, the resulting solution was extracted with CH_2Cl_2 (5 mL × 3), dried over $MgSO_4$ and concentrated *in vacuo*. The purification of the crude product by flash column chromatography on silica gel (eluent: EtOAc + 3% Et_3N) gave **1e** (80.0 mg, 0.155 mmol, 78% yield) as a pale yellow solid.

Large scale reaction (31.2 mmol scale)

To a suspension of **8** (13.7 g, 31.2 mmol), 4-bromopyridine hydrochloride (12.2 g, 62.7 mmol), RuPhos (1.10 g, 2.36 mmol) and potassium carbonate (26.0 g, 188 mmol) in *t*-BuOH (313 mL) was added $Pd(dba)_2$ (721 mg, 1.25 mmol) at room temperature. The reaction mixture was heated to refluxed temperature and vigorously stirred for 24 h, and then water (100 mL) was added and filtered off. After filtrate was concentrated *in vacuo*, the resulting solution was extracted with CH_2Cl_2 (100 mL × 3), dried over $MgSO_4$ and concentrated *in vacuo*. The purification of the crude product by flash column chromatography on silica gel (eluent: EtOAc + 3% Et_3N) gave **1e** (11.2 g, 21.7 mmol, 70% yield) as a pale yellow solid.

^{1}H NMR (400 MHz, $CDCl_3$) δ 8.60 (s, 2H), 8.22 (d, *J* = 5.7 Hz, 2H), 8.04 (d, *J* = 7.9 Hz, 2H), 7.55 (t, *J* = 7.9 Hz, 2H), 7.37 (t, *J* = 7.9 Hz, 2H), 7.31 (d, *J* = 7.9 Hz, 2H), 6.78 (d, *J* = 5.7 Hz, 2H), 5.95 (d, *J* = 13.1 Hz, 2H), 4.46-4.32 (m, 4H), 3.53 (d, *J* = 13.1 Hz, 2H), 1.37 (t, *J* = 7.1 Hz, 6H); ^{13}C NMR (100 MHz, $CDCl_3$) δ 168.0, 153.5, 150.1, 137.0, 132.8, 132.7, 132.0, 131.2, 129.6, 128.6, 128.1, 127.4, 127.0, 109.1, 61.9, 44.3, 14.4; IR (KBr) 2963, 2501, 2352, 1972, 1726, 986, 754, 588 cm^{-1}; HRMS (FAB$^+$) calculated for $C_{33}H_{29}N_2O_4$ $[M+H]^+$ 517.2121, found 517.2099; mp 195.5–196.6 ºC; $[\alpha]^{19}_D$ –388.9 (*c* 0.53, CH_2Cl_2, (*S*)-configuration).

Synthesis of 1f

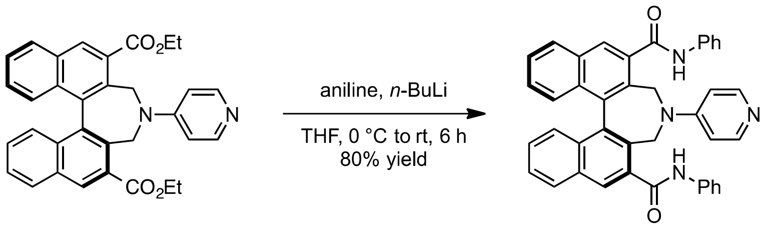

1e 1f

(*S*)-*N*^{2},*N*^{6}-diphenyl-4-(pyridin-4-yl)-4,5-dihydro-3*H*-dinaphtho[2,1-*c*:1′,2′-*e*]azepine-2,6-dicarboxamide (1f)

To a solution of aniline (7.30 μL, 80.1 μmol) in THF (0.145 mL) was added a solution of *n*-BuLi in hexane (1.62 M, 99.0 μL, 160 μmol) at 0 °C. The reaction mixture was warmed up to room temperature and stirred for 1 h. After re-cooling to 0 °C, solution of **1e** (8.30 mg, 16.1 μmol) in THF (0.290 mL) was added dropwise to reaction mixture. The reaction mixture was warmed up to room temperature and stirred for 6 h. Then, the reaction mixture was poured into water (1 mL) and extracted with EtOAc (3 mL × 3). The organic layer was dried over $MgSO_4$ and concentrated *in vacuo*. The purification of the crude product by flash column chromatography on silica gel (eluent: EtOAc) gave **1f** (7.80 mg, 12.8 μmol, 80% yield) as a pale brown solid.

^{1}H NMR (400 MHz, $CDCl_3$) δ 8.68 (bs, 2H), 8.23 (s, 2H), 7.93 (d, *J* = 7.6 Hz, 2H), 7.59–7.21 (m, 14H), 7.11 (t, *J* = 7.2 Hz, 2H), 6.45 (d, *J* = 5.6 Hz, 2H), 5.28 (d, *J* = 13.2 Hz, 2H), 3.53 (d, *J* = 13.2 Hz, 2H), 2.44 (bs, 2H); ^{13}C NMR (100 MHz, $CDCl_3$) δ 167.7, 153.4, 149.1, 137.9, 136.9, 134.4, 132.2, 131.8, 129.7, 129.2, 129.1, 129.0, 128.1, 127.5, 127.3, 125.0, 120.3, 109.4, 77.4, 45.9; IR (KBr) 3055, 2924, 1645, 1597, 1533, 1437, 1319, 1250, 901, 752 cm^{-1}; HRMS (EI$^+$) calculated for $C_{41}H_{30}N_4O_2$ $[M]^+$ 610.2363, found 610.2390; mp >280 ºC; $[\alpha]^{25}_D$ –208.0 (*c* 0.29, $CHCl_3$, (*S*)-configuration).

Synthesis of 1g

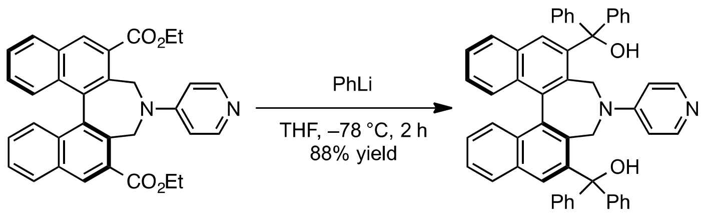

1e 1g

(*S*)-2,6-bis(hydroxydiphenylmethyl)-4-(pyridin-4-yl)-4,5-dihydro-3*H*-dinaphtho[2,1-*c*:1′,2′-*e*]azepine (1g)

To a solution of **1e** (103 mg, 0.200 mmol) in THF (2.00 mL) was added a solution of PhLi in *n*-Bu_2O (1.6 M, 750 µL, 1.20 mmol) at −78 °C. The reaction mixture was stirred for 2 h at −78 °C. Then, water (30 mL) was added to reaction mixture and extracted with $CHCl_3$ (10 mL × 3). The organic layer was washed with brine (10 mL × 3), dried over $MgSO_4$ and concentrated *in vacuo*. The purification of the crude product by recrystallization from $CHCl_3/Et_2O$ gave **1g** (130 mg, 0.176 mmol, 88% yield) as a colorless solid.

^1H NMR (400 MHz, $CDCl_3$) δ 7.77 (d, *J* = 6.0 Hz, 2H), 7.64 (d, *J* = 8.4 Hz, 2H), 7.49–7.17 (m, 18H), 7.15-6.95 (m, 10H), 5.91 (d, *J* = 6.0 Hz, 2H), 5.23 (d, *J* = 13.2 Hz, 2H), 3.40 (d, *J* = 13.2 Hz, 2H), 3.34 (bs, 1H); ^{13}C NMR (150 MHz, $CDCl_3$) δ 152.9, 147.9, 147.6, 145.6, 142.5, 138.3, 132.3, 131.6, 131.2, 130.7, 128.8, 128.3, 127.9, 127.8, 127.5, 127.4, 127.2, 126.6, 126.2, 108.7, 83.2, 45.6; IR (KBr) 3057, 1597, 1447, 1396, 1242, 997, 748, 696 cm^{-1}; HRMS (ESI^+) calculated for $C_{53}H_{41}N_2O_2$ $[M+H]^+$ 737.3163, found 737.3130; mp >280 ºC; $[\alpha]^{19}_D$ –260.5 (*c* 0.27, $CHCl_3$, (*S*)-configuration).

Synthesis of 1h

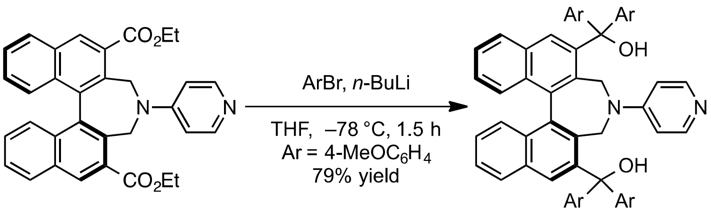

1e 1h

(*S*)-2,6-bis(bis(4-methoxyphenyl)(hydroxy)methyl)-4-(pyridin-4-yl)-4,5-dihydro-3*H*-dinaphtho[2,1-*c*:1′, 2′-*e*]azepine (1h)

To a solution of 4-bromoanisole (37.0 µL, 0.300 mmol) in THF (0.250 mL) was added dropwise a solution of *n*-BuLi in hexane (1.60 M, 190 µL, 0.304 mmol) for 1 min at −78 °C and stirred for 20 min. After stirring for 20 min at −78 °C, a solution of **1e** (25.7 mg, 49.7 µmol) in THF (1.00 mL) was added to reaction mixture at −78 °C. The reaction mixture was stirred for 1.5 h at −78 °C. Then, additional solution of *n*-BuLi in hexane (1.60 M, 190 µL, 0.304 mmol) was added to reaction mixture to destroy 1-bromobutane which was generated in the reaction. The reaction mixture was warmed up to room temperature and stirred for 1 h, and then water (20 mL) was added. The resulting solution was extracted with $CHCl_3$ (10 mL × 3), dried over $MgSO_4$ and concentrated *in vacuo*. The resulting solid was subsequently thoroughly washed with Et_2O. After filtration and drying of the solid, **1h** (33.6 mg, 39.2 µmol, 79% yield) was isolated as a pale yellow solid.

^1H NMR (400 MHz, $CDCl_3$) δ 7.68–7.56 (m, 4H), 7.36 (ddd, *J* = 7.9, 5.5, 2.5 Hz, 2H), 7.25–7.17 (m, 6H), 7.13 (d, *J* = 8.5 Hz, 4H), 6.91 (d, *J* = 8.5 Hz, 4H), 6.84 (d, *J* = 8.5 Hz, 4H), 6.47 (d, *J* = 8.5 Hz, 4H), 5.87 (d, *J* = 6.4 Hz, 2H), 5.35 (d, *J* = 13.2 Hz, 2H), 3.98 (bs, 1.4H), 3.80 (s, 6H), 3.60 (s, 6H), 3.37 (d, *J* = 13.2 Hz, 2H); ^{13}C NMR (100 MHz, $CDCl_3$, 50 ºC) δ 159.0, 158.8, 153.0, 147.8, 143.1, 140.7, 138.3, 138.3, 132.7, 131.7, 131.3, 130.5, 129.2, 128.9, 128.7, 127.5, 126.4, 126.1, 113.6, 113.1, 108.8, 82.8, 55.4, 55.1, 45.6; IR

(KBr) 2363, 1603, 1506, 1248, 1175, 1031, 993, 829, 750 cm^{-1}; HRMS (ESI$^+$) calculated for $C_{57}H_{49}N_2O_6$ [M+H]$^+$ 857.3585, found 857.3546; mp 235 ºC dec; $[\alpha]^{24}_D$ –206.4 (*c* 0.29, $CHCl_3$, (*S*)-configuration).

Synthesis of 1i

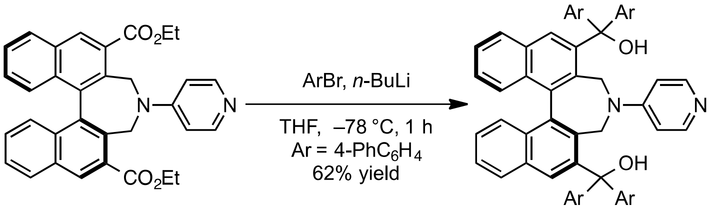

1e **1i**

(*S*)-2,6-bis(di([1,1′-biphenyl]-4-yl)(hydroxy)methyl)-4-(pyridin-4-yl)-4,5-dihydro-3*H*-dinaphtho[2,1-*c*:1′,2′-*e*]azepine (1i)

To a solution of 4-bromobiphenyl (280 mg, 1.20 mmol) in THF (3.00 mL) was added dropwise a solution of *n*-BuLi in hexane (1.55 M, 775 μL, 1.20 mmol) for 1 min at −78 °C and stirred for 20 min. After stirring for 20 min at −78 °C, a solution of **1e** (103 mg, 0.200 mmol) in THF (1.50 mL) was added to reaction mixture at −78 °C. The reaction mixture was stirred for 1 h at −78 °C. Then, additional solution of *n*-BuLi in hexane (1.55 M, 775 μL, 1.20 mmol) was added to reaction mixture to destroy 1-bromobutane which was generated in the reaction. The reaction mixture was warmed up to room temperature and stirred for 1 h, and then water (20 mL) was added. The resulting mixture was extracted with $CHCl_3$ (20 mL × 3), dried over $MgSO_4$ and concentrated *in vacuo*. The purification of the crude product by flash column chromatography on silica gel (eluent: $CHCl_3$/MeOH = 30/1, v/v) gave **1i** (128 mg, 0.123 mmol, 62% yield) as a colorless solid.
^{1}H NMR (400 MHz, $CDCl_3$) δ 7.69 (d, *J* = 7.8 Hz, 2H), 7.64–7.54 (m, 10H), 7.47–7.54 (m, 36 H), 6.01 (d, *J* = 5.4 Hz, 2H), 5.43 (d, *J* = 12.9 Hz, 2H), 4.39 (bs, 1.2H), 3.46 (d, *J* = 12.9 Hz, 2H); ^{13}C NMR (100 MHz, $CDCl_3$) δ 153.2, 147.0, 145.0, 142.5, 140.5, 140.4, 140.1, 140.1, 138.4, 132.4, 131.7, 131.2, 130.6, 128.9, 128.9, 128.2, 127.6, 127.3, 127.2, 127.2, 127.0, 126.6, 126.5, 126.2, 108.9, 83.0, 45.8; IR (KBr) 3055, 3028, 1593, 1508, 1485, 1398, 1242, 995, 893, 837, 748 cm^{-1}; HRMS (ESI$^+$) calculated for $C_{77}H_{57}N_2O_2$ [M+H]$^+$ 1041.4415, found 1041.4385; mp 257 ºC dec; $[\alpha]^{23}_D$ –172.6 (*c* 0.26, $CHCl_3$, (*S*)-configuration).

Synthesis of 1j

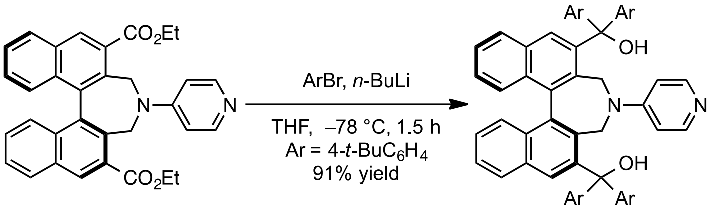

1e **1j**

(*S*)-2,6-bis(bis(4-(*tert*-butyl)phenyl)(hydroxy)methyl)-4-(pyridin-4-yl)-4,5-dihydro-3*H*-dinaphtho[2,1-*c*:1′,2′-*e*]azepine (1j)

To a solution of 1-bromo-4-tert-butylbenzene (2.00 g, 9.38 mmol) in THF (9.75 mL) was added dropwise a solution of *n*-BuLi in hexane (1.63 M, 5.75 mL, 9.37 mmol) for 1 min at −78 °C and stirred for 20 min. After stirring for 20 min at −78 °C, a solution of **1e** (1.01 g, 1.96 mmol) in THF (19.5 mL) was added to reaction mixture at −78 °C. The reaction mixture was stirred for 1 h at −78 °C, then water (30 mL) was added. The resulting mixture was extracted with $CHCl_3$ (75 mL × 3), dried over $MgSO_4$ and concentrated *in vacuo*. The purification of the crude product by twice flash column chromatography on silica gel (eluent: EtOAc to EtOAc + 3% Et_3N and Et_2O to EtOAc to EtOAc + 3% Et_3N) gave **1j** (1.71 g, 1.78 mmol, 91%

yield) as a pale yellow solid.

^{1}H NMR (400MHz, $CDCl_3$) δ 7.77–7.62 (m, 6H), 7.49–6.97 (m, 22H), 6.37 (d, *J* = 7.2 Hz, 2H), 5.58 (d, *J* = 13.2 Hz, 2H), 3.60 (d, *J* = 13.2 Hz, 2H), 1.32 (s, 18H), 1,26 (s, 18H); ^{13}C NMR (150 MHz, $CDCl_3$) δ 153.0, 149.8, 149.8, 147.6, 145.2, 143.6, 143.2, 138.1, 132.7, 131.6, 131.1, 130.3, 128.8, 127.7, 127.5, 127.4, 126.2, 125.8, 125.0, 124.5, 108.6, 82.6, 45.5, 34.6, 34.4, 31.5, 31.5; IR (KBr) 2961, 1595, 1508, 1398, 1233, 1107, 993, 829, 748 cm^{-1}; HRMS (ESI^+) calculated for $C_{69}H_{73}N_2O_2$ $[M+H]^+$ 961.5667, found 961.5626; mp 231.0 °C dec; $[\alpha]^{21}_D$ –280.4 (*c* 0.26, $CHCl_3$, (*S*)-configuration).

Synthesis of 1k

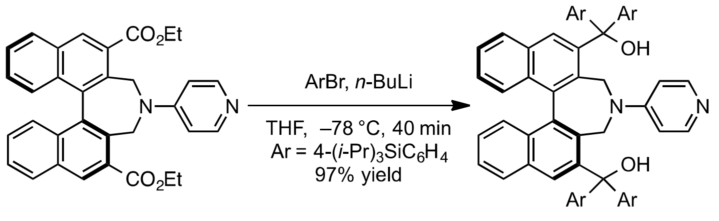

1e **1k**

(*S*)-2,6-bis(hydroxybis(4-(triisopropylsilyl)phenyl)methyl)-4-(pyridin-4-yl)-4,5-dihydro-3*H*-dinaphtho[2,1-*c*:1′,2′-*e*]azepine (1k)

To a solution of 1-bromo-4-[tris(iso-propyl)silyl)-benzene[14] (175 μL, 0.603 mmol) in THF (0.500 mL) was added dropwise a solution of *n*-BuLi in hexane (1.60 M, 375 μL, 0.600 mmol) for 1 min at −78 °C and stirred for 20 min. After stirring for 20 min at −78 °C, a solution of **1e** (51.9 mg, 0.100 mmol) in THF (1.50 mL) was added to reaction mixture at −78 °C. The reaction mixture was stirred for 40 min at −78 °C. Then, additional solution of *n*-BuLi in hexane (1.60 M, 375 μL, 0.600 mmol) was added to reaction mixture to destroy 1-bromobutane which was generated in the reaction. The reaction mixture was warmed up to room temperature and stirred for 1 h, and then water (20 mL) was added. The resulting mixture was extracted with $CHCl_3$ (20 mL × 3), dried over $MgSO_4$ and concentrated *in vacuo*. The purification of the crude product by flash column chromatography on silica gel (eluent: $CHCl_3$ to $CHCl_3$/MeOH = 4/1, v/v) gave **1k** (132 mg, 96.9 μmol, 97% yield) as a greenish yellow solid.

^{1}H NMR (400MHz, $CDCl_3$) δ 7.87 (d, *J* = 6.0 Hz, 2H), 7.53 (d, *J* = 8.0 Hz, 2H), 7.50–7.35 (m, 12H), 7.33–7.23 (m, 8H), 7.19 (d, *J* = 8.0 Hz, 2H), 7.08 (bs, 2H), 6.24 (bd, *J* = 6.0 Hz, 2H), 5.26 (bd, *J* = 12.6 Hz, 2H), 3.43 (bd, *J* = 12.6 Hz, 2H), 1.50–1.30 (m, 12H), 1.16–0.96 (m, 72H); ^{13}C NMR (100 MHz, $CDCl_3$) δ 153.7, 147.7, 146.9, 143.0, 138.2, 135.2, 134.9, 134.1, 132.2, 131.8, 131.2, 130.9, 128.8, 127.6, 127.3, 126.5, 126.4, 126.2, 109.2, 82.9, 46.2, 18.8, 10.9, 10.9; IR (KBr) 2943, 2864, 1595, 1464, 1242, 1101, 995, 883, 750 cm^{-1}; HRMS (ESI^+) calculated for $C_{89}H_{121}N_2O_2Si_4$ $[M+H]^+$ 1361.8500, found 1361.8480; mp 250 °C dec; $[\alpha]^{24}_D$ –172.8 (*c* 0.29, $CHCl_3$, (*S*)-configuration).

Synthesis of 1l

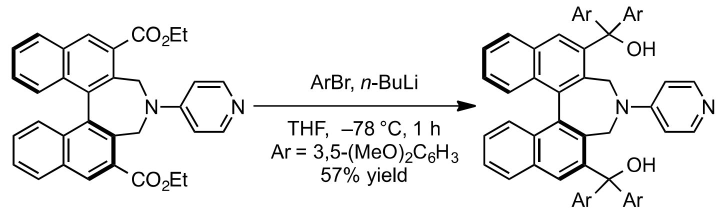

1e **1l**

(*S*)-2,6-bis(bis(3,5-dimethoxyphenyl)(hydroxy)methyl)-4-(pyridin-4-yl)-4,5-dihydro-3*H*-dinaphtho[2,1-*c*:1′,2′-*e*]azepine (1l)

To a solution of 1-bromo-3,5-dimethoxybenzene (522 mg, 2.40 mmol) in THF (2.00 mL) was added dropwise solution of *n*-BuLi in hexane (1.60 M, 1.50 mL, 2.40 mmol) for 1min at −78 °C and stirred for 20 min. After stirring for 20 min at −78 °C, a solution of **1e** (207 mg, 0.400 mmol) in THF (2.50 mL) was added to reaction mixture at −78 °C. The reaction mixture was stirred for 1 h at −78 °C. Then, additional solution of *n*-BuLi in hexane (1.60 M, 1.50 mL, 2.40 mmol) was added to reaction mixture to destroy 1-bromobutane which was generated in the reaction. The reaction mixture was warmed up to room temperature and stirred for 1 h at room temperature, and then water (30 mL) was added. The resulting mixture was extracted with $CHCl_3$ (30 mL × 3), dried over $MgSO_4$ and concentrated *in vacuo*. The purification of the crude product by flash column chromatography on silica gel (eluent: $CHCl_3$/MeOH = 20/1 + 3% Et_3N, v/v) gave **1l** (224 mg, 0.229 mmol, 57% yield) as a colorless solid.

^{1}H NMR (400 MHz, $CDCl_3$) δ 7.68 (d, *J* = 7.8 Hz, 4H), 7.39 (ddd, *J* = 7.1, 3.8, 2.9 Hz, 2H), 7.30 (s, 2H), 7.24–7.16 (m, 4H), 6.42 (s, 4H), 6.40 (s, 2H), 6.27 (s, 4H), 5.99–5.93 (m, 4H), 5.26 (d, *J* = 13.2 Hz, 2H), 3.70 (s, 12H), 3.59 (s, 12H), 3.46 (d, *J* = 13.2 Hz, 2H); ^{13}C NMR (100 MHz, $CDCl_3$) δ 160.7, 160.4, 150.0, 147.5, 141.8, 138.2, 131.9, 131.7, 131.2, 130.7, 128.9, 127.4, 126.6, 126.2, 108.7, 106.6, 106.5, 99.4, 97.8, 83.3, 55.5, 55.2, 45.6; IR (KBr) 2935, 1597, 1458, 1204, 1155, 1057, 752 cm^{-1}; HRMS (ESI$^+$) calculated for $C_{61}H_{57}N_2O_{10}$ $[M+H]^+$ 977.4008, found 977.3993; mp 242 ºC dec; $[\alpha]^{23}_D$ –212.1 (*c* 0.28, $CHCl_3$, (*S*)-configuration).

Synthesis of 1m

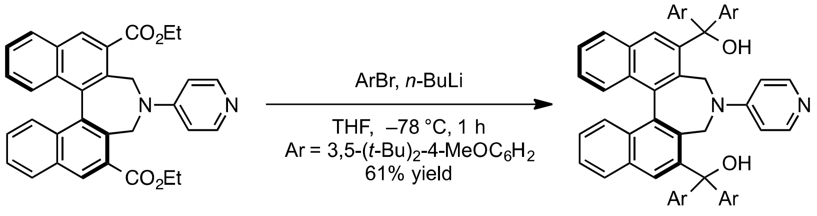

1e **1m**

(*S*)-2,6-bis(bis(4-(*tert*-butyl)-3,5-dimethoxyphenyl)(hydroxy)methyl)-4-(pyridin-4-yl)-4,5-dihydro-3*H*-dinaphtho[2,1-*c*:1′,2′-*e*]azepine (1m)

To a solution of 4-bromo-2,6-di-*tert*-butylanisole[15] (178 mg, 0.595 mmol) in THF (1.00 mL) was added dropwise a solution of *n*-BuLi in hexane (1.60 M, 375 μL, 0.600 mmol) for 1 min at −78 °C and stirred for 20 min. After stirring for 20 min at −78 °C, a solution of **1e** (51.7 mg, 0.100 mmol) in THF (1.50 mL) was added to the reaction mixture at −78 °C. The reaction mixture was stirred for 1 h at −78 °C. Then, additional solution of *n*-BuLi in hexane (1.60 M, 375 μL, 0.600 mmol) was added to reaction mixture to destroy 1-bromobutane which was generated in the reaction. The reaction mixture was warmed up to room temperature and stirred for 1 h, and then water (10 mL) was added. The resulting mixture was extracted with $CHCl_3$ (20 mL × 3), dried over $MgSO_4$ and concentrated *in vacuo*. The purification of the crude product by

flash column chromatography on silica gel (eluent: Et_2O/MeOH = 30/1, v/v) gave **1m** (80.1 mg, 61.3 μmol, 61% yield) as a pale yellow solid.

^{1}H NMR (600 MHz, $CDCl_3$) δ 7.82 (bd, *J* = 5.4 Hz, 2H), 7.64 (d, *J* = 7.5 Hz, 2H), 7.32 (t, *J* = 7.5 Hz, 2H), 7.23-6.85 (m, 14H), 6.35 (bd, *J* = 5.4 Hz, 2H), 5.30 (d, *J* = 13.2 Hz, 2H), 3.67 (s, 6H), 6.03 (s, 6H), 3.33 (d, *J* = 13.2 Hz, 2H), 3.02 (bs, 1H), 2.94 (bs, 1H), 1.30 (s, 36H), 1.22 (s, 36H); ^{13}C NMR (150 MHz, $CDCl_3$) δ 158.7, 158.7, 143.2, 143.0, 143.0, 141.4, 140.2, 138.4, 131.8, 131.0, 130.4, 128.8, 127.2, 127.0, 126.5, 126.4, 125.7, 124.1, 109.2, 83.0, 64.4, 64.3, 46.7, 36.0 (2), 32.3, 32.2; IR (KBr) 2961, 1645, 1595, 1539, 1227, 1115, 1012, 887, 748 cm^{-1}; HRMS (ESI$^+$) caluculated for $C_{89}H_{113}N_2O_6$ $[M+H]^+$ 1305.8593, found 1305.8599; mp 202 ºC dec; $[\alpha]^{22}_D$ –218.6 (*c* 0.26, $CHCl_3$, (*S*)-configuration).

Synthesis of 1j´

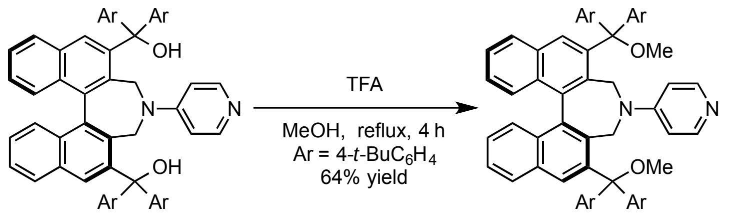

(*S*)-2,6-bis(bis(4-(*tert*-butyl)phenyl)(methoxy)methyl)-4-(pyridin-4-yl)-4,5-dihydro-3*H*-dinaphtho[2,1-*c*:1´,2´-*e*]azepine (1j´)

To a solution of **1j** (172 mg, 0.179 mmol) in MeOH (2.00 mL) was added trifluoroacetic acid [TFA] (89.2 μL, 1.20 mmol) at room temperature. The reaction mixture was heated to refluxed temperature and stirred for 4 h, and then 2 M aqueous NaOH (5 mL) was added. The resulting mixture was extracted with CH_2Cl_2 (5 mL × 3), dried over $MgSO_4$ and concentrated in *vacuo*. The purification of the crude product by flash column chromatography on silica gel (eluent: hexane/EtOAc = 1/1, v/v) gave **1j'** (113 mg, 0.114 mmol, 64% yield) as a pale yellow solid.

^{1}H NMR (400 MHz, $CDCl_3$) δ 8.13 (s, 2H), 8.09 (d, *J* = 5.4 Hz, 2H), 7.86 (d, *J* = 8.1 Hz, 2H), 7.49 (ddd, *J* = 8.1, 5.5, 2.5 Hz, 2H), 7.39–7.22 (m, 16H), 7.19 (d, *J* = 8.0 Hz, 4H), 6.08 (d, *J* = 5.4 Hz, 2H), 5.02 (d, *J* = 12.8 Hz, 2H), 3.12 (d, *J* = 12.8 Hz, 2H), 2.79 (s, 6H), 1.29 (s, 18H), 1,24 (s, 18H); ^{13}C NMR (100 MHz, $CDCl_3$, 40 ºC) δ 153.5, 149.5, 149.4, 149.0, 144.5, 140.3, 138.3, 138.2, 133.1, 132.1, 131.7, 131.5, 128.9, 128.1, 127.5, 126.7, 126.4, 126.1, 125.0, 124.8, 109.1, 87.8, 53.0, 45.1, 34.5, 31.5; IR (KBr) 2961, 1591, 1508, 1244, 1074, 750 cm^{-1}; HRMS (ESI$^+$) calculated for $C_{71}H_{77}N_2O_2$ $[M+H^+]$ 989.5980, found 989.5981; mp 221 ºC dec; $[\alpha]^{24}_D$ –78.7 (*c* 0.26, $CHCl_3$, (*S*)-configuration).

An overview for synthesis of catalyst 1n

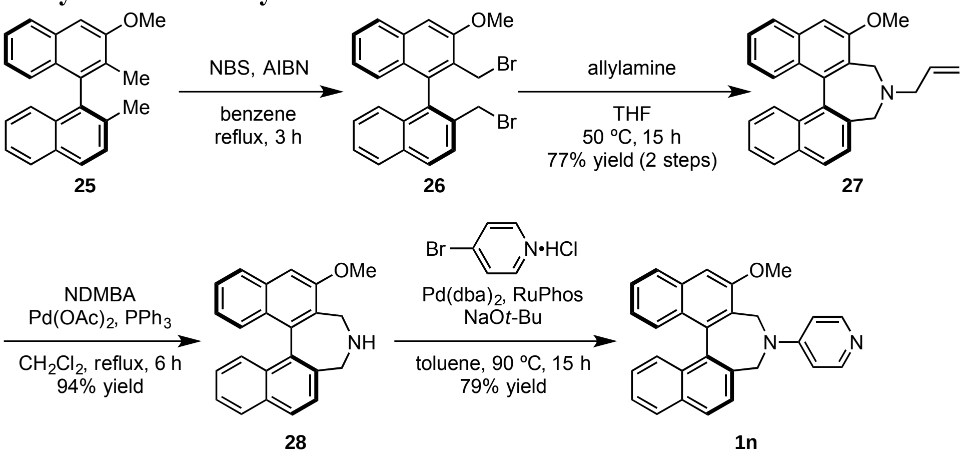

Synthesis of 27

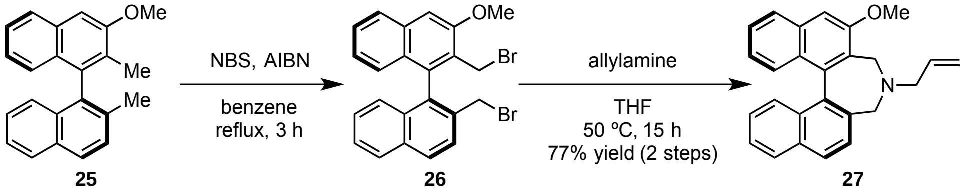

(*S*)-4-allyl-2-methoxy-4,5-dihydro-3*H*-dinaphtho[2,1-*c*:1′,2′-*e*]azepine (27)

To a suspension of **25**[7] (4.45 g, 14.3 mmol), NBS (5.58 g, 31.3 mmol) in benzene (71.0 mL) was added AIBN (234 mg, 1.43 mmol) at room temperature. The reaction mixture was heated to refluxed temperature and stirred for 3 h. After being cooled to room temperature, the reaction mixture was poured into water (100 mL) and extracted with EtOAc (100 mL × 3). The organic layer was washed with brine (50 mL × 3), dried over $MgSO_4$ and concentrated *in vacuo* to give the crude product of **26** (7.58 g) as a colorless solid. This crude product was directly used for the following reaction without further purification.

To a solution of **26** (4.03 g, 8.57 mmol) in THF (28.0 mL) was added allylamine (2.12 mL, 28.3 mmol) at room temperature. The reaction mixture was heated to 50 ºC and stirred for 15 h. After being cooled to room temperature, the reaction mixture was poured into water (50 mL). The resulting solution was extracted with toluene (30 mL × 3) and the organic layer was washed with water (10 mL × 3), dried over $MgSO_4$ and concentrated *in vacuo*. The purification of the crude product by flash column chromatography on silica gel (eluent: hexane/EtOAc = 5/1 to 2/1 to 1/1, v/v) gave **27** (2.12 g, 5.80 mmol, 77% yield based on **23**) as a pale yellow foam.

^{1}H NMR (600 MHz, $CDCl_3$) δ 7.99 (t, *J* = 8.1 Hz, 2H), 7.89 (d, *J* = 7.8 Hz, 1H), 7.60 (d, *J* = 7.8 Hz, 1H), 7.56 (d, *J* = 9.0 Hz, 1H), 7.51–7.41 (m, 3H), 7.35 (s, 1H), 7.30 (bt, *J* = 7.8 Hz, 1H), 7.15 (bt, *J* = 7.8 Hz, 1H), 6.15-6.05 (m, 1H), 5.31 (d, *J* = 17.4 Hz, 1H), 5.26 (d, *J* = 9.6 Hz, 1H), 4.46 (d, *J* = 12.6 Hz, 1H), 4.06 (s, 3H), 3.82 (d, *J* = 12.3 Hz, 1H), 3.30–3.22 (m, 2H), 3.12 (dd, *J* = 13.2, 6.0 Hz, 1H), 2.88 (d, *J* = 12.6 Hz, 1H); ^{13}C NMR (150 MHz, $CDCl_3$) δ 155.5, 137.2, 136.4, 134.9, 134.1, 133.6, 133.1 131.4, 128.4, 128.3, 127.8, 127.6, 127.5, 127.1, 126.9, 126.1, 126.0, 125.8, 125.4, 123.4, 117.7, 105.5, 58.8, 55.5, 55.0, 46.6; IR (KBr) 3069, 2807, 1508, 1334, 752; HRMS (FAB$^+$) calculated for $C_{26}H_{24}NO$ $[M+H]^+$ 366.1852, found 366.1848; $[\alpha]^{23}_D$ +375.9 (*c* 1.18, $CHCl_3$, (*S*)-configuration).

Synthesis of 28

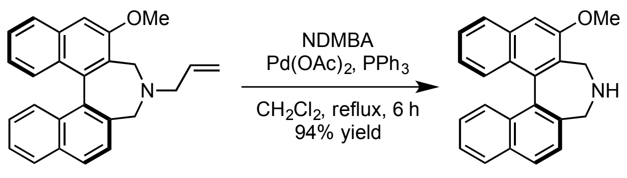

(*S*)-2-methoxy-4,5-dihydro-3*H*-dinaphtho[2,1-*c*:1′,2′-*e*]azepine (28)

To a solution of **27** (2.12 g, 5.80 mmol), NDMBA (2.72 g, 17.4 mmol) and PPh_3 (122 mg, 0.460 mmol) in CH_2Cl_2 (29.0 mL) was added $Pd(OAc)_2$ (26.0 mg, 0.115 mmol) at room temperature. The reaction mixture was heated to refluxed temperature, and stirred for 6 h. After being cooled to room temperature, the reaction mixture was added saturated aqueous $NaHCO_3$ (20 mL). The resulting solution was extracted with CH_2Cl_2 (30 mL × 3). The organic layer was washed with saturated aqueous $NaHCO_3$ (20 mL × 3), dried over $MgSO_4$ and concentrated *in vacuo*. The purification of the crude product by flash column chromatography on silica gel (eluent: EtOAc +1% Et_3N to EtOAc/MeOH = 10/1 to 1/1, v/v) gave **28** (1.77 g, 5.43 mmol, 94% yield) as a pale yellow foam.

^{1}H NMR (600 MHz, $CDCl_3$) δ 7.97 (d, *J* = 8.4 Hz, 1H), 7.94 (dd, *J* = 7.5, 1.5 Hz, 1H), 7.84 (bd, *J* = 8.4 Hz, 1H), 7.59 (d, *J* = 8.4 Hz, 1H), 7.48–7.43 (m, 2H), 7.42 (ddd, *J* = 8.1, 6.8, 1.4 Hz, 1H), 7.32 (bd, *J* = 8.4 Hz, 1H), 7.30 (s, 1H), 7.28–7.25 (m, 1H), 7.11 (ddd, *J* = 8.1, 6.8, 1.4 Hz, 1H), 4.53 (d, *J* = 12.9 Hz, 1H), 4.02 (s, 3H), 3.90 (d, *J* = 12.0 Hz, 1H), 3.53 (d, *J* = 12.0 Hz, 1H), 3.09 (d, *J* = 12.9 Hz, 1H), 2.60 (bs, 1H); ^{13}C NMR (150 MHz, $CDCl_3$) δ 154.9, 137.1, 135.0, 134.3, 134.2, 133.3, 131.5, 129.2, 128.4, 127.6, 127.5, 127.3, 127.2, 126.9, 126.6, 126.2, 126.0, 125.6, 123.6, 106.1, 55.6, 48.4, 39.6; IR (KBr) 3060, 3002, 2956, 2362, 1951, 1596, 1288, 752 cm^{-1}; HRMS (FAB$^+$) calculated for $C_{23}H_{20}NO$ $[M+H]^+$ 326.1539, found 326.1555; $[\alpha]^{21}_D$ +413.3 (*c* 1.14, $CHCl_3$, (*S*)-configuration).

Synthesis of 1n

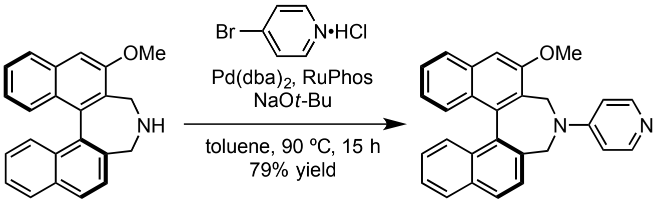

(*S*)-2-methoxy-4-(pyridin-4-yl)-4,5-dihydro-3*H*-dinaphtho[2,1-*c*:1′,2′-*e*]azepine (1n)

To a suspension of **28** (1.66 g, 5.06 mmol), 4-bromopyridine hydrochloride (1.97 g, 10.1 mmol), RuPhos (94.0 mg, 0.201 mmol) and NaO*t*-Bu (1.95 g, 20.2 mmol) in toluene (25.0 mL) was added $Pd(dba)_2$ (57.0 mg, 99.1 μmol) at room temperature. The reaction mixture was heated to 90 ºC and vigorously stirred for 15 h. Then, the reaction mixture was added water (30 mL) and filtered throught celite. The filtrate was extracted with toluene (30 mL × 3), washed with brine (20 mL × 3), dried over $MgSO_4$ and concentrated *in vacuo*. The purification of the crude product by flash column chromatography on silica gel (eluent: EtOAc to CH_2Cl_2 to CH_2Cl_2/MeOH = 2/1, v/v) gave **1n** (1.61 g, 4.00 mmol, 79% yield) as a pale brown solid.

^{1}H NMR (600 MHz, $CDCl_3$) δ 8.27 (dd, *J* = 5.3, 1.7 Hz, 2H), 7.95 (dd, *J* = 8.1, 2.1 Hz, 2H), 7.84 (d, *J* = 8.4 Hz, 1H), 7.55-7.47 (m, 3H), 7.45 (ddd, *J* = 8.1, 6.9, 1.2 Hz, 1H), 7.39 (d, *J* = 8.4 Hz, 1H), 7.31 (ddd, *J* = 8.4, 7.1, 1.4 Hz, 1H), 7.28 (s, 1H), 7.15 (ddd, *J* = 8.4, 7.1, 1.4 Hz, 1H), 6.79 (dd, *J* = 5.3, 1.7 Hz, 2H), 5.40 (d, *J* = 13.2 Hz, 1H), 4.59 (d, *J* = 12.6 Hz, 1H), 3.95 (s, 3H), 3.78 (d, *J* = 12.6 Hz, 1H), 3.41 (d, *J* = 13.2 Hz, 1H); ^{13}C NMR (150 MHz, $CDCl_3$) δ 154.7, 153.6, 150.1, 137.0, 135.2, 134.3, 133.4, 132.9, 131.5, 129.4, 128.4,

127.7, 127.6 (2), 127.3, 126.8, 126.6, 126.3, 126.1, 125.6, 123.9, 108.7, 106.3, 55.8, 50.8, 41.3; IR (KBr) 3047, 2363, 1595, 1507, 752; HRMS (FAB+) calculated for $C_{28}H_{23}N_2O$ $[M+H]^+$ 403.1804, found 403.1796; mp 223.7–224.6 ºC; $[\alpha]^{23}_D$ −338.4 (*c* 1.11, $CHCl_3$, (*S*)-configuration).

An overview for synthesis of catalyst 1o

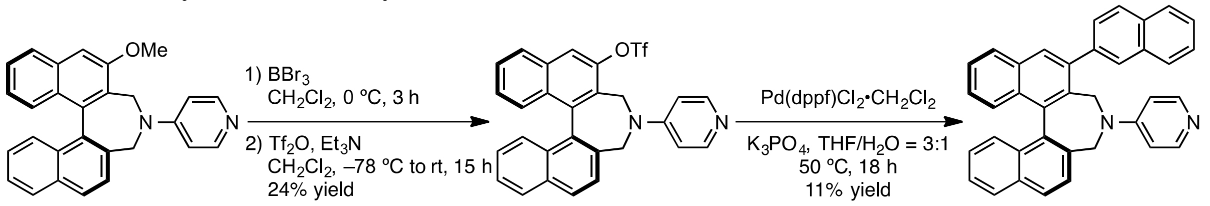

1n **29** **1o**

Synthesis of 29

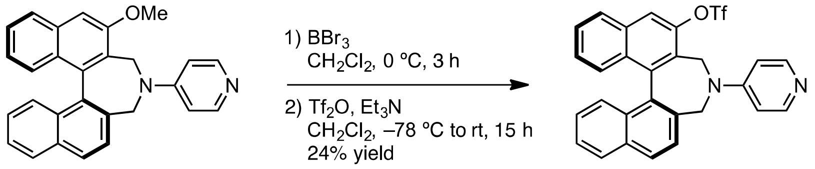

1n **29**

(*S*)-4-(pyridin-4-yl)-4,5-dihydro-3*H*-dinaphtho[2,1-*c*:1′,2′-*e*]azepin-2-yl trifluoromethanesulfonate (29)

To a solution of **1n** (256 mg, 0.636 mmol) in CH_2Cl_2 (6.40 mL) was added a solution of BBr_3 in CH_2Cl_2 (1.00 M, 1.50 mL, 1.50 mmol) at 0 ºC. The reaction mixture was stirred for 3 h at 0 ºC, then poured into MeOH (10 mL) and concentrated *in vacuo*. The residue was dissolved in CH_2Cl_2 (6.40 mL) and Et_3N (0.270 mL, 1.92 mmol) was added. To this solution was added Tf_2O (0.130 mL, 0.773 mmol) at −78 ºC. The reaction mixture was warmed up to room temperature and stirred for 15 h, then water (15 mL) was added. The resulting solution was extracted with CH_2Cl_2 (15 mL × 3), washed with saturated aqueous $NaHCO_3$ (20 mL × 3), dried over $MgSO_4$ and concentrated *in vacuo*. The purification of the crude product by flash clumn chromatography on silica gel (eluent: EtOAc/MeOH = 50/1, v/v) gave **29** (80.2 mg, 0.154 mmol, 24% yield) as a pale yellow foam.

^{1}H NMR (600 MHz, $CDCl_3$) δ 8.31 (d, *J* = 6.3 Hz, 2H), 8.04 (d, *J* = 8.4 Hz, 1H), 8.00 (t, *J* = 6.6 Hz, 2H), 7.95 (s, 1H), 7.62 (t, *J* = 6.6 Hz, 1H), 7.59–7.51 (m, 3H), 7.44 (d, *J* = 8.4 Hz, 1H), 7.42–7.35 (m, 2H), 6.81 (d, *J* = 6.3 Hz, 2H), 5.15 (d, *J* = 13.5 Hz, 1H), 4.68 (d, *J* = 12.6 Hz, 1H), 3.82 (d, *J* = 12.6 Hz, 1H), 3.69 (d, *J* = 13.5 Hz, 1H); ^{13}C NMR (150 MHz, $CDCl_3$) δ 153.0, 150.0, 144.6, 139.1, 133.4, 133.3, 132.9, 132.7, 131.0, 130.4, 130.2, 128.5, 127.8, 127.7, 127.4, 127.2, 127.0, 126.7, 126.3, 125.7, 120.2, 118.6 (q, *J* = 319.0 Hz), 108.6, 50.3, 42.5; IR (KBr) 1593, 1508, 1421, 1215, 810, 752 cm^{-1}; HRMS (FAB+) calculated for $C_{28}H_{20}F_3N_2O_3S$ $[M+H]^+$ 521.1141, found 521.1124; $[\alpha]^{21}_D$ –220.4 (*c* 0.27, $CHCl_3$, (*S*)-configuration).

Synthesis of 1o

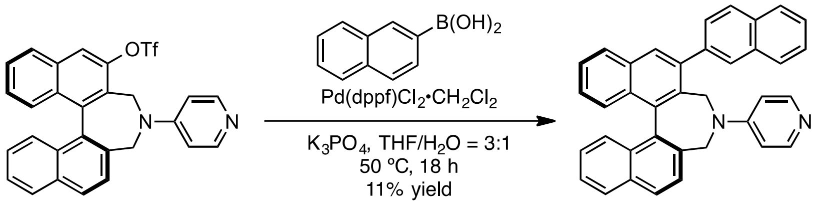

29 **1o**

To a suspension of **29** (26.1 mg, 0.501 mmol), 2-naphthaleneboronic acid (8.60 mg, 50.0 μmol) and K_3PO_4 (43.4 mg, 0.204 mmol) in THF (0.500 mL) and water (0.170 mL) was added $Pd(dppf)Cl_2·CH_2Cl_2$ (3.30 mg,

4.04 μmol) at room temperature. The reaction mixture was heated to 50 ºC and stirred for 18 h. After cooled to room temperature, the reaction mixture was diluted with CH_2Cl_2 (2 mL). The resulting solution was extracted with CH_2Cl_2 (5 mL × 3), dried over $MgSO_4$ and concentrated *in vacuo*. The purification of the crude product by flash column chromatography on silica gel (eluent: EtOAc to EtOAc + 3% Et_3N) gave **1o** (2.70 mg, 5.41 μmol, 11% yield) as a colorless solid.

^{1}H NMR (600 MHz, $CDCl_3$) δ 8.05 (s, 1H), 7.99–7.97 (m, 6H), 7.89 (d, *J* = 7.8 Hz, 2H), 7.58–7.49 (m, 8H), 7.36–7.31 (m, 3H), 6.36 (bs, 2H), 4.87 (d, *J* = 12.9 Hz, 1H), 4.71 (d, *J* = 12.6 Hz, 1H), 4.06 (d, *J* = 12.6 Hz, 1H), 3.59 (d, *J* = 12.9 Hz, 1H); ^{13}C NMR (150 MHz, $CDCl_3$) δ 153.0, 149.4, 139.9, 138.2, 136.0, 135.3, 133.5, 133.3, 132.9, 132.6, 132.5, 131.5, 130.8, 130.2, 129.5, 128.8, 128.5, 128.5, 128.2, 127.8, 127.7, 127.6, 127.4, 126.7, 126.6, 126.5, 126.4, 126.3, 126.1, 108.5, 50.7, 46.3; IR (KBr) 3651, 3049, 2963, 2365, 1506 cm^{-1}; HRMS (FAB$^+$) calculated for $C_{37}H_{27}N_2$ $[M+H]^+$ 499.2168, found 499.2168; mp 181 ºC dec; $[\alpha]^{22}_D$ –157.6 (*c* 0.27, $CHCl_3$, (*S*)-configuration).

An overview for synthesis of catalyst 1p

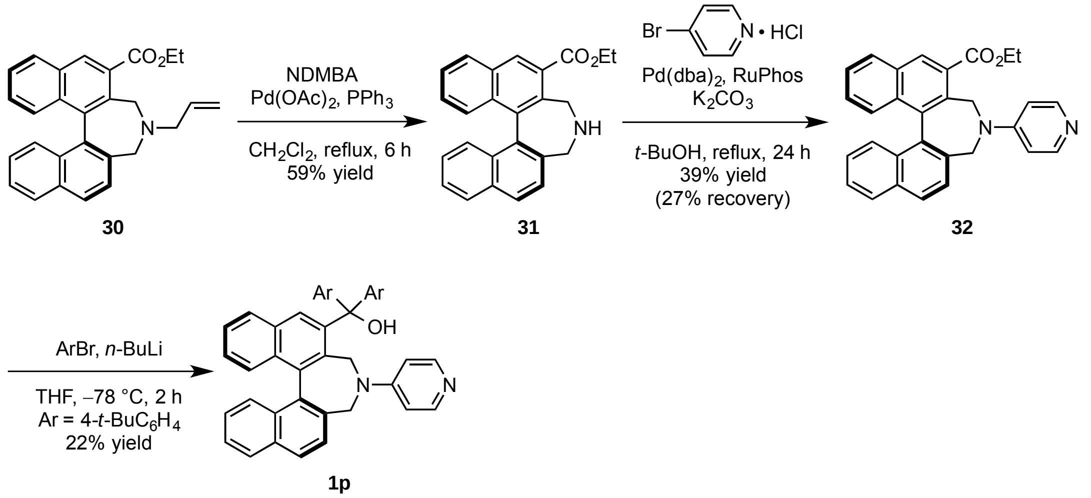

Synthesis of 31

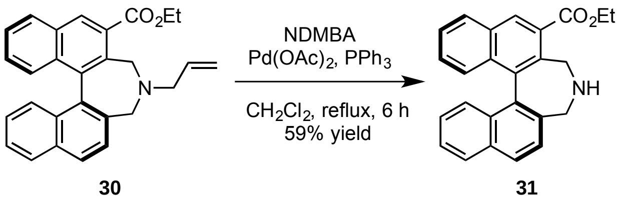

(*S*)-Ethyl 4,5-dihydro-3H-dinaphtho[2,1-*c*:1′,2′-*e*]azepine-2-carboxylate (31)

To a solution of **30**[6] (2.52 g, 6.17 mmol), NDMBA (1.94 g, 12.4 mmol) and Ph_3P (324 mg, 1.23 mmol) in CH_2Cl_2 (20.6 mL) was added $Pd(OAc)_2$ (69.6 mg, 0.310 mmol) at room temperature. The reaction mixture was heated to refluxed temperature and stirred for 6 h, then water (20 mL) was added. The resulting solution was extracted with CH_2Cl_2 (20 mL × 3). The organic layer was washed with saturated aqueous $NaHCO_3$ (20 mL × 3), dried over $MgSO_4$ and concentrated in vacuo. The purification of the crude product by flash column chromatography on silica gel (eluent: Et_2O to Et_2O/MeOH = 20/1 to 10/1, v/v) gave **31** (1.35 g, 3.68 mmol, 59% yield) as a pale yellow foam.

^{1}H NMR (400 MHz, $CDCl_3$) NMR (400 MHz, $CDCl_3$) δ 8.55 (s, 1H), 8.00 (t, *J* = 8.1 Hz, 2H), 7.95 (d, *J* = 8.1 Hz, 1H), 7.63 (d, *J* = 8.1 Hz, 1H), 7.52–7.42 (m, 2H), 7.41–7.29 (m, 3H), 7.29–7.22 (m, 1H), 4.63 (d, *J* = 13.3 Hz, 1H), 4.56–4.43 (m, 2H), 3.86 (d, *J* = 11.2 Hz, 1H), 3.50 (d, *J* = 11.2 Hz, 1H), 3.26 (d, *J* = 13.3 Hz, 1H), 2.53 (bs, 1H), 1.50 (t, *J* = 7.1 Hz, 3H); ^{13}C NMR (100 MHz, $CDCl_3$) δ 168.1, 136.6, 135.0, 134.6, 133.9,

133.0, 132.6, 131.6 (2), 131.2, 129.2 (2), 128.3, 127.7, 127.4, 127.3, 127.1, 126.1, 125.8, 125.4, 61.3, 48.4, 44.3, 14.4; IR (KBr) 3053, 2953, 2384, 2349, 1717, 1294 cm^{-1}; HRMS (FAB$^+$) calculated for $C_{25}H_{22}NO_2$ $[M+H]^+$ 368.1639, found 368.1637; $[\alpha]^{23}_{D}$ +413.7 (*c* 1.02, CH_2Cl_2, (*S*)-configuration).

Synthesis of 32

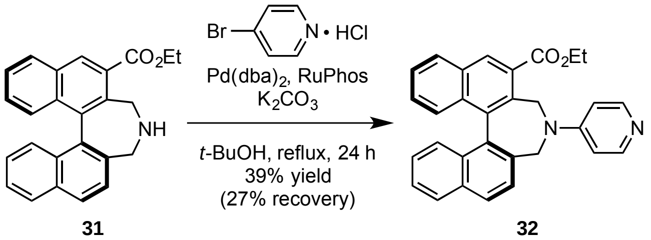

31 32

(*S*)-Ethyl 4-(pyridin-4-yl)-4,5-dihydro-3H-dinaphtho[2,1-*c*:1′,2′-*e*]azepine-2-carboxylate (32)

To a suspension of **31** (1.30 g, 3.54 mmol), 4-bromopyridine hydrochloride (1.37 g, 7.07 mmol), RuPhos (132 mg, 0.283 mmol) and potassium carbonate (2.94 g, 21.2 mmol) in *t*-BuOH (35.4 mL) was added $Pd(dba)_2$ (81.4 mg, 0.142 mmol) at room temperature. The reaction mixture was heated to refluxed temperature and vigorously stirred for 24 h, and then subsequently thoroughly washed with CH_2Cl_2. After filtrate was concentrated *in vacuo*, the purification of the crude product by flash column chromatography on silica gel (eluent: Et_2O to Et_2O/MeOH = 20/1 to 10/1 to 4/1, v/v) gave **32** (616 mg, 1.39 mmol, 39% yield) as a pale brown solid, along with recovered of **31** (357 mg, 0.972 mmol, 27% yield).

^{1}H NMR (400 MHz, $CDCl_3$) NMR (400 MHz, $CDCl_3$) δ 8.55 (s, 1H), 8.25 (d, *J* = 5.3 Hz, 2H), 7.99 (d, *J* = 8.0 Hz, 1H), 7.93 (d, *J* = 8.0 Hz, 2H), 7.54–7.23 (m, 7H), 6.71 (d, *J* = 5.3 Hz, 2H), 5.96 (d, *J* = 13.3 Hz, 1H), 4.52 (d, *J* = 12.4 Hz, 1H), 4.46–4.30 (m, 2H), 3.72 (d, *J* = 12.4 Hz, 1H), 3.62 (d, *J* = 13.3 Hz, 1H), 1.35 (t, *J* = 7.1 Hz, 3H); ^{13}C NMR (100 MHz, $CDCl_3$) δ 168.0, 153.3, 150.0, 137.0, 134.6, 133.3, 132.7, 132.6, 132.4, 131.8, 131.3, 131.2, 129.6, 129.3, 128.4, 128.3, 128.1, 127.5, 127.2 (2), 126.8, 126.4, 126.0, 108.7, 61.7, 50.5, 44.1, 14.3; IR (KBr) 2382, 1717, 1591, 1508, 1296, 1244 cm^{-1}; HRMS (FAB$^+$) calculated for $C_{30}H_{24}N_2O_2$ $[M+H]^+$ 445.1905, found 445.1896; mp 86.6–88.0 °C; $[\alpha]^{22}_{D}$ –243.4 (*c* 0.585, CH_2Cl_2, (*S*)-configuration).

Synthesis of 1p

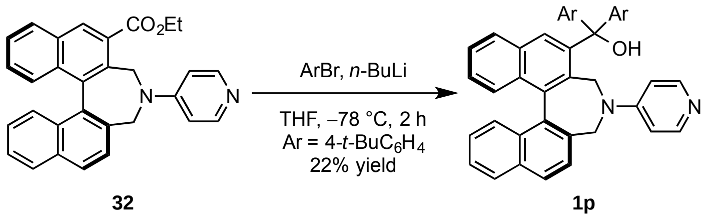

32 1p

(*S*)-2-(bis(4-(*tert*-butyl)phenyl)(methoxy)methyl)-4-(pyridin-4-yl)-4,5-dihydro-3*H*-dinaphtho[2,1-*c*:1′,2′-*e*]azepine (1p)

To a solution of 1-bromo-4-*tert*-butylbenzene (104 benzene of thoxy)methyl)-4-(pyridin-4-yl)-4,5-pwise a solution of *n*-BuLi in hexane (1.63 M, 375 μL) at –78 ºC and stirred for 20 min. After stirring for 20 min at −78 ºC, a solution of **32** (88.9 mg, 0.200 mmol) in THF (2.00 mL) was added to reaction mixture at −78 °C. The reaction mixture was stirred for 2 h at −78 °C. Then, water (5 mL) was added to the reaction mixture and extracted with $CHCl_3$ (10 mL × 3), dried over $MgSO_4$, and concentrated *in vacuo*. The purification of the crude product by flash column chromatography on silica gel (eluent: $CHCl_3$/MeOH = 20/1 to 10/1, v/v) and

the resulting solid was subsequently thoroughly washed with EtOAc. After filtration and drying of the solid, **1p** (29.1 mg, 43.6 μmol, 22% yield) was obtained as a pale yellow solid.

^{1}H NMR (400 MHz, CDCl$_3$) δ 8.07 (d, J = 6.6 Hz, 2H), 7.92 (d, J = 8.2 Hz, 1H), 7.89 (d, J = 8.2 Hz, 1H), 7.72 (d, J = 8.2 Hz, 1H), 7.52–7.30 (m, 9H), 7.28–7.21 (m, 5H), 7.16 (d, J = 8.2 Hz, 2H), 6.41 (d, J = 6.6 Hz, 2H), 5.23 (d, J = 12.4 Hz, 1H), 4.63 (d, J = 13.3 Hz, 1H), 3.89 (d, J = 13.3 Hz, 1H), 3.49 (bs, 1H), 3.15 (d, J = 12.4 Hz, 1H), 1.32 (s, 9H), 1.26 (s, 9H); ^{13}C NMR (100 MHz, CDCl$_3$) δ 153.1, 150.2, 150.0, 149.1, 144.7, 143.8, 143.1, 138.4, 134.8, 133.3, 132.9, 132.2, 132.0, 131.5, 130.9, 130.3, 129.4, 129.0, 128.4, 127.8, 127.5, 127.3, 126.6, 126.4, 126.2, 125.9, 125.1, 124.7, 108.5, 82.9, 50.3, 45.7, 34.6, 34.4, 31.5 (2); IR (KBr) 2961, 2384, 1607, 1506, 1236, 995 cm^{-1}; HRMS (FAB$^+$) calculated for $C_{48}H_{47}N_2O$ [M+H]$^+$ 667.3677, found 667.3709; mp 222 °C dec; $[\alpha]^{25}_D$ –340.5 (c 0.305, CHCl$_3$, (S)-configuration).

Synthesis of substrate 3c, 3q and 3r

Synthesis of 3c

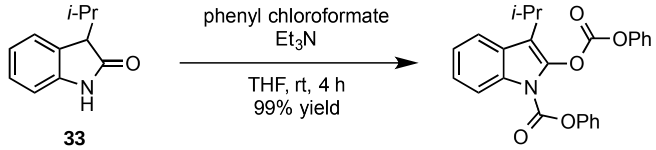

33

9c

Phenyl 3-isopropyl-2-((phenoxycarbonyl)oxy)-1*H*-indole-1-carboxylate (3c)

To a solution of **33**[8] (262 mg, 1.50 mmol) and triethylamine (850 μL, 6.10 mmol) in THF (5.00 mL) was added phenyl chloroformate (753 μL, 6.00 mmol) at room temperature. The reaction mixture was stirred for 4 h at room temperature, and then 1 M aqueous HCl (10 mL) was carefully added. The resulting solution was extracted with EtOAc (10mL × 3), dried over MgSO$_4$ and concentrated *in vacuo*. The purification of the crude product by flash column chromatography on silica gel (eluent: hexane/Et$_2$O = 5/1, v/v) gave **9c** (619 mg, 1.49 mmol, 99% yield) as a colorless solid.

^{1}H NMR (400 MHz, CDCl$_3$) δ 8.19 (d, J = 7.6 Hz, 1H), 7.66 (d, J = 7.2 Hz, 1H), 7.47 (t, J = 7.9 Hz, 2H), 7.39–7.29 (m, 8H), 7.11 (d, J = 7.9 Hz, 2H), 3.27 (quin, J = 7.0 Hz, 1H), 1.46 (d, J = 7.0 Hz, 6H); ^{13}C NMR (100 MHz, CDCl$_3$) δ 151.4, 150.9, 149.9, 148.6, 136.0, 132.4, 129.7, 129.6, 126.7, 126.6, 126.5, 124.9, 123.6, 121.6, 120.7, 119.9, 115.7, 115.7, 24.5, 21.6; IR (KBr) 2963, 1790, 1749, 1456, 1364, 1120, 748 cm^{-1}; HRMS (FAB$^+$) calculated for $C_{25}H_{22}NO_5$ [M+H]$^+$ 416.1492, found 416.1505; mp 75.9–76.6 ºC.

Synthesis of 9q

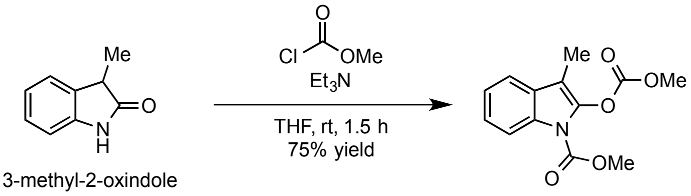

3-methyl-2-oxindole

9q

Methyl 2-((methoxycarbonyl)oxy)-3-methyl-1*H*-indole-1-carboxylate (9q)

To a solution of 3-methyl-2-oxindole (1.47 g, 9.99 mmol) and triethylamine (5.60 mL, 40.2 mmol) in THF (30.0 mL) was added methyl chloroformate (3.10 mL, 40.1 mmol) at room temperature. The reaction mixture was stirred for 1.5 h at room temperature, and then 1 M aqueous HCl (10 mL) was carefully added. The resulting solution was extracted with EtOAc (30 mL× 3), dried over MgSO$_4$ and concentrated *in vacuo*. The

purification of the crude product by flash column chromatography on silica gel (eluent: hexane/Et_2O = 3/1 to 1/1, v/v) gave **9q** (1.97 g, 7.48 mmol, 75% yield) as a orange solid.

^{1}H NMR (600 MHz, $CDCl_3$) δ 8.02 (d, *J* = 7.7 Hz, 1H), 7.47 (d, *J* = 7.7 Hz, 1H), 7.33 (td, *J* = 7.7, 1.6 Hz, 1H), 7.28 (td, *J* = 7.7, 1.0 Hz, 1H), 4.01 (s, 3H), 3.98 (s, 3H), 2.16 (s, 3H); ^{13}C NMR (150 MHz, $CDCl_3$) δ 153.3, 150.9, 137.6, 132.0, 128.1, 124.8, 123.4, 119.0, 115.4, 105.4, 56.3, 53.9, 7.0; IR (KBr) 2961, 1775, 1732, 1653, 1460, 1356, 1273, 1040, 934, 754 cm^{-1}; HRMS (FAB$^+$) calculated for $C_{13}H_{14}NO_5$ [M+H]$^+$ 264.0866, found 264.0877; mp 51.3–51.9 ºC.

Synthesis of 9r

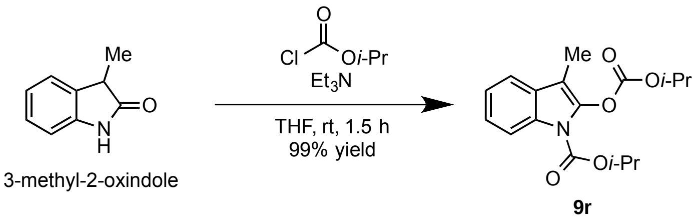

Isopropyl 2-((isopropoxycarbonyl)oxy)-3-methyl-1*H*-indole-1-carboxylate (9r)

To a solution of 3-methyl-2-oxindole (1.47 g, 9.99 mmol) and triethylamine (5.60 mL, 40.2 mmol) in THF (30.0 mL) was added isopropyl chloroformate (5.50 mL, 40.0 mmol) at room temperature. The reaction mixture was stirred for 1.5 h at room temperature, and then 1 M aqueous HCl (10 mL) was carefully added. The resulting solution was extracted with EtOAc (30 mL × 3), dried over $MgSO_4$ and concentrated *in vacuo*. The purification of the crude product by flash column chromatography on silica gel (eluent: hexane/Et_2O = 6/1, v/v) gave **9r** (3.16 g, 9.90 mmol, 99% yield) as a pale yellow oil.

^{1}H NMR (600 MHz, $CDCl_3$) δ 8.07 (d, *J* = 7.8 Hz, 1H), 7.46 (d, *J* = 7.8 Hz, 1H), 7.31 (td, *J* = 7.8, 1.6 Hz, 1H), 7.28–7.22 (m, 1H), 5.26 (sep, *J* = 6.1 Hz, 1H), 5.02 (sep, *J* = 6.1 Hz, 1H), 2.15 (s, 3H), 1.43 (d, *J* = 6.2 Hz, 6H), 1.42 (d, *J* = 6.2 Hz, 6H); ^{13}C NMR (150 MHz, $CDCl_3$) δ 152.1, 150.0, 137.9, 132.2, 128.1, 124.5, 123.1, 118.8, 115.5, 74.3, 71.5, 22.0, 21.8, 7.0; IR (KBr) 2984, 1755, 1708, 1651, 1456, 1099, 912, 752 cm^{-1}; HRMS (FAB$^+$) calculated for $C_{12}H_{22}NO_5$ [M+H]$^+$ 320.1492, found 320.1487.

General procedure for the Steglich rearrangement of oxindole derivatives

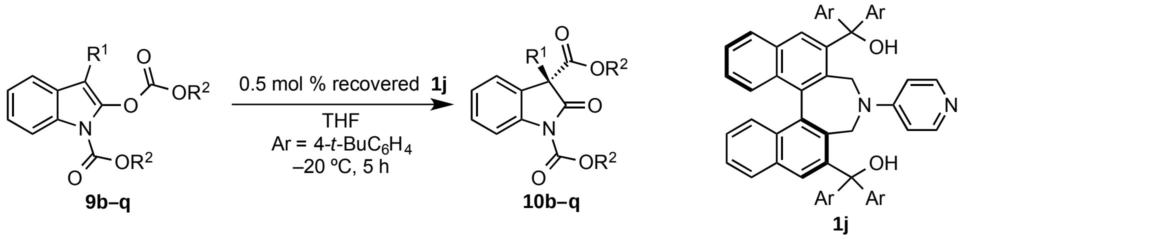

When the catalytic amounts was less than 0.5 mol %, a solution of the catalyst in THF (50.0 mM) was prepared in advance and this stock solution was used the following reaction.

To a solution of substrate **9b−r** in THF was added a catalyst or a solution of the catalyst at −20 ºC. The reaction mixture was stirred for 5 h and then 1 M aqueous HCl was added. The resulting mixture was extracted with EtOAc, dried over $MgSO_4$ and concentrated *in vacuo*. The purification of the crude product by flash column chromatography on a short pad of silica gel (eluent: hexane/Et_2O = 1/1, v/v) gave the corresponding product **10b−m** (>98% yield). The enantiomeric ratio was determined by chiral HPLC analysis.

The absolute configuration of the product **10j** was assigned to be (*S*)–configuration according to Vedejs's

paper[9] and **10b**, **10d–l** and **10n–p** were assigned to be (*S*)–configuration according to our previous paper (**10k**; (*R*)-configuration)[10]. For product **10c**, the absolute configuration was determined by single crystal X-ray structure analysis (Supplementary Figure 67).

Steglich rearrangement on multi-gram scale (Figure 4b)

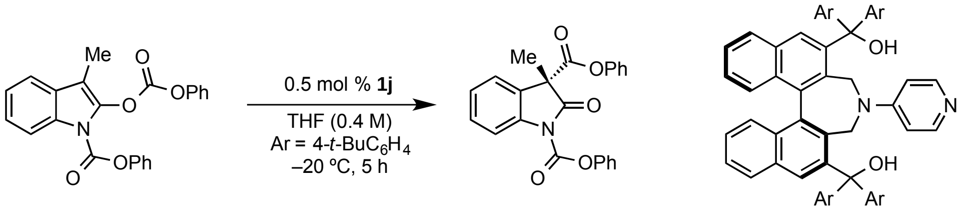

9a 15.00 g (40.2 mmol)

10a **14.99 g (40.2 mmol)** >98% yield (plug filtration) with 98:2 er 95% recovery of **1j**

1j

We carried out large scale reaction (15 g, 40.2 mmol). The reaction proceeded smoothly and **10a**[9] was obtained in >98% yield (14.99 g, 40.2 mmol) along with 95% recovery of catalyst **1j** after simple plug filtration (eluent: hexane/Et_2O = 1/1 for obtaining **10a**; then Et_2O/MeOH = 5/1 for catalyst recovery). ^{1}H NMR spectra of **10a** clearly indicated that simple plug filtration was enough for obtaining analytically pure **10a**. The procedure was as follows.

To a solution of **9a**[9] (15.0 g, 42.0 mmol) in THF (95.5 mL) was added solution of **1j** (193.2 mg, 0.2 mmol) in THF (5.00 mL) at –20 ºC. The reaction mixture was stirred for 5 h at –20 ºC, then monitored by TLC. After checking of disappearance of **9a**, the reaction solution was transferred to other flask using Et_2O to wash the reaction vessel and concentrated in *vacuo*. The purification of the crude product by flash column chromatography on a short pad of silica gel (eluent: hexane/Et_2O = 1/1, v/v) gave the **10a** (15.0 g, 42.0 mmol, >98% yield) and the catalyst **1j** was recovered by elution of polar solvent (Et_2O/MeOH = 5/1, v/v): enantiomeric ratio was determined by HPLC with DAICEL CHIRALPAK® AS-3 (hexane/*i*-PrOH = 85/25, v/v, flow rate = 0.30 mL/min, 30 °C, UV = 254 nm), T_R = 13.0 min (major) and T_R = 14.0 min (minor), 98:2 er (Supplementary Figure 40); ^{1}H NMR (400 MHz, $CDCl_3$) δ 8.10 (d, J = 7.6 Hz, 1H), 7.51–7.27 (m, 10H), 7.22 (d, J = 7.6 Hz, 1H), 6.97 (d, J = 8.0 Hz, 2H), 1.90 (s, 3H); ^{13}C NMR (100 MHz, $CDCl_3$) δ 172.4, 167.6, 150.2, 150.1, 149.3, 139.1, 129.9, 129.6, 129.5, 128.6, 126.6, 126.4, 125.7, 123.0, 121.5, 121.1, 115.9, 55.9, 20.9; IR (KBr) 3069, 1802, 1741, 1724, 1591, 1477, 1341, 1209, 1092, 959, 758 cm^{-1}; $[\alpha]^{21}_{D}$ +74.1 (*c* 1.03, CH_2Cl_2, 98:2 er, (*S*)-configuration) [lit.[10] $[\alpha]^{20}_{D}$ +34.0 (*c* 0.16, $CHCl_3$, 92:8 er, (*S*)-configuration)].

The Steglich rearrangement with the recovered catalyst 1j

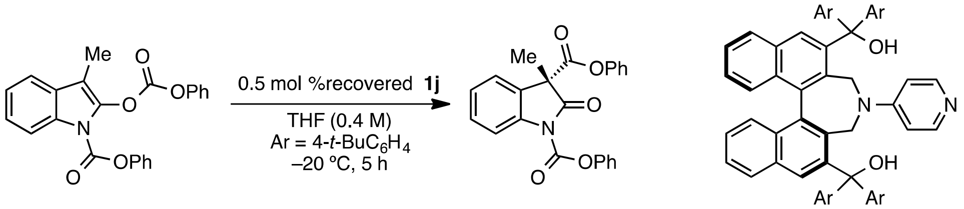

9a 37.4 mg (0.100 mmol)

10a **36.9 mg (98.8 µmol)** 99% yield (plug filtration) with 98:2 er

1j

Furthermore, the same reaction (37.4 mg of **9a**, 0.1 mmol scale) was carried out with recovered catalyst **1j** from large scale reaction, and the reaction proceeded smoothly to give desired product in 99% yield with

98:2 er. ^{1}H NMR spectra of **10a** after simple plug filtration showed analytically pure **10a**. It was indicated that our catalyst can be used at least twice without loss of any catalytic activity and enantioselectivity.

To a solution of recovered **9a** (37.4 mg, 0.100 mmol) in THF (0.250 ml) was added solution of **1j** (50 mM, 10 µL, 2.50 µmol) in THF at –20 ºC. The reaction mixture was stirred for 5 h at –20 ºC and then 1 M aqueous HCl was added. The resulting mixture was extracted with EtOAc, dried over $MgSO_4$ and concentrated *in vacuo*. The purification of the crude product by flash column chromatography on a short pad of silica gel (eluent: hexane/Et_2O = 1/1, v/v) gave the **10a** (36.9 mg, 98.9 µmol, 99% yield): enantiomeric ratio was determined by HPLC with DAICEL CHIRALPAK® AS-3 (hexane/*i*-PrOH = 85/25, v/v, flow rate = 0.30 mL/min, 30 °C, UV = 254 nm), T_R = 13.0 min (major) and T_R = 14.0 min (minor), 98:2 er (Supplementary Figure 41). Other analytical data was shown as above.

Analytical data for products 9b−q of Steglich rearrangement

(*S*)-Diphenyl 3-ethyl-2-oxoindoline-1,3-dicarboxylate (10b)[10]

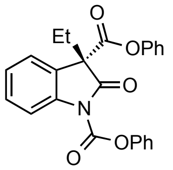

10b

According to general procedure, substrate **9b**[10] (80.9 mg, 0.202 mmol) with a solution of catalyst **1j** (20.0 µL, 50.0 mM in THF, 1.00 µmol) in THF (0.500 mL) at −20 ºC gave a yellow solid (80.2 mg, 0.200 mmol, >98% yield): enantiomeric ratio was determined by HPLC with DAICEL CHIRALPAK AD-H® (hexane/*i*-PrOH = 97.5/2.5, v/v, flow rate = 1.00 mL/min, 30 °C, UV = 254 nm), T_R = 21.8 min (minor) and T_R = 26.6 min (major), 98:2 er (Supplementary Figure 55); ^{1}H NMR (400 MHz, $CDCl_3$) δ 8.05 (d, *J* = 8.4 Hz, 1H), 7.50–7.39 (m, 4H), 7.38–7.27 (m, 6H), 7.22 (t, *J* = 7.2 Hz, 1H), 6.98 (d, *J* = 8.8 Hz, 2H), 2.56 (dq, *J* =13.9, 7.4 Hz, 2H), 2.42 (dq, *J* = 13.9, 7.4 Hz, 2H), 0.86 (t, *J* = 7.4 Hz, 3H); ^{13}C NMR (100 MHz, $CDCl_3$) δ 171.8, 167.5, 150.3, 150.2, 149.4, 140.0, 130.0, 129.8, 129.6, 126.7, 126.6, 126.5, 125.8, 123.3, 121.6, 121.3, 115.9, 60.9, 28.4, 8.3; IR (KBr) 3078, 2970, 1811, 1784, 1749, 1722, 1591, 1476, 1206 cm^{-1}; HRMS (FAB^+) calculated for $C_{24}H_{20}NO_5$ $[M+H]^+$ 402.1335, found 402.1363; mp 133.1–133.7 ºC; $[\alpha]^{20}_{D}$ +60.3 (*c* 0.51, CH_2Cl_2, 98:2 er, (*S*)-configuration) [lit.[10] $[\alpha]^{24}_{D}$ +19.8 (*c* 0.49, $CHCl_3$, 91:9 er, (*S*)-configuration)].

(*S*)-Diphenyl 3-isopropyl-2-oxoindoline-1,3-dicarboxylate (10c)

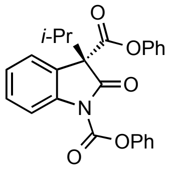

10c

According to general procedure, substrate **9c** (41.1 mg, 0.0989 mmol) with catalyst **1j** (3.00 mg, 3.12 µmol) in THF (0.250 mL) at 25 ºC gave a colorless solid (40.9 mg, 0.0984 mmol, >98% yield): enantiomeric ratio was determined by HPLC with DAICEL CHIRALPAK AD-H® (hexane/*i*-PrOH = 99.3/0.7, v/v, flow rate = 1.50 mL/min, 30 °C, UV = 254 nm), T_R = 13.7 min (minor) and T_R = 22.3 min (major), 96:4 er (Supplementary Figure 56); ^{1}H NMR (400 MHz, $CDCl_3$) δ 8.03 (d, *J* = 8.0 Hz, 1H), 7.51 (d, *J* = 7.6 Hz, 1H), 7.50–7.19 (m, 10H), 7.02 (d, *J* = 7.2 Hz, 2H), 2.96 (sep, *J* = 7.1 Hz, 1H), 1.31 (d, *J* = 7.1 Hz, 3H), 0.94 (d, *J* = 7.1 Hz, 3H); ^{13}C NMR (100 MHz, $CDCl_3$) δ 170.5, 167.2, 150.4, 150.2, 149.4, 139.7, 129.8, 129.7, 129.6, 126.7, 126.6, 126.5, 125.6, 123.9, 121.7, 121.4, 115.5, 63.8, 36.3, 17.5, 17.2; IR (KBr) 2968, 1811, 1748, 1489, 1207, 761 cm^{-1}; HRMS (ESI^+) calculated for $C_{25}H_{22}NO_5$ $[M+H]^+$ 416.1486, found 416.1489; mp 153.1–154.1 ºC; $[\alpha]^{24}_{D}$ +30.0 (*c* 0.62, CH_2Cl_2, 96:4 er, (*S*)-configuration).

(*S*)-Diphenyl 3-benzyl-2-oxoindoline-1,3-dicarboxylate (10d)[10]

According to general procedure, substrate **9d**[10] (92.6 mg, 0.200 mmol) with a solution of catalyst **1j** (20.0 µL, 50.0 mM in THF, 1.00 µmol) in THF (0.500 mL) at −20 ºC gave a pale yellow solid (91.6 mg, 0.198 mmol, >98% yield): enantiomeric ratio was determined by HPLC with DAICEL CHIRALPAK AD-H®

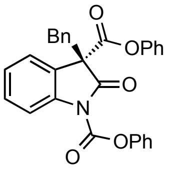

10d

(hexane/*i*-PrOH = 90/10, v/v, flow rate = 1.00 mL/min, 30 °C, UV = 254 nm), T_R = 13.1 min (major) and T_R = 15.2 min (minor), 96:4 er (Supplementary Figure 57); ^{1}H NMR (400 MHz, $CDCl_3$) δ 7.78 (d, *J* = 7.8 Hz, 1H), 7.52 (dd, *J* = 7.8, 1.2 Hz, 1H), 7.48–7.05 (m, 13H), 7.04–6.99 (m, 2H), 6.93 (d, *J* = 6.8 Hz, 2H), 3.78 (d, *J* = 13.6 Hz, 1H), 3.68 (d, *J* = 13.6 Hz, 1H); ^{13}C NMR (100 MHz, $CDCl_3$) δ 171.3, 167.4, 150.4, 150.1, 148.9, 139.9, 133.4, 130.2, 130.1, 129.7, 129.6, 128.2, 127.5, 126.7, 126.6, 126.3, 125.5, 123.7, 121.6, 121.3, 115.8, 61.8, 41.1; IR (KBr) 3062, 1809, 1778, 1747, 1497, 1350, 1184, 744 cm^{-1}; HRMS (FAB^+) calculated for $C_{29}H_{22}NO_5$ $[M+H]^+$ 464.1492, found 464.1473; mp 147.9–148.3 ºC; $[\alpha]^{24}_D$ +37.9 (*c* 0.45, CH_2Cl_2, 96:4 er, (*S*)-configuration) [lit.[10] $[\alpha]^{24}_D$ +13.9 (*c* 0.50, $CHCl_3$, 99:1 er, (*S*)-configuration)].

(*S*)-Diphenyl 3-allyl-2-oxoindoline-1,3-dicarboxylate (10e)[10]

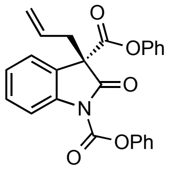

10e

According to general procedure, substrate **9e**[10] (82.7 mg, 0.200 mmol) with a solution of catalyst **1j** (20.0 μL, 50.0 mM in THF, 1.00 μmol) in THF (0.500 mL) at −20 ºC gave a pale yellow solid (82.5 mg, 0.200 mmol, >98% yield): enantiomeric ratio was determined by HPLC with DAICEL CHIRALPAK AD-H® (hexane/*i*-PrOH = 98/2, v/v, flow rate = 1.00 mL/min, 30 °C, UV = 254 nm), T_R = 24.9 min (minor) and T_R = 30.7 min (major), 99:1 er (Supplementary Figure 58); ^{1}H NMR (400 MHz, $CDCl_3$) δ 8.03 (d, *J* = 8.0 Hz, 1H), 7.55–7.39 (m, 5H), 7.55–7.26 (m, 6H), 6.99 (d, *J* = 8.0 Hz , 2H) 5.60–5.45 (m, 1H), 5.18 (dd, *J* = 17.0, 1.4 Hz, 1H), 5.09 (d, *J* = 10.4, 1H), 3.17 (d, *J* = 7.2 Hz); ^{13}C NMR (100 MHz, $CDCl_3$) δ 171.2, 167.1, 150.3, 150.2, 149.3, 139.8, 130.1, 129.7, 129.6, 126.7, 126.5, 126.4, 126.3, 125.7, 123.6, 121.6, 121.3, 121.1, 115.9, 60.2, 39.2; IR (KBr) 3066, 1788, 1751, 1477, 1224, 1159, 750 cm^{-1}; HRMS (FAB^+) calculated for $C_{25}H_{20}NO_5$ $[M+H]^+$ 414.1335, found 414.1329; mp 164.1–165.0 ºC; $[\alpha]^{24}_D$ +67.4 (*c* 0.76, CH_2Cl_2, 99:1 er, (*S*)-configuration) [lit.[10] $[\alpha]^{24}_D$ +29.1 (*c* 0.49, $CHCl_3$, 89:11 er, (*S*)-configuration)].

(*S*)-Diphenyl 3-(but-2-yn-1-yl)-2-oxoindoline-1,3-dicarboxylate (10f)[10]

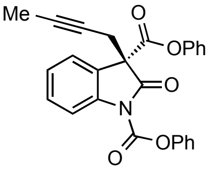

10f

According to general procedure, substrate **9f**[10] (42.0 mg, 0.0987 mmol), THF (0.250 mL) with a solution of catalyst **1j** (10.0 μL, 50.0 mM in THF, 0.500 μmol) at −20 ºC gave a colorless solid (41.7 mg, 0.0980 mmol, >98% yield): enantiomeric ratio was determined by HPLC with DAICEL CHIRALPAK AD-H® (hexane/*i*-PrOH = 85/15, v/v, flow rate = 1.00 mL/min, 30 °C, UV = 254 nm), T_R = 12.4 min (major) and T_R = 37.1 min (minor), 97:3 er (Supplementary Figure 59); ^{1}H NMR (400 MHz, $CDCl_3$) δ 8.06 (d, *J* = 8.0 Hz, 1H), 7.52–7.42 (m, 4H), 7.38–7.28 (m, 6H), 7.23 (t, *J* = 7.6 Hz, 1H), 6.99 (d, *J* = 8.0 Hz, 2H), 3.25 (dd, *J* = 2.3, 2.0 Hz, 2H), 1.64 (t, *J* = 2.3 Hz, 3H); ^{13}C NMR (100 MHz, $CDCl_3$) δ 170.9, 166.5, 150.2, 150.2, 149.3, 140.1, 130.2, 129.7, 129.6, 126.7, 126.4, 125.7, 123.4, 121.6, 121.2, 115.8, 79.5, 71.8, 59.5, 25.8, 3.5; IR (KBr) 2627, 2359, 2340, 1778, 1748, 1732, 1348, 1217, 1157, 689 cm^{-1}; HRMS (EI^+) calculated for $C_{26}H_{19}NO_5$ $[M]^+$ 425.1257, found 425.1257; mp 99.3–100.2 ºC; $[\alpha]^{24}_D$ +9.9 (*c* 0.85, CH_2Cl_2, 97:3 er, (*S*)-configuration) [lit.[10] $[\alpha]^{22}_D$ +17.0 (*c* 0.48, $CHCl_3$, 91:9 er, (*S*)-configuration)].

(*R*)-Diphenyl 3-(2-((methoxycarbonyl)amino)ethyl)-2-oxoindoline-1,3-dicarboxylate (10g)[10]

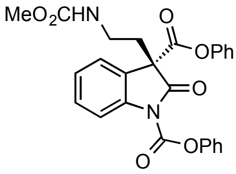

10g

According to general procedure, substrate **9g**[10] (47.4 mg, 0.100 mmol), THF (0.250 mL) with a solution of catalyst **1j** (10.0 μL, 50.0 mM in THF, 0.500 μmol) at −20 ºC gave a pale yellow solid (47.2 mg, 0.0991 mmol, >98% yield): enantiomeric ratio was determined by HPLC with DAICEL CHIRALPAK AD-H® (hexane/*i*-PrOH = 80/20, v/v, flow rate = 1.00 mL/min, 30 °C, UV = 254 nm), T_R = 17.0 min (major) and T_R =

21.6 min (minor), 97:3 er (Supplementary Figure 60); ^{1}H NMR (400 MHz, $CDCl_3$) δ 8.06 (d, *J* = 8.0 Hz, 1H), 7.55–7.40 (m, 4H), 7.39–7.27 (m, 6H), 7.22 (t, *J* = 7.2 Hz, 1H), 6.97 (d, *J* = 8.0 Hz, 2H), 4.81 (bs, 1H), 3.57 (s, 3H), 3.37–3.15 (m, 2H), 2.28–2.75 (m, 1H), 2.67–2.52 (m, 1H); ^{13}C NMR (100 MHz, $CDCl_3$) δ 171.8, 167.1, 156.7, 150.2, 150.1, 149.2, 139.7, 130.3, 129.7, 129.6, 126.7, 126.6, 126.0, 123.4, 121.6, 121.2, 116.2, 58.7, 52.3, 36.7, 34.6; IR (KBr) 3404, 2951, 1748, 1593, 1479, 1350, 1221, 910, 750 cm^{-1}; HRMS (EI^+) calculated for $C_{26}H_{22}N_2O_7$ $[M]^+$ 474.1421, found 474.1409; mp 54.3–55.0 ºC; $[\alpha]^{23}_D$ +68.7 (*c* 0.51, CH_2Cl_2, 97:3 er, (*S*)-configuration) [lit.[10] $[\alpha]^{23}_D$ +47.8 (*c* 0.52, $CHCl_3$, 92:8 er, (*S*)-configuration)].

(*S*)-diphenyl 3-(2-((*tert*-butyldimethylsilyl)oxy)ethyl)-2-oxoindoline-1,3-dicarboxylate (10h)[10]

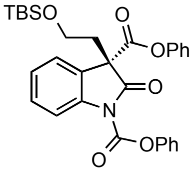

10h

According to general procedure, substrate **10h**[10] (53.0 mg, 0.0997 mmol) with a solution of catalyst **1j** (10.0 μL, 50.0 mM in THF, 0.500 μmol) in THF (0.250 mL) at −20 ºC gave a colorless oil (52.7 mg, 0.0991 mmol, >98% yield): enantiomeric ratio was determined by HPLC with DAICEL CHIRALPAK AD-H® (hexane/*i*-PrOH = 90/10, v/v, flow rate = 1.00 mL/min, 30 °C, UV = 254 nm), T_R = 7.3 min (major) and T_R = 20.4 min (minor), 92:8 er (Supplementary Figure 61); ^{1}H NMR (400 MHz, $CDCl_3$) δ 8.08 (d, *J* = 8.4 Hz, 1H), 7.48–7.46 (m, 3H), 7.38–7.27 (m, 7H), 7.21 (t, *J* = 7.6 Hz, 1H), 6.95 (d, *J* = 7.6 Hz, 2H), 3.71 (ddd, *J* = 10.2, 4.8, 3.1 Hz, 1H), 3.41 (td, *J* = 10.2, 4.8 Hz, 1H), 2.89 (ddd, *J* = 14.9, 10.2, 4.8 Hz, 1H), 2.67 (dt, *J* = 14.9, 3.1 Hz, 1H), 0.76 (s, 9H), −0.12 (s, 3H), −0.15 (s, 3H); ^{13}C NMR (100 MHz, $CDCl_3$) δ 171.7, 167.8, 150.4, 150.3, 149.6, 140.5, 129.9, 129.6, 129.5, 126.5, 126.5, 125.9, 125.2, 123.5, 121.7, 121.3, 116.1, 58.9, 58.8, 36.2, 25.9, 18.4, −5.8, −5.8; IR (KBr) 2930, 1811, 1790, 1748, 1479, 1350, 1186, 752 cm^{-1}; HRMS (ESI^+) calculated for $C_{30}H_{34}NO_6Si$ $[M+H]^+$ 532.2146, found 532.2128; $[\alpha]^{22}_D$ +29.4 (*c* 1.12, CH_2Cl_2, 92:8 er, (*S*)-configuration) [lit.[10] $[\alpha]^{22}_D$ +38.2 (*c* 0.48, $CHCl_3$, 92:8 er, (*S*)-configuration)].

(*S*)-Diphenyl 3-(cyanomethyl)-2-oxoindoline-1,3-dicarboxylate (10i)[10]

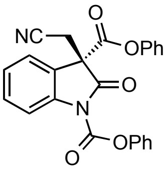

10i

According to general procedure, substrate **9i**[10] (41.0 mg, 0.0994 mmol) with a solution of catalyst **1j** (10.0 μL, 50.0 mM in THF, 0.500 μmol) in THF (0.250 mL) at −20 ºC gave a pale yellow solid (40.8 mg, 0.0989 mmol, >98% yield): enantiomeric ratio was determined by HPLC with DAICEL CHIRALPAK AD-H® (hexane/*i*-PrOH = 82/18, v/v, flow rate = 1.00 mL/min, 30 °C, UV = 254 nm), T_R = 17.6 min (major) and T_R = 19.2 min (minor), 91:9 er (Supplementary Figure 62); ^{1}H NMR (400 MHz, $CDCl_3$) δ 8.12 (d, *J* = 8.4 Hz, 1H), 7.59–7.22 (m, 11H), 6.97 (d, *J* = 8.4 Hz, 2H), 3.51 (d, *J* = 16.8 Hz, 1H), 3.31 (d, *J* = 16.8 Hz, 1H); ^{13}C NMR (100 MHz, $CDCl_3$) δ 169.4, 165.3, 150.0, 150.0, 148.9, 140.0, 131.5, 129.9, 129.8, 127.1, 127.0, 126.4, 124.1, 123.4, 121.5, 121.0, 116.7, 115.0, 56.8, 23.4; IR (KBr) 3059, 2978, 1809, 1782, 1755, 1602, 1481, 1348, 1217, 746, 689 cm^{-1}; HRMS (EI^+) calculated for $C_{24}H_{16}N_2O_5$ $[M]^+$ 412.1053, found 412.1079; mp 130.0–130.9 ºC; $[\alpha]^{21}_D$ +35.2 (*c* 0.50, CH_2Cl_2, 91:9 er, (*S*)-configuration) [lit.[10] $[\alpha]^{24}_D$ +19.3 (*c* 0.52, $CHCl_3$, 99:1 er, (*S*)-configuration)].

(*S*)-Diphenyl 3-phenyl-2-oxoindoline-1,3-dicarboxylate (10j)[9, 10, 16]

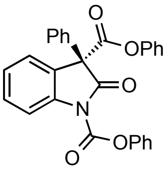

10j

According to general procedure, substrate **9j**[9, 16] (45.2 mg, 0.101 mmol) with a catalyst **1j** (2.90 mg, 3.00 μmol) in THF (0.250 mL) at −20 ºC gave a colorless solid (45.0 mg, 0.100 mmol, >98% yield): enantiomeric ratio was determined by HPLC with DAICEL CHIRALPAK AD-H® (hexane/*i*-PrOH = 90/10, v/v, flow rate = 1.00 mL/min, 30 °C, UV = 254 nm), T_R = 15.7 min (minor) and T_R = 22.9 min (major), 84:16 er (Supplementary Figure 63); ^{1}H NMR (400 MHz, $CDCl_3$) δ 8.15 (d, *J* = 8.4 Hz, 1H), 7.65 (dd, *J* = 7.4, 1.0 Hz, 1H), 7.56 (td, *J* = 7.8, 1.5 Hz, 1H), 7.48–7.20 (m, 14H), 7.04 (d, *J* = 7.6 Hz, 2H); ^{13}C NMR (100 MHz, $CDCl_3$) δ 170.0, 167.2,

150.5. 150.2, 149.5, 140.2, 135.0, 130.6, 129.7, 129.6, 129.1, 129.0, 128.4, 126.7, 126.6, 126.0, 125.8, 125.6, 121.6, 121.2, 116.3, 64.8; IR (KBr) 3043, 2925, 1813, 1749, 1725, 1596, 1492, 1229, 841 cm^{-1}; $[\alpha]^{22}_{D}$ +165.6 (*c* 0.27, CH_2Cl_2, 84:16 er, (*S*)-configuration) [lit.[10] $[\alpha]^{22}_{D}$ +139.5 (*c* 0.49, $CHCl_3$, 92:8 er, (*S*)-configuration)].

(*R*)-diphenyl 2-oxo-3-(thiophen-2-yl)indoline-1,3-dicarboxylate (10k)[10]

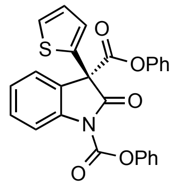

9k

According to general procedure, substrate **9k**[10] (45.3 mg, 0.0995 mmol) with a catalyst **1j** (3.00 mg, 3.12 μmol) in THF (0.250 mL) at −20 ºC gave a brown solid (45.2 mg, 0.0992 mmol, >98% yield): enantiomeric ratio was determined by HPLC with DAICEL CHIRALPAK AD-H® (hexane/*i*-PrOH = 90/10, v/v, flow rate = 1.00 mL/min, 30 °C, UV = 254 nm), T_R = 20.5 min (minor) and T_R = 26.8 min (major), 79:21 er (Supplementary Figure 64); ^{1}H NMR (400 MHz, $CDCl_3$) δ 8.12 (d, *J* = 8.2 Hz, 1H), 7.70 (d, *J* = 8.2 Hz, 1H), 7.54 (t, 8.2 Hz, 1H), 7.49–7.18 (m, 11H), 7.09–6.99 (m, 3 H); ^{13}C NMR (100 MHz, $CDCl_3$) δ 169.3, 166.2, 150.4, 150.1, 149.3, 139.7, 136.6, 130.8, 129.7, 129.6, 128.3, 128.3, 127.5, 127.4, 127.1, 127.1, 126.7, 126.5, 125.8, 125.3, 121.5, 121.2, 116.2, 61.4; IR (KBr) 3108, 2929, 1813, 1749, 1722, 1600, 1491, 846 cm^{-1}; HRMS (ESI^+) calculated for $C_{26}H_{18}NO_5S$ $[M+H]^+$: 456.0821; found: 456.0911; mp 165.8–166.3 ºC; $[\alpha]_D^{21}$ +112.6 (*c* 0.51, CH_2Cl_2, 79:21 er, (*R*)-configuration) [lit.[10] $[\alpha]^{23}_{D}$ +60.4 (*c* 0.48, $CHCl_3$, 99:1 er, (*R*)-configuration)].

(*S*)-Diphenyl 5-bromo-3-methyl-2-oxoindoline-1,3-dicarboxylate (10l)[10]

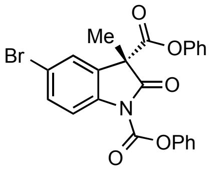

10l

According to general procedure, substrate **9l**[10] (93.5 mg, 0.201 mmol) with a solution of catalyst **1j** (20.0 μL, 50.0 mM in THF, 1.00 μmol) in THF (0.500 mL) at −20 ºC gave a colorless solid (92.5 mg, 0.198 mmol, >98% yield): enantiomeric ratio was determined by HPLC with DAICEL CHIRALPAK AD-H® (hexane/*i*-PrOH = 90/10, v/v, flow rate = 1.00 mL/min, 30 °C, UV = 254 nm), T_R = 13.1 min (minor) and T_R = 17.5 min (major), 92:8 er (Supplementary Figure 65); ^{1}H NMR (400 MHz, $CDCl_3$) δ 7.95 (d, *J* = 8.4 Hz, 1H), 7.60–7.51 (m, 2H), 7.45 (t, *J* = 7.8 Hz, 2H), 7.40–7.20 (m, 6H), 7.00 (dd, *J* = 8.0 Hz, 2H), 1.89 (s, 3H); ^{13}C NMR (100 MHz, $CDCl_3$) δ 171.6, 167.1, 150.3, 150.0, 149.3, 138.2, 133.0, 130.5, 129.8, 129.7, 126.8, 126.7, 126.3, 121.5, 121.2, 118.7, 117.7, 55.9, 21.0; IR (KBr) 3072, 2630, 1813, 1782, 1739, 1475, 1340, 742 cm^{-1}; HRMS (FAB^+) calculated for $C_{23}H_{17}BrNO_5$ $[M+H]^+$ 464.1492, found 464.1482; mp 59.8–60.3 ºC; $[\alpha]^{24}_{D}$ +2.52 (*c* 0.52, CH_2Cl_2, 92:8 er, (*S*)-configuration) [lit.[10] $[\alpha]^{24}_{D}$ +3.90 (*c* 0.50, $CHCl_3$, 90.5:9.5 er, (*S*)-configuration)].

(*S*)-Phenyl 1,3-dimethyl-2-oxoindoline-3-carboxylate (10m)[9, 17]

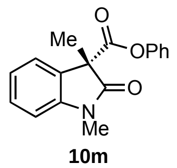

10m

According to general procedure, substrate **9m**[9, 17] (27.9 mg, 0.100 mmol) with a solution of catalyst **1j** (10.0 μL, 50.0 mM in THF, 0.500 μmol) in THF (0.250 mL) at −20 ºC gave a colorless solid (27.9 mg, 0.100 mmol, >98% yield): enantiomeric ratio was determined by HPLC with DAICEL CHIRALPAK AD-H® (hexane/*i*-PrOH = 90/10, v/v, flow rate = 1.00 mL/min, 30 °C, UV = 254 nm), T_R = 9.8 min (minor) and T_R = 15.0 min (major), 87:13 er (Supplementary Fugure 66); ^{1}H NMR (400 MHz, $CDCl_3$) δ 7.46–7.22 (m, 4H), 7.21–7.09 (m, 2H), 6.97–6.88 (m, 3H), 3.29 (s, 3H), 1.76 (s, 3H); ^{13}C NMR (100 MHz, $CDCl_3$) δ 174.8, 168.6, 150.6, 143.9, 129.9, 129.5, 126.2, 123.2, 123.1, 121.4, 108.8, 55.3, 26.8, 20.1; IR (KBr) 1761, 1728, 1610, 1491, 1182, 1086, 743 cm^{-1}; $[\alpha]^{22}_{D}$ +28.4 (*c* 0.50, CH_2Cl_2, 87:13 er, (*S*)-configuration).

(*S*)-Bis(4-methoxyphenyl) 3-methyl-2-oxoindoline-1,3-dicarboxylate (10n)[10]

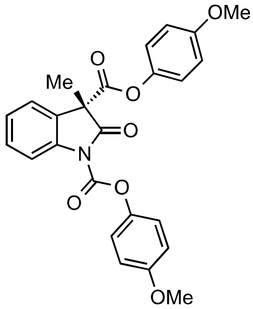

10n

According to general procedure, substrate **9n**[10] (40.1 mg, 0.0993 mmol) with a catalyst **1j** (4.80 mg, 5.00 μmol) in THF (1.00 mL) at −20 ºC gave a brown oil (40.0 mg, 0.0991 mmol, >98% yield): enantiomeric ratio was determined by HPLC with DAICEL CHIRALPAK AS-3® (hexane/*i*-PrOH = 95/5, v/v, flow rate = 0.5 mL/min, 30 °C, UV = 254 nm), T_R = 47.8 min (major) and T_R = 62.8 min (minor), 98:2 er (Supplementary Figure 35); ^{1}H NMR (400 MHz, $CDCl_3$) δ 8.04 (d, *J* = 8.0 Hz, 1H), 7.47–7.38 (m, 2H), 7.32–7.16 (m, 3H), 6.98–6.80 (m, 6H), 3.83 (s, 3H), 3.77 (m, 3H), 1.88 (s, 3H); ^{13}C NMR (100 MHz, $CDCl_3$) δ 172.6, 168.1, 157.9, 157.7, 149.8, 143.8, 143.7, 139.2, 129.9, 128.8, 125.8, 123.0, 122.4, 122.0, 116.0, 114.7, 114.5, 56.0, 55.7, 55.7, 21.0; IR (KBr) 2359, 1807, 1780, 1748, 1715, 1504, 1348, 1221, 1175, 1099, 764 cm^{-1}; HRMS (FAB$^+$) calculated for $C_{25}H_{22}NO_7$ $[M+H]^+$ 448.1390, found 488.1417; $[\alpha]^{24}_D$ +103.3 (*c* 0.25, CH_2Cl_2, 98:2 er, (*S*)-configuration) [lit.[10] $[\alpha]^{24}_D$ +30.5 (*c* 0.45, $CHCl_3$, 91:9 er, (*S*)-configuration)].

(*S*)-Bis(4-fluorophenyl) 3-methyl-2-oxoindoline-1,3-dicarboxylate (10o)[10]

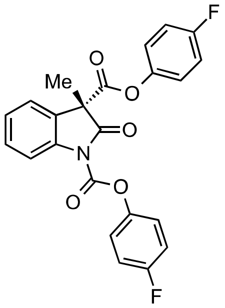

9o

According to general procedure, substrate **9o**[10] (42.9 mg, 0.101 mmol) with a catalyst **1j** (5.00 mg, 5.20 μmol) in THF (1.00 mL) at −20 ºC gave a colorless solid (42.7 mg, 0.101 mmol, >98% yield): enantiomeric ratio was determined by HPLC with DAICEL CHIRALPAK AD-H® (hexane/*i*-PrOH = 97.5/2.5, v/v, flow rate = 1.00 mL/min, 30 °C, UV = 254 nm), T_R = 33.6 min (minor) and T_R = 68.6 min (major), 96:4 er (Supplementary Figure 36); ^{1}H NMR (600 MHz, $CDCl_3$) δ 8.04 (d, *J* = 7.9 Hz, 1H), 7.46 (td, *J* = 7.9, 1.1 Hz, 1H), 7.41 (dd, *J* = 7.9, 1.1 Hz, 1H), 7.38–7.20 (m, 3H), 7.19–6.90 (m, 6H), 1.89 (s, 3H); ^{13}C NMR (100 MHz, $CDCl_3$) δ 172.2, 167.6, 160.6 (d, *J* = 244.4 Hz), 160.5 (d, *J* = 244.4 Hz), 149.2, 145.9 (d, *J* = 13.4 Hz), 138.9, 130.0, 128.6, 125.8, 123.0 (d, *J* = 8.6 Hz), 122.6 (d, *J* = 8.6 Hz), 116.4, 116.3, 116.2, 116.0, 115.9, 55.8, 20.9; IR (KBr) 1780, 1751, 1506, 1179, 831, 768 cm^{-1}; HRMS (FAB$^+$) calculated for $C_{23}H_{16}F_2NO_5$ $[M+H]^+$: 424.0991, found: 424.0995; mp 127.8–128.7 ºC; $[\alpha]^{24}_D$ +73.9 (*c* 0.58, CH_2Cl_2, 96:4 er, (*S*)-configuration) [lit.[10] $[\alpha]^{20}_D$ +57.8 (*c* 0.28, $CHCl_3$, 68:32 er, (*S*)-configuration)].

(*S*)-Dibenzyl 3-methyl-2-oxoindoline-1,3-dicarboxylate (10p)[10, 11]

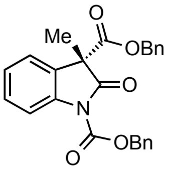

10p

According to general procedure, substrate **9p**[10] (37.1 mg, 0.100 mmol) with a catalyst **1j** (4.80 mg, 5.00 μmol) in THF (1.00 mL) at −20 ºC gave a colorless solid (36.9 mg, 94% conv): enantiomeric ratio was determined by HPLC with DAICEL CHIRALPAK AD-H® (hexane/*i*-PrOH = 97.5/2.5, v/v, flow rate = 1.00 mL/min, 30 °C, UV = 254 nm), T_R = 25.8 min (major) and T_R = 30.6 min (minor), 92:8 er (Supplementary Figure 37); ^{1}H NMR (400 MHz, $CDCl_3$) δ 7.93 (d, *J* = 8.0 Hz, 1H), 7.51 (d, *J* = 7.2 Hz, 2H), 7.44–7.31 (m, 4H), 7.26–7.06 (m, 7H), 5.46 (s, 2H), 5.11 (s, 2H), 1.74 (s, 3H); ^{13}C NMR (100 MHz, $CDCl_3$) δ 173.0, 168.8, 150.8, 139.3, 135.2, 134.9, 129.5, 128.8, 128.6, 128.6, 128.3, 128.2, 127.4, 125.2, 123.1, 115.7, 68.9, 67.6, 55.8, 20.7; IR (KBr) 2359, 1776, 1738, 1288, 1227, 696 cm^{-1}; $[\alpha]^{23}_D$ +26.9 (*c* 0.52, CH_2Cl_2, 92.5:7.5 er, (*S*)-configuration) [lit.[10] $[\alpha]^{23}_D$ +37.6 (*c* 0.13, $CHCl_3$, 88:12 er, (*S*)-configuration)].

(*S*)-dimethyl 3-methyl-2-oxoindoline-1,3-dicarboxylate (10q)

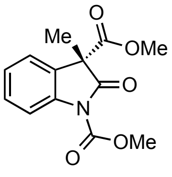

10q

According to general procedure, substrate **9q** (26.1 mg, 0.0991 mmol) with a catalyst **1j** (4.70 mg, 4.89 μmol) in THF (1.00 mL) at −20 ºC gave a colorless solid (25.8 mg, 88% conv): enantiomeric ratio was determined by HPLC with DAICEL CHIRALPAK AD-H® (hexane/*i*-PrOH = 97.5/2.5, v/v, flow rate = 1.00 mL/min, 30 °C, UV = 254 nm), T_R = 14.0 min (major) and T_R = 15.7 min (minor), 95:5 er (Supplementary Figure 38); ^{1}H NMR (400 MHz, $CDCl_3$) δ 7.96 (d, *J* = 7.7 Hz, 1H), 7.38 (td, *J* = 7.7, 1.3 Hz, 1H), 7.28 (dd, *J* = 7.7, 1.3 Hz, 1H), 7.22 (t, *J* = 7.7 Hz, 1H), 4.04 (s, 3H), 3.67 (s, 3H), 1.73 (s, 3H); ^{13}C NMR (100 MHz, $CDCl_3$) δ 173.2, 169.5, 151.4, 139.2, 129.5, 128.9, 125.3, 123.0, 115.6, 55.6, 54.2, 53.4, 21.0; IR (KBr) 1801, 1778, 1742, 1439, 1350, 1244, 770 cm^{-1}; HRMS (FAB$^+$) calculated for $C_{13}H_{14}NO_5$ $[M+H]^+$ 264.0866, found 264.0849; mp 89.3–89.9 ºC; $[\alpha]^{22}_D$ +80.3 (*c* 0.57, CH_2Cl_2, 95:5 er, (*S*)-configuration).

Considerations for the scrambling in Steglich rearrangement

Crossover experiment showed that treatment of 1:1 mixture of enol carbonates **9a** and **9n** using 0.5 mol % **1j** gave **9a** and **9n**; enol carbonates probably **9a′** and **9n′**; **10a** and **10n**; and **10a′**and **10n′** due to scrambling. This reaction was quenched after 30 min and the conversion was 30% that was determined by ^{1}H NMR analysis of unpurified reaction mixture. Consequently, scrambling was occurred rapidly before C-C bond forming step. Same scrambling results were reported in the Steglich rearrangement of azlactone.[18-20]

When the reaction quenched after 5 h, the conversion to products was >98% and the enantiomeric ratios of products were determined by HPLC analysis as 96:4 er (**10a**), 97:3 er (**10n**), 96.5:3.5 er and 97:3 er (**10a′**or **10n′**) respectively. The enantiomeric ratio was determined by HPLC with CHIRALPAK AD-H® (hexane/*i*-PrOH = 92.5/7.5, v/v), flow rate = 1.00 mL/min, 30 °C, UV = 254 nm) (Supplementary Figure 68).

General procedure for kinetic experiment and data in Steglich rearrangement

To a solution of **9a** (112 mg, 0.299 mmol) in THF (0.750 mL) was added a solution of **1j**, **1j′**, or DMAP (50.0 mM, 2.50 μL, 1.50 μmol) in THF at −20 ºC. After stirring the indicated periods in Supplementaly Table 6, the reaction mixture was sucked 100 μL by syringe, poured into 1 M aqueous HCl (2 mL), extracted with EtOAc (3 mL × 3), dried over $MgSO_4$, and concentrated *in vacuo*. The crude product was passed through a short pad of silica gel (eluent: hexane/Et_2O = 1/1, v/v) and ^{1}H NMR and HPLC were measured. The conversion was calculated by ^{1}H NMR analysis and the enantiomeric ratio was determined by HPLC analysis.

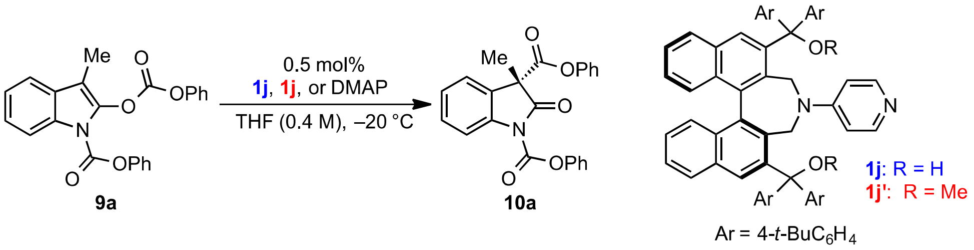

Kinetic resolution of secondary carbinol (Figure 9a)

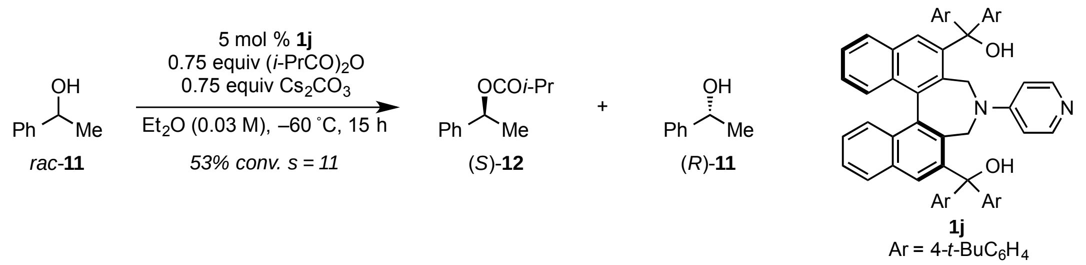

To a solution of racemic **11** (9.20 μL, 76.3 μmol), Cs_2CO_3 (18.7 mg, 57.4 μmol) and **1j** (3.50 mg, 3.64 μmol) in THF (2.25 mL) was added (*i*-PrCO)$_2$O (9.48 μL, 57.2 μmol) at –60 ºC. The reaction mixture was stirred for 15 h at –60 ºC, then MeOH (3 mL) was added to reaction vessel and stirred for 15 min at room temperature. The resulting solution was transferred to other flask using Et_2O to wash the reaction vessel and concentrated in *vacuo*. The residue was through a short pad of silica gel (eluent: hexane/Et_2O = 1/1, v/v) to give the crude mixture (11.9 mg, 53% conv, *s* = 11.4, er of acylate **12** = 84:16, er of substrate **11** = 88:12). The conversion was determined by ^{1}H NMR analysis of the crude mixture and, enantiomeric ratios were determined by HPLC analysis. The conversion (*C*) and selectivity factor (*s*) of kinetic resolution were calculated by following Kagan's equation.[21]

$$s = \frac{\ln[1- C(1+ee')]}{\ln[1- C(1-ee')]} = \frac{\ln[(1-C)(1-ee)]}{\ln[(1-C)(1+ee)]} = \frac{k_{fast}}{k_{slow}}$$

$$ee = enantiomeric\ excess\ measured\ for\ the\ starting\ material$$

$$ee' = enantiomeric\ excess\ measured\ for\ the\ product$$

$$C = \frac{ee}{ee+ee'}100 = conversion$$

Analytical data for 11 and 12

(*R*)-1-Phenylethanol (11)[22]

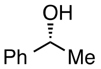

(*R*)-**11**

Enantiomeric ratio was determined by HPLC with DAICEL CHIRALPAK® OJ-H (hexane/*i*-PrOH = 99/1, v/v, flow rate = 0.500 mL/min, 30 °C, UV = 254 nm), T_R = 57.6 min (minor) and T_R = 61.4 min (major), 88:12 er (Supplementary Figure 71); ^{1}H NMR (400 MHz, $CDCl_3$) δ 7.42–7.30 (m, 4H), 7.30–7.21 (m, 1H), 4.91 (q, *J* = 6.5 Hz, 1H), 1.51 (d, *J* = 6.5Hz, 3H); ^{13}C NMR (100 MHz, $CDCl_3$) δ 145.9, 128.5, 127.5, 125.5, 70.4, 25.2; IR (KBr) 3354, 2974, 1454, 1282, 899, 760, 698 cm^{-1}; $[\alpha]^{20}_D$ +12.4 (*c* 0.49, $CHCl_3$, 68:32 er) [lit.[22] $[\alpha]^{20}_D$ +27.0 [*c* 0.270, $CHCl_3$, 38% ee, (*R*) form].

(*S*)-1-Phenylethyl isobutyrate (12)[23]

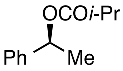

(*S*)-**12**

Enantiomeric ratio was determined by HPLC with DAICEL CHIRALPAK® OJ-H (hexane /*i*-PrOH = 99/1, v/v, flow rate = 0.500 mL/min, 30 °C, UV = 254 nm), T_R = 12.1 min (minor) and T_R = 13.7 min (major), 84:16 er (Supplementary Figure 72); ^{1}H NMR (400 MHz, CD Cl$_3$) 7.40–7.21 (m, 5H), 5.87 (q, *J* = 6.7 Hz, 1H), 2.57 (sep, *J* = 6.9 Hz, 1H), 1.52 (d, *J* = 6.7 H z, 3H), 1.18 (d, *J* = 6.9 Hz, 3H), 1.16 (d, *J* = 6.9 Hz, 3H); ^{13}C NMR (100 MHz, $CDCl_3$) δ 176.4, 142.1, 128.6, 127.8, 126.0, 72.0, 34.2, 22.4, 19.0; IR (KBr) 3034, 2978, 2934, 2876, 1732, 1454,

1192, 1157, 1065, 760, 698 cm^{-1}; $[\alpha]^{22}_{D}$ −29.7 (*c* 1.09, $CHCl_3$, 81:19 er).

Kinetic resolution of racemic 1,2-diol (Figure 9b)

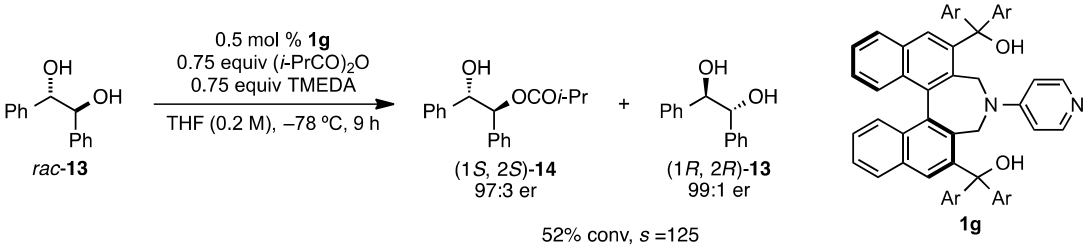

To a solution of racemic **13** (64.1 mg, 0.299 mmol), TMEDA (33.5 μL, 0.225 mmol) and **1g** (1.1 mg, 1.50 μmol) in THF (1.50 mL) was added (i-PrCO)$_2$O (37.3 μL, 0.225 mmol) at –78 ºC. The reaction mixture was stirred for 9 h at –78 ºC, then MeOH (3 mL) was added to reaction vessel and stirred for 15 min at room temperature. The resulting solution was transferred to other flask using Et_2O to wash the reaction vessel and concentrated in *vacuo*. The residue was through a short pad of silica gel (eluent: hexane/Et_2O = 1/1, v/v) to give the crude mixture (75.1 mg, 52% conv, *s* = 125, er of monoacylate **14** = 97:3, er of substrate **13** = 99:1). The conversion was determined by ^{1}H NMR analysis of the crude mixture and, enantiomeric ratios were determined by HPLC analysis. The conversion (*C*) and selectivity factor (*s*) of kinetic resolution were calculated by preceding Kagan's equation.[21]

Analytical data for 13 and 14

(1*R*,2*R*)-hydrobenzoin (13)[24]

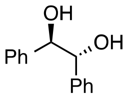
(1*R*, 2*R*)-**13**

Colorless solid. Enantiomeric ratio was determined by HPLC with DAICEL CHIRALPAK® AS-3 (hexane/*i*-PrOH = 97/3, v/v, flow rate = 0.85 mL/min, 30 °C, UV = 254 nm), T_R = 18.9 min (major) and T_R = 21.3 min (minor), 99:1 er (Supplementary Figure 75); ^{1}H NMR (400 MHz, $CDCl_3$+0.03% TMS) δ 7.29–7.19 (m, 6H), 7.17–7.09 (m, 4H), 4.72 (s, 2H), 2.84 (s, 2H); ^{13}C NMR (100 MHz, $CDCl_3$) δ 140.0, 128.2, 128.0, 127.1, 79.2; IR (KBr) 3499, 3395, 2895, 1452, 1198, 1045, 777, 706, 694 cm^{-1}; $[\alpha]^{24}_{D}$ +14.6 (*c* 1.03, EtOH, 72:28 er, (1*R,* 2*R*)-configuration) [lit[24]. $[\alpha]^{28}_{D}$ +92.7 (*c* 1.0, EtOH, 97% ee, (1*R,* 2*R*)-configuration)].

(1*S*,2*S*)-2-hydroxy-1,2-diphenylethyl isobutyrate (14)[25]

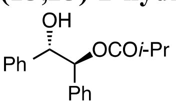
(1*S*, 2*S*)-**14**

Colorless solid. Enantiomeric ratio was determined by HPLC with DAICEL CHIRALPAK® AS-3 (hexane/*i*-PrOH = 95/5, v/v, flow rate = 0.95 mL/min, 30 °C, UV = 254 nm), T_R = 8.8 min (major) and T_R = 11.8min (minor), 97:3 er (Supplementary Figure 76); ^{1}H NMR (400 MHz, $CDCl_3$+0.03% TMS) δ 7.51–7.27 (m, 10H), 7.22 (d, *J* = 7.6 Hz, 1H), 6.97 (d, *J* = 8.0 Hz, 2H), 1.90 (s, 3H); ^{13}C NMR (100 MHz, $CDCl_3$) δ 176.2, 139.2, 137.2, 128.2, 127.1, 79.8, 34.2, 19.1, 19.0; IR (KBr) 3445, 3032, 2972, 1740, 1705, 1456, 1209, 1159, 766, 700 cm^{-1}; $[\alpha]^{22}_{D}$ +4.91 (*c* 1.05, MeOH, 84:16 er (1*S,* 2*S*)-configuration) [lit.[25] $[\alpha]^{22}_{D}$ +1.7 (*c* 1.0, MeOH, 65% ee, (1*S,* 2*S*)-configuration)].

Enantioselective desymmetrization of *meso*-1,2-diol

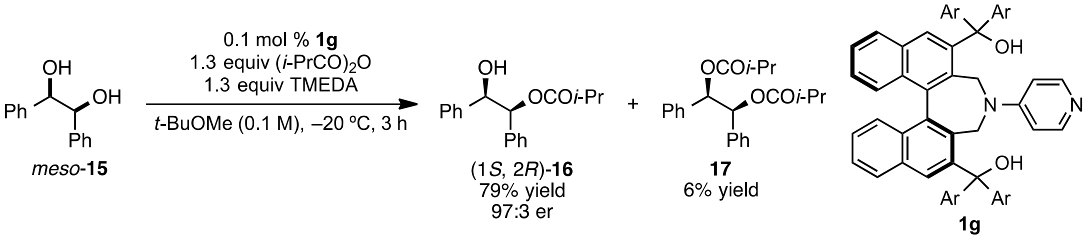

To a reaction vessel was added solution of catalyst **1g** (10.0 mM in chloroform, 40.0 μL, 0.400 μmol) and reaction vessel was evaporated and dried under reduced pressure. The reaction vessel was added *meso*-hydrobenzoin **15** (85.6 mg, 0.400 mmol), Et_3N (72.2 μL, 0.519 mmol) and, TBME (4.00 mL) at room temperature. The mixture was cooled to −20 °C and then isobutyric anhydride (86.2 μL, 0.520 mmol) was added. The reaction mixture was stirred at –20 ºC for 3 h, then methanol was added to reaction vessel and stirred for 1 h at room temperature. The resulting solution was transferred to other flask using Et_2O to wash the reaction vessel and concentrated in *vacuo*. The residue was through a short pad of silica gel (eluent: Et_2O) to dive the crude mixture. The purification of the crude product by flash column chromatography on a silica gel (eluent: hexane/Et_2O = 5/1 to 3/1 to1/1 to Et_2O, v/v) gave the **16** (89.7 mg, 0.315 mmol, 79% yield, 97:3 er), **17** (8.6 mg, 24.3 μmol, 6% yield), recovered substrate **15** (10.3 mg, 0.0481 mmol, 12% yield of recovered).

Analytical data for products *meso*-15–17

***meso*-1,2-diphenylethane-1,2-diol (15)**[12]

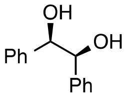

15

Colorless solid. ^{1}H NMR (400 MHz, $CDCl_3$+0.03% TMS) δ 7.35–7.21 (m, 10H), 4.83 (s, 2H), 2.20 (s, 2H); ^{13}C NMR (100 MHz, $CDCl_3$) δ 139.9, 128.3, 128.2, 127.2, 78.2; IR (KBr) 3375, 3321, 2901, 1452, 1279, 1034, 1022, 754, 700 cm^{-1}

(1*S*,2*R*)-2-hydroxy-1,2-diphenylethyl isobutyrate (16)[26]

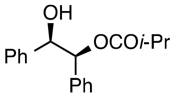

(1*S*, 2*R*)-**16**

Colorless solid. Enantiomeric ratio was determined by HPLC with DAICEL CHIRALPAK® OJ-H (hexane/*i*-PrOH = 98/2, v/v, flow rate = 1.0 mL/min, 30 °C, UV = 254 nm), T_R = 27.6 min (major) and T_R = 37.6 min (minor), 97:3 er (Supplementary Figure 78); ^{1}H NMR (400 MHz, $CDCl_3$+0.03% TMS) δ 7.36–7.24 (m, 10H), 5.90 (d, *J* = 6.5 Hz, 1H), 4.96 (dd, *J* = 6.5, 3.5 Hz, 1H), 2.48 (sep., *J* = 7.0 Hz, 1H), 2.11 (d, *J* = 3.5 Hz, 1H), 1.05 (d, *J* = 7.0 Hz, 3H), 1.01 (d, *J* = 7.0 Hz, 3H); ^{13}C NMR (150 MHz, $CDCl_3$) δ 175.8, 139.8, 137.1, 128.5, 128.4, 128.2, 128.2 (2), 127.7, 127.6, 127.2, 78.5, 76.7, 34.1, 18.8; 3536, 2974, 2866, 1717, 1452, 1389, 1152, 970, 748, 706 cm^{-1}; $[\alpha]^{21}_{D}$ +16.9 (*c* 0.85, EtOAc, 96:4 er, (1*S*, 2*R*)-configuration) [lit.[26] $[\alpha]_D$ +27.2 (*c* 0.85, EtOAc, 99:1 er, (1*S*, 2*R*)-configuration)].

(1*R*,2*S*)-1,2-diphenylethane-1,2-diyl bis(2-methylpropanoate) (17)[26]

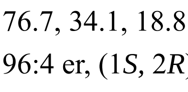

17

Colorless solid. ^{1}H NMR (400 MHz, $CDCl_3$, 0.03% TMS) δ 7.31–7.19 (m, 10H), 6.07 (s, 2H), 2.48 (sep., *J* = 7.0 Hz, 2H), 1.06 (d, *J* = 7.0 Hz, 6H), 1.03 (d, *J* = 7.0 Hz, 6H); ^{13}C NMR (100 MHz, $CDCl_3$) δ 175.5, 136.6, 128.4, 128.2, 127.7, 76.3, 34.2, 18.9, 18.8; IR (KBr) 2974, 2936, 1728, 1458, 1192, 1157, 762, 700 cm^{-1}

Supplementary Reference

1. Frisch, M. J. et al. Gaussian 03, revision C.02. 2004.
2. Becke, A. D. Density-functional exchange-energy approximation with correct asymptotic behavior. *Phys. Rev. A* **38**, 3098-3100 (1988).
3. Lee, C., Yang, W. & Parr, R. G. Development of the colle-salvetti correlation-energy formula into a functional of the electron density. *Phys. Rev. B* **37**, 785-789 (1988).
4. Ooi, T., Kameda, M. & Maruoka, K. Molecular design of a C_2-symmetric chiral phase-transfer catalyst for practical asymmetric synthesis of α-amino acids. *J. Am. Chem. Soc.* **121**, 6519-6520 (1999).
5. Ooi, T., Kameda, M. & Maruoka, K. Design of *N*-spiro C_2-symmetric chiral quaternary ammonium bromides as novel chiral phase-transfer catalysts: Synthesis and application to practical asymmetric synthesis of α-amino acids. *J. Am. Chem. Soc.* **125**, 5139-5151 (2003).
6. Liang, D. C., Luo, R. S., Yin, L. H., Chan, A. S. & Lu, G. A new synthetic route for axially chiral secondary amines with binaphthyl backbone and their applications in asymmetric michael reaction of aldehydes to nitroalkenes. *Org. Biomol. Chem.* **10**, 3071-3079 (2012).
7. Kano, T., Lan, Q., Wang, X. & Maruoka, K. Effects of aromatic substituents on binaphthyl-based chiral spiro-type ammonium salts in asymmetric phase-transfer reactions. *Adv. Synth. Catal.* **349**, 556-560 (2007).
8. Trost, B. M., Zhang, Y. & Zhang, T. Direct N-carbamoylation of 3-monosubstituted oxindoles with alkyl imidazole carboxylates. *J. Org. Chem.* **74**, 5115-5117 (2009).
9. Shaw, S. A. et al. Enantioselective TADMAP-catalyzed carboxyl migration reactions for the synthesis of stereogenic quaternary carbon. *J. Am. Chem. Soc.* **128**, 925-934 (2006).
10. Mandai, H. et al. Enantioselective steglich rearrangement of oxindole derivatives by easily accessible chiral *N,N*-4-(dimethylamino)pyridine derivatives. *Org. Lett.* **17**, 4436-4439 (2015).
11. Rajeswaran, W. G. & Cohen, L. A. Studies on protection of oxindoles. *Tetrahedron* **54**, 11375-11380 (1998).
12. Yamada, S., Misono, T., Iwai, Y., Masumizu, A. & Akiyama, Y. New class of pyridine catalyst having a conformation switch system: Asymmetric acylation of various *sec*-alcohols. *J. Org. Chem.* **71**, 6872-6880 (2006).
13. Qian, C. T., Huang, T. S., Zhu, C. J. & Sun, J. Synthesis of 3,3 '-, 6,6 '- and 3,3 ',6,6 '-substituted binaphthols and their application in the asymmetric hydrophosphonylation of aldehydes–an obvious effect of substituents of BINOL on the enantioselectivity. *J. Chem. Soc., Perkin Trans. 1* 2097-2103 (1998).
14. Martin, R., Mathieu, V., Sorokin, V., Bachawala, P. & Mazur, W. A., inventors; *N*-Phenyl carbazole-based host material for light-emitting diodes patent WO 2010/043691, April 22, 2010.
15. Zhang, Y., Lu, Z., Desai, A. & Wulff, W. D. Mapping the active site in a chemzyme: Diversity in the *N*-substituent in the catalytic asymmetric aziridination of imines. *Org. Lett.* **10**, 5429-5432 (2008).
16. Shaw, S. A., Aleman, P. & Vedejs, E. Development of chiral nucleophilic pyridine catalysts: Applications in asymmetric quaternary carbon synthesis. *J. Am. Chem. Soc.* **125**, 13368-13369

(2003).

17. Thomson, J. E. et al. *N*-Heterocyclic carbene catalysed oxygen-to-carbon carboxyl transfer of indolyl and benzofuranyl carbonates. *Synthesis* 2805-2818 (2008).

18. Ruble, J. C. & Fu, G. C. Enantioselective construction of quaternary stereocenters: Rearrangements of *O*-acylated azlactones catalyzed by a planar-chiral derivative of 4-(pyrrolidino)pyridine. *J. Am. Chem. Soc.* **120**, 11532-11533 (1998).

19. Campbell, C. D., Concellón, C. & Smith, A. D. Catalytic enantioselective steglich rearrangements using chiral *N*-heterocyclic carbenes. *Tetrahedron: Asymmetry* **22**, 797-811 (2011).

20. Joannesse, C. et al. Isothiourea-mediated asymmetric *O*- to *C*-carboxyl transfer of oxazolyl carbonates: Structure-selectivity profiles and mechanistic studies. *Chem. Eur. J.* **18**, 2398-2408 (2012).

21. Kagan, H. B. & Fiaud, J. C. Kinetic resolution. *Top. Stereochem.* **18**, 249-330 (1988).

22. Rendler, S. & Oestreich, M. Conclusive evidence for an S_N2-Si mechanism in the $B(C_6F_5)_3$-catalyzed hydrosilylation of carbonyl compounds: Implications for the related hydrogenation. *Angew. Chem. Int. Ed. Engl.* **47**, 5997-6000 (2008).

23. Klomp, D. et al. Combined epimerisation and acylation: Meerwein-Ponndorf-Verley-Oppenauer catalysts in action. *Org. Biomol. Chem.* **3**, 483-489 (2005).

24. Takenaka, N., Xia, G. & Yamamoto, H. Catalytic, highly enantio- and diastereoselective pinacol coupling reaction with a new tethered bis(8-quinolinolato) ligand. *J. Am. Chem. Soc.* **126**, 13198-13199 (2004).

25. Matsumura, Y., Maki, T., Murakami, S. & Onomura, O. Copper ion-induced activation and asymmetric benzoylation of 1,2-diols: Kinetic chiral molecular recognition. *J. Am. Chem. Soc.* **125**, 2052-2053 (2003).

26. Anju, K. S., Ramakrishnan, S., Thomas, A. P., Suresh, E. & Srinivasan, A. 9,10,19,20-Tetraarylporphycenes. *Org. Lett.* **10**, 5545-5548 (2008).
